# Collagenous gastritis: current understanding of a rare immune-mediated gastropathy across pediatric and adult phenotypes—from pathogenesis to therapeutic strategies

**DOI:** 10.3389/fcell.2026.1847333

**Published:** 2026-07-30

**Authors:** Tai Zhang, Ting Chen, Beihua Zhang, Xudong Tang

**Affiliations:** 1 Peking University Traditional Chinese Medicine Clinical Medical School (Xiyuan), Peking University Health Science Center, Beijing, China; 2 Peking University Health Science Center, Beijing, China; 3 Institute of Digestive Diseases, Xiyuan Hospital, China Academy of Chinese Medical Sciences, Beijing, China; 4 Department of Gastroenterology, Xiyuan Hospital, China Academy of Chinese Medical Sciences, Beijing, China

**Keywords:** autoimmunity, collagenous colitis, collagenous gastritis, collagenous gastroenteropathy, iron-deficiency anemia

## Abstract

Collagenous gastritis (CG) is a rare chronic inflammatory disorder defined histologically by a subepithelial collagen band exceeding 10 μm, together with a chronic inflammatory infiltrate within the lamina propria. First described in 1989, CG presents with a striking age-stratified dichotomy. The pediatric phenotype is dominated by treatment-refractory iron-deficiency anemia and chronic abdominal pain, with disease usually confined to the stomach. The adult phenotype is dominated by chronic watery diarrhea and frequently coexists with collagenous colitis as part of a broader collagenous gastroenteropathy spectrum. The pathogenesis is widely held to be immune-mediated. Strong associations exist with autoimmune conditions, including celiac disease, common variable immunodeficiency (CVID), and systemic lupus erythematosus (SLE). Recent gene-expression and single-cell studies have identified mixed T-helper 1 (Th1) and T-helper 2 (Th2) cytokine profiles in gastric tissue, together with α4β7-mediated mucosal homing of activated CD4^+^ T cells, suggesting complex immune dysregulation rather than a primary disorder of collagen biosynthesis. Implicated triggers include certain medications—notably olmesartan—and, more speculatively, infectious agents; a single case report has increased the possibility of Epstein–Barr virus (EBV) reactivation. A single proteomics study has identified reduced epidermal growth factor (EGF) expression as a candidate biomarker, suggesting impaired mucosal repair, although further validation is needed. Diagnosis demands a high index of clinical suspicion and rests on histopathological examination of multi-site gastric biopsies since endoscopic appearances range from normal mucosa to characteristic nodular patterns. Management is empirical and individualized: symptomatic support with proton pump inhibitors (PPIs) and iron supplementation, anti-inflammatory therapy with topically targeted budesonide, dietary intervention in selected patients, and emerging mechanism-targeted approaches including α4β7 blockade. This narrative review synthesizes 101 articles published between 1989 and 31 August 2025, describing approximately 730 histopathologically confirmed CG cases (≈40% pediatric, ≈60% adult; overall female-to-male ratio ≈2.1:1). Replicated findings are distinguished throughout from those based on single reports, with preliminary observations explicitly flagged as hypothesis-generating.

## 1 Introduction

Collagenous gastritis (CG) is a rare and enigmatic entity within the spectrum of chronic inflammatory diseases of the gastrointestinal (GI) tract. Colletti and Trainer first described it in 1989 as focal subepithelial collagen thickening in the gastric biopsy of a 15-year-old girl with GI bleeding and abdominal discomfort (Kamimura et al., 2015; Mandaliya et al., 2013). The disorder is defined histopathologically by a thick, irregular subepithelial collagen band (typically >10 μm), accompanied by a prominent inflammatory infiltrate within the lamina propria. Despite this distinct histological signature, CG remains a diagnostic challenge because of its low prevalence, non-specific symptoms, and variable endoscopic appearance.

CG belongs to a broader family of collagenous gastrointestinal disorders that can affect the colon (collagenous colitis), small intestine (collagenous sprue), and ileum (collagenous ileitis), all sharing the dual hallmarks of subepithelial collagen deposition and chronic mucosal inflammation (Gopal and McKenna, 2010; O'Brien et al., 2011). Collagenous colitis is, by far, the most common member of this family (Camarero et al., 2003; Freeman, 2008), whereas CG and collagenous sprue—which involve the proximal gastrointestinal tract—are considerably rarer.

Clinically, CG presents with a striking age-stratified dichotomy. In children and adolescents, the disorder typically causes profound, treatment-refractory iron-deficiency anemia and persistent unexplained abdominal pain, often precipitating extensive investigation before the correct diagnosis is established (Zheng et al., 2022; Illan Montero et al., 2023; Hayashi et al., 2018; Kozai et al., 2023). In adults, by contrast, chronic watery diarrhea predominates—a profile that overlaps substantially with collagenous colitis (Suskind et al., 2009; Kagihara et al., 2023; Cai et al., 2023; Kiryukhin et al., 2024).

The etiology and pathogenesis of CG are not fully understood. Three pathogenic frameworks have been proposed—an inflammation-and-repair model, a plasma-protein leakage model, and a model of aberrant fibroblast proliferation—but a compelling body of clinical and immunological evidence places immune-mediated mechanisms at the center of the disorder. CG is strongly associated with autoimmune and immunodeficiency conditions, including celiac disease, common variable immunodeficiency (CVID), and systemic lupus erythematosus (SLE), suggesting a predisposition rooted in systemic immune dysregulation (Ma et al., 2015; Macaigne et al., 2010; Mandaliya et al., 2016). External triggers, notably certain medications and—more speculatively—infectious agents, have been implicated in initiating or unmasking the disease in susceptible individuals (Varelas et al., 2022; Duncan et al., 2024).

Definitive diagnosis requires histopathological examination of gastric biopsies, and management is empirical owing to both the rarity of the disease and the absence of large-scale clinical trials. Treatment is tailored to symptom burden and disease severity, ranging from symptomatic support with proton pump inhibitors (PPIs) and iron supplementation to anti-inflammatory and immunomodulatory therapy (Macaigne et al., 2010; Rosell-Camps et al., 2015). Although focused case series and topical reviews have been published (Isoldi et al., 2024; Camar et al., 2011; Wu et al., 2023; De Ronde et al., 2020), an updated synthesis integrating recent immunological, single-cell transcriptomic, and therapeutic developments is currently lacking.

This review aims to fill that gap. We integrate (i) the single-cell transcriptomic and T-helper 1/ T-helper 2 (Th1/Th2) cytokine-profiling data, (ii) explicit mechanistic distinctions between the pediatric and adult phenotypes, (iii) an evidence-graded stepwise treatment algorithm with dosing recommendations, and (iv) a discussion of candidate molecular targets—most notably the α4β7–MAdCAM-1 axis and epidermal growth factor (EGF) signaling—relevant to future therapeutic translation.

## 2 Study framework

This article is a non-systematic, narrative review prepared in line with the Scale for the Assessment of Narrative Review Articles (SANRA) (Baethge et al., 2019). Because the available evidence is dominated by individual case reports, small case series, and a few moderate-sized retrospective cohorts, we favored the broad, interpretive scope of a narrative format over the quantitative pooling characteristic of systematic reviews. No protocolized eligibility framework, formal risk-of-bias appraisal, or PRISMA-guided study selection was performed; instead, the methodology is rendered transparent so that readers may appraise the evidentiary basis of each claim.

We surveyed PubMed, SCOPUS, Web of Science, and Google Scholar from 1989—the year of the original Colletti and Trainer description—through 31 August 2025. Search terms, developed iteratively by the author team, included “collagenous gastritis,” “microscopic gastritis,” “subepithelial collagen band,” “collagenous gastropathy,” “gastric nodularity,” “nodular gastritis,” “gastric nodules,” “subepithelial thickening,” and “subepithelial collagen thickening.” Articles were selected by author consensus based on relevance to the clinical and pathological characterization of CG, with priority given to publications providing histopathologically confirmed cases (subepithelial collagen band >10 μm with chronic lamina-propria inflammation) and sufficient clinical detail to inform the present synthesis.

Of the records initially identified (PubMed, n = 117; SCOPUS, n = 110; Web of Science, n = 95; Google Scholar, n = 78), duplicate removal and review of title, abstract, and full text yielded 101 articles for final inclusion. These comprised 73 single-patient case reports, 14 small case series (2–10 patients), 13 retrospective or registry-based cohort studies (>10 patients), and one systematic scoping review. By population, 44 articles reported exclusively pediatric cohorts, 47 exclusively adult cohorts, 9 mixed pediatric–adult cohorts, and one was the scoping review of Kozai et al. (2023).

Across the 101 included articles, approximately 730 unique histopathologically confirmed cases were synthesized, excluding patients pooled within the scoping review to avoid double-counting. Of these, ≈295 (40%) were pediatric (≤18 years), and ≈435 (60%) were adult (>18 years). The overall female-to-male ratio was approximately 2.1:1, broadly consistent with the female predominance documented in the principal cohorts: 123F:44M; F:M ≈ 2.8:1 (Genta et al., 2021), 145F:59M; F:M ≈ 2.5:1 (Genta and Rugge, 2024), 26F:14M; F:M ≈ 1.9:1 (Arnason et al., 2015), 42F:22M; F:M ≈ 1.9:1 (Choung et al., 2022), 23F:17M; F:M ≈ 1.4:1 (Beinvogl et al., 2021), and 12F:3M; F:M = 4:1 (Käppi et al., 2020).

The median age at diagnosis was approximately 18 years (range, 9 months–89 years), reflecting a bimodal distribution: a first peak in the second decade of life, dominated by the pediatric phenotype with iron-deficiency anemia and abdominal pain, and a second, broader peak in patients aged ≥60 years, dominated by the adult phenotype with chronic watery diarrhea and frequent autoimmune comorbidities. Female predominance was apparent across both age peaks and was most pronounced in the older adult subgroup, consistent with the increased burden of associated systemic autoimmune disease in this population. Supplementary Table S1 summarizes patient-level demographics, clinical presentation, endoscopic appearance, and histological features.

Throughout the review, findings supported by replicated observations across multiple cohorts are distinguished from mechanistic claims derived from single case reports or unreplicated studies; the latter are explicitly identified as preliminary or hypothesis-generating wherever they appear.

## 3 Histopathology and pathophysiology of CG

The histopathological diagnosis of CG is based on three obligatory features: (i) a subepithelial collagen band exceeding 10 μm in thickness; (ii) a chronic inflammatory infiltrate within the lamina propria, typically lymphoplasmacytic with a variable eosinophilic component; and (iii) entrapment of capillaries, fibroblasts, and inflammatory cells within the deposited collagen matrix—descriptively termed “trapillaries” (a portmanteau of “trapped capillaries”). Mechanistically, the convergent weight of clinical, histological, immunohistochemical, and limited molecular evidence favors CG as a reactive, immune-mediated process rather than a primary disorder of collagen biosynthesis. Three non-exclusive contributors are implicated: mixed Th1/Th2 cytokine signaling, eosinophil activation with degranulation, and dysregulated mucosal repair.

### 3.1 Defining histological features

The sine *qua non* for the diagnosis of CG is a thickened, irregular, hyaline-like collagen band deposited directly beneath the surface epithelium of the gastric mucosa (Kamimura et al., 2015). The normal subepithelial collagen layer measures 0.30–2.81 μm (mean ≈1.33 μm), whereas reported mean band thicknesses in CG range from 27 to 37 μm—more than a 20-fold increase (Côté et al., 1998; Kajino et al., 2003; Park et al., 2005; Soeda et al., 2014; Nielsen et al., 2014)—with focal areas frequently exceeding 50–100 μm and a documented maximum of 225 μm (Kajino et al., 2003; Vesoulis et al., 2000; Winslow et al., 2001). The band most commonly exhibits either pan-gastric distribution or corpus-predominant involvement in roughly equal proportions (Arnason et al., 2015).

#### 3.1.1 Morphological characteristics and distribution patterns

Subepithelial collagen deposition in CG is heterogeneous in both thickness and distribution, manifesting in either diffuse or patchy configurations. Notably, collagen-band thickness correlates more closely with biopsy location than with disease duration or severity, which underscores the importance of obtaining multiple biopsies from various gastric regions—including macroscopically normal-appearing tissue—to minimize the sampling error (Arnason et al., 2015).

The collagen band is characteristically irregular and focal rather than uniform, with a ragged or serrated deeper margin. At this lower edge, the band engulfs adjacent lamina-propria elements, producing the pathognomonic ultrastructural signature of CG: dilated, partially obliterated capillaries embedded—together with adjacent fibroblasts and inflammatory cells—within the dense subepithelial collagen matrix. These trapillaries distinguish CG from amyloidosis, ischemic injury, and other causes of subepithelial hyalinization (Romano et al., 2023; Evans et al., 2023). Surface epithelial injury frequently accompanies the deposition, manifesting as flaking, denudation, or frank disruption of the overlying epithelium.

On hematoxylin and eosin (H&E) staining, the collagen band appears as a homogeneous, densely eosinophilic subepithelial structure, with the trapillaries serving as the defining diagnostic clue at the light-microscopic level (Kajino et al., 2003; Stancu et al., 2001). Specialized stains are, nonetheless, indispensable: trichrome-based methods (Masson’s trichrome and Azan) accentuate the collagen fibers, and combined trichrome positivity with negative Congo red staining definitively excludes amyloid deposition.

Immunohistochemical collagen subtyping has yielded mechanistically informative findings. Across multiple investigations, immunoreactivity for collagen type IV—the network-forming collagen of the gastrointestinal basement membrane—is consistently absent within the pathological band, indicating that collagen accumulates beneath, rather than within, the basement membrane (Brain et al., 2009; Pompili et al., 2021; Aigner et al., 1997). The interstitial fibrillar collagens, by contrast, show heterogeneous positivity in CG specimens: type I expression is variable, while types III and VI are more frequently detected, often together with the extracellular matrix glycoprotein tenascin (Wu et al., 2023; Arnason et al., 2015; Kajino et al., 2003; Winslow et al., 2001; Brain et al., 2009; Castellano et al., 1999). This pattern contrasts with collagenous colitis, in which types I, III, and VI are co-expressed uniformly throughout a continuous band (Aigner et al., 1997). Because type III collagen and tenascin are characteristically secreted by activated subepithelial myofibroblasts during tissue repair, their detection within the CG band is most compatible with a dysregulated reparative response to chronic inflammation, rather than with a primary disorder of collagen biosynthesis (Arnason et al., 2015). The hallmark subepithelial deposit is, therefore, best regarded as a secondary manifestation of sustained inflammatory damage rather than the inciting pathogenic event.

#### 3.1.2 Inflammatory component and cellular infiltrate

Beyond the diagnostic collagen band, the gastric lamina propria commonly demonstrates marked expansion by a dense inflammatory infiltrate enriched in lymphocytes and plasma cells, with variable eosinophilic prominence (Zhang et al., 2010). A subset of patients display prominent eosinophilic infiltration, which can occasionally prompt initial misdiagnosis as eosinophilic gastritis when the subepithelial collagen deposition is focal or inconspicuous (Côté et al., 1998; Zhang et al., 2010). Electron microscopy has added two findings beyond those visible on light microscopy: many of the eosinophils and mast cells embedded within the trapillary matrix exhibit active degranulation—indicating ongoing release of inflammatory mediators within the band itself—and focal thickening of the basement membrane surrounds atrophic glands and small blood vessels (Li et al., 2025). Pericryptal and subepithelial fibroblasts appear morphologically unremarkable on ultrastructural examination, which may suggest that excessive collagen output, rather than fibroblast malformation, contributes to the deposition (Li et al., 2025).

The overlying surface epithelium manifests multiple forms of injury: detachment from the basement membrane, cellular flattening with diminished mucin content, widening of intercellular spaces, and regenerative changes. An increased density of intraepithelial lymphocytes is frequent and, when sufficiently prominent, justifies a concurrent diagnosis of lymphocytic gastritis (Stancu et al., 2001). Topographic mapping using multi-site biopsies has demonstrated that CG involvement is frequently diffuse or corpus-predominant, affecting both the gastric body and antrum, albeit unevenly (Freeman, 2001).

Three histological subtypes of CG have been delineated based on the predominant inflammatory pattern. These patterns may overlap, and individual patients may demonstrate multiple features within a single case (Arnason et al., 2015). Their defining criteria, relative frequency, and clinical correlates are summarized in Table 1.

**TABLE 1:** Histological subtypes of collagenous gastritis: defining criteria, relative frequency, predominant cytokine signature, and clinical correlates.

Subtype	Defining histological criterion	Relative frequency	Predominant cytokine signature	Distinctive clinical correlates
Eosinophil-rich	Prominent eosinophilic infiltrate in the lamina propria (no universally established threshold; ≥30 eosinophils per high-power field in ≥5 HPFs has been applied when concurrent eosinophilic gastritis is considered)	Likely underrecognized; may overlap with the lymphocytic and atrophic patterns	Not subtype-specific; CG overall shows combined Th1/Th2 activation, with Th2 cytokines (IL-5, IL-13, and IL-4) potentially relevant to eosinophil recruitment	Diagnostic confusion with eosinophilic gastritis; emerging evidence that adult-onset CG and eosinophilic gastritis may exist on a spectrum (Shahrvini et al., 2026)
Lymphocytic-gastritis-like	Increased intraepithelial lymphocytes (commonly referencing the threshold of ≥25 per 100 surface epithelial cells used for lymphocytic gastritis, although no CG-specific cut-off is established)	Reported but not quantified	Not subtype-specific; CG overall shows combined Th1/Th2 activation	Strong association with celiac disease (including atypical forms) and autoimmune comorbidities
Atrophic	Marked corpus glandular loss (parietal cell loss), with or without pyloric metaplasia; intestinal metaplasia and enterochromaffin-like (ECL) cell hyperplasia are typically absent and reported only rarely on long-term follow-up	Most commonly encountered single subtype	Not subtype-specific; CG overall shows combined Th1/Th2 activation and reduced EGF expression	Often associated with isolated gastric involvement, treatment refractoriness, and rare progression to intestinal metaplasia, ECL cell hyperplasia, or epithelial changes indeterminate for dysplasia (Winslow et al., 2001; Anderson and Ravi, 2019; Patel et al., 2023)

Among these, the atrophic pattern is the most common single subtype and frequently associates with isolated gastric involvement, low rates of clinical remission, and rare histologic remission (Anderson and Ravi, 2019; Patel et al., 2023). Anatomically, glandular atrophy is confined to the corpus in roughly two-thirds of atrophic CG cases, with the remainder showing diffuse antro-corpus involvement (Li et al., 2025). Intestinal metaplasia is generally absent—an important distinction from autoimmune gastritis. Although uncommon, longitudinal observation of corpus-predominant atrophic CG has documented progression to neuroendocrine cell hyperplasia, mild-to-moderate glandular atrophy with emergent intestinal metaplasia, and reactive epithelial changes indeterminate for dysplasia (Winslow et al., 2001). These observations support targeted endoscopic surveillance in this subtype.

#### 3.1.3 Relationship to collagenous gastroenteropathies

A note on terminology is warranted. CG refers strictly to subepithelial collagen-band deposition (>10 μm) confined to the gastric mucosa. Collagenous gastroenteritis denotes a more extensive disorder, in which analogous histological changes simultaneously affect two or more anatomically contiguous segments—most commonly the stomach, together with the duodenum, small bowel, or colon. Although CG and collagenous gastroenteritis are presumed to lie on a shared etiological spectrum, they behave as clinically distinguishable phenotypes: isolated CG predominates in children and frequently presents as treatment-refractory iron-deficiency anemia and abdominal pain, whereas collagenous gastroenteritis has been proposed to predominate in adults, often featuring chronic watery diarrhea, malabsorption, weight loss, and systemic immune-mediated comorbidities. The umbrella term collagenous gastroenteropathy encompasses the entire family of these conditions irrespective of anatomical distribution. Throughout this review, “CG” is reserved for isolated gastric disease, and “collagenous gastroenteritis” or “collagenous gastroenteropathy” is used only when referring to multi-segment involvement.

The pattern of shared features differs in instructive ways across anatomical sites. Collagenous colitis typically exhibits a relatively diffuse, continuous, and uniform collagen band with strong, homogeneous immunoreactivity for collagen types I, III, and VI (44). CG, by contrast, demonstrates a patchy, heterogeneous distribution and the variable positivity for collagen types III and VI staining pattern described above (Wu et al., 2023; Kajino et al., 2003; Winslow et al., 2001; Brain et al., 2009; Castellano et al., 1999). When the two conditions coexist in the same patient, this divergent histology is preserved: gastric biopsies remain heterogeneous and focal even when synchronous colonic biopsies show diffuse, continuous deposition (Brain et al., 2009). Pediatric and adult cases show no consistent differences in mean collagen-band thickness, gastric site of involvement, or eosinophilic prominence (Brain et al., 2009).

#### 3.1.4 Diagnostic considerations and biomarker potential

Detailed histopathological evaluation is crucial for distinguishing CG from other gastropathies, as illustrated by cases in which findings on partial gastrectomy specimens have clarified the diagnosis (Almazeedi et al., 2013). Careful assessment of inflammatory cell composition and the specific location of mucosal injury is, therefore, central to the diagnostic process (Hissong et al., 2025). In the search for non-invasive diagnostic adjuncts, a single proteomic and immunoassay study has reported reduced expression of EGF in the serum of patients with CG compared with patients with non-collagenous gastritis, increasing the possibility that EGF could serve as a candidate biomarker, reflecting impaired mucosal repair (Curci et al., 2022). Whether this finding represents a CG-specific signature or a non-specific consequence of chronic gastric inflammation cannot yet be determined.

### 3.2 Pathophysiological mechanisms: an immunological perspective

**FIGURE 1:**
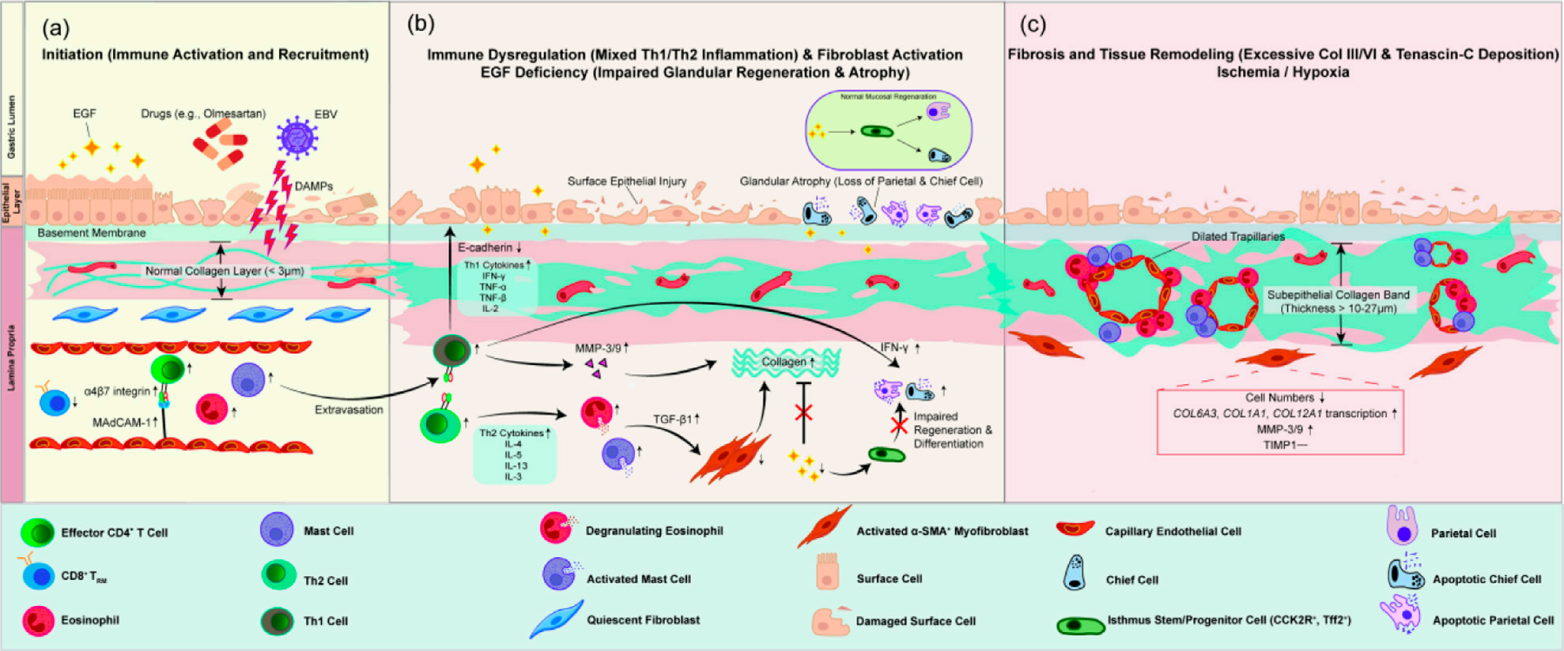
Proposed model of immunopathogenesis and tissue remodeling in collagenous gastritis. This schematic depicts three sequential, interconnected stages of collagenous gastritis (CG) pathogenesis. **(a)** Initiation—immune activation and recruitment. Putative triggers—including drugs (e.g., olmesartan), viral agents (e.g., EBV), or other environmental factors—induce epithelial stress and release of damage-associated molecular patterns (DAMPs). In the lamina propria, endothelial cells upregulate MAdCAM-1, while circulating T cells express elevated α4β7 integrin, facilitating selective extravasation into the gastric mucosa. Tissue-resident memory CD8^+^ T cells are progressively displaced by newly recruited effector T lymphocytes. The normal subepithelial collagen layer (<3 μm) remains intact at this stage. **(b)** Immune dysregulation, fibroblast activation, and impaired glandular regeneration. Infiltrating CD4^+^ T cells co-polarize into Th1 and Th2 subsets. Th1 cells secrete IFN-γ, TNF-α/β, and IL-2, which downregulate E-cadherin on surface epithelial cells, causing epithelial injury. IFN-γ also directly damages parietal and chief cells, leading to glandular atrophy and impairing normal mucosal regeneration by isthmus stem/progenitor cells (CCK2R^+^ and Tff2^+^). Th2 cells secrete IL-4, IL-5, IL-13, and IL-3, recruiting and activating eosinophils and mast cells. Degranulating eosinophils release TGF-β1, which transforms quiescent fibroblasts into activated α-SMA^+^ myofibroblasts that produce excess collagen. MMP-3/9 is upregulated in an attempt to remodel the matrix, but TGF-β1-driven collagen synthesis overwhelms degradation, resulting in net collagen accumulation. Concurrently, EGF deficiency impairs the repair capacity of damaged gastric epithelial cells, preventing effective wound closure and diminishing the inhibition of collagen deposition, thereby indirectly promoting fibrosis. **(c)** Fibrosis and tissue remodeling—ischemia/hypoxia. Progressive fibrosis produces the diagnostic subepithelial collagen band (>10–27 μm) composed of collagen III/VI and tenascin-C. Despite reduced fibroblast numbers, remaining cells markedly upregulate COL6A3, COL1A1, and COL12A1 transcription, while TIMP1 remains unchanged. The thickened collagen band physically separates the epithelium from submucosal capillaries, which become dilated, creating relative tissue ischemia/hypoxia that further compromises epithelial renewal and perpetuates a self-reinforcing cycle of injury and fibrosis. Abbreviations: DAMPs, damage-associated molecular patterns; EGF, epidermal growth factor; EBV, Epstein–Barr virus; MAdCAM-1, mucosal addressin cell adhesion molecule-1; α-SMA, alpha-smooth muscle actin; MMP, matrix metalloproteinase; TIMP1, tissue inhibitor of metalloproteinase-1.

Although the precise etiology of CG remains unknown, the prevailing hypothesis centers on a dysregulated immune response as the principal driver of disease (Figure 1). This framework is supported by strong clinical associations with autoimmune disease, the lymphoplasmacytic and eosinophilic characteristics of the inflammatory infiltrate, characteristic local cytokine and cellular profiles, and the partial—albeit incomplete—response to immunomodulatory therapy.

#### 3.2.1 Autoimmune associations

A substantial body of the literature provides circumstantial but cumulatively compelling evidence for an immunological basis underlying CG, derived from frequent associations with autoimmune and immune-mediated conditions. The breadth of these associations is summarized in Table 2 and ranges from organ-specific endocrine disease (Hashimoto thyroiditis, autoimmune hypothyroidism, and type 1 diabetes mellitus) through systemic connective-tissue disorders (SLE, Sjögren syndrome, and scleroderma) to humoral and cellular immunodeficiency states (CVID, selective IgM deficiency, IgA deficiency, hypogammaglobulinemia, and HIV).

**TABLE 2:** Reported autoimmune, immune-mediated, and immunodeficiency associations in collagenous gastritis. Frequencies, where available, derive from the largest registry-based and retrospective cohorts; case-level associations are noted as such.

Category	Specific condition	Cohort prevalence (where reported)	Case-level report	Key reference
Organ-specific autoimmunity (gastrointestinal)	Celiac disease	4.2% (7/168) in Genta et al. (2021); 4.5% (4/88) in the pooled scoping review	Multiple	Genta et al. (2021), Freeman (2009), and Varela Rey et al. (2019)
	Lymphocytic gastritis	4.2% (7/168) in Genta et al. (2021)	Multiple	Genta et al. (2021) and Stancu et al. (2001)
	Autoimmune (atrophic) gastritis	7.1% (12/168) in Genta et al. (2021)	Multiple	Genta et al. (2021) and Lenti et al. (2025)
	Microscopic colitis (collagenous + lymphocytic)	21.2% of CG patients with same-day colonoscopy in Genta et al. (2024)	Multiple	Kiryukhin et al. (2024), Camar et al. (2011), Genta et al. (2021), Ziaei et al. (2022), and Genta and Rugge (2024)
	Inflammatory bowel disease	Sporadic	Yes	Matta et al. (2018)
Organ-specific autoimmunity (endocrine)	Hashimoto thyroiditis/autoimmune hypothyroidism	Sporadic across cohorts	Multiple	Käppi et al. (2020) and Ankamah and Aaron (2022)
	Type 1 diabetes mellitus	Sporadic	Yes	Käppi et al. (2020) and Shahrvini et al. (2026)
Systemic connective-tissue disease	Systemic lupus erythematosus	Sporadic	Yes	Al-Kandari et al. (2014)
	Sjögren syndrome	Sporadic	Yes	Matta et al. (2018)
	Scleroderma	Sporadic	Case-level	Anwar et al. (2017)
	Behçet disease	Sporadic	Case-level	Kiryukhin et al. (2021)
	Psoriasis	Sporadic	Case-level	Shahrvini et al. (2026) and Matta et al. (2018)
	Achalasia	Sporadic	Case-level	Shahrvini et al. (2026)
Other autoimmune/autoinflammatory diseases	Autoimmune hemolytic anemia	Sporadic	Case-level	Isoldi et al. (2024)
	IgG4-related disease	Sporadic	Case-level	Tiftikçi et al. (2023)
	Yao syndrome	Sporadic	Case-level	Damianos et al. (2025)
	Autoimmune encephalitis	Sporadic (with CVID)	Case-level	Jacob et al. (2021)
Immunodeficiency	Common variable immunodeficiency (CVID)	Multiple cases	Yes	Mandaliya et al. (2016) and Jacob et al. (2021)
	Selective IgM deficiency	Sporadic	Yes	Narsai et al. (2021)
	IgA deficiency	Sporadic	Yes	Choung et al. (2022) and Anwar et al. (2017)
	Hypogammaglobulinemia	Sporadic	Yes	Aigner et al. (1997)
	HIV infection	Sporadic	Yes	Kiryukhin et al. (2021)
Genetic/HLA	HLA-DQ2/HLA-DQ8 carriage	53% in Käppi pediatric series (n = 15); DQ2.5 subtype in 33%, modestly above Scandinavian controls (20%–25%)	—	Käppi et al. (2020) and Leung et al. (2009)

The consistency and breadth of these associations across independent cohorts argue strongly against coincidence and instead suggest shared mechanisms—plausibly involving chronic mucosal lymphocytic activation, impaired immune regulation, and genetic susceptibility—linking CG to a broader autoimmune diathesis. Two cohort-level observations reinforce this framework. In the Käppi pediatric series, 60% of children showed evidence of autoimmune predisposition (autoimmune-related genetic factors in 47% or positive autoantibodies in 40%), and 53% carried HLA-DQ2 or HLA-DQ8 haplotypes (HLA-DQ2.5 most prevalent at 33%) (Käppi et al., 2020). Furthermore, the association with celiac disease—and with HLA-DQ2/DQ8 carriage and serological reactivity—has prompted exploration of gluten-related mechanisms.

#### 3.2.2 Local immune environment and inflammatory mechanisms

The immunological basis of CG is further substantiated by analyses of the local mucosal environment. The lamina-propria infiltrate contains diverse immune cells, whose sustained activity perpetuates the characteristic cycle of inflammation and fibrosis (Zhang et al., 2010), with documented infiltration of lymphocytes, plasma cells, and eosinophils, the presence of CD25^+^ cells, and an increased density of CD3^+^ intraepithelial T lymphocytes (Côté et al., 1998; Akkari et al., 2018). Recent gene-expression profiling has revealed concurrent upregulation of Th1- and Th2-associated cytokines within CG mucosa (Liu et al., 2024), suggesting that the disease departs from conventional polarized inflammatory paradigms. The simultaneous activation of Th1 and Th2 pathways may synergistically drive the dual pathological features of CG: sustained chronic inflammation and progressive fibrotic remodeling (Kajino et al., 2003; Pulimood et al., 1999).

The transition from chronic inflammation to subepithelial fibrosis is best understood within the canonical wound-healing axis in which transforming growth factor-beta 1 (TGF-β1) functions as a master profibrotic cytokine. Eosinophils—abundantly present in the CG lamina propria and observed actively degranulating within the trapillary matrix on ultrastructural examination (Li et al., 2025; Pulimood et al., 1999)—have been hypothesized to represent a major local source of TGF-β1, providing a putative mechanistic link between eosinophil-rich inflammation and fibroblast activation. Liu et al., (2024), interrogating 778 inflammation- and immunology-related transcripts in gastric biopsies from 10 patients with CG, demonstrated significant upregulation of *TGFB1* (2.13-fold; *p* = 0.040), matrix metalloproteinase-3 (*MMP3*; 6.70-fold; *p* = 0.029), and matrix metalloproteinase-9 (*MMP9*; 11.62-fold; *p* = 0.027), with no compensatory increase in the tissue inhibitor of metalloproteinase-1 (*TIMP1*). Activated TGF-β1 signaling may, together with relative TIMP1 deficiency, contribute to dysregulated extracellular matrix turnover skewed toward net collagen deposition rather than balanced remodeling. Although platelet-derived growth factor (PDGF) signaling has not been specifically interrogated in CG or, to our knowledge, in collagenous colitis, its established role in driving fibroblast-to-myofibroblast transition in fibrosing conditions (Aigner et al., 1997; Günther et al., 1999) suggests a plausible analogous contribution that warrants direct investigation.

Ultrastructural studies have identified activated subepithelial myofibroblasts as the principal cellular source of the abnormal collagen band (Li et al., 2025; Pulimood et al., 1999). By analogy with collagenous colitis, these cells exhibit a contractile phenotype with α-smooth muscle actin expression and the abnormal band variably contains type III collagen and tenascin-C—a signature consistent with an active wound-healing matrix rather than mature scar (Arnason et al., 2015; Kajino et al., 2003; Brain et al., 2009). The myofibroblast compartment in CG appears to operate within a sustained crosstalk loop involving (i) damaged surface epithelium releasing damage-associated signals and showing possible impaired EGF-mediated repair (Curci et al., 2022), (ii) infiltrating Th1/Th2 lymphocytes providing IFN-γ-, IL-4-, IL-5-, and IL-13-driven activation cues (Liu et al., 2024), (iii) eosinophils providing TGF-β1- and major basic protein-mediated profibrotic input, and (iv) entrapped capillaries within the band experiencing ischemic stress that further perpetuates the inflammatory milieu.

Single-cell transcriptomic interrogation of a mixed pediatric–adult CG cohort by Walsh et al. (2026) has refined this model considerably. Fibroblasts in CG mucosa expressed markedly higher levels of fibrillar-collagen genes—including a near 6-fold increase in *COL6A3*—and the lamina propria was enriched for highly activated, antigen-experienced CD4^+^ T cells with elevated *ITGA4* expression (encoding integrin α4), implicating the α4β7–MAdCAM-1 mucosal homing axis in the recruitment and retention of pathogenic lymphocytes. In the same study, profound depletion of parietal cells and an inverted gastric CD4:CD8 ratio were identified that was not present in matched duodenal mucosa, providing the first cell-resolution evidence that adult CG involves both selective glandular destruction and tissue-targeted recruitment of mucosa-homing CD4^+^ effector populations (Walsh et al., 2026).

The prominent eosinophilic component observed in many CG cases has prompted investigation of eosinophil-driven pathogenic pathways. Ultrastructural evidence of active eosinophil degranulation suggests a Th2-mediated or allergen-driven contribution in some patients. One prevailing hypothesis posits that eosinophilic degranulation directly contributes to fibrotic tissue remodeling, potentially initiated by an unidentified intraluminal antigen (Winslow et al., 2001); continued dietary exposure to such antigens could explain the characteristic refractoriness of CG to pharmacological therapy. However, investigations into specific eosinophil-related chemokines have yielded counter-intuitive results (Arnason et al., 2015). The eotaxin family—particularly eotaxin-3 (CCL26)—is the dominant chemoattractant axis driving mucosal eosinophilia in eosinophilic esophagitis and other Th2-skewed gastrointestinal disorders and, on theoretical grounds, therefore represents a logical candidate mediator linking the eosinophilic infiltrate of CG to fibrotic remodeling. The available immunohistochemical data, however, do not support this expectation: eotaxin has not been shown to be upregulated in gastric biopsies from patients with CG, in marked contrast to the robust upregulation of eotaxin-3 in the esophageal epithelium of patients with eosinophilic esophagitis. Two implications follow. First, eosinophil recruitment in CG appears to operate, at least in part, through eotaxin-3-independent pathways that remain to be elucidated. Second, the well-documented suppressive effect of PPIs on epithelial *CCL26*/eotaxin-3 transcription—an off-target mechanism that contributes to the histological response of PPI-responsive eosinophilic esophagitis—is unlikely to operate analogously in CG, because baseline eotaxin-3 expression is already low. The frequent symptomatic response to PPIs in CG is, therefore, more plausibly attributable to acid suppression and non-specific anti-inflammatory effects than to chemokine-targeted attenuation of eosinophil recruitment.

A single proteomic and immunoassay study by Curci et al. (2022)—the only molecular-level interrogation of CG tissue published so far—identified aberrant expression of 63 proteins, together with reduced serum EGF concentrations, in patients with CG compared with non-collagenous gastritis controls. *In silico* STRING pathway analysis from the same study placed EGF within networks linked to extracellular matrix collagen turnover, fibrillogenesis, vascularization, and epithelial remodeling. Because EGF physiologically both upregulates collagenase activity and suppresses collagen gene transcription, its loss has been hypothesized to tip the balance toward net subepithelial deposition. This framework, however, remains conjectural: it has not been replicated in independent CG cohorts, has not been demonstrated *in vivo*, and cannot yet distinguish a primary mechanistic role for EGF deficiency from its appearance as a secondary epiphenomenon of chronic inflammation. Until corroborated by independent multicenter studies incorporating direct manipulation of EGF signaling in relevant gastric models, EGF should be regarded as a candidate biomarker of interest, rather than an established pathogenic mediator.

By analogy with collagenous colitis, three pathogenic factors have been proposed for increased subepithelial collagen deposition: chronic inflammation and autoimmune injury, aberrant function of the pericryptal fibroblastic sheath, and leakage of plasma proteins and fibrinogen with subsequent collagen replacement (Hwang et al., 1986; Suda et al., 2023). Given the histological similarities and clinical associations between collagenous colitis and CG, these mechanisms may also apply to gastric involvement. According to this model, an initial insult—infectious, pharmacological, or allergic—damages the mucosal surface, triggering autoimmune responses and sustained inflammation in susceptible individuals; the cytokines released activate pericryptal myofibroblasts that migrate upward and drive subepithelial collagen deposition. The frequent coexistence of CG with lymphocytic gastritis, immune-mediated diseases, and celiac disease, together with the proposed therapeutic analogy to the gluten-free diet used in celiac disease and the involvement of Th2 immunity, broadly supports this immunological framework.

#### 3.2.3 Alternative and complementary hypotheses

Beyond the primary immunological model, several complementary hypotheses have been advanced. The injury–repair hypothesis posits that the collagen band reflects participation in post-injury fibrotic remodeling of the digestive tract and other organs (Chakir et al., 2003; Gillessen et al., 1993); positive tenascin staining within the subepithelial collagen layer in the two CG cases reported by Brain et al. (2009), supports this framework as tenascin marks mesenchymal cell proliferation and migration during embryonic development and wound healing (Günther et al., 1999; Jones and Jones, 2000). Hypersensitivity-driven mechanisms may also contribute, by analogy with the significantly increased histamine content and secretion observed in the colonic mucosa of patients with collagenous colitis (Schwab et al., 2002).

A specific eosinophilic-progression hypothesis has been informed by the report of Klein et al., who documented apparent progression from chronic inactive gastritis through eosinophilic gastritis to ultimately CG (Lucendo et al., 2011), and by the observation that the eosinophilic pattern is a common histological motif in CG, with eosinophils promoting profibrotic cytokine expression, increased collagen deposition, and tissue remodeling (Arnason et al., 2015; Chakir et al., 2003; Lucendo et al., 2011). This concept is further substantiated by a recent case series of eight adults with CG that revealed a consistent and previously underemphasized association with eosinophilic gastritis: all patients met diagnostic criteria for both conditions, and although budesonide, PPIs, and elimination diets produced symptomatic improvement, only a minority achieved histological improvement in collagen deposition (Shahrvini et al., 2026). The authors concluded that adult-onset CG and eosinophilic gastritis may exist on a spectrum, with chronic eosinophilic inflammation potentially progressing to collagen deposition (Shahrvini et al. (2026)). Other investigators have proposed primary vascular anomalies that increase vascular permeability—resulting in protein extravasation and collagen deposition—and fibrotic reactions secondary to primary inflammatory processes in susceptible subjects (Hijaz et al., 2013).

#### 3.2.4 Integrated pathophysiological model and pediatric–adult mechanistic divergence

Synthesizing these observations, a working pathophysiological model can be assembled, in which immune injury or hypersensitivity acts as a candidate etiological factor in susceptible individuals. Under such stimulation, the gastric mucosa develops inflammation and microscopic injury that recruits inflammatory cells, whose cytokines promote mesenchymal cell activation and extracellular matrix deposition. Excessive matrix deposition forms the abnormally thickened subepithelial collagen plate, effectively encapsulating the inflammatory sites. As the plate thickens, the burden on the lamina-propria vasculature increases, producing epithelial ischemia and hypoxia, along with a secondary wave of injury. Concurrently, the pro-angiogenic milieu of the lamina propria accelerates neovascularization in response to mucosal hypoxia; the resulting dilated superficial capillaries entrapped within the collagen layer have been proposed as a potential source of bleeding in some patients, although most patients lack clinical or occult evidence of gastrointestinal hemorrhage and alternative mechanisms such as impaired iron absorption may contribute; either way, these processes may participate in perpetuation of the inflammatory–fibrotic cycle. In patients with corpus-predominant disease, progressive collagen-induced and inflammation-driven collapse of the oxyntic glandular compartment provides an additional pathway by which atrophy and refractoriness can arise. By cautious analogy with telomere-biology-disorder-associated gastritis, in which depletion of CCK_2_R^+^/Ki67^+^ and TFF2^+^/Ki67^+^ progenitor cells within the gastric isthmus has been linked to chronic atrophic changes and persistent inflammation (Setia et al., 2024), it is conceivable—although not yet demonstrated in CG specifically—that gastric stem/progenitor cell dysfunction could contribute to the chronic atrophic features observed in some patients. This analogical hypothesis remains untested in CG and warrants direct investigation before any pathogenic role can be attributed to it.

The mechanistic divergence between pediatric- and adult-onset CG, although unresolved, can now be tentatively framed by convergent epidemiological, immunological, and single-cell observations. In pediatric-onset disease, gastric involvement is typically isolated, eosinophil-rich histology predominates (Arnason et al., 2015; Käppi et al., 2020), iron-deficiency anemia is the dominant clinical feature, and serological autoimmunity—when detected—is often subclinical (Käppi et al., 2020). The Th2 component of the mixed Th1/Th2 cytokine signature documented by Liu et al. (2024), with prominent IL-5 and IL-13 expression, is consistent with an environmental-allergen or food-antigen-driven hypersensitivity component, providing a plausible mechanistic substrate for the partial responsiveness of selected pediatric cases to elimination diets (Matta et al., 2018; Bajwa et al., 2015). By contrast, adult-onset CG more frequently forms part of a multi-segment collagenous gastroenteropathy, is associated with clinically established autoimmune comorbidities (Sjögren syndrome, Hashimoto thyroiditis, SLE, and autoimmune gastritis) (Genta et al., 2021), and may show a stronger atrophic histological pattern with parietal-cell loss and pseudopyloric metaplasia (Li et al., 2025). The upregulation of ITGA4 on activated CD4^+^ T cells in adult CG mucosa, together with corresponding upregulation of the endothelial ligand MAdCAM-1 (Walsh et al., 2026), suggests that the disease may be sustained, at least in part, by ongoing recruitment of mucosa-homing effector lymphocytes rather than by Th2-driven luminal hypersensitivity alone. Whether these two phenotypes represent distinct pathogenic entities sharing a final histological pathway, or sequential stages along a single immunological trajectory, remains an open question for which longitudinal cohort data are required.

### 3.3 Potential triggers: drugs and infectious agents

Beyond underlying immune predisposition, external triggers may initiate or unmask CG in susceptible individuals. Several drugs have been implicated in causing a spectrum of collagenous and lymphocytic gastrointestinal inflammation that can affect multiple sites, including the stomach (Varelas et al., 2022). The angiotensin II receptor blocker olmesartan—already known to cause a sprue-like enteropathy—has been associated with the development of CG (Higashimori et al., 2024), suggesting that certain pharmacological agents can trigger the histopathological changes characteristic of the disease. Additional case reports have documented CG attributed to serotonin–norepinephrine reuptake inhibitors (venlafaxine and duloxetine), expanding the list of potential pharmaceutical triggers (Varelas et al., 2022; Ankamah and Aaron, 2022).

Infectious agents have also been investigated. CG is generally considered a *Helicobacter pylori*-negative gastritis (Assa et al., 2022; Kiran et al., 2023), although co-occurrence with *H. pylori* infection has occasionally been reported without establishing a causal link (Jain and Chetty, 2010). Notably, *H. pylori* prevalence is comparable between pediatric (n = 6) (Kori et al., 2007; Dray et al., 2007; Billiémaz et al., 2009; Leiby et al., 2008) and adult (n = 4) (Al-Kandari et al., 2014; Jain and Chetty, 2010; Lagorce-Pages et al., 2001) CG patients, and *H. pylori* eradication has not produced reproducible therapeutic benefit (Narsai et al., 2021), arguing against a pathogenic role.

Most recently, a single case report has speculatively increased the possibility that reactivation of EBV—a ubiquitous γ-herpesvirus latent in the majority of adults—could be implicated in CG recurrence/relapse in selected patients (Duncan et al., 2024). In that single observation, the authors hypothesized, without supporting mechanistic data, that an antecedent infection such as SARS-CoV-2 might create immune conditions permissive to EBV reactivation, which could, in turn, precipitate the gastric inflammatory response. This proposal is based on n = 1, and no causal, temporal, dose–response, or epidemiological evidence currently links EBV reactivation to CG initiation or relapse. Of separate but related concern, a previously published case has documented progression from long-standing CG to EBV-positive gastric carcinoma over an 8-year interval (Narsai et al., 2021); given EBV’s established oncogenic role in a subset of gastric cancers, this observation has been read as suggestive of viral involvement but cannot, in the absence of larger datasets, considered more than an isolated coincidence. The EBV–CG association should, therefore, be regarded as anecdotal and hypothesis-generating only. Larger seroprevalence studies, EBV-encoded RNA *in situ* hybridization (EBER-ISH) on CG biopsies, and longitudinal observations linking acute EBV reactivation to incident CG are required before any mechanistic role can reasonably be entertained. Importantly, the recent single-cell transcriptomic interrogation of CG tissue by Walsh et al. (2026) did not assess viral contributions and did not report EBV-related transcripts or virally driven T-cell activation profiles in the CG mucosa they profiled; the absence of such an analysis does not exclude a permissive role for EBV in selected cases, but neither does it provide support for EBV as a generalized pathogenic mechanism.

## 4 Clinical presentation and epidemiology

CG is a rare gastric disorder with distinctive epidemiological features and age-dependent clinical manifestations. Reported prevalence is approximately 13 cases per 100,000 upper gastrointestinal endoscopic examinations, with no significant ethnic predisposition (Genta et al., 2021). Despite its rarity, CG demonstrates a characteristic bimodal age distribution that fundamentally shapes clinical presentation, creating two distinct phenotypes with markedly different symptomatological profiles. Although pediatric cases are predominantly characterized by anemia and abdominal pain, adult presentations frequently involve diarrhea and broader gastrointestinal involvement. The disease’s potential to cause severe complications further underscores the importance of timely recognition.

### 4.1 The dichotomy of clinical phenotypes

Lagorce-Pages et al. (2001), on the basis of 14 CG patients, delineated two clinicopathologic subsets of CG by age at onset and clinical presentation. CG exhibits a bimodal age distribution, with a first peak in patients younger than 20 years and a second peak in patients older than 60 years, the latter heavily female-predominant (Choi et al., 2022). The female-to-male incidence ratio is approximately 1.6–4:1 (Choi et al., 2022).

Across published cohorts, clinical presentation is heterogeneous in both type and intensity. In the pooled scoping review of Kozai et al. (2023), anemia (61.4%), abdominal discomfort (60.5%), diarrhea (25.3%), and nausea/vomiting (23.0%) were the dominant complaints (Illan Montero et al., 2023; Kozai et al., 2023). In the registry-based study of Genta et al., (2021), presentations included diarrhea (18%), anemia (12%), weight loss (11%), and vomiting (10%), with significant associations with celiac disease, duodenal intraepithelial lymphocytosis, and lymphocytic gastritis. Choung et al. (2022) reported gastrointestinal bleeding in 20% of patients (45% of whom were children), substantially exceeding earlier estimates of approximately 5%. Abdominal pain and chronic anemia are the predominant initial complaints, each manifesting in roughly half of symptomatic individuals; pain ranges from intermittent diurnal, meal-unrelated cramping to debilitating persistent episodes, and anemia ranges from incidental laboratory abnormalities to clinically evident fatigue, pallor, exercise intolerance, palpitations, with recurrence of anemia after discontinuation of iron supplementation.

Anemia in CG arises through several converging mechanisms. The principal contributor is ongoing low-grade gastrointestinal blood loss from dilated, fragile capillaries entrapped within the thickened, irregular subepithelial collagen layer of the gastric mucosa (Côté et al., 1998). This continuous blood loss progressively depletes iron stores, producing iron-deficiency anemia. Concurrent gastric hypochlorhydria in CG patients further reduces dietary iron bioavailability (Pinis et al., 2024), and these combined processes account for the characteristically recurrent anemia observed in this population.

**FIGURE 2:**
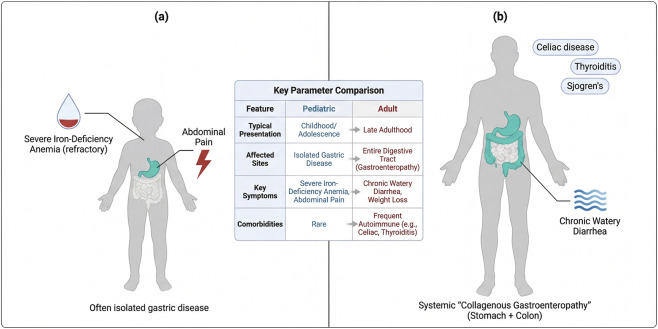
Distinct clinical phenotypes of collagenous gastritis. **(a)** Pediatric phenotype: typically presents in childhood or adolescence; disease is often isolated to the stomach. Key features include severe iron-deficiency anemia (frequently refractory to oral iron) and abdominal pain. **(b)** Adult phenotype: typically presents in late adulthood; often manifests as part of a systemic collagenous gastroenteropathy involving stomach and colon (collagenous colitis). Key features are chronic watery diarrhea and weight loss, with frequent autoimmune comorbidities (e.g., celiac disease and autoimmune thyroiditis).

The clinical manifestations of CG demonstrate strong age-dependent patterns, leading to the characterization of two distinct clinical phenotypes (Figure 2) (Suskind et al., 2009; Brain et al., 2009). This age-based distinction is one of the most consistent findings in the CG literature and has direct implications for diagnosis and management.

#### 4.1.1 Pediatric-onset phenotype

The pediatric-onset phenotype is the more commonly described form. CG presenting in children is mainly characterized by abdominal pain and severe anemia, with collagen deposition typically limited to the gastric mucosa. Other common features include recurrent epigastric pain, nausea, and vomiting (Mandaliya et al., 2013; Côté et al., 1998; Stancu et al., 2001; Leung et al., 2009; Lagorce-Pages et al., 2001; Rustagi et al., 2011; Zamani et al., 2018). Several case series have reinforced this classic presentation in this age group (Zheng et al., 2022; Côté et al., 1998; Park et al., 2005; Brain et al., 2009; Kori et al., 2007; Meunie et al., 2001; Ma et al., 2025). The anemia is frequently profound and characteristically refractory to oral iron supplementation, reflecting a substantial component of either malabsorption or ongoing occult blood loss from the inflamed gastric mucosa (Romano et al., 2023; Lee et al., 2019); in some cases, it is severe enough to require blood transfusion (Kamimura et al., 2015).

Mechanistically, gastrointestinal bleeding in CG may arise from a combination of factors: chronic bleeding from dilated capillaries entrapped in the collagen layer, underlying mucosal inflammation and atrophy, and a thickened, friable mucosa predisposed to barotrauma. Leung et al. (2022) reported a patient with CG in whom diffuse mucosal bleeding (barotrauma) occurred during gastroscopic insufflation, with serpiginous ulceration found on repeat endoscopy 3 days later, supporting that inflammation and atrophy may predispose the gastric mucosa to insufflation-related injury. Although iron supplementation can correct CG-related anemia—consistent with occult ongoing blood loss being the underlying mechanism—hypochlorhydria-related impairment of iron absorption may also contribute (Pinis et al., 2024).

Numerous case reports and series have underscored this presentation, highlighting CG as a critical, albeit rare, diagnosis to consider in any child with unexplained, persistent anemia and abdominal pain (Hayashi et al., 2018; Kozai et al., 2023; Beinvogl et al., 2021). The severity of anemia can be striking, with some pediatric cases requiring intensive management (Akkari et al., 2018; Blackmore and Leach, 2023; Çakar and Çakır, 2022; Lima Ferreira et al., 2025). In many pediatric cases, disease appears confined to the stomach, although co-occurring collagenous colitis has also been reported (Rosell-Camps et al., 2015; Matta et al., 2018). The chronic nature of these symptoms commonly produces a significant diagnostic delay during which children may undergo extensive evaluation before gastric biopsy reveals the underlying pathology (Wu et al., 2023). The clinical course in children is chronic, with variable responses to different treatment modalities (Hijaz et al., 2013).

#### 4.1.2 Adult-onset phenotype

In contrast, the adult-onset phenotype follows a distinct clinical trajectory. Adult CG is more commonly accompanied by collagenous colitis—and, less frequently, by collagenous involvement of other gastrointestinal sites—typically presenting with chronic watery diarrhea and weight loss, and shows a closer association with systemic immune-mediated disorders, particularly Sjögren syndrome, autoimmune thyroiditis, and concurrent lymphocytic colitis or ulcerative colitis (Vesoulis et al., 2000; Stancu et al., 2001; Brain et al., 2009; Anwar et al., 2017). These associations reinforce the view that adult CG often arises within a more generalized context of immune dysregulation.

The predominant symptom in adults is chronic watery diarrhea (Kagihara et al., 2023; Cai et al., 2023), a profile often indistinguishable from that of microscopic colitis; indeed, adult-onset CG is frequently associated with concurrent collagenous colitis (Camar et al., 2011; Ziaei et al., 2022). This strong association suggests that in adults, CG is often one component of a more widespread collagenous gastroenteropathy affecting both upper and lower gastrointestinal tracts (Genta and Rugge, 2024). Additional symptoms—abdominal pain, nausea, and dyspepsia—also occur in adults (Zamani et al., 2018; Jin et al., 2013). Anemia can also be present in adults (Zamani et al., 2018). Weight loss in adults may reflect more extensive intestinal involvement, often in the context of coexisting collagenous enteritis or celiac disease (Anwar et al., 2017). Of note, however, the comparative study by Matta et al. (2018) found CG in children and adults to be the same disorder with associated immune-mediated diseases, including pediatric patients with concurrent collagenosis at other gastrointestinal sites; whether pediatric and adult onset represent different stages of a single disease spectrum or biologically distinct entities therefore remains unresolved.

### 4.2 Severe and atypical manifestations

Although the typical presentations of CG are generally indolent, the disease can also produce severe, acute, and occasionally life-threatening complications. Chronic inflammation and mucosal friability can result in significant gastrointestinal bleeding; case reports have described patients presenting with hemorrhagic ulcers and even hemorrhagic shock requiring emergency intervention (Haase and Kelsen, 2012; Davis et al., 2025). One patient with collagenous gastroduodenitis developed a Dieulafoy-like ulcer—a rare cause of massive upper gastrointestinal hemorrhage (Soeda et al., 2014)—and another previously undiagnosed patient was identified after presenting with severe hemorrhagic gastric barotrauma (Leung et al., 2022). Recurrent gastric ulcers have been reported in collagenous gastroduodenitis (Koide et al., 2015).

Beyond bleeding, gastric perforation has been reported as a rare but catastrophic complication (Appelman et al., 2016), and generalized edema has been documented when chronic gastric inflammation is associated with substantial loss of serum proteins (Eke et al., 2019). Long-term sequelae of chronic inflammation in CG remain incompletely defined, although the report of progression to EBV-positive gastric cancer in a single patient raises concerns about a potential, albeit likely very rare, risk of malignant transformation (Narsai et al., 2021).

### 4.3 Association with pan-gastrointestinal disease

CG does not invariably occur as an isolated entity. It is increasingly recognized as part of a broader collagenous gastroenteropathy spectrum, of which collagenous gastroenteritis represents the multi-segment phenotype, characterized by subepithelial collagen deposition and chronic inflammation affecting multiple gastrointestinal segments simultaneously (Gopal and McKenna, 2010). Although isolated CG predominates in pediatric and younger patients, multi-segment involvement is more characteristic of adults.

The most well-established and frequently observed association is with collagenous colitis. The co-occurrence of CG and collagenous colitis is particularly common in adults, often presenting as a unified clinical syndrome dominated by chronic diarrhea (Kiryukhin et al., 2024; Camar et al., 2011). Although this association has also been documented in pediatric patients, isolated CG, nonetheless, predominates in this age group (Matta et al., 2018; Hangard et al., 2016).

Beyond gastric and colonic involvement, the disease spectrum can extend further. Collagenous involvement of the small bowel has also been described as part of the proximal collagenous gastroenteritides (O'Brien et al., 2011); collagenous duodenitis denotes comparable changes in the duodenum and frequently coexists with CG (35); and collagenous sprue represents a more extensive small-bowel disorder that presents with severe malabsorption and chronic diarrhea, with established associations with celiac disease (Vakiani et al., 2010). Pan-intestinal disease has even been documented in infants and adolescents, with concurrent CG, collagenous sprue, and collagenous colitis underscoring the potential for extensive involvement from early childhood (Billiémaz et al., 2009; Kang et al., 2021).

This broad disease spectrum suggests an underlying systemic pathogenic mechanism capable of manifesting at various sites along the gastrointestinal tract, with clinical phenotypes determined by the anatomical distribution and extent of collagen deposition. Existing evidence indicates that patients with isolated collagenous gastritis (with or without colonic involvement) generally experience outcomes equivalent to the background population, whereas those with collagenous sprue may have impaired prognoses, including a linkage to malignancy (especially T-cell lymphoma) and complications of malnutrition (Nielsen et al., 2014). Furthermore, celiac disease and celiac-like duodenal histology demonstrate a significant association with CG, co-occurring in approximately 10% of patients; however, these cases exhibit variable histopathological severity and inconsistent responses to gluten avoidance, suggesting that the relationship between CG and celiac disease may be more complex than simple comorbidity (Leung et al., 2009).

## 5 Diagnosis

The diagnosis of CG presents significant clinical challenges owing to its rarity, non-specific symptomatology, and variable endoscopic presentation. Diagnosis is, therefore, frequently delayed, and definitive confirmation rests primarily on histopathological examination of gastric biopsies.

### 5.1 The diagnostic approach: integrating clinical, endoscopic, and histological findings

**FIGURE 3:**
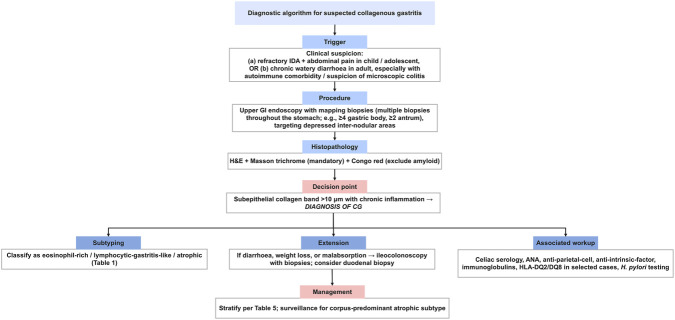
Diagnostic algorithm for suspected collagenous gastritis. Clinical entry triggers (refractory iron-deficiency anemia with abdominal pain in a child or adolescent; chronic watery diarrhea in an adult, particularly with autoimmune comorbidity or suspected microscopic colitis) prompt upper gastrointestinal endoscopy with mapping biopsies, including from the depressed inter-nodular zones where the collagen band is most concentrated. Histological confirmation requires a subepithelial collagen band exceeding 10 μm on Masson trichrome staining (Congo red-negative to exclude amyloid), accompanied by chronic lamina-propria inflammation. Once confirmed, histological subtyping and assessment of multi-segment involvement (ileocolonoscopy with biopsies if diarrhea or weight loss; duodenal biopsy if features of malabsorption) guide subsequent management. Targeted associated workup includes celiac serology, autoimmune antibodies, immunoglobulin levels, HLA-DQ2/DQ8 typing in selected cases, and Helicobacter pylori testing.

The path to a CG diagnosis begins with a high index of clinical suspicion—particularly in a child with refractory iron-deficiency anemia and abdominal pain, or in an adult with chronic watery diarrhea, especially when collagenous colitis is also present (Kozai et al., 2023; Wu et al., 2023). A pragmatic diagnostic algorithm is presented in Figure 3.

#### 5.1.1 Endoscopic evaluation

**FIGURE 4:**
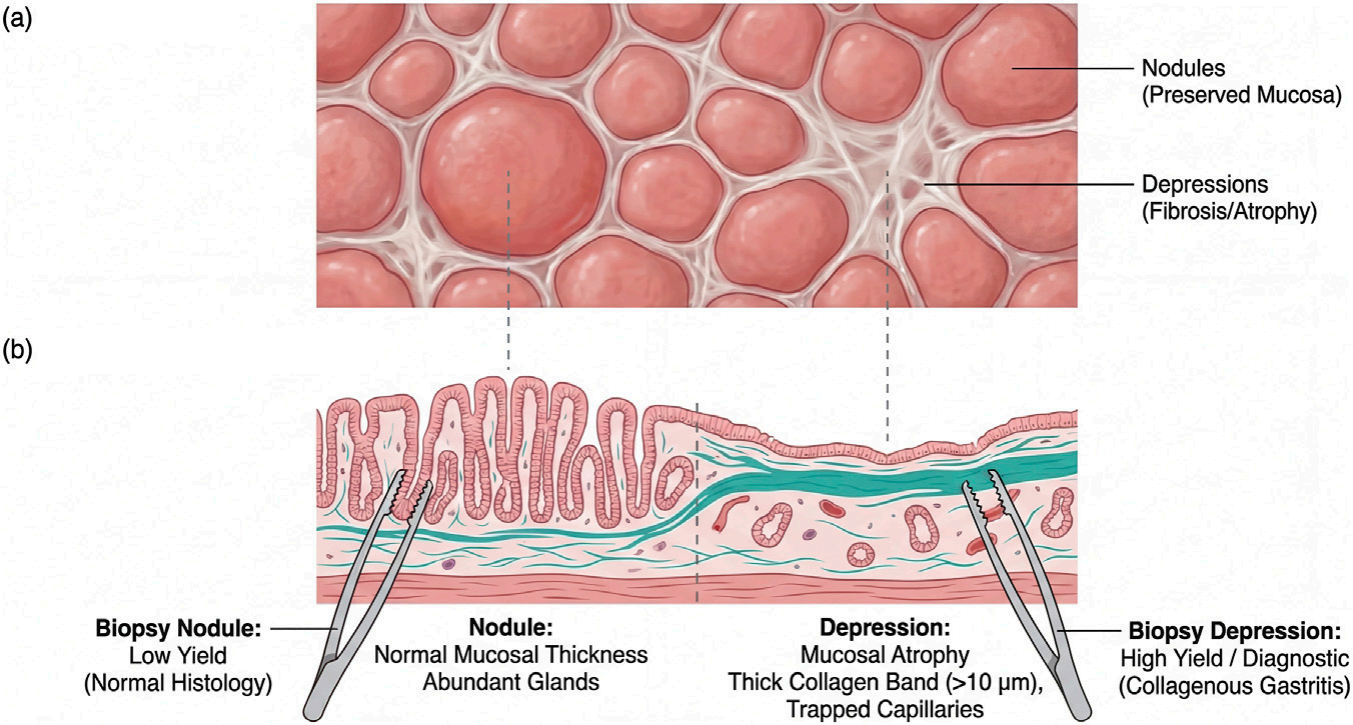
Correlation between endoscopic morphology and histopathology. **(a)** Endoscopic view: The characteristic “nodular” or “cobblestone” appearance of the gastric mucosa. **(b)** Histological cross-section: Schematic showing that the visible “nodules” correspond to islands of preserved mucosa, while the “depressed” perinodular areas contain the thickest collagen deposition and glandular atrophy. **(c)** Diagnostic implication: Biopsies from depressed inter-nodular areas yield higher diagnostic sensitivity, whereas nodular tissue may appear histologically near-normal.

Upper gastrointestinal endoscopy is the critical diagnostic gateway for suspected CG, but findings range from completely normal mucosa to highly characteristic nodular and atrophic patterns and can sometimes be misleading. The principal findings reported across the published literature are summarized in Table 3. The most consistent abnormality—present in roughly 53%–80% of patients across cohorts, with similar frequency in children and adults—is a diffuse or patchy mucosal nodularity variably described as “cobblestone,” “mosaic,” or “gooseneck” in pattern (Liao et al., 2019; Wilson et al., 2009). The nodules represent residual mucosa with relatively preserved glandular architecture and a low density of inflammatory infiltrate; their apparent height arises predominantly from depression of the surrounding inter-nodular mucosa, where atrophy, glandular collapse, and the rigid, downward-tethering collagen band concentrate the disease process (Winslow et al., 2001; Kamimura et al., 2007). This pathophysiological correlation has direct biopsy implications: the depressed inter-nodular zones—not the nodules themselves—yield the highest diagnostic sensitivity, and any biopsy strategy that samples only the visibly nodular tissue risks a false-negative result (Cortez et al., 2020) (Figure 4).

**TABLE 3:** Endoscopic findings in collagenous gastritis: prevalence and clinical correlates.

Endoscopic finding	Reported prevalence	Notes/clinical correlates	Key references
Mucosal nodularity (“cobblestone” and “mosaic”)	∼53%–80%; 77.5% in the Beinvogl pediatric cohort; 53.3%–77.5% across cohorts, with similar frequency in pediatric and adult patients	Most characteristic finding; nodules represent residual mucosa, with collagen band concentrated in depressed inter-nodular areas	Kamimura et al. (2015), Beinvogl et al. (2021), Choi et al. (2022), and Kang et al. (2021)
Mucosal erythema	Not systematically recorded (excluded as too subjective in the largest pediatric cohort)	Reported qualitatively in case reports (e.g., focal hyperemia; mucosal erythema with erosions); often diffuse	Kiryukhin et al. (2024) and Kang et al. (2021)
Erosions/superficial ulceration	∼10%	Reported in pediatric cohort; potential source of gastrointestinal blood loss	Beinvogl et al. (2021) and Kang et al. (2021)
Frank ulcer	∼7.5% in the Beinvogl cohort	Dieulafoy-like presentations and perforation reported only in isolated case reports, not in this cohort	Beinvogl et al. (2021), Soeda et al. (2014), and Appelman et al. (2016)
Visible blood/bleeding	∼20%	Reported in the pediatric cohort; consistent with gastrointestinal blood loss as a source of iron-deficiency anemia	Beinvogl et al. (2021)
Atrophic mucosa	∼4.5–5.7%	Corpus-predominant atrophic histological subtype reported; periodic endoscopic surveillance suggested in some reports	Winslow et al. (2001) and Appelman et al. (2016)
Pangastric “tubular” stomach	Rare, single reported case	Likely associated with subepithelial collagen deposition	Cortez et al. (2020)
Normal-appearing mucosa	∼12.5%	Underscores the necessity of biopsy when CG is clinically suspected	Kozai et al. (2023)

Beyond white-light findings, magnifying narrow-band imaging (M-NBI) demonstrates that nodule surfaces retain a regular foveolar (tubular) pattern with preserved, occasionally widened subepithelial capillaries, while inter-nodular depressed mucosa shows an obscure or absent surface architecture together with irregular, narrowed, tortuous capillaries—features mechanistically consistent with the underlying glandular atrophy and collagen entrapment (Zheng et al., 2022; Kamimura et al., 2007; Kawasaki et al., 2018). Endoscopic ultrasound (EUS) shows variable, irregular thickening of the second hypoechoic mucosal layer with the third (submucosal) layer usually preserved but does not directly visualize the subepithelial collagen band. Endocytoscopy has been described in single reports (Wang et al., 2025) but remains investigational. Endoscopic appearances differ between age groups: children and adolescents typically show striking nodularity, prominent rugal folds, and frank pseudopolypoid changes in body and corpus, whereas adults more commonly show mucosal erythema, atrophy, and only patchy or absent nodularity, with a “tubular” stomach reported in rare cases (Appelman et al., 2016; Cortez et al., 2020; Ravikumara et al., 2007). Even with usual air insufflation, fragile mucosa can rarely produce barotrauma-related hemorrhage (Leung et al., 2022). Because endoscopic appearance overlaps with H. pylori-related nodular gastritis, definitive diagnosis depends on histopathology, with multi-site biopsies including the depressed inter-nodular zones (Tanabe et al., 2013).

#### 5.1.2 Histopathology: the gold standard

Histopathological examination of gastric biopsy specimens remains the definitive method for diagnosing CG. The hallmark diagnostic features—a thickened subepithelial collagen band exceeding 10 μm and a chronic inflammatory infiltrate within the lamina propria—both confirm the diagnosis and differentiate CG from other inflammatory gastric disorders (Kamimura et al., 2015). The diagnostic process must, however, take account of age-related variation in disease distribution. In pediatric patients, collagen deposition typically exhibits a patchy distribution and predominant localization within the gastric body (Arnason et al., 2015); this heterogeneity has direct implications for the biopsy strategy and necessitates a systematic, multi-site approach to avoid false-negative results. In a pooled scoping review, subepithelial collagen banding was identified in 65.9% of cases and mucosal inflammatory infiltration in 37.5% of patients (Kozai et al., 2023), reflecting the variable presentation of CG and the importance of comprehensive histopathological evaluation.

### 5.2 Laboratory and other investigations

Laboratory tests are essential for assessing the systemic impact of disease. A complete blood count typically reveals microcytic anemia consistent with iron deficiency, particularly in children, and iron studies (serum iron, ferritin, and transferrin saturation) confirm the iron-deficient state (Agrawal et al., 2024). More recently, gastric-specific biomarkers—including serum pepsinogen I, pepsinogen II, the pepsinogen I/II ratio, and gastrin-17—have been proposed as the candidate non-invasive indicators of disease progression and treatment response (Zheng et al., 2022). These offer the advantage of reflecting gastric mucosal status without invasive procedures, although their utility in CG remains preliminary and they are not yet incorporated into routine practice.

### 5.3 Differential diagnosis

The differential diagnosis for CG is broad and depends on clinical presentation. In children presenting with anemia and abdominal pain, the principal alternatives are celiac disease, eosinophilic gastrointestinal disorders, *H. pylori* gastritis, and inflammatory bowel disease (Klein et al., 2021); the clinical and histological overlap with celiac disease can be particularly challenging, and selected cases of CG have closely mimicked it (Evans et al., 2023). In adults with chronic diarrhea, the dominant differential is with other causes of microscopic colitis, malabsorption, and atrophic gastritis. The principal entities to consider, along with the features that allow them to be distinguished histopathologically, are summarized in Table 4. Across all age groups, while endoscopic features such as mucosal nodularity or erythema may suggest CG, none is specific or consistently present; definitive diagnosis depends on histopathological identification of the characteristic thickened subepithelial collagen band on multi-site biopsies.

**TABLE 4:** Differential diagnosis of collagenous gastritis. Core discriminating features are emphasized; entities may co-occur.

Entity	Typical clinical features	Typical endoscopic features	Discriminating histological features	Key references
Collagenous gastritis	Refractory iron-deficiency anemia and abdominal pain (pediatric); chronic watery diarrhea (adult)	Nodular (“cobblestone”), erythematous, and sometimes atrophic mucosa	Subepithelial collagen band >10 μm with entrapped capillaries (“trapillaries”); chronic lymphoplasmacytic ± eosinophilic infiltrate; *H. pylori* usually absent	Kamimura et al. (2015) and Arnason et al. (2015)
*H. pylori* gastritis	Dyspepsia, peptic ulcer disease, and occasional anemia	Antral nodularity (especially in children) and erythema	Active chronic gastritis with *H. pylori* organisms; no diagnostic subepithelial collagen band	Kiran et al. (2023)
Eosinophilic gastritis	Abdominal pain and vomiting; frequent atopic background	Nodularity and erythema	Dense eosinophilic infiltrate (>20–30/HPF) of mucosa/submucosa; no diagnostic subepithelial collagen band; eosinophil-rich CG can overlap and may exist on a spectrum	Shahrvini et al. (2026) and Raffaele et al. (2021)
Autoimmune (atrophic) gastritis	Pernicious anemia and neurological B12 deficiency	Corpus-predominant atrophy	Corpus-predominant glandular atrophy with intestinal metaplasia; positive parietal-cell/intrinsic-factor antibodies; no diagnostic subepithelial collagen band	Zhang and Tang (2025)
Celiac disease	Diarrhea, malabsorption, and iron deficiency	Often normal stomach; duodenal scalloping and atrophy	Duodenal villous atrophy with intraepithelial lymphocytosis; positive TTG/EMA serology; CG may co-occur	Evans et al. (2023) and Freeman (2009)
Lymphocytic gastritis	Often subclinical or dyspeptic	Variable; may show varioliform gastritis	≥25 IELs per 100 epithelial cells; may overlap with the lymphocytic-gastritis-like CG subtype but lacks the diagnostic subepithelial collagen band when isolated	Stancu et al. (2001)
Amyloidosis (gastric)	Systemic disease-dependent	Variable	Subepithelial hyaline deposit; Congo red-positive with apple-green birefringence (negative in CG)	—

## 6 Natural history, long-term sequelae, and management

### 6.1 Natural history, prognosis, and long-term sequelae

The natural history of CG is incompletely defined, owing to the limited availability of long-term follow-up data from sizeable cohorts. The disease is, however, generally chronic and persistent. In children, histological lesions have been reported to remain unchanged over several years, despite various medical therapies (PPIs, sucralfate, and corticosteroids) (Côté et al., 1998), although clinical symptoms—particularly anemia—are often manageable with iron supplementation and supportive care.

Several case-level observations illustrate the heterogeneity of long-term trajectories. Kamimura et al. (2007) followed a CG patient for 14 years and observed that the nodular endoscopic appearance became more pronounced and extended throughout the entire stomach, with histology showing increased collagen deposition. In contrast, Winslow et al. (2001), during 12 years of follow-up of a CG patient, found that the nodular appearance remained unchanged on endoscopy and that biopsies showed patchy chronic active gastritis without progressive thickening of the collagen band, although histologic disease severity gradually worsened—suggesting that collagen-band thickness does not necessarily parallel disease severity. Improvement has also been documented: Lagorce-Pages et al. (2001) described two CG patients in whom biopsies obtained 2 and 8 years after initial diagnosis revealed complete disappearance (in a 40-year-old woman) or moderate reduction (in an 11-year-old boy) of the subepithelial collagen deposit; Hijaz et al. (2013) reported inflammatory improvement and disappearance of the collagen deposit in a CG patient followed for 5 years on oral iron and PPI therapy; and Leung et al. (2009) documented disappearance of the collagen deposit in a 19-year-old male CG patient at a biopsy 3 months after diagnosis (treated with sucralfate), although lymphocytic gastritis newly emerged at that time. In most reported cases, however, the collagen deposit remains unchanged or thickens further. Pulimood et al. (1999) reported a young adult male, initially with isolated collagenous gastritis, who developed collagenous colitis approximately 6 years later, suggesting that subepithelial collagen deposition may involve the gastrointestinal tract more diffusely over time.

Long-term sequelae are an emerging area of concern, and the available evidence indicates that this concern applies disproportionately to the corpus-predominant atrophic subtype. The most informative single observation remains the 12-year longitudinal follow-up by Winslow et al. (2001), in which corpus-predominant atrophic CG progressed to glandular atrophy, intestinal metaplasia, endocrine cell hyperplasia, reduced antral gastrin-cell numbers, and reactive epithelial changes indeterminate for dysplasia. This pattern of endocrine-cell dysregulation is mechanistically analogous to the milieu that predisposes patients with autoimmune corpus-predominant atrophic gastritis to type 1 gastric neuroendocrine (carcinoid) tumors, providing the principal biological rationale for surveillance (Ravikumara et al., 2007). Convergent supporting evidence comes from the report of Narsai et al. (2021), in which long-standing CG with selective IgM deficiency progressed to EBV-positive gastric adenocarcinoma over an 8-year interval (, and from cohort observations in which the atrophic CG subtype has been associated with treatment refractoriness and rare progression to dysplasia or neuroendocrine hyperplasia (Winslow et al., 2001; Anderson and Ravi, 2019; Patel et al., 2023).

Although none of these reports permits formal quantification of malignancy risk—and the absolute risk in CG is almost certainly low—the convergence of histological, mechanistic, and case-level signals justifies preferential endoscopic surveillance for patients with corpus-predominant atrophic CG. Routine surveillance of non-atrophic, antrum-only, or short-duration disease cannot be supported on present evidence. These observations should also prompt clinicians to reconsider any previously assumed entirely benign prognosis of CG and to incorporate explicit long-term surveillance planning into management of high-risk subtypes.

### 6.2 Therapeutic strategies: an individualized approach

#### 6.2.1 Treatment challenges and goals

The management of CG remains one of the most challenging aspects of this rare condition. No universally accepted treatment algorithm exists, and therapeutic strategies are derived primarily from case reports and small case series, rather than from controlled trials. This limitation reflects both the rarity of the disease and the existence of two clinically distinct subgroups, which together have precluded randomized clinical trials. The fundamental challenge is finding therapy that not only improves symptoms but also addresses the underlying collagenous proliferation that results from chronic gastric inflammation (Brain et al., 2009). Treatment goals are threefold: symptomatic relief, correction of iron-deficiency anemia, and induction of histological remission. In practice, however, many interventions provide significant symptomatic benefit but fail to achieve sustained histological improvement.

#### 6.2.2 Evidence-graded stepwise treatment framework

Given the limited evidence base, therapeutic approaches to CG have evolved through empirical testing of pharmacological and dietary interventions. The most comprehensive aggregate data come from retrospective analyses describing real-world treatment patterns and outcomes. In the pooled scoping review of Kozai et al. (2023), the most commonly employed treatments were iron supplementation (42% of patients), followed by PPIs (30.7%), prednisone (9.1%), and budesonide (6.8%), with an overall clinical improvement rate of 64.2%. Current treatment paradigms typically use combination therapy rather than single-agent approaches, with PPIs and oral iron supplementation forming the foundation, augmented by corticosteroids (particularly budesonide) or histamine-2 receptor antagonists (H_2_RAs) according to disease severity and patient-specific factors (Kozai et al., 2023). The chronic and sometimes relapsing nature of the disease necessitates long-term follow-up to monitor symptoms, nutritional status, and potential complications (Hijaz et al., 2013). Evidence-graded options, dosing, and reported response rates are summarized in Table 5.

**TABLE 5:** Evidence-graded stepwise treatment options for collagenous gastritis. Doses reflect those reported in the published literature; pediatric doses are weight-adjusted, and most are off-label for CG. Reported response rates derive from retrospective case series and small cohorts; no randomized trial has been conducted in CG.

Tier	Intervention	Adult dose	Pediatric dose	Reported clinical response	Reported histological response	Evidence base	Key references
First line—symptomatic/supportive	Oral iron (ferrous asparto glycinate plus polysaccharide-iron complex, or ferrous sulfate)	Not reported	0.7–2.5 mg/kg/day	Anemia correction in all treated patients; recurrence of iron deficiency reported in ∼93% (13/14) of pediatric patients in the Käppi cohort	Not applicable	Multiple case series; retrospective cohorts	Beinvogl et al. (2021) and Käppi et al. (2020)
	Intravenous ferric carboxymaltose	-	15 mg/kg per infusion (off-label in young children)	Median hemoglobin increase of 2.9 g/dL (range 1.6–3.3) per infusion in the Isoldi pediatric series (n = 3); no red blood cell transfusion required	Not applicable	Small pediatric case series	Isoldi et al. (2024)
	Proton pump inhibitor (omeprazole, pantoprazole, and esomeprazole)	No pooled dosing recommendation (esomeprazole 40 mg/day used in one De Ronde pediatric case)	No pediatric dosing recommendation	81% PPI response in the pooled scoping review (mixed adult + pediatric); 76.2% PPI clinical response (78.6% with oral iron) in the De Ronde pediatric cohort	9.5% histological improvement with PPI (overall PPI group; 14.3% in PPI + iron subgroup) in the De Ronde pediatric cohort	Pooled retrospective data (Kozai: mixed-age scoping review; De Ronde: pediatric cohort)	Kozai et al. (2023) and De Ronde et al. (2020)
Second line—anti-inflammatory	Topically targeted (open-capsule or compounded) budesonide	Open-capsule budesonide 3 mg three times daily (9 mg/day); maintenance either low-dose or full-dose (specific milligram amounts not reported)	Not reported as a specific regimen; paper only cautions that TTB be used cautiously with regular adrenal-axis monitoring	Complete or partial response in 25/28 (89%) overall; among the 24 responders, 75% became budesonide-dependent	Complete or partial response in 13/15 (87%) of patients with follow-up biopsies	Largest open-label retrospective series	Choung et al. (2022)
	Corticosteroids	Prednisone 40 mg/day, tapered over ∼2.5 months (Lim, 2018); budesonide 9 mg/day tapered to 3 mg/day (Macaigne, 2010)	Not reported in these cases	Clinical response in selected cases; complete clinical resolution reported in an 85-year-old patient	Variable: histology normalized in one elderly case (Lim, 2018); collagen band persisted despite clinical improvement in another (Macaigne, 2010)	Case-level reports	Macaigne et al. (2010), Hissong et al. (2025), and Lim et al. (2018)
Third line—immunomodulator/biologic	Azathioprine	Not reported	Induction: 12.5 mg/day (with prednisone 20 mg); maintenance: 6.25 mg/day (with prednisone 2.5 mg)	Reported in a steroid-dependent case; sustained remission >8 months	Not reported	Case-level reports	Eke et al. (2019)
	Vedolizumab	300 mg IV at weeks 0, 2, 6, and then every 8 weeks. Off-label; dosing extrapolated from IBD use	Not reported	Sustained clinical response with histological epithelial restitution in a young adult; collagen band largely unchanged	Histological epithelial repair; collagen deposition largely unchanged on follow-up biopsy in the reported case	Single case report; mechanistic rationale strengthened by single-cell transcriptomic data	Patel et al. (2023) and Walsh et al. (2026)
Dietary	Gluten-free diet	Not reported	May be trialed in CG, including without celiac disease	Symptomatic improvement reported in selected cases; histological resolution rare	Histology typically unchanged	Case-level reports	Matta et al. (2018) and Bajwa et al. (2015)
	Empiric multi-food elimination (elemental) diet	-	Simultaneous elimination of multiple foods (cow-milk protein, egg, soy, legumes, gluten, fish/shellfish, and nuts), followed by gradual reintroduction	Sustained clinical remission described in a pediatric atrophic-pattern case refractory to corticosteroids	Endoscopic/histological abnormality persisted despite clinical remission	Case-level reports	Varela Rey et al. (2019)
Trigger removal	Discontinuation of olmesartan/other implicated agents (e.g., SNRIs)	When implicated	Not applicable	Symptom resolution within 2 weeks; weight recovery within 2 months in the Higashimori case (no follow-up data for the SNRI case)	Complete histological resolution at 6 months in the same case (follow-up biopsy not performed in the SNRI case)	Case-level and drug-induced subset	Varelas et al. (2022) and Higashimori et al. (2024)
Adjunct (when indicated)	*H. pylori* eradication (standard regimen when co-infection confirmed)	Per standard *H. pylori* guidelines (not specified in CG literature)	Per age-appropriate *H. pylori* guidelines (not specified in CG literature)	Variable; symptomatic improvement reported in some co-infected patients after eradication	Variable; partial or complete resolution of endoscopic findings and the subepithelial collagen band reported in some cases, no improvement in others	Case-level	Narsai et al. (2021) and Kiran et al. (2023)
Surveillance (atrophic pattern)	Endoscopy with mapping biopsies	Reasonable in corpus-predominant atrophic CG, especially with autoimmune comorbidity	Not currently recommended on present evidence	Surveillance interval of 2–3 years suggested by analogy with autoimmune corpus-predominant atrophic gastritis	—	Expert opinion based on longitudinal case observations	Winslow et al. (2001) and Narsai et al. (2021)

#### 6.2.3 Symptomatic and supportive management

Management of CG is characterized by a frequent dissociation between symptomatic improvement and histological response. For most patients, symptom control and correction of nutritional deficiencies represent the most achievable and clinically meaningful endpoints.

PPIs form the cornerstone of symptomatic management and are typically used as first-line therapy. Beyond gastric acid suppression, PPIs may confer modest non-specific anti-inflammatory effects and promote healing of mucosal erosions, although they do not alter the underlying subepithelial collagen band. The clinical response approaches 80%–86% in pooled and pediatric cohorts, whereas histological improvement is documented in fewer than a quarter of follow-up biopsies (Kozai et al., 2023; De Ronde et al., 2020; Choung et al., 2022). Symptom recurrence after treatment discontinuation is frequently reported, underscoring the chronic relapsing nature of disease.

Correction of iron-deficiency anemia is a critical but challenging component of pediatric CG management. Although oral iron supplementation initially normalizes hemoglobin and iron parameters, iron deficiency frequently recurs, possibly because of decreased iron absorption due to gastric hypochlorhydria; in the Käppi pediatric cohort, iron deficiency with or without anemia recurred in 13 of 14 patients within a median of 1 year after initial treatment (Käppi et al., 2020). A separate, theoretical concern is that oral iron preparations themselves can induce or exacerbate gastric mucosal injury, a phenomenon best characterized in iron-pill gastritis, in which surface deposition of ferrous salts produces erosions and reactive epithelial change (Meliţ et al., 2017; Ji and Yardley, 2004). Direct CG-specific evidence for this effect is lacking, and the concern is, therefore, extrapolated from the general iron-pill gastritis literature rather than demonstrated in CG cohorts. Nonetheless, the fragility of the already collagen-encased gastric mucosa in CG provides a reasonable basis for a low threshold for switching to parenteral iron in patients who develop new or worsening dyspepsia during oral therapy. Intravenous ferric carboxymaltose has emerged as an effective alternative; in the Isoldi pediatric series, a single infusion produced a median hemoglobin increase of 2.9 g/dL (range 1.6–3.3 g/dL), and in the one patient with severe anemia, it was used *in lieu* of red-cell transfusion (Isoldi et al., 2024). In severe cases with multi-segment involvement and substantial nutritional compromise, total parenteral nutrition has been used successfully, particularly in patients with concurrent collagenous sprue and colitis (Billiémaz et al., 2009).

#### 6.2.4 Anti-inflammatory and immunomodulatory therapies

When symptomatic measures prove insufficient, therapies targeting the inflammatory process are required. Corticosteroids are the most commonly used agents for inducing remission and have demonstrated effectiveness in both adult and pediatric populations, with both systemic prednisone and topically targeted budesonide employed successfully. Budesonide, in particular, has shown promise, leading to both clinical and histological improvement—especially in patients with concurrent collagenous colitis (Macaigne et al., 2010; Choung et al., 2022; Hangard et al., 2016). In the largest retrospective open-label uncontrolled study of budesonide in CG, 89% of treated patients achieved a complete or partial clinical response, and 88% achieved a complete or partial histological response (Choung et al., 2022; Zhang et al., 2010; Lim et al., 2018); however, even when anemia and upper abdominal pain improve, endoscopic and pathological findings may show a more variable response, with collagen deposits often persisting (Choung et al., 2022; Lim et al., 2018).

Beyond corticosteroids, other anti-inflammatory agents have been explored. Mesalamine (5-aminosalicylic acid), commonly used in inflammatory bowel disease, has been reported as effective in selected CG cases (Mishra et al., 2022); additional agents employed in case-level practice include sucralfate (Ma et al., 2015; Côté et al., 1998; Winslow et al., 2001), bismuth compounds (Leiby et al., 2008), and azathioprine (Eke et al., 2019), although the supporting evidence for each remains limited. For refractory cases, more potent immunomodulators may be considered. Vedolizumab, a gut-selective integrin antagonist used for inflammatory bowel disease, has been successfully used in patients with CG (Patel et al., 2023), suggesting that targeting α4β7-mediated lymphocyte trafficking to the gut—mechanistically supported by the single-cell transcriptomic findings of Walsh et al. (2026)—may represent a viable therapeutic strategy for severe or treatment-resistant disease.

#### 6.2.5 Dietary interventions

Given the strong association between CG and celiac disease, as well as celiac-like presentations, dietary modification has emerged as a potentially important therapeutic approach in selected patients. A gluten-free diet has been reported to result in clinical and, occasionally, histological remission in some pediatric cases of CG, suggesting that gluten may serve as a trigger in a subset of patients (Stancu et al., 2001; Bajwa et al., 2015; Freeman, 2004). Matta et al. (2018) and Bajwa et al. (2015) described pediatric patients with CG who experienced clinical benefit on a gluten-free diet; however, in neither report, patients showed endoscopic or histological resolution of the characteristic collagenous gastric changes during follow-up, indicating that clinical and histological responses do not necessarily correlate.

A gluten-free diet should not, however, be considered the default dietary intervention in pediatric CG. It is most appropriate in patients with serological or histological evidence of celiac disease, or with HLA-DQ2/DQ8 carriage (Käppi et al., 2020; Matta et al., 2018). Beyond gluten avoidance, broader food-elimination protocols and amino-acid–based elemental formula deserve specific consideration, particularly in patients with Th2-leaning histological features and in those with atrophic-pattern disease unresponsive to corticosteroids. Varela Rey et al. (2019) described a pediatric patient with CG, longstanding celiac disease, and atrophic gastritis with achlorhydria who failed empirical *H. pylori* eradication and oral prednisone but achieved sustained clinical remission on a hypoallergenic regimen excluding cow-milk protein, egg, soy, legumes, gluten, fish, seafood, and nuts, allowing graded food reintroduction over 6 months while maintaining clinical control. This response is mechanistically consistent with the Th2 cytokine activity documented in CG mucosa (Liu et al., 2024) and with a rationale for antigen-driven exacerbation in a subset of patients. In practice, a graded approach—beginning with single-allergen elimination (most commonly cow-milk protein) and escalating to amino-acid-based formula in refractory cases—is reasonable in young children with severe disease, in whom prolonged systemic corticosteroid exposure carries unfavorable risk–benefit considerations.

The current evidence base for dietary intervention in CG remains very limited (Ma et al., 2015; Käppi et al., 2020; Matta et al., 2018; Bajwa et al., 2015), and the heterogeneous case-level data preclude definitive recommendations regarding which patients are most likely to benefit. Further research is needed to identify responsive subgroups, define optimal duration of dietary restriction, and develop standardized outcome measures.

#### 6.2.6 Role of *Helicobacter pylori* eradication

Although infectious agents have been postulated to trigger the inflammatory cascade underlying CG, the available evidence does not support a pathogenic role for *H. pylori* specifically. *H. pylori* prevalence is comparable between pediatric and adult CG cohorts and broadly resembles background population prevalence, arguing against a disease-specific causal association. Critically, in the small number of co-infected patients in whom successful eradication has been documented, the effect on CG has been variable: while some reports describe partial or complete resolution of the characteristic endoscopic findings and the subepithelial collagen band after treatment, others show no reproducible improvement, and eradication has not been consistently shown to alter the natural history of CG (Kori et al., 2007; Dray et al., 2007; Billiémaz et al., 2009; Leiby et al., 2008). Earlier suggestions that eradication might confer symptomatic benefit are best understood as resolution of concurrent *H. pylori*-attributable dyspepsia rather than as a CG-directed effect.

A pragmatic clinical position therefore emerges. *H. pylori* status should be assessed in patients with CG in line with general gastritis recommendations, because untreated infection carries its own well-established risks (peptic ulcer disease, gastric MALT lymphoma, and gastric adenocarcinoma) that are independent of CG. Where infection is confirmed, eradication is appropriate for those background indications. However, eradication should not be presented to patients as a CG-directed therapy, should not be expected to improve the collagenous histology or refractory anemia, and should not displace symptomatic, anti-inflammatory, and supportive measures.

#### 6.2.7 Surveillance and long-term monitoring

Although the absolute risk of malignant transformation in CG is almost certainly low and prospective surveillance data are not available, the convergent observations summarized in Section 6.1 provide a biological rationale for endoscopic surveillance in selected patients. The 12-year longitudinal follow-up of Winslow et al. (2001), in which corpus-predominant atrophic CG progressed to ECL-cell hyperplasia, intestinal metaplasia, and reactive epithelial changes indeterminate for dysplasia, and the documented progression to EBV-positive gastric adenocarcinoma over an 8-year interval reported by Narsai et al. (2021), together support preferential surveillance of the corpus-predominant atrophic subtype rather than indiscriminate surveillance of all CG patients.

In the absence of evidence-based intervals, it is reasonable to perform repeat upper endoscopy with mapping biopsies (multiple sites in body and antrum, including depressed inter-nodular areas) every 2–3 years in patients with corpus-predominant atrophic-pattern disease, especially when accompanied by autoimmune gastric or systemic comorbidities. Routine surveillance of non-atrophic, antrum-only, or short-duration disease cannot be supported on present evidence and should not be recommended. Any surveillance recommendation should be discussed with the patient in the context of the limitations of the data on which it rests.

## 7 Conclusion

CG is a histologically defined, immune-mediated gastropathy whose clinical recognition has lagged behind its histopathological characterization. Two age-stratified phenotypes dominate the clinical landscape: a pediatric phenotype centered on refractory iron-deficiency anemia and abdominal pain with isolated gastric involvement, and an adult phenotype centered on chronic watery diarrhea within a broader collagenous gastroenteropathy and a higher burden of autoimmune comorbidity. Recent immunological and single-cell transcriptomic data have begun to refine this dichotomy mechanistically, implicating a mixed Th1/Th2 cytokine signature, eosinophil-driven TGF-β1 input, and α4β7-mediated CD4^+^ T-cell recruitment as plausible drivers of subepithelial collagen deposition and atrophic remodeling (Box 1) (Walsh et al., 2026).

BOX 1 **Key points.**

For clinicians

1. Consider CG in any child or adolescent with refractory iron-deficiency anemia and recurrent abdominal pain, and in any adult with chronic watery diarrhea, particularly when collagenous colitis is also suspected.
2. Diagnosis is histopathological. Multiple mapping biopsies—including from the depressed inter-nodular zones where the collagen band is most concentrated—with Masson trichrome staining are mandatory; biopsies confined to the visibly nodular mucosa risk a false-negative result.
3. Three histological subtypes (eosinophil-rich, lymphocytic-gastritis-like, and atrophic) carry distinct clinical and prognostic implications and should be reported explicitly. The atrophic subtype, when corpus-predominant, has the most concerning long-term trajectory.
4. Treatment is symptomatic and individualized: parenteral iron (ferric carboxymaltose) for refractory anemia, topical budesonide for inflammation-driven disease, dietary intervention (single-allergen elimination → amino-acid-based formula in pediatric refractory cases; gluten-free diet only in celiac-overlap cases), and α4β7 blockade with vedolizumab as an emerging mechanism-targeted option for refractory disease.
5. Endoscopic surveillance with mapping biopsies every 2–3 years is reasonable in patients with corpus-predominant atrophic-pattern CG; routine surveillance is not currently supported for non-atrophic, antrum-only, or short-duration disease.

For basic and translational researchers

1. CG is best understood as an immune-mediated disorder with mixed Th1/Th2 cytokine activation, eosinophil-driven profibrotic input via TGF-β1, and substantial CD4^+^ T-cell mucosal homing through the α4β7–MAdCAM-1 axis (Walsh et al., 2026).
2. The reduced epithelial EGF signature reported in CG mucosa is single-study and bioinformatic; independent replication and direct functional validation are required before mechanistic translation.
3. Distinct pediatric (Th2-leaning, allergen-permissive, and isolated-gastric) and adult (autoimmunity-overlapping, atrophic, and multi-segment) phenotypes may reflect divergent immunopathogenic trajectories rather than a continuous disease spectrum; longitudinal cohorts will be required to resolve this.
4. Priority research questions include identification of luminal antigenic triggers, elucidation of fibroblast-to-myofibroblast activation pathways, prospective evaluation of α4β7 blockade in refractory disease, and dedicated EBER-ISH studies to test the proposed but unverified EBV–CG association.

Management remains empirical and largely individualized. The treatment hierarchy outlined in Table 5—symptomatic support with PPIs and parenteral iron, topically targeted budesonide for inflammation-driven disease, dietary intervention in selected pediatric cases, and α4β7 blockade with vedolizumab as a mechanism-targeted option for refractory disease—represents the best current synthesis but rests on retrospective and case-level evidence. The corpus-predominant atrophic subtype, with its rare but documented progression to dysplasia, neuroendocrine cell hyperplasia, and (in one report) EBV-positive gastric adenocarcinoma (Winslow et al., 2001; Narsai et al., 2021), provides the principal biological rationale for endoscopic surveillance.

Several substantive uncertainties remain. The role of EGF deficiency, the mechanistic relationship between CG and collagenous colitis, the contribution of EBV reactivation, and the genetic and environmental determinants of phenotype expression all require independent replication and prospective study. Multi-center disease registries, dedicated single-cell and proteomic datasets, and prospective evaluation of mechanism-targeted therapies—most notably, α4β7 blockade—are the most immediate translational priorities. Until such data accrue, the most actionable contribution of clinicians remains heightened diagnostic awareness: in any child with refractory iron-deficiency anemia or any adult with chronic diarrhea and autoimmune comorbidity, multi-site gastric biopsies with appropriate histochemical stains can transform a frequently overlooked diagnosis into a treatable one.

## Glossary

CG: collagenous gastritis
GI: gastrointestinal
CVID: common variable immunodeficiency
SLE: systemic lupus erythematosus
H&E: hematoxylin and eosin
EGF: epidermal growth factor
HIV: human immunodeficiency virus
Th1/Th2: T-helper 1/T-helper 2
EBV: Epstein–Barr virus
EBER-ISH: EBV-encoded RNA *in situ* hybridization
NBI/M-NBI: narrow-band imaging/magnifying narrow-band imaging
EUS: endoscopic ultrasound
PPI: proton pump inhibitor
**H**_**2**_ **RA**: histamine-2 receptor antagonist
TGF-β1: transforming growth factor-beta 1
PDGF: platelet-derived growth factor
MMP: matrix metalloproteinase
TIMP1: tissue inhibitor of metalloproteinase-1
MAdCAM-1: mucosal addressin cell adhesion molecule-1
ECL: enterochromaffin-like (cell)
HLA: human leukocyte antigen
TTG/EMA: tissue transglutaminase/endomysial antibodies
IEL: intraepithelial lymphocyte
HPF: high-power field
IBD: inflammatory bowel disease
IDA: iron-deficiency anemia
IgA/IgM/IgG/IgG4: immunoglobulin A/M/G/G4
**CCK**_**2**_ **R**: cholecystokinin-2 receptor
TFF2: trefoil factor 2
SNRI: serotonin–norepinephrine reuptake inhibitor
SARS-CoV-2: severe acute respiratory syndrome coronavirus 2
FCM: ferric carboxymaltose
SANRA: Scale for the Assessment of Narrative Review Articles
MALT: mucosa-associated lymphoid tissue
5-ASA: 5-aminosalicylic acid
