# Supplementary material for: Collagenous gastritis: current understanding of a rare immune-mediated gastropathy across pediatric and adult phenotypes—from pathogenesis to therapeutic strategies
Source: Front Cell Dev Biol. 2026 Jul 30;14:1847333. doi: 10.3389/fcell.2026.1847333 (PMC13487584; doi:10.3389/fcell.2026.1847333)
Supplement: Supplementary file 1 [file Table1.docx]

**Supplementary Table 1** Summary of collagenous gastritis patients

| Ref. | Age | Gender | Symptoms | | | | | | Autoimmune diseases association | Endoscopic Findings | | | | *H. pylori* | Collagen band (μm) | | Histological findings | Treatment | Follow-up | Histological changes on follow-up | Clinical course |
| --- | --- | --- | --- | --- | --- | --- | --- | --- | --- | --- | --- | --- | --- | --- | --- | --- | --- | --- | --- | --- | --- |
|  |  |  | Abdominal pain | Nausea, vomiting | Diarrhea | Anemia | Early satiety | Other Symptoms |  | Nodularity | Erythema | Erosion | Others |  | Stomach | Colon |  |  |  |  |  |
| Ref(1) | 15 | Female | Yes (recurrent epigastric pain for 1 year, intermittent) | Yes (hematemesis) | No | Yes (acute anemia) | Not mentioned | Not mentioned | None reported (antibodies to nuclei, parietal cells, intrinsic factor and thyroid were all negative) | Yes (gastric corpus) | Yes (gastric corpus) | Yes (tiny areas of erosion of surface epithelium) | Multiple diffuse scattered discrete submucosal gastric hemorrhages; granularity of gastric corpus | Multiple diffuse scattered discrete submucosal gastric hemorrhages; granularity of gastric corpus | Up to 75 μm (more often averaged 20-30 μm) | No fibrosis (normal colonic biopsies) | Patchy chronic active gastritis with focal atrophy of gastric glands; focal thick band of collagen immediately beneath surface epithelial cells; inflammatory infiltrate of plasma cells, lymphocytes, eosinophils, neutrophils, and macrophages; fibrosis did not extend to deeper portions of lamina propria; focal and patchy process | Ranitidine and sucralfate (2 years)  Furazolidone (2-week course)  Bismuth subsalicylate (discontinued after 2 days due to side effects)  Antacids (occasional use) | 2.5 years (biopsies obtained at 4-month intervals; total of 6 biopsies from gastric body over 2.5-month period) | No improvement (similar process seen in all specimens of the body of the stomach throughout the follow-up period) | No clinical or pathologic improvement despite treatment; daily epigastric pain continued; persistent endoscopic findings of nodularity and erythema |
| Ref(2) | 9 | Female | Yes (intermittent epigastric pain, retrosternal pain) | Not mentioned | No | Yes (severe anemia, hemoglobin 2.8 g/dL) | Not mentioned | Major pallor, gastric bleeding | None (autoantibodies negative: parietal cells, intrinsic factor, mitochondria, endoplasmic reticulum, smooth muscle, antinuclear, thyroid; no IgG and IgA antigliadin, antireticulin, antiendomysium) | No (without nodularity mentioned) | Yes (diffuse erythema) | Yes (erosions in body of stomach and antrum) | No deep ulcerations; normal esophagus and duodenum | Negative (serology, CLO test, 13C-urea breath test, and cultures all negative) | 13 to 96 μm (average close to 40 μm) | No collagen deposits (no features of collagenous colitis) | Subepithelial band of collagen (discontinuous, irregular, containing entrapped dilated capillaries and inflammatory mononuclear cells); surface epithelium showed focal changes with patchy detachment, flattened cells, rare abrasions; chronic gastritis with inflammatory infiltrate (mainly lymphocytes and plasma cells, few eosinophils, exceptional neutrophils); no mucosal atrophy or intestinal metaplasia; increased intraepithelial lymphocytes (12-20 IEL per 100 epithelial cells); overexpression of HLA-DR by epithelial cells; CD25^+^ cells in lamina propria | Initial: transfusion, iron supplement, omeprazole, sucralfate  Later: prednisone (1 mg/kg/day) for 4 months plus omeprazole  Long-term: iron and folic acid supplementation | 30 months (biopsies taken at M0, M2, M17, M18, M20, and M26 over 26-month period; total follow-up 30 months) | No improvement; all gastric specimens showed similar histopathological changes throughout follow-up, including after 4 months of corticosteroid treatment; signs of immune activation remained present | No clinical or pathological improvement despite treatment; anemia recurred when iron supplement was stopped; asymptomatic except for intermittent epigastric pain; patient discharged on iron and folic acid supplementation only |
| Ref(3) | 20 (at presentation) | Male | Yes (epigastric pain for 2 years) | Yes (vomiting) | Yes (watery diarrhea, presented 6 years after initial onset) | Yes (hemoglobin 6.3 g/dl, chronic anemia) | Not mentioned | Easy fatiguability, malaise, pallor, angular stomatitis, weight loss, stool occult blood strongly positive (3+) | Not mentioned | Yes (diffuse nodularity of gastric mucosa, persistent over 6 years) | Not mentioned | Not mentioned | No abnormalities in esophagus or duodenum initially; rectal mucosa normal on sigmoidoscopy | Negative (modified Giemsa stain did not show evidence of *H. pylori*) | Initially 15-43 μm; after 3 years: 23-225 μm (eightfold increase noted over 3 years) | 20-30 μm (rectal biopsies after onset of diarrhea, 6 years after initial presentation) | - Gastric: Diffuse chronic gastritis of body and antrum with focal moderate to severe atrophy; subepithelial collagen bands below surface epithelium with entrapped capillaries, lymphocytes, and eosinophils; surface epithelium partly detached in areas; neutrophilic infiltration initially; increased eosinophils; no increase in intraepithelial leukocytes; fibrosis of lamina propria and smooth muscle upgrowth from muscularis mucosa - Rectal (after 6 years): Mild chronic colitis with patchy subepithelial collagen bands; surface epithelium shortened in foci; few intraepithelial leukocytes; mild infiltrate of plasma cells, lymphocytes, eosinophils, neutrophils; preserved crypt architecture - Ultrastructural: Haphazardly arranged collagen fibers; prominent degranulating eosinophils and mast cells; activated pericryptal fibroblasts in rectal mucosa; reduplication of basement membranes | - H2 receptor blockers - Antihelminthics (hookworm ova found in stools) - Oral iron supplements | 6 years total (upper endoscopy at 3 months, 3 years, and 4 years after initial presentation; rectal involvement noted at 6 years) | Progressive disease - notable eightfold increase in thickness of subepithelial collagen band over 3 years (from 15-43 μm to 23-225 μm); linear extent of band also increased; non-progressive patchy atrophic gastritis; development of collagenous colitis after 6 years | Recurrent symptoms despite treatment over 4 years; chronic blood loss with anemia not improving with treatment; progressive disease with gastric collagen band increasing in thickness; development of watery diarrhea and collagenous colitis 6 years after initial presentation; weight loss; continued gastric nodularity on endoscopy throughout follow-up period |
| Ref(4) | 57 | Male | No | Not mentioned | Yes (loose stools, later bloody diarrhea) | Yes (hypochromic, microcytic) | Not mentioned | 5 lb weight loss | Celiac sprue, mucosal ulcerative colitis | Not mentioned | Yes (gastric erythema, rectosigmoid erythema) | Not mentioned | Duodenal atrophy, rectosigmoid exudate, marked colonic erythema with granularity and ulceration | Negative | Mean 27.07 μm (range 13.9-45.0 μm, mean of measurements 24.95 μm) | No significant subepithelial collagen deposition | Diffuse chronic gastritis, patchy thick bandlike subepithelial collagen (63.7-71.1% of mucosal length), focal superficial epithelial degeneration, numerous intraepithelial lymphocytes (mean 61.5 per 100 epithelial cells), dense lamina propria lymphoplasmacytic infiltrate, entrapped capillaries in collagen | Prednisone, Asacol, gluten-free diet | 3 months after therapy | Not mentioned | Resolution of diarrhea and gastrointestinal bleeding; antigliadin and antiendomysial antibodies became negative |
| Ref(5) | 35 | Female | Not mentioned (abdominal distension mentioned) | Not mentioned | Yes (watery diarrhea) | Yes (watery diarrhea) | Not mentioned | Abdominal distension | Lymphocytic colitis | Yes | Not mentioned | Not mentioned | Diffuse gastric edema | Not reported | Average 20-30 μm (surface epithelium) | Not specified | Patchy surface damage, nonspecific gastritis | Sucralfate, famotidine, sulfasalazine | Yes (duration not specified) | Not reported | Resolution of diarrhea only |
| Ref(6) | 75 | Female | Not mentioned | Not mentioned | Yes (watery diarrhea for 6 months) | Not mentioned | Not mentioned | None mentioned | Collagenous colitis, sprue | Not reported | | | | Not reported | 30-60 μm (antrum and body surface epithelium) | Not specified | Active chronic gastritis | Not given | Not given | Not given | Not given |
| Ref(7) | 67 | Female | Not mentioned | Not mentioned | Yes (watery diarrhea for 2 years) | Not mentioned | Not mentioned | None mentioned | Collagenous sprue, collagenous colitis | No | No | No | Normal endoscopy | Not reported | 60 μm (location not specified) | Present (collagenous colitis diagnosed) | Active chronic gastritis | Loperamide | Yes, rebiopsy performed | Residual gastric collagen at rebiopsy | Not fully described |
| Ref(8) | 11 | Male | Not mentioned | Not mentioned | Not mentioned | Yes (severe anemia) | Not mentioned | None mentioned | None (parietal cells, intrinsic factor, endomysium, endoplasmic reticulum, smooth muscle, mitochondrias, antinuclear, gliadin antibodies all absent) | Yes (scattered submucosal nodules measuring 2-8 mm in gastric corpus) | Not mentioned | Not mentioned | None | Absent | Antrum 50 μm, Fundus 69 μm | Absent (normal mucosa, no collagen deposit) | Moderate density inflammatory infiltrate, 30 IEL/100 epithelial cells, mild glandular atrophy, no intestinal metaplasia, diffuse chronic gastritis with moderate inflammatory activity, infiltration of fundic glands by neutrophils, diffuse fibrosis of lamina propria mainly in fundus | Not specified | Yes, biopsies at 1, 3, 4, and 8 years after initial diagnosis | Moderate decrease in thickness of subepithelial collagen band, decrease in IELs, but collagen band remained abnormal on all samples; cellularity and atrophy degree similar to initial samples | Not fully described |
| Ref(8) | 22 | Female | Not mentioned | Not mentioned | Not mentioned | Yes (severe anemia) | Not mentioned | None mentioned | None (parietal cells, intrinsic factor, endomysium, endoplasmic reticulum, smooth muscle, mitochondrias, antinuclear, gliadin antibodies all absent) | Yes (gastric corpus and antrum appeared diffusely nodular) | Not mentioned | Not mentioned | None | Absent | Antrum 40 μm, Fundus 45 μm | Not available | Moderate density inflammatory infiltrate, 5 IEL/100 epithelial cells, mild glandular atrophy, no intestinal metaplasia, diffuse chronic gastritis with moderate inflammatory activity, diffuse fibrosis of lamina propria mainly in fundus | Not specified | Not mentioned | Not mentioned | Not described |
| Ref(8) | 36 | Male | Not available | | | | | | Not mentioned | Not available | | | | Absent | Antrum 15 μm, Fundus 20 μm | Not available | Mild density inflammatory infiltrate, 3 IEL/100 epithelial cells, no glandular atrophy, no intestinal metaplasia | Not specified | Not mentioned | Not mentioned | Not described |
| Ref(8) | 40 | Female | Not mentioned | Not mentioned | Yes (chronic diarrhea) | Yes (chronic diarrhea) | Not mentioned | None mentioned | Collagenous colitis | No | No | No | Normal gastroscopy and colonoscopy | Absent | Antrum 40 μm, Fundus 41 μm | 20 μm (typical features of collagenous colitis) | Moderate density inflammatory infiltrate, 4 IEL/100 epithelial cells, no glandular atrophy, no intestinal metaplasia, diffuse chronic gastritis with moderate inflammatory activity, diffuse fibrosis of lamina propria mainly in fundus | Prednisolone, salazopyrine, complete parenteral alimentation | Biopsies after 3 months of treatment, then 2 years later | After 3 months: colonic layer 30 μm, no collagen deposit in stomach and duodenum; After 2 years: gastric and colonic biopsies within normal limits | Resolution with treatment |
| Ref(8) | 77 | Male | Not mentioned | Not mentioned | Not mentioned | Not mentioned | Not mentioned | Asymptomatic (gastroscopy performed before major vascular surgery) | None | Not mentioned | Yes (diffuse erythema of gastric mucosa) | Yes (areas of superficial ulcerations and erosions located mainly in fundus) | None | Marked (present) | Antrum 28 μm, Fundus 20 μm | Not available | Mild density inflammatory infiltrate, 14 IEL/100 epithelial cells, no glandular atrophy, no intestinal metaplasia | Not specified | Not mentioned | Not mentioned | Not described |
| Ref(8) | 52 | Female | Not mentioned | Not mentioned | Not mentioned | Yes | Not mentioned | None mentioned | None | No | No | No | Normal gastroscopy and colonoscopy | Marked (present) | Antrum 20 μm, Fundus 15 μm | Absent (normal mucosa, no collagen deposit) | Moderate density inflammatory infiltrate, 4 IEL/100 epithelial cells, mild glandular atrophy, no intestinal metaplasia, diffuse chronic gastritis with moderate inflammatory activity | Not specified | Not mentioned | Not mentioned | Not described |
| Ref(9) | 11 (born October 7, 1981, hospitalized December 1992) | Male | Yes (epigastric sensitivity, later epigastralgia) | Not mentioned | No | Yes (severe: hemoglobin 47 g/L, microcytic, hypochromic) | Not mentioned | Progressive asthenia, pallor, dyspnea on effort, pica behavior (eating mattress foam), enuresis, later polyphagia | None (extensive autoimmune workup negative: vitamin B12, folic acid, basal and stimulated acid secretion under pentagastrin, intrinsic factor search, anti-intrinsic factor antibodies, anti-thyroid hormone antibodies, antinuclear antibodies, anti-smooth muscle, anti-endoplasmic reticulum all negative) | Yes (pseudopolypoid formations in 1995) | Yes (diffuse erythematous gastritis) | Not mentioned | Large folds of fundus, chronic atrophic gastritis appearance on last follow-up (March 1999) | Not mentioned | 200 μm (diagnosed in 1995) | Absent (normal colonoscopy with staged biopsies in March 1999) | Initial: dedifferentiated epithelium, inflammatory infiltrate (lymphoplasmocytes and eosinophils), fibrosis of chorion predominantly in fundus. Six months later: diffuse chronic preatrophic gastritis with rarefaction of glandular cells. Two years later: fibrosis of gastric mucosa, beginning atrophy, inflammatory infiltrate rich in eosinophils, predominantly fundic. 1995: diffuse chronic gastritis predominantly fundic, atrophic with collagen deposits (200 μm) and great richness in eosinophils | Iron supplementation | Multiple endoscopies over 7 years (6 months, 2 years, 3 years after diagnosis, and March 1999) | Macroscopic appearance of mucosal atrophy accentuated, but histological lesions stable; last control (March 1999): chronic atrophic gastritis without histological modification | Initial response to iron therapy (hemoglobin 135 g/L at 6 months), but ferritin remained low. Two years later: stable (hemoglobin 106 g/L) without iron treatment. No lower digestive symptoms over nearly 7 years of follow-up |
| Ref(9) | 11 (born July 28, 1987, hospitalized March 1999; actually 11-12 years old) | Female | Yes (lower limb pain with minimal effort) | Not mentioned | Not mentioned | Yes (severe: hemoglobin 29 g/L, microcytic with MCV 59 fL) | Not mentioned | Progressive asthenia, pallor; normal growth | Anti-smooth muscle antibodies (non-actin type) at 50 IU/mL detected on follow-up | No | Not mentioned | No | Gastritis with large folds, pale mucosa; no macroscopic modification on follow-up endoscopy | Negative | Focal subepithelial collagen deposit (thickness not precisely quantified in abstract, but consistent with early collagenous gastritis lesions) | No collagenous colitis or lymphocytic colitis criteria met (non-ulcerated colitis without specific character) | Unusual gastritis lesions: superficial epithelial and cryptic lesions, inflammatory infiltrate in chorion rich in lymphocytes and eosinophils, associated with focal subepithelial collagen deposit (confirmed by trichrome and chromotrope staining); compatible with early collagenous gastritis lesions. Bone marrow: normal except for hypochromic erythroblasts. Colonic biopsies: non-ulcerated colitis without specific character | Iron supplementation after initial transfusion | 7 months after diagnosis (May 1999, then follow-up exam) | No macroscopic modification on endoscopy; histology identical to initial findings | Asymptomatic at 7 months; hemoglobin improved from 29 g/L to 79 g/L (May 1999), then to 137 g/L with ferritin 17.6 μg/L (under iron treatment) |
| Ref(10) | 74 | Female | Yes (left-sided abdominal pain for 3 months) | Not mentioned | Yes (persistent diarrhea for 3 months) | No (hemogram normal) | Not mentioned | None mentioned | Collagenous colitis | Not mentioned | Yes (mucosal erythema in gastric antrum) | No (no ulcers or erosions) | Gastric antrum diffusely abnormal with loss of vascular pattern and diffuse areas of mucosal pallor; all areas of gastric antrum involved; similar changes in gastric body mucosa but vascular pattern more clearly discerned, particularly on greater curve | Negative (not detected; other organisms also not detected) | Present diffusely (thickness not specified in μm); subepithelial deposits present diffusely throughout gastric antral biopsies and extended proximally to the "transitional zone" with the gastric body | Present (collagenous colitis diagnosed on rectal biopsy; persistent subepithelial collagen deposits on post-treatment biopsies) | Mixed lymphocytic and neutrophilic infiltrate in each biopsy, with some gastric body and antral mucosal glandular atrophy. Subepithelial deposits of collagen present diffusely throughout gastric antral biopsies and extended proximally to transitional zone with gastric body. Ultrastructural studies confirmed subepithelial deposits contained collagen fibres with typical periodic spacing of normally formed collagen. Small intestinal biopsies from duodenal bulb and descending duodenum were normal | Omeprazole 20 mg daily for 28 days (empirical treatment) | Post-treatment endoscopy with multiple biopsies (n=20) from identical sites; symptoms resolved during treatment and had not recurred since completion | Post-treatment biopsies showed identical distribution of gastric mucosal deposits of subepithelial collagen; previously defined foci of polymorphonuclear leukocyte infiltration had completely resolved; persistent subepithelial collagen deposits in both stomach and colon | Symptoms of abdominal pain and diarrhea resolved during omeprazole treatment and have not recurred since completion of treatment course |
| Ref(11) | 27 (at time of report; initially diagnosed at age 15 in 1989) | Female | Yes (recurrent epigastric abdominal pain starting at age 14; continues to have intermittent upper abdominal pain) | Not mentioned (acute hematemesis occurred at age 15) | Not mentioned | Yes (with hypotension at age 15) | Not mentioned | Acute hematemesis, hematochezia at age 15 | None (antiparietal cell antibodies, anti-intrinsic factor antibodies, antinuclear antibodies, and antineutrophil cytoplasmic antigen antibodies all negative) | Yes (gastric body mucosa had nodular appearance on all examinations after 4 months from initial presentation) | Not specifically mentioned | Not mentioned | Initial examination showed multiple diffusely scattered discrete gastric mucosal hemorrhages; 1 week later showed granularity of gastric corpus with resolution of hemorrhages; antral mucosa appeared normal; upper GI contrast studies showed normal pliability and mosaic-like surface pattern within gastric corpus | Negative (urease test negative; Steiner stains on multiple occasions negative) | Up to 75 μm in thickness in gastric corpus; distribution and thickness variable both within and between biopsy specimens; did not appear to become more diffusely distributed or thicker over time; not identified in antrum | Not applicable (colon biopsies remained normal during 12-year study period) | Gastric corpus: Patchy chronic active gastritis with gradual progression in disease severity over 12 years; mixed inflammatory infiltrate (lymphocytes, plasma cells, neutrophils, eosinophils, mast cells); subepithelial collagen bands up to 75 μm in most corpus biopsies; smooth muscle hyperplasia becoming progressively more pronounced over time; progression from moderate to marked mononuclear cell population, from mild to marked neutrophil component, from absence to moderate degree of intestinal metaplasia; patchy glandular atrophy (mild to moderate) with shortening of oxyntic glands but parietal and chief cells present in every corpus biopsy; mild increase in intraepithelial T lymphocytes (14-26 per 100 surface epithelial cells, mean 20); patchy epithelial cell damage and reactive changes; focal sloughing over areas of thickened subepithelial collagen; regenerative surface epithelial changes sufficiently abnormal in some biopsies to warrant interpretation of indeterminate for dysplasia; significant endocrine cell hyperplasia in corpus (average 201 chromogranin-positive cells per linear mm vs 132 in controls, P=0.0003); many biopsies met criteria for linear endocrine cell hyperplasia. Antrum: Mild to moderate mononuclear inflammatory infiltrate in lamina propria; focal mild glandular atrophy; no thickened subepithelial collagen bands; significantly lower number of gastrin cells (average 106 per linear mm vs 161 in controls, P=0.0167). Duodenum: Initially normal, recent development of mild chronic duodenitis. 109 gastric mucosal biopsies from 19 endoscopic procedures examined | Multiple treatments over 12 years: sucralfate (Carafate), ranitidine (Zantac), misoprostol (Cytotec), furazolidone (Furoxone), clarithromycin (Biaxin), metronidazole (Flagyl), omeprazole (Prilosec), and prednisone - all without lasting benefit | 12 years (from age 15 to 27); 19 separate endoscopic examinations; 109 gastric mucosal biopsies (83 from corpus, 26 from antrum) | Gradual progression in disease severity; progression from moderate to marked mononuclear cells, from mild to marked neutrophils, from absence to moderate intestinal metaplasia; glandular atrophy progressed from mild to moderate (patchy); smooth muscle hyperplasia became progressively more pronounced, extending up to surface epithelium in some biopsies; development of endocrine cell hyperplasia in corpus and decrease in gastrin cells in antrum; development of intestinal metaplasia and reactive epithelial changes indeterminate for dysplasia; collagen band distribution and thickness did not appear to become more diffuse or thicker over time; recent development of mild chronic duodenitis in duodenum (previously normal) | No recurrences of gastrointestinal bleeding after initial episode; continues to have intermittent upper abdominal pain; currently not being treated with prescription medications; serum gastrin levels ranged from 25-339 pg/mL (median 114 pg/mL) with only 1 clearly elevated value; borderline elevations in serum gastrin seen only during therapy with ranitidine or omeprazole |
| Ref(12) | 42 | Male | Yes (periumbilical cramps relieved by bowel movements) | Not mentioned | Yes (intermittent diarrhea) | Yes (chronic iron deficiency anemia, mild) | Not mentioned | Rectal bleeding, postprandial flatulence; family history positive for colon cancer | Celiac disease (serology positive for antigliadin, antiendomysial, and antitissue transglutaminase antibodies) | Not mentioned | Yes (mild diffuse gastric erythema) | Not mentioned | Small hiatal hernia, fundic polyp; duodenum showed geographic pattern with scalloping and erythema; colonoscopy revealed 4 colonic polyps. Follow-up endoscopy (3 months): mild antritis and partially recovered distal duodenal mucosa | Negative (modified Giemsa stain; follow-up antrum showed chronic *H. pylori*-negative gastritis) | - Fundus: 10-26 μm (mean 20 μm); discontinuous, involved 30-60% of subepithelial regions (mean 50%) - Body: 10-14 μm (mean 12 μm); focal, encompassing 15% of total biopsy length - Antrum: Initially no thickened subepithelial collagen band; on follow-up (3 months) showed discontinuous band with uniform thickness of 19 μm involving 50% of mucosal length | Normal subepithelial collagen plate; no collagenous or lymphocytic colitis | - Initial biopsies - Fundus and body: Diffuse moderate to marked lymphoplasmacytic infiltrate, rare intraepithelial neutrophils, mild glandular atrophy; surface focally eroded with focal simplification; lymphocytic gastritis in fundus (17-44 IEL/100 epithelial cells, mean 29); body showed slightly increased IELs (13.6/100); thick hyalinized subepithelial collagen band with entrapped capillaries, fibroblasts, inflammatory cells; focal degenerative changes in corresponding surface epithelium; mild deep fibrosis of lamina propria in gastric body - Initial biopsies - Antrum: Similar chronic inflammation to fundus, no neutrophils, mild atrophy; lymphocytic gastritis (12-57 IEL/100 epithelial cells, mean 34.8); no thickened subepithelial collagen band - Duodenum/jejunum: Severe villous atrophy and crypt hyperplasia; moderate to severe transmucosal lymphoplasmacytic infiltrates with rare neutrophils; increased IELs; surface epithelial damage; villi-crypt ratio 0:3 to 1:3; mucosal thickness 0.51 mm; crypt hyperplasia with increased mitotic activity; no collagen plate thickening; no Giardia - Colon: 4 tubular adenomas (0.3-0.8 cm) with low-grade dysplasia in sigmoid, proximal transverse, and distal ascending colon; nonadenomatous colonic mucosa showed normal IELs, normal subepithelial collagen, no surface epithelial degenerative changes - Collagen deposition confirmed by Masson trichrome; Congo red negative for amyloid | Gluten-free diet | 3 months; upper endoscopy with biopsies from stomach (6 fundus and body, 1 antrum) and distal duodenum | - Gastric fundus and body: Residual predominantly superficial mild lymphoplasmacytic infiltrate; no atrophy, no intraepithelial lymphocytosis; subepithelial collagen band discontinuously present in only 1 of 6 mucosal fragments, involving 30% of biopsy length, uniform thickness of 15 μm - Antrum: Chronic H. pylori-negative gastritis with moderate transmucosal lymphoplasmacytic infiltrate and mild atrophy; IELs less numerous (13-28/100 epithelial cells, mean 21); discontinuous subepithelial collagen band with uniform thickness of 19 μm involving 50% of mucosal length (in contrast to initial biopsy which showed no collagen) - Duodenum: Partial height recovery of villi; villi-crypt ratio 1:1 to 2:1; mucosal thickness 0.85 mm; residual mild to moderate chronic inflammation; rare clusters of IELs; mild reactive epithelial changes | - Patient placed on gluten-free diet after initial diagnosis; subsequent improvement of symptoms; asymptomatic at 3-month follow-up; clinical and histologic improvement with partial recovery of duodenal villi and resolution of lymphocytic gastritis in fundus |
| Ref(13) | 20 (in early 20s) | Female | Yes (repeated epigastric pain) | No | No (no intestinal symptoms) | Yes | Not mentioned | None mentioned | Atopic dermatitis (receiving treatment) and history of bronchial asthma | Yes (small polypoid lesions in gastric body; later multifocal pitted atrophic lesions) | Not mentioned | Yes (erosion in gastric body; small ulcers in gastric body at initial presentation) | Impression of "toughness" of gastric wall; multifocal atrophic areas and scars in gastric body; polypoid and atrophic lesions in gastric body on second examination | (urease test negative, tissue culture negative, histological examination negative, serum *H. pylori* IgG negative) | Approximately 30-70 μm thick; distribution varied (collagen band found in some areas but not others) | No pathological findings (no collagenous colitis) | Thick eosinophilic band-like lesion underneath epithelium of gastric mucosa; positive on Azan stains, negative on Congo red stains; immunohistochemically positive for type III collagen and type VI collagen, negative for type I collagen, type IV collagen, and laminin; basal membrane remained without hypertrophy (based on laminin and type IV collagen staining); mucosa with collagen band was atrophic with (pseudo) pyloric metaplasia; marked mononuclear cell infiltration with eosinophils in mucosa with collagen band; mononuclear cell infiltration randomly composed of CD4+ cells, CD8+ cells, CD79a+ cells, and mast cells; intraepithelial lymphocytic infiltration rarely seen (no lymphocytic gastritis); mucosa without collagen band had sparse inflammatory cell infiltration, no atrophy, no intestinal metaplasia; no pathological findings in lower intestine (terminal ileum, cecum, entire colon, rectum); comparisons showed subepithelial collagen deposition not only in erosive, damaged and atrophic mucosa, but also in endoscopically intact mucosa | Proton-pump inhibitor and cytoprotective agent during period between first and second endoscopy | 2 years with 3 endoscopic examinations (initial, 2 months later, 1 year after second exam); colonoscopy performed 1 month after third gastric examination | Thickness of collagen bands did not change significantly between examinations (remained 30-70 μm); all three examinations revealed thick eosinophilic band-like lesion | Continued to have repeated epigastric pain; no intestinal symptoms; collagen band size did not change for 2 years; no development of collagenous colitis during follow-up |
| Ref(14) | 37 | Male | No (no abdominal pain or distress) | Not initially, but developed nausea and vomiting on Asacol trial | Yes (postprandial nonbloody diarrhea with 3-4 loose stools per day) | Initially vitamin B12 deficiency (corrected with supplements); hemoglobin 13.6 g/dL at presentation to their institution | Not mentioned | Profound weight loss (lost 14.8 kg during 1 year from baseline 60.7 kg to 43.6 kg at presentation; cachectic appearance); weakness; some bloating; no fever, dysphagia, odynophagia, or sitophobia | None identified (serum anti-parietal-cell antibody screen negative); initial vitamin B12 deficiency with megaloblastic anemia (but pernicious anemia unlikely as responded well to oral B12) | Yes (nodular gastric mucosa) | Yes (erythematous gastric mucosa) | Not mentioned | Normal esophagus; granular duodenal mucosa; colonoscopy essentially normal except small benign nodules in ileum and colon consistent with lymphoid hyperplasia | Negative (no *Helicobacter*-like microorganisms appreciated; identified as negative in gastric biopsies at other hospital) | 20-120 μm (patchy thickening); most striking finding; varied in thickness | Normal (no collagenous colitis) | Dense, diffuse, superficial lamina propria lymphoplasmacytic infiltrate with occasional neutrophils; number of intraepithelial lymphocytes appeared slightly increased in foveolar surface, which appeared mildly damaged; no lymphoid aggregates; most striking finding was patchy thickening of subepithelial collagen layer (20-120 μm); no significant fibrosis in other areas of lamina propria. Duodenum, ileum, colon: Essentially unremarkable except few prominent lymphoid follicles without lymphoepithelial lesions; no celiac disease, collagenous or lymphocytic enterocolitis, or inflammatory bowel disease; no microorganisms or viral inclusions; lamina propria lymphocytes and plasma cells were polyclonal (IgM, IgG, IgA, κ and λ positive); Bcl-2 staining confirmed benign nature of lymphoid follicles; no lymphoproliferative disorder. Review of prior biopsies from 2 years ago also showed collagenous gastritis with similar degree of subepithelial collagen deposition; no evidence of lymphoproliferative disorder or other enterocolitis | - Prednisone 40 mg daily initially, later tapered - Nutritional supplements - Metronidazole and ciprofloxacin empirically for bacterial overgrowth - Azathioprine (failed to allow prednisone taper; escalating doses up to 200 mg) - Budesonide 9 mg daily (introduced at 20 months to reduce systemic steroid effects) - Calcium, magnesium, phosphate, iron, vitamins D, and vitamin B12 supplements - Previously: Tetracycline for 1 year (for empiric treatment of tropical sprue) - no improvement; Asacol trial (discontinued due to nausea/vomiting) | At least 20 months (currently ongoing); multiple endoscopies with biopsies | Most recent biopsy from gastric antrum still exhibited morphologic features of collagenous gastritis without significant decrease in thickness of collagen band; recent duodenal biopsy revealed no histopathologic abnormality and no lymphoid follicles | - 4 months after starting prednisone: weight increased to 48.6 kg - 8 months: weight reached 55.3 kg as prednisone tapered - Patient could not taper prednisone without relapse (steroid dependent) - 14 months: weight fell to 48.1 kg despite azathioprine; prednisone increased to 30 mg - 16 months: weight improved to 52.2 kg; prednisone down to 20 mg - Currently (20+ months): body weight relatively stable; about 2 stools daily; on prednisone 20 mg, budesonide 9 mg, azathioprine 200 mg plus supplements - Significant weight gain with steroid therapy but persistent collagen band on histology |
| Ref(15) | 11 (initially diagnosed; anemia first detected at age 8) | Male | Yes (intermittent abdominal pain for 3 years; epigastric soreness) | Yes (intermittent blood-tinged vomiting 1-4 times a day) | No | Yes (severe) - hemoglobin 6.8 g/dL at age 8; increased to 11.7 g/dL at age 9 with iron replacement; 10.6 g/dL at presentation (age 11); refractory iron deficiency anemia (did not improve with iron replacement initially) | Not mentioned | Dizziness, pale appearance (at age 8); poor oral intake | None mentioned | Yes (diffuse nodular pattern in mucosa throughout stomach; cobble-stone appearance; polypoid mucosal nodules around pyloric ring) | Not mentioned | Not mentioned | Easy touch bleeding throughout stomach; no remarkable changes in esophagus, duodenum, and colonic mucosa | Negative (CLO test negative at age 9; not detected on pathologic examination; serologic test within normal limits) | - Antrum: Up to 150 μm (thickest) - Fundus and body: Averaged 30-50 μm - Appeared as discontinuous bands with focally irregular extension into deeper part of antral mucosa | No pathologic abnormalities | Similar histopathologic changes in all gastric specimens; subepithelial deposition of thick, homogenous eosinophilic collagen; strongly stained with Masson-trichrome, negative for amyloid with Congo red; collagenous deposition appeared as band-like pattern involving most subepithelial areas but often discontinuous and irregularly extended deeper into proper glands, especially in antrum; collagen bands contained entrapped dilated capillaries and mild inflammatory infiltrates (lymphoplasma cells and eosinophils); mild to moderate atrophy of foveolar glands and focal surface epithelial damages in all biopsies; surface epithelial changes included partial detachment, nuclear stratification, and rare mitosis; moderate inflammatory infiltrate of lamina propria without collagen deposits, composed of lymphocytes, plasma cells and many eosinophils; muscularis mucosa showed marked hypertrophy with frequent smooth muscle up-growth into lamina propria; intraepithelial lymphocytic infiltration rarely seen; neither *H. pylori* nor endocrine cell hyperplasia found; ultrastructural examination: subepithelial collagen bands revealed haphazardly arranged collagen fibrils; focal thickening of basement membrane of proper glands | Oral antacid and iron supplementation | Oral antacid and iron supplementation | Not reported (ongoing follow-up) | Patient being managed with oral antacid and iron supplementation; regular follow-up for monitoring |
| Ref(16) | 19 (at presentation to their center); symptoms since age 4; diagnosed with nodular gastritis and H. pylori at age 15; gastritis persisted at age 16 | Female | Not mentioned | Yes (frequent postprandial vomiting since age 4) | Yes (intermittent diarrhea since age 15) | Yes (iron-deficiency anemia at age 15: hemoglobin 8.4 g/dL, ferritinemia 3 ng/mL) | Not mentioned | Failure to thrive (body mass index 15.2); puberty retardation (menarche at 17 years) | None mentioned; parental consanguinity noted (parents were first cousins) | Yes (diffuse gastric nodular pattern; nodular gastritis at age 15; gastric nodular pattern still present at age 16) | Not mentioned | Not explicitly mentioned | Easy bleeding on contact; gastric findings persisted despite *H. pylori* eradication | Initially positive at age 15; successfully eradicated with treatment; absent at age 16 and at presentation (age 19) | Up to 100 μm; irregular, discontinuous; with some extensions into the lamina propria | Normal (ileocolonoscopy with biopsies showed normal findings; no collagenous colitis) | Subepithelial collagenous deposits found in all gastric specimens; collagenous band irregular, discontinuous, measured up to 100 μm; extensions into lamina propria; entrapped dilated capillaries; mild nonspecific inflammatory infiltrates; partial epithelial detachment at edge of some subepithelial deposits; duodenal biopsies normal (at age 15); jejunoscopy, ileocolonoscopy biopsies normal (at age 19) | - Initially (ages 15-16): Successful *H. pylori* eradication treatment; long course of proton-pump inhibitors; iron supplementation - no improvement in chronic vomiting and diarrhea - At their center (age 19): Prednisone 50 mg/day for 4 weeks with subsequent tapering and cessation | 10 months after prednisone treatment | Not reported (no repeat biopsies mentioned) | - Before prednisone: Chronic vomiting (since age 4) and intermittent diarrhea (since age 15) unchanged despite *H. pylori* eradication, proton-pump inhibitors, and iron supplementation - After prednisone: Rapid clinical remission - At 10 months: Sustained clinical remission |
| Ref(17) | 12 | Female | No (without associated abdominal pain) | Yes (4-month history of nausea and recurrent vomiting) | No | Yes (iron-deficiency anemia; hemoglobin 7.5 mg/dL 6 months before symptoms; returned to normal with iron therapy; hemoglobin 13.1 mg/L at presentation) | Not mentioned | Decrease in appetite but no documented weight loss; pallor | None mentioned; negative antigliadin, tissue transglutaminase, antiendomysial antibodies | Yes (gastric body and fundus appeared thickened and nodular) | Yes (elevated erythematous plaques on repeat endoscopy) | Yes (gastric mucosal erosions; duodenal bulb appeared scarred/deformed) | Initial - esophagus and gastric antrum normal; gastric body and fundus thickened, nodular, friable mucosa with exudates; duodenal bulb with round elevated lesions without ulcerations. Repeat endoscopy (3.5 months later) - severely inflamed gastric body and fundus with elevated erythematous plaques and areas with multiple small polypoid lesions. Follow-up endoscopy (1 year later) - severe erosive gastritis | Initially negative (first 2 biopsies); positive on third gastroscopy (unclear if new acquisition or missed in earlier biopsies) | - Not specified; described as "thickened subepithelial collagen bands". - Thickened subepithelial collagen bands in gastric body (on third biopsy) | Normal (biopsies throughout colon and ileum normal) | - Initial biopsy: Duodenum and antrum normal; duodenal bulb - erosive duodenitis with gastric-type mucosa/gastric heterotopia; stomach body - erosions with focal pseudomembrane formation, acute and chronic inflammation, severe regenerative changes; CMV negative, *H. pylori* negative - Second biopsy (3.5 months): Gastric mucosal erosions, focal atrophy; lamina propria with moderate to severe acute and chronic inflammatory infiltrate with eosinophils and focal fibrosis (not present in first biopsy) - Third biopsy (2 months later): Duodenum - one fragment with preserved villous architecture, second fragment with absence of villi, acute and chronic inflammation with lymphoid formation, thickened subepithelial collagen band; Stomach - moderate atrophy, severe acute and chronic inflammation in lamina propria, severe regenerative changes, focal fibrosis, thickened subepithelial collagen bands; *H. pylori* identified for first time - Follow-up biopsy (1 year later): Severe erosive gastritis macroscopically and histologically | - Initially: Omeprazole 20 mg twice daily - Later: Triple therapy for *H. pylori* (after third biopsy) - Ongoing: Omeprazole intermittently | At least 1 year (follow-up endoscopy performed 1 year after diagnosis) | No resolution - follow-up endoscopy at 1 year showed severe erosive gastritis macroscopically and histologically | - Initially: vomiting ceased, nausea improved but did not resolve on omeprazole - 3 months: symptoms recurred while still on omeprazole - After third biopsy and *H. pylori* treatment: no marked improvement - Currently: continues to have intermittent nausea and vomiting, receives omeprazole intermittently, but gaining weight and functioning well - Pathological findings persistent on follow-up despite treatment |
| Ref(17) | 12 (at presentation); diagnosis of collagenous gastritis at age 13 years 8 months | Female | Yes (intermittent postprandial epigastric pain throughout previous year; continued during follow-up) | Not mentioned | No | Yes (persistent iron-deficiency anemia refractory to oral iron therapy; hemoglobin 8.7 g/dL initially, 9.4 g/dL at presentation; MCV 54; iron 26 μg/dL; ferritin 1.5 ng/mL; increased to 12.0 g/dL with IV iron; drifted down 9 months later; positive occult blood in stool) | Not mentioned | Weight loss (3 months preceding presentation); poor appetite; short stature; pubertal delay (Tanner 1 at age 12); positive occult blood in stool | None mentioned; negative endomysial antibodies | Yes (dense array of raised plaques and nodules in body and fundus with paler intervening areas) | Not mentioned | No (without erosions initially); yes (at age 18 - polypoid mass in fundus) | Esophagus, antrum, duodenum normal macroscopically; second gastroscopy (3 months) - similar macroscopic findings; third gastroscopy (age 13y8m) - macroscopic appearance unchanged; fourth gastroscopy (age 18) - polypoid mass in fundus with significant decrease in plaques and nodules in body | Positive initially (antral biopsies); eradicated with clarithromycin-based triple therapy (documented 2 months later) | - Not specified; described as "thick subepithelial collagen band". - Thick subepithelial collagen band (on third biopsy at age 13y8m) | Normal (ileocolonoscopy normal) | - Initial biopsy: Duodenum and esophagus normal; antrum - chronic active gastritis with *H. pylori* (Giemsa stain); gastric body - chronic active gastritis (mainly lymphocytes and plasma cells) - Second biopsy (3 months): Similar macroscopic findings; inflammatory component with numerous eosinophils, intraepithelial eosinophils, focal atrophy - Third biopsy (age 13y8m): Macroscopic appearance unchanged; thick subepithelial collagen band, dense polyclonal infiltrate of plasma cells and eosinophils with eosinophilic crypt abscesses, areas of atrophic gastritis - diagnosis of collagenous gastritis made - Biopsy after 6 months of prednisone: No change despite marked clinical improvement - Biopsy at age 18: Polypoid mass consisting of inflammatory infiltrate with subepithelial collagen deposits | - Initially: Omeprazole for 2 months; Clarithromycin-based triple therapy for *H. pylori* - Intravenous iron (anemia refractory to 3 months oral iron) - Renewed: Omeprazole - Nutritional supplementation - Prednisone for 10 weeks (after diagnosis at age 13y8m) - Later: Intermittent omeprazole for 2-3 months as needed | Long-term follow-up of 6 years (age 12 to 18) | - No change after 6 months of prednisone despite marked clinical improvement - At age 18: polypoid mass with inflammatory infiltrate and subepithelial collagen deposits | - 3 months after initial endoscopy: ongoing abdominal pain, poor appetite, no weight gain despite omeprazole and *H. pylori* eradication - 9 months: hemoglobin started to drift down, underweight but otherwise asymptomatic - After 10 weeks of prednisone: Significant clinical improvement - weight gain, growth spurt, normal appetite; reached 10th centile for weight over 6 months; by age 15.5 reached 50th centile for height and weight - Following 2 years: failed regular clinic visits; intermittent epigastric pain requiring occasional omeprazole; blood counts remained normal, anemia did not recur - Marked clinical improvement with prednisone but no histological resolution |
| Ref(17) | 12 | Female | Yes (few months duration; epigastric pain with heartburn) | No | No | No (normal blood count, normal iron levels) | Not mentioned | Heartburn; normal appetite; normal weight | None mentioned; negative celiac screening | Yes (nodular appearance in gastric body and antrum) | Yes (erythema in duodenum) | Yes (erosions in stomach and duodenum) | Duodenum with erythema and erosions | Negative (rapid urease test negative); father had *H. pylor*i gastritis 1 year before | - Not specified; described as "patchy subepithelial collagenous deposition". - Patchy subepithelial collagenous deposition in gastric body | Not examined | Gastric biopsy - oxyntic mucosa with chronic inflammation and patchy subepithelial collagenous deposition; duodenal biopsy - normal | Proton pump inhibitors (PPIs) (omeprazole) | 2 months | Not reported (no repeat biopsy mentioned) | Improvement in symptoms (abdominal pain and heartburn) on follow-up 2 months after starting omeprazole |
| Ref(18) | 35 (at presentation to their center); had gastritis diagnosed 14 years previously (age 21) | Male | No (asymptomatic at presentation); history of epigastric pain 14 years previously | No | No | No (normal laboratory data) | Not mentioned | No gastrointestinal symptoms at presentation (completely asymptomatic; detected on screening); epigastric pain 14 years ago improved immediately after diagnosis | None mentioned; no history of allergic disease or food allergy | Yes (diffuse nodularity of gastric mucosa in antrum and greater curvature of gastric body; composed of mucosal nodules 1-5 mm in diameter; nodular appearance became unclear when stomach filled with sufficient air; nodularity already present in gastric body 14 years previously but less prominent; nodular appearance became prominent in antrum over 14-year period and supplemented in body) | Not mentioned | No (mucosa between nodules was discolored and relatively depressed but did not cause erosive change) | - Upper GI barium radiography: abnormal surface with mosaic-like pattern in body of stomach - Normal-looking mucosa seen in greater curvature of lower body between nodular lesion in antrum and lesion in gastric body - No gastric hemorrhages or ulcerative lesions - Chromoendoscopy: mucosa between nodules discolored and relatively depressed - Mucosal nodularity spread widely to all of greater curvature of gastric body - Esophagus, duodenum, terminal ileum, colorectum normal on endoscopy - Small intestine normal on barium radiograph | Negative (anti-*H. pylori* IgG and urease breath test both negative); absent on histology | - Biopsy from nodule in gastric body: Thickened subepithelial collagen band <10 μm with no remarkable inflammation - Mucosa near nodule: Thick collagen band 50-100 μm with prominent inflammatory infiltration - 14 years previously: Subepithelial collagen band averaging 20 μm with mild inflammation - Progression over 14 years: from 20 μm → 50-100 μm | Normal (biopsies from colorectum and terminal ileum normal, no collagenous deposit) | - Biopsy from nodule in greater curvature of body:   - Lamina propria lymphoplasmacytosis  - Thickened subepithelial collagen band <10 μm with no remarkable inflammation  - Mucosa not thickened  - Glandular structure well preserved   - Mucosa near nodule:   - Thick collagen band 50-100 μm  - Prominent infiltration of inflammatory cells (lymphocytes and eosinophils)  - Inflammatory infiltrates remarkable and destructive to glands, resulting in mucosal thinning  - Collagen band mucosa always associated with increased inflammatory cells  - Collagen plate contained many entrapped superficial capillaries and inflammatory cells  - Focal surface epithelial damage (epithelial cell flattening and partial detachment)   - General findings:   - Findings varied and irregular even within biopsy specimens  - Collagen band confirmed by Masson trichrome staining  - Sydney system: moderate chronic interstitial inflammation, mild neutrophil polymorph activity  - Mild atrophy with type of pseudopyloric gland  *- H. pylori* absent   - Lesser curvature (without nodules): Mild chronic inflammation - Duodenum, colorectum, terminal ileum: Normal, no collagenous deposit - 14 years previously: Subepithelial collagen band averaging 20 μm with mild inflammation | No medical treatment (followed up without medication) | 3 years after diagnosis (also reviewed endoscopy from 14 years previously) | - Progression over 14 years:   - Collagen band thickness: 20 μm → 50-100 μm  - More prominent inflammation  - No repeat biopsy after diagnosis mentioned | - 14 years previously (age 21): Epigastric pain, diagnosed with gastritis; symptoms improved immediately, no epigastric discomfort afterward, received no medical treatment - At presentation (age 35): Completely asymptomatic (detected on screening) - Endoscopic progression over 14 years:   - Nodular appearance became more conspicuous  - Extended to antrum (not present 14 years ago)  - Supplemented in body   - Histological progression over 14 years: More thickened collagen band - 3 years after diagnosis: Remains asymptomatic (no symptoms without medication) |
| Ref(19) | 9 | Female (Caucasian) | Yes (one-year history of epigastric pain) | No (without any associated vomiting) | No (without any associated diarrhea) | Yes (severe iron deficiency anemia; hemoglobin 5.9 g/dl [normal 11.7-15.3], MCV 67 fl [normal 83-100], serum ferritin 3 μg/L [normal 15-200]; anaemia recurred on stopping iron supplements; responded well to oral iron initially with Hb increasing to 12.8 g/dl; needed prolonged course of iron supplementation; for last 18 months completely asymptomatic with normal blood counts and ferritin levels) | Not mentioned | Pallor, glossitis, koilonychia; positive stool occult blood test; thriving well with weight and height along 75th centile | None; negative anti-gliadin and anti-endomysial antibodies; negative autoimmune markers; normal immunoglobulins | Yes (nodularity of gastric corpus and fundus) | Yes (erythema of gastric corpus and fundus) | Not mentioned | - Duodenum and esophagus normal - Four upper GI endoscopies over 50-month follow-up: gastric findings both at endoscopy and histology unchanged - One colonoscopy: normal | Negative (negative *H. pylori* serology; negative urease breath test; no *H. pylori* on H&E stain, Giemsa stain, or immunohistochemical staining) | - Prominent subepithelial pink zone with entrapped capillaries and a few chronic inflammatory cells - Discontinuous collagenous zone highlighted with Masson trichrome stain - Varied in thickness, measuring up to 35 μm in maximum thickness - Band did not decrease in thickness in sequential biopsies | Normal (all colonic biopsies normal on H&E and Masson trichrome staining) | - All gastric body and antral biopsies:   - Diffuse chronic gastritis of moderate severity  - Inflammation composed of scattered eosinophils, plasma cells, lymphocytes, lymphoid aggregates and lymphoid follicles with germinal centers  - Regenerative changes in surface epithelium  - Associated mild atrophy of gastric pits  - Third and fourth biopsies: mild focal activity with neutrophils present in lamina propria and infiltrating surface epithelium  - No *H. pylori* (H&E, Giemsa, immunohistochemical staining)  - No erosions, ulcers or granulomas  - No intestinal metaplasia  - No increase in intraepithelial lymphocytes   - Collagen findings:   - All gastric biopsies showed prominent subepithelial pink zone with entrapped capillaries and few chronic inflammatory cells  - Discontinuous collagenous zone highlighted with Masson trichrome stain  - Varied in thickness, up to 35 μm maximum  - Band did not decrease in sequential biopsies  - Elsewhere, lamina propria did not exhibit increased collagen  -Immunohistochemical staining for Collagen IV: basement membrane of normal thickness   - Duodenal, esophageal, colonic biopsies: All normal on H&E and Masson trichrome staining | - Oral iron supplements (responded well initially; needed prolonged course) - Omeprazole (epigastric pain responded partially) - For last 18 months: no treatment and completely asymptomatic | 50 months; four upper GI endoscopies and one colonoscopy during follow-up | Unchanged - gastric findings both at endoscopy and histology were unchanged over 50 months; collagen band did not decrease in thickness in sequential biopsies | - Initially: Severe anemia; responded well to oral iron with Hb increasing to 12.8 g/dl - After stopping iron: Anaemia recurred; needed prolonged iron supplementation - Epigastric pain: Responded partially to omeprazole - During 50-month follow-up: Gastric findings unchanged both endoscopically and histologically - Last 18 months: Completely asymptomatic without any treatment; normal blood counts and ferritin levels - Remarkable: Spontaneous clinical remission despite persistent endoscopic and histological abnormalities |
| Ref(20) | 25 | Male | Yes (epigastric pain) | Not mentioned | No (no history of watery diarrhea) | Not mentioned (no mention of anemia or bleeding) | Not mentioned | Did not have any other symptoms; not on any medications | Not mentioned; however, authors note no symptomatic or clinically evident celiac disease | Not mentioned (gastric and duodenal mucosa was normal at endoscopy) | Not mentioned | Not mentioned | - Gastric and duodenal mucosa normal at endoscopy - Only random gastric biopsy taken - Colonoscopy was not done | Positive (*H. pylori* infection was present; *H. pylori* organisms visible on surface) | - Markedly thickened, frayed subepithelial collagen band - No specific measurement given in micrometers - Thick prominent collagen table - Entrapped capillaries and inflammatory cells within thickened collagen band | Not evaluated (colonoscopy not done; no evidence of collagenous colitis) | - Typical features of collagenous gastritis:   - Markedly thickened, frayed subepithelial collagen band  - Within thickened collagen table: entrapped capillaries and inflammatory cells  - Artifactual detachment of overlying epithelium  - Dense homogenous material highlighted with Masson's trichrome stain, confirmed to be collagen   - *H. pylori* infection: Present - No increase in intra-epithelial lymphocytes (no lymphocytic gastritis) - Stains for amyloid: Negative - Authors note characteristic features:   - Collagen table frequently ragged and frayed of variable thickness  - Artifactual detachment of surface epithelium, exposing underlying collagen band | Not mentioned | Not mentioned | Not applicable (no follow-up mentioned) | Not described (only presenting features mentioned) |
| Ref(21) | 9 | Female | Yes (intermittent nonspecific abdominal pain) | No | No | Yes (severe; hemoglobin 5.7 g/dl, MCV 48 fl, low iron saturation and total iron level; reticulocytes increased; previous history of iron deficiency anemia treated with iron supplementation before adoption at 18 months) | Not mentioned | Fatigue, decreased exercise tolerance, pallor; stool heme-occult positive | None mentioned; celiac screening normal | Yes (diffuse gastric nodularity) | Yes (associated erythema) | Not mentioned | Not specified | Negative (*Helicobacter* antibodies normal) | Not specified (biopsies revealed collagenous gastritis) | Not evaluated | - Biopsies revealed collagenous gastritis - No specific details provided | Iron therapy initiated | Several months to years | Not mentioned | - Over next 4 months: Fatigue and pallor improved with complete resolution of laboratory abnormalities - After iron discontinued: MCV and iron indices slightly abnormal but remained asymptomatic (without abdominal pain) - After iron restarted: Labs normalized again - Current status: Remains asymptomatic with normal laboratory studies - No other treatment considered since symptoms abated |
| Ref(21) | 15 | Male | Yes (intermittent sharp left upper quadrant pain a few times per week after 2 months; generalized abdominal pain when relapsed) | Not mentioned initially | YES - Major presenting symptom:   - Non-bloody diarrhea - Initially: loose, "mashed potato-to-watery" consistency, 3-5 times/day - Increased to 10-15 stools per day over first 2 months - Associated urgency and tenesmus - Voluminous watery diarrhea (characteristic of adult-onset disease) - On relapse: loose, non-bloody, non-mucousy stools (approx 2-3 times/day) | Yes (developed pancytopenia: WBC 2.9 K/mm³, absolute lymphocyte count 551/mm³, absolute neutrophil count 1450/mm³, platelets 143 K/mm³, hematocrit 24.7%; autoimmune hemolytic anemia diagnosed) | Not mentioned | - Large number of oral ulcers - Intermittent fever (up to 102°C) - 10 lb weight loss - Fatigue (worsened) - Pallor - Splenomegaly - Early childhood history of food allergies - Denied joint pain, loss of appetite, or rash | - Autoimmune hemolytic anemia with pancytopenia secondary to splenic sequestration - Positive serologies:   - Anticardiolipin IgM: 23 MPL (normal <15)  - Cbir1: 39.9 EU/ml (normal <21)  - Anti-gliadin IgA: 12 U/mL (normal <11)  - Anti-gliadin IgG: 99 U/mL (normal <11) - significantly elevated   - Tissue transglutaminase: Normal (no celiac disease) - Normal: IgG, IgM, IgA, IgE, C3 complement, CH50 - Low: C4 complement (10 mg/dL; normal 16-52) - Negative: ASCA IgA/IgG, Anti OmpC, pANCA | - Initial colonoscopy (after 3 months of symptoms):   - Grossly normal  - Histology: subepithelial collagen deposition   - Repeat endoscopy and colonoscopy (after relapse):   - Esophagus: Normal appearing  - Stomach: Mild patchy gastric erythema  - Duodenum: Moderate flattening, scalloping and erythema throughout  - Terminal ileum: Normal appearing  - Colon: Normal appearing | | | | Not mentioned | Mild/moderate chronic active gastritis with patchy subepithelial collagen deposition (some areas >30 microns) | Pancolonic diffuse moderate chronic active colitis with foci of marked subepithelial collagen deposition (30-50 microns) | - Esophagus: Normal - Stomach:   - Mild/moderate chronic active gastritis  - Patchy subepithelial collagen deposition (some areas >30 microns)   - Duodenum:   - Moderate chronic duodenitis  - Patchy villous blunting  - Mild subepithelial collagen accumulation   - Terminal ileum:   Patchy enteritis with lymphoid hyperplasia   - Colon:   - Pancolonic diffuse moderate chronic active colitis  - Foci of marked subepithelial collagen deposition (30-50 microns)   - Bone marrow biopsy:   - Hypercellular bone marrow  - Marked erythroid hyperplasia  - Megaloblastic changes  - Left shift in myeloid maturation with many bands (consistent with peripheral consumption)  - Negative for malignant cells   - Pathology: Consistent with collagenous gastritis AND colitis | - For autoimmune hemolytic anemia:   - Prednisone 65 mg/day → normalization of blood counts and resolution of GI symptoms  - Slowly weaned over 5 months   - After relapse (1 month post-prednisone cessation):   - Lansoprazole  - Mesalamine | Over 1 year | Not described (only one set of biopsies mentioned after relapse) | - Initial presentation:   - Non-bloody diarrhea → worsening over 2 months (3-5 to 10-15 stools/day)  - After 2 months: developed LUQ pain, oral ulcers  - After 3 months: labs initially normal; colonoscopy grossly normal but showed collagenous colitis  - Weeks later: fatigue and diarrhea worsened; developed pancytopenia   - Diagnosis:   - Autoimmune hemolytic anemia with pancytopenia secondary to splenic sequestration  - Collagenous gastritis and colitis   - On prednisone:   - Blood counts normalized  - GI symptoms resolved   - Prednisone weaned over 5 months   1 month after prednisone cessation:  - Relapse: loose stools (2-3/day), generalized abdominal pain  - Repeat endoscopy/colonoscopy: confirmed collagenous gastritis/colitis   - On lansoprazole + mesalamine:   - Symptoms resolved  - Remained asymptomatic on therapy for over 1 year |
| Ref(22) | 16 (at initial presentation), followed up to age 23 years | Female | Yes (epigastric pain radiating to left shoulder) | Not mentioned | No (no change in bowel pattern) | Yes (Hb 6.0 g/dl initially, 10.5 g/dl at follow-up) | Not mentioned | Black stools, dysmenorrhea | None documented (EMA negative) | Yes (irregular, nodular gastric mucosa) | Yes (erythema on greater curve) | Not mentioned | Initially normal endoscopy, later showed irregular gastric mucosa on greater curve | Negative | >10 μm (exact measurement not specified) | No collagen band (normal colonic biopsies) | Patchy collagenous gastritis; distal duodenal biopsies normal | Ranitidine, omeprazole, iron sulphate, cessation of NSAIDs (mefanamic acid), exclusion diet; later H2 antagonist and PPI combination | 7 months after initial presentation and again at age 23 (approximately 6 years later) | Persistent collagenous gastritis on repeat biopsies at 7 months and 6 years | Symptoms initially improved with treatment and exclusion diet. Relapsed at age 23 with epigastric pain, fatigue, possible melaena, and anemia. Persistent disease over 6+ years |
| Ref(22) | 39 | Female | No | Not mentioned | Yes (stool frequency 2-3 times per day, loose, sometimes pale and buoyant) | Yes (Hb 8.0 g/dl, MCV 68.2 fl; iron deficiency) | Not mentioned | Fatigue, angular stomatitis, low vitamin B12 | - Coeliac disease (atypical, Marsh type I with persistent duodenal intraepithelial lymphocytosis) - Hypothyroidism (TSH 45.5 mU/l) | No | No | No | Macroscopically normal gastric mucosa on all endoscopies | Negative | >10 μm (exact measurement not specified) | One biopsy showed thickening with collagen (possible collagenous colitis) | Collagenous gastritis; distal duodenum showed normal villous architecture but increased intraepithelial lymphocytes; one colonic biopsy showed increased inflammatory cells and thickened subepithelial membrane with collagen | Iron and B12 replacement initially; gluten-free diet and thyroid replacement | Over 4 years with recurrent iron deficiency and multiple endoscopies | Persistent duodenal intraepithelial lymphocytosis over multiple biopsies; collagenous gastritis diagnosed on 4th endoscopy | Recurrent iron deficiency over 4 years despite initial treatment. Now well on gluten-free diet and thyroid replacement |
| Ref(23) | 15 (Female), with 7 follow-up biopsies over 41 months | Female | Yes (epigastric pain) | Not mentioned | No | Yes | Not mentioned | None | None (HLA DQ2 carrier with strong family history of celiac disease, but patient had normal duodenal biopsies and negative celiac serologies) | Yes | Not mentioned | Not mentioned | None | Negative (negative serology) | 33-69 μm (diffuse) initially; decreased to 18-40 μm (patchy) at 32 months | Normal | Moderate chronic inflammation with mild neutrophil inflammation; variable intraepithelial lymphocytes (5-9 per 100 epithelial cells); no intestinal metaplasia or atrophy | Budesonide | 7 biopsies over 41 months (3, 12, 16, 24, 32, 41 months) | Persistent collagenous gastritis with decrease in collagen thickness and change from diffuse to patchy distribution | Symptomatic improvement with Budesonide; persistent disease over 41 months with decreased collagen thickness |
| Ref(23) | 14 | Female | No | Yes | No | No | Not mentioned | None | None | No | Yes | Not mentioned | None | Negative (negative immunohistochemistry and serology) | 23 μm (patchy in body/fundus) | Not available | Mild chronic inflammation; 32 intraepithelial lymphocytes per 100 epithelial cells (lymphocytic gastritis); prominent eosinophilic infiltrate | Protonix and Carafate | Not available | Not available | No symptomatic improvement after treatment |
| Ref(23) | 13 | Female | No | Not mentioned | No | Yes | Not mentioned | Dysphagia secondary to Plummer-Vinson syndrome | Plummer-Vinson syndrome | Yes | Not mentioned | Not mentioned | None | Negative (negative immunohistochemistry) | 26-76 μm (patchy in both antrum and body/fundus) | Not available | Moderate chronic inflammation; 7 intraepithelial lymphocytes per 100 epithelial cells; prominent eosinophilic infiltrate; 2 prior biopsies (7 and 6 months before) showed inactive chronic gastritis without collagenous gastritis | None documented | No follow-up | Not available | No follow-up available |
| Ref(23) | 19 | Male | Yes | Not mentioned | No | Yes | Not mentioned | Perforating gastric ulcers (50 months before diagnosis) | None | Yes | Not mentioned | Not mentioned | Had partial gastrectomy 50 months before for perforating ulcers | Not mentioned | 20-40 μm (diffuse in antrum, patchy in body) initially; no evidence of collagenous gastritis at 3-month follow-up | Normal | Mild chronic inflammation; 6 intraepithelial lymphocytes per 100 epithelial cells initially; 33 at 3-month follow-up (lymphocytic gastritis without collagenous gastritis); 4 prior biopsies and partial gastrectomy specimen showed inactive chronic gastritis without collagenous gastritis | Carafate | 3 months | No evidence of collagenous gastritis at 3 months; developed lymphocytic gastritis (not present initially) | Symptomatic improvement after treatment; collagenous gastritis resolved but lymphocytic gastritis developed |
| Ref(23) | 62 | Female | No | Yes | No | No | No | None | Hashimoto thyroiditis and polymyositis | No | No | Not mentioned | Atrophic appearance | Negative | 29-94 μm (diffuse in antrum, patchy in body) | Not available | Moderate chronic inflammation; <5 intraepithelial lymphocytes per 100 epithelial cells; prominent eosinophilic infiltrate | Not documented | No follow-up | Not available | No follow-up available |
| Ref(23) | 52 | Female | Yes | Not mentioned | Yes | No | Not mentioned | None | Celiac sprue (clinical diagnosis) and questionable ulcerative colitis | No | No | Not mentioned | Atrophic appearance; colonic polyps | Negative (negative immunohistochemistry) | Negative (negative immunohistochemistry) | Not available | Mild chronic inflammation with mild neutrophil inflammation; <5 intraepithelial lymphocytes per 100 epithelial cells; duodenal biopsy showed celiac sprue | Gluten-free diet | Not available | Not available | Symptomatic improvement on gluten-free diet |
| Ref(23) | 57 | Female | Yes | Not mentioned | Not mentioned | Not mentioned | Not mentioned | None | Celiac sprue | Yes | Not mentioned | Not mentioned | None | Negative | 19.5-40 μm (patchy in body/fundus; antrum not available) | Not available | Mild chronic inflammation; 35 intraepithelial lymphocytes per 100 epithelial cells (lymphocytic gastritis); duodenal biopsy showed celiac sprue | Gluten-free diet | Not available | Not available | Symptomatic improvement on gluten-free diet |
| Ref(23) | 80 | Male | No | Not mentioned | Yes (chronic) | Yes | Not mentioned | None | Collagenous colitis | Yes | Not mentioned | Not mentioned | Normal colon on endoscopy | Negative (negative immunohistochemistry and serology) | 20-47 μm (diffuse in both antrum and body) | Collagenous colitis present | Moderate chronic inflammation with mild neutrophil inflammation; 12 intraepithelial lymphocytes per 100 epithelial cells; duodenal biopsy normal; colonic biopsy showed collagenous colitis | Budesonide | No follow-up | Not available | No follow-up available |
| Ref(23) | 56 | Male | No | Yes | No | No | Not mentioned | Weight loss | Celiac sprue | Yes | Not mentioned | Not mentioned | Normal colon | Negative | 24-87 μm (diffuse in both antrum and body) | Normal | Mild chronic inflammation; <5 intraepithelial lymphocytes per 100 epithelial cells; duodenal biopsy showed celiac sprue; colonic biopsy normal | Prednisone and gluten-free diet | Not available | Not available | Mild symptomatic improvement on prednisone and gluten-free diet |
| Ref(23) | 34 | Male | Yes | Not mentioned | No | No | Not mentioned | Upper gastrointestinal bleeding (history since late teens) | None | Yes | Not mentioned | Not mentioned | None | Negative | 62-78 μm (diffuse) initially; increased to 70-120 μm (diffuse in antrum) at 119 months | Not available | Moderate chronic inflammation with mild neutrophil inflammation initially; moderate chronic inflammation at 119 months; intraepithelial lymphocytes decreased from 10 to 6 per 100 epithelial cells; focal intestinal metaplasia developed at 119 months | Transfusion of packed red blood cells | 3 biopsies over 119 months (22 and 119 months) | Persistent collagenous gastritis with dramatic increase in collagen thickness (78 μm to 120 μm) over 10 years; focal intestinal metaplasia developed; diffuse antral involvement but no collagenous gastritis in fundus at 119 months | No further bleeding episodes; no medical therapy administered; progressive increase in collagen thickness |
| Ref(23) | 68 | Male | No | Not mentioned | Yes | No | Not mentioned | Weight loss | Collagenous sprue | No | Not mentioned | Not mentioned | Atrophic appearance | Negative | 20 μm (patchy in antrum) initially; increased to 30-80 μm (patchy) at 9 months | Not available | Moderate chronic inflammation with variable neutrophil inflammation; 22-41 intraepithelial lymphocytes per 100 epithelial cells (lymphocytic gastritis); duodenal biopsy showed collagenous sprue with negative T-cell receptor rearrangement | Prednisone and gluten-free diet | 3 biopsies over 10 months (8 and 9 months) | Persistent collagenous gastritis with increase in collagen thickness (20 μm to 30-80 μm); persistent lymphocytic gastritis | No symptomatic improvement; refractory to gluten-free diet and symptomatically steroid-dependent |
| Ref(23) | 59 | Male | No | Not mentioned | No | No | Not mentioned | Dysphagia | None | No | Not mentioned | Not mentioned | Normal gastric endoscopy; Barrett esophagus surveillance | Negative | 39-54 μm (patchy in cardia only) | Not available | Moderate chronic inflammation with mild neutrophil inflammation; <5 intraepithelial lymphocytes per 100 epithelial cells; only cardia-type mucosa sampled | Not available | Not available | Not available | Not available |
| Ref(24) | 12 | Female | No (asymptomatic) | No | No | Yes (severe - Hemoglobin 4.8 g/dL, Hematocrit 17.3%) | Not mentioned | Pallor; stools intermittently positive for occult blood; low serum iron | Autoimmune hypothyroidism (developed during follow-up); gastric parietal cell antibodies were negative | Yes (striking nodularity of the gastric body with smooth peninsula-shaped areas) | Not mentioned | No erosions | Relative antral sparing; normal appearance of other areas of upper and lower gastrointestinal tracts; described as "chronic varioliform gastritis" appearance | Negative (no *Helicobacter*) | Present (thickened subepithelial collagenous band within the superficial lamina propria - specific thickness not measured) | Colonoscopic biopsies showed no collagenous band | Moderate-to-marked chronic and eosinophilic inflammation; collagenous band within the superficial lamina propria; no *Helicobacter* or granulomas; no intraepithelial lymphocyte count mentioned | Oral iron supplementation; elimination diet trial (minimal if any response) | Long-term follow-up available (duration not specified) | Not mentioned (no repeat biopsies described) | - Anemia corrected with oral iron supplementation - Patient remained asymptomatic with normal growth - Required ongoing oral iron supplementation - Developed autoimmune hypothyroidism during follow-up - No peripheral eosinophilia - Minimal if any response to elimination diet - Normal serum albumin, celiac screen, anti-Saccharomyces cerevisiae antibodies, anti-neutrophil cytoplasmic antibodies, and erythrocyte sedimentation rate - Normal gastrin and pepsinogen II levels - Denied nonsteroidal anti-inflammatory drug use and long-distance running |
| Ref(25) | 9 months at presentation; followed for 14 years (until age 14 years) | Male | Not mentioned | Yes (chronic intermittent vomiting since 15 days from birth) | Yes (watery diarrhea; chronic intermittent diarrhea since 15 days from birth; acute severe dehydration at presentation) | Not mentioned | Not mentioned | Low weight for height; hypoproteinemia (3.5 g/100 mL); hypoalbuminemia (1.4 g/100 mL); hypogammaglobulinemia (4.6 g/L); lymphocytosis (8.4 G/L) | None mentioned | - At 9 months (initial presentation): No - At age 3 years: Not mentioned - At age 10 years: Yes (multiple small submucosal nodules in stomach/duodenum; diffuse nodular colonic mucosa) | - At 9 months (initial presentation): Not mentioned - At age 3 years: Yes (erythematous mucosal areas in colon) - At age 10 years: Yes (diffuse erythema in duodenum) | - At 9 months (initial presentation): Yes (gastric erosions) - At age 3 years: Not mentioned - At age 10 years: Not mentioned | - At 9 months (initial presentation): Atrophic gastric mucosa; no other alterations - At age 3 years: Pseudopolyps with inflammatory areas in the gastric body - At age 10 years: Thick small tubular stomach; disappearance of gastric folds; diffuse atrophic areas extending to duodenum; colonic mucosa diffusely pale, thickened, and nodular with disappearance of normal vascular pattern | Not mentioned | - At 9 months (initial presentation): 50 μm (diffuse irregular thickening of subepithelial collagen tissue) - At age 10 years: Gastrointestinal tract: 12-100 μm (diffuse; progressive increase in subepithelial collagen tissue throughout gastrointestinal tract) | - At 9 months (initial presentation): 12-17 μm - At age 10 years: Gastrointestinal tract: 12-100 μm (diffuse; progressive increase in subepithelial collagen tissue throughout gastrointestinal tract) | - At 9 months: Diffuse irregular thickening of subepithelial collagen tissue; erosive epithelium; jejunal villous atrophy (grade III); gastric and colonic lymphocytic infiltrate with collagen deposits - At age 10 years: Diffuse atrophic mucosa; increase in subepithelial collagen tissue (12-100 μm) throughout gastrointestinal tract | - Prednisolone 0.5-1 mg/kg/day, alternating with budesonide 3-4 mg/day - Night enteral nutrition - Gluten-free diet - Total parenteral nutrition started at age 14 years - Corticotherapy discontinued 2 months after starting total parenteral nutrition | 14 years (from 9 months to age 14 years) with multiple endoscopic and histological evaluations at ages 9 months, 3 years, 10 years, and 14 years | - Progressive disease with increase in subepithelial collagen tissue from 12-50 μm at 9 months to 12-100 μm at age 10 years - Development of diffuse atrophic mucosa throughout gastrointestinal tract - Persistence of collagenous deposits in stomach, duodenum/jejunum, and colon | - Despite 13 years of aggressive treatment (corticosteroids, enteral nutrition, gluten-free diet), clinicopathological disease progression was observed - Progressive endoscopic changes: from atrophic gastric mucosa with erosions at 9 months → pseudopolyps with inflammatory areas at age 3 → thick tubular stomach with disappearance of folds and diffuse nodular changes at age 10 - No clinicopathological improvement with medical therapy - At age 14 years, total parenteral nutrition was initiated - Complete clinical improvement after starting total parenteral nutrition - Corticotherapy was discontinued 2 months after starting total parenteral nutrition |
| Ref(26) | 56 | Male | Yes | Yes (nausea and vomiting) | Not mentioned | Not mentioned | Not mentioned | Loss of appetite; weight loss (symptoms for 6-7 months) | None mentioned (previously healthy man) | Not mentioned | Not mentioned | Not mentioned | Endoscopic findings described as "esophagitis, gastritis, and duodenitis" | Not mentioned | Present (specific thickness not measured; described as deposition of eosinophilic material resembling collagen within the superficial lamina propria) | Not mentioned | - Patchy lamina propria inflammation composed of plasma cells, lymphocytes, and eosinophils - Patchy surface epithelium lymphocytosis - Deposition of eosinophilic material resembling collagen within the superficial lamina propria - Subsurface deposition highlighted with Masson trichrome staining - Features typical of collagenous gastritis | Not mentioned | Not mentioned | Not mentioned | Not mentioned |
| Ref(27) | 73 | Female | Yes (diffuse spasmodic abdominal pains) | Not mentioned | Yes (chronic diarrhea for 4 months; 4-5 urgent daily stools, diurnal, liquid and sometimes pasty, without mucus or blood) | Not mentioned | Not mentioned | Severe general condition deterioration with major asthenia; anorexia; weight loss of 12 kg; no extra-digestive signs | - Yes (Hashimoto's thyroiditis requiring substitutive therapy with Levothyroxine since 1982) - Autoimmune atrophic gastritis (Biermer's disease - probable) - Anti-parietal cell antibodies: Positive (on retrospective testing) - Anti-intrinsic factor antibodies: Positive (on retrospective testing) | No | Yes (erythematous non-ulcerated antritis) | No | Gastroscopy, colonoscopy with terminal ileoscopy, and barium transit of small intestine were normal except for erythematous non-ulcerated antritis | Negative (histological search for *H. pylori* was negative) | - Initial biopsy (at diagnosis):   - Stomach (antrum): 50 μm (maximum)  - Stomach (fundus): 20 μm (maximum)   - Follow-up biopsy (after 4 months of budesonide treatment):   Stomach: Normalization of basement membrane with disappearance of collagenous thickening | - Initial biopsy (at diagnosis):   Colon: 20-58 μm (depending on biopsy site)   - Follow-up biopsy (after 4 months of budesonide treatment):   Rectocolon: Decrease in collagenous thickening but still abnormally thick | - Gastric (antrum and fundus):   - Widely detached epithelium with decreased mucus secretion  - Moderate lymphoplasmacytic inflammatory infiltrate of the chorion  - Collagenous thickening of subepithelial basement membrane (predominant in antrum: 50 μm; fundus: 20 μm)  - Discrete elevation of intraepithelial lymphocytosis: 28 lymphocytes per 100 epithelial cells  - At fundus: Glandular atrophy with disappearance of parietal cells, antro-pyloric metaplasia, and early intestinal metaplasia   - Colonic (staged biopsies):   - Mucosal atrophy with detachment of surface epithelium  - Moderate lymphoplasmacytic infiltrate of the chorion  - Intraepithelial lymphocytosis: 10 lymphocytes per 100 epithelial cells  - Collagenous thickening of subepithelial basement membrane: 20-58 μm   - Follow-up biopsies (after 4 months of budesonide):   - Gastric (fundus): Normalization of basement membrane with disappearance of collagenous thickening; decreased interstitial inflammatory infiltrate; severe glandular atrophy with intestinal metaplasia; micronodular neuroendocrine hyperplasia (confirmed by chromogranin A immunostaining - also present retrospectively on initial biopsies)  - Rectocolonic: Decreased density of chorion inflammatory infiltrate; increased glandular mucus secretion; decreased collagenous thickening of basement membrane (still abnormally thick) | - Initial attempts: Fenofibrate discontinuation (no improvement); 5-ASA derivatives (ineffective); cholestyramine (ineffective); transit slowing agents (ineffective) - Successful treatment: Budesonide   - Initial dose: 9 mg daily  - After 4 months: 3 mg daily  - Subsequently: Weaning off budesonide without recurrence of clinical signs   - Vitamin B12 supplementation (parenteral route) | - Clinical follow-up: At least 4 months - Histological control performed after 4 months of budesonide treatment | After 4 months of budesonide treatment:   - Stomach: Complete disappearance of collagenous thickening; normalization of basement membrane - Rectocolon: Decrease in collagenous thickening but still abnormally thick - Revealed severe glandular atrophy with intestinal metaplasia and micronodular neuroendocrine hyperplasia at fundus (previously underestimated on initial biopsies) | - Improvement noted after 15 days of budesonide treatment - After 4 months on 3 mg budesonide:   - Transit normalized with tendency to constipation  - Abdominal pains disappeared  - General condition markedly improved with return of appetite  - Decreased asthenia  - Weight gain of 5 kg   - Biological improvements:   - No recurrence of hypokalemia  - Vitamin B12 levels normalized after parenteral supplementation  - Initially normal gastrin level became markedly elevated (727 ng/L; normal upper limit: 120 ng/L) on control testing   - Successful weaning off budesonide without recurrence of clinical signs - Laboratory findings:   - Initial: Hypokalemia (3 mmol/L); minimal hypoalbuminemia (37 g/L) with hypoproteinemia (62 g/L); discrete vitamin B12 deficiency (197 pg/mL; normal >211 pg/mL); normal TSH, 5-HIAA urinary, serotonin, gastrin; negative anti-endomysium IgA, anti-gliadin IgA and IgG antibodies, antinuclear antibodies; normal immunoglobulin levels; negative HIV serology; normal d-xylose test; negative fecal α1-antitrypsin clearance; negative 24-hour proteinuria; normal average daily stool weight with normal fat and protein excretion (with reservation due to collection difficulties)  - Follow-up: Positive anti-parietal cell antibodies and anti-intrinsic factor antibodies; markedly elevated gastrin (727 ng/L); normal vitamin B12 |
| Ref(28) | 25 years (presented at age 25 in 2011; symptoms started at age 20 in 2005; first EGD at local doctor was in July 2008 when he was approximately 23 years old) | Male | Yes (epigastralgia/epigastric pain since 2005) | Yes (nausea since 2005) | No | No (hemoglobin within normal limits) | Not mentioned | Abdominal distention; feeling of fullness (3-year history) | None mentioned; no past medical history | Yes (diffuse mucosal nodularity in the body of the stomach; nodularity with cobblestone appearance; pseudopolyposis-like findings) | Not mentioned | Not mentioned | Extensively discolored depression from upper gastric body to lower gastric body, mainly including greater curvature; residual mucosa with multiple islands; pseudopolyposis-like appearance | Negative (urea breath test, gastric biopsy specimens, and serum anti-*H. pylori* antibody all negative) | Present (membranous collagen band in superficial layer area of gastric mucosa confirmed by Elastica-Masson's stain; specific thickness not measured but described as >10 μm based on diagnostic criteria mentioned; remarkable collagen band in depression lesions; no collagen band in nodular lesions) | Colonoscopy normal; no pathological findings in random colonic biopsies; no collagen band | - Depression lesions:   - Moderate infiltration of inflammatory cells in lamina propria of mucous membrane  - Membranous, weakly eosinophilic and fibrous tissue in superficial layer area of gastric mucosa  - Light green-philic collagen band confirmed by Elastica-Masson's stain   - Nodular lesions:   - Only mild infiltration of inflammatory cells  - No remarkable collagen band   - Initial biopsies (at local doctor): Chronic gastritis; atrophic mucosa with diffuse inflammatory cell infiltration; no malignant findings - Colon biopsies: Normal | - Initially: PPI (rabeprazole 10 mg) given by local doctor - At Tohoku University Hospital: Changed to preprandial administration of rabeprazole 10 mg morning and evening (20 mg/day total) - After 40 days: Decreased to 10 mg once daily before breakfast - Also: Improvement of lifestyle | - 3-year follow-up by local doctor (2008-2011): Underwent EGD three times; gastric findings unchanged in both endoscopy and histology - At Tohoku University Hospital: At least 40 days follow-up mentioned | - During 3-year follow-up at local doctor: No changes in histology - No improvement in endoscopic images or pathological findings reported | - Initially: No obvious improvement in symptoms during 3-year follow-up at local doctor - After treatment at Tohoku University Hospital: Symptoms gradually became stable - After 40 days: Symptoms did not worsen after dose reduction - Near disappearance of symptoms achieved with preprandial administration of rabeprazole and improvement of lifestyle - Laboratory findings:   - Normal: Hemoglobin, mean corpuscular volume, inflammatory response, serum iron, liver function, kidney function  - Elevated: IgE (236 IU/mL; normal <170 IU/mL); serum gastrin (510 pg/mL; normal 37-172 pg/mL - elevation seems to be due to effect of oral PPIs)  *- H. pylori*: Negative   - Colonoscopy: Normal - Abdominal CT and ultrasonography: Normal |
| Ref(29) | 18-19 years (presented at age 18; was 19 years old at time of case report publication) | Male | Yes (chronic intermittent abdominal pain for about 6 months) | Not mentioned | No | Not mentioned | Not mentioned | Constipation (chronic intermittent constipation for about 6 months) | - None (autoimmune evaluation including myeloperoxidase antibody, proteinase 3 antibody, parietal cell antibody, ANCA was negative) - Celiac, thyroid, and pancreatic enzyme studies were normal | Yes (nodular appearing gastric mucosa prominent in gastric body and antrum; nodules were well demarcated) | Not mentioned for stomach | Not mentioned for stomach | Peptic duodenitis noted | Negative (CLO test was negative) | - Initial biopsy: Approximately 15 μm (thickened collagen mucosal band) - Follow-up biopsy (after 6 months): Up to 25 μm (increased from previously 15 μm - showing progression) | Colonoscopy and biopsy were normal (no collagen band) | - Initial biopsy:   - H&E stain: Surface disruption of inflamed gastric mucosa with intraepithelial lymphocytosis; lamina propria contains mixture of plasma cells, lymphocytes, and eosinophils; capillary trapping and vascular dilatation  - Trichrome stain: Irregular thickening of subepithelial band; increased collagen deposition around dilated capillaries; lamina propria contains lacelike pattern of increased collagen; collagen deposition measures 15 μm in most affected area   - Follow-up biopsy (after 6 months):   - H&E stain: Flattening of surface foveolar epithelium with increased intraepithelial lymphocytes; lymphoplasmacytic expansion of lamina propria  - Trichrome stain: Prominent subepithelial basement membrane thickening in well-organized pattern with increased collagen deposition measuring up to 25 μm (previously 15 μm) - showing histological progression   - Colon biopsy: Normal | - Initially: Miralax and increased fiber diet (for functional constipation) - After diagnosis: PPIs for 3 months - Diet therapy and daily Miralax (for constipation) | - 6 months follow-up with repeat endoscopy - Capsule endoscopy performed (no abnormality in small bowel) | After 6 months:   - Progression of collagenous gastritis - Greater inflammatory infiltrate - Increase in collagen band thickness (from 15 μm to 25 μm) - Persistent finding of nodular gastric mucosa on repeat endoscopy | - Initially: Thought to have functional constipation - After diagnosis and treatment:   - Constipation improved on diet therapy and daily Miralax  - Abdominal pain decreased in severity   - Laboratory findings:   - Basic serological studies completely normal  - Liver function tests unremarkable  - Celiac, thyroid, and pancreatic enzyme studies normal  - Autoimmune evaluation negative  - Gastric aspirate showed acidic pH of 2.0   - Imaging studies:   - Abdomen X-ray: Normal  - Ultrasound: Normal   - Endoscopic findings over time:   - Persistent nodular gastric mucosa after 6 months  - Capsule endoscopy: No small bowel abnormality |
| Ref(30) | 25 years old (when first referred in October 2008; 29 years old at follow-up in October 2012) | Female | Yes (5-year history of epigastralgia since approximately age 20) | Not mentioned | No | Yes (moderate iron-deficiency anemia initially; resolved at follow-up) | Not mentioned | - Tarry stools (in May 2010) - History of gastric ulcer bleeding at ages 17 and 19 | None mentioned | - Initial examination (October 2008):   - Nodularity: Yes (diffuse mucosal nodular pattern in gastric body; resembling nodular gastritis)  - Erythema: Not mentioned  - Erosion: Not mentioned  - Others: Nodular pattern NOT observed in antral mucosa   - Follow-up examination (October 2012):   - Nodularity: Yes (pseudopolyposis or cobblestone-like mucosal nodularity in gastric body)  - Erythema: Not mentioned  - Erosion: Not mentioned  - Others: Antrum appeared normal; no lesions related to bleeding (no ulcer or cancer found)   - May 2010 (during tarry stools episode):   EGD, colonoscopy, wireless capsule endoscopy did not show source of bleeding | | | | - Negative (no *H. pylori* on HE stain, Giemsa stain, or immunohistochemical staining) - Serum anti-*H. pylori* IgG antibody negative (3.0 U/ml) - Ruled out *H. pylori* infection | - Initial biopsy (October 2008):   Stomach: Patchy, band-like subepithelial collagen of less than 10 μm (some specimens showed deposition)   - Follow-up biopsy (October 2012):   - Stomach (depressed lesion between nodules): Greater than 10 μm (specifically measured at 16.5 μm in some areas)  - Stomach (nodular lesion): No remarkable subepithelial collagen band   - Colon: Colonoscopy showed normal mucosal appearance; mucosal biopsies not undertaken | | - Initial biopsy (October 2008):   - HE sections from gastric body: Mild atrophy and chronic active inflammation  - Some specimens: Patchy, band-like subepithelial collagen deposition (<10 μm) highlighted with Masson trichrome stain  - No *H. pylori* on multiple staining methods  - Uncertain whether biopsies from nodular lesions or mucosa between nodules   - Follow-up biopsy (October 2012):   - Depressed lesion between nodules:   1. HE stain: Focal superficial epithelial degeneration; numerous intraepithelial lymphocytes; dense lymphoplasmacytic infiltrates lacking lymphoid follicles with germinal centers in lamina propria; no intestinal metaplasia 2. MT stain: Thickened subepithelial collagen band >10 μm (measured 16.5 μm) that did not extend to deeper portions of lamina propria 3. Some dilated capillaries containing red blood cells entrapped in subepithelial collagen band 4. All specimens from depressed lesion showed thickened collagen band   - Nodular lesion:   1. Almost normal findings with only mild infiltration of inflammatory cells 2. No remarkable subepithelial collagen band   - Comparison with 2008: No clear improvement found   - Colonoscopy (May 2010): Normal mucosal appearance (biopsies not taken) | - Since 2003: Almost continuously treated with oral proton-pump inhibitors - Refused: Regular nonsteroidal anti-inflammatory drug treatment - May 2010 onward: Oral famotidine and omeprazole supplemented with iron - Overall result: Treatment with oral proton-pump inhibitors and histamine-2-receptor antagonists proved ineffective for collagenous gastritis | - 4-year follow-up from October 2008 to October 2012 - May 2010: Admission for tarry stools and severe anemia - October 2012: Follow-up endoscopic examination | After 4 years (October 2008 to October 2012):   - Collagen band thickness increased (from <10 μm to >10 μm, specifically 16.5 μm) - No clear improvement in pathological findings - Persistent subepithelial collagen band with no improvement - Similar endoscopic findings (pseudopolyposis/cobblestone appearance) | - Initial presentation (October 2008):   - Moderate iron-deficiency anemia:   1. Hb: 8.4 g/dl (normal 11.5-15.5 g/dl) 2. MCV: 65 fl (normal 83-100 fl) 3. Serum ferritin: 5 ng/ml (normal 8-180 ng/ml)   - 5-year history of epigastralgia  - History of gastric ulcer bleeding at ages 17 and 19  - Diagnosis: Initially thought to be nodular gastritis   - May 2010 (during follow-up):   - Admitted for tarry stools and severe anemia  - EGD, colonoscopy, wireless capsule endoscopy: No source of bleeding identified  - Treated with oral famotidine and omeprazole supplemented with iron   - October 2012 (4-year follow-up):   - No obvious symptoms  - No abnormalities on physical examination  - Anemia resolved:   1. Hb: 14.2 g/dl 2. MCV: 93.2 fl   - Endoscopy: Similar findings to 2008 (pseudopolyposis/cobblestone appearance)  - Final diagnosis: Collagenous gastritis confirmed   - Laboratory findings:   - Serum pepsinogen I: 54.7 U/ml  - Serum pepsinogen II: 12.3 U/ml  - Pepsinogen I/II ratio: 4.4  - These results ruled out gastric atrophy   - Treatment response:   - Anemia improved with iron supplementation  - Symptoms resolved  - However, histological findings worsened (increased collagen band thickness)  - PPIs and H2-receptor antagonist treatment ineffective for collagenous gastritis itself |
| Ref(31) | 22 years old (first diagnosed with Dieulafoy ulcer in February 2006 at age approximately 16-17; current presentation in January 2011 at age 22) | Female | Yes (stomach ache in January 2011) | No | No (specifically stated: "had no symptoms of diarrhea or malabsorption") | Yes (hemoglobin 8.6 g/dl; iron-deficiency anemia) | Not mentioned | - Hematemesis and melena (the day after stomach ache) - Repeated upper gastrointestinal bleeding from Dieulafoy lesion of the fornix - No history of alcohol ingestion, smoking, aspirin or other anti-inflammatory drugs | - None mentioned - Serum IgG, IgM, and IgE levels were normal | Yes (innumerable fine fading nodules in antrum; fine nodules with smooth peninsula-shaped normal areas in body) | Not mentioned | No | Dieulafoy ulcer in fornix (normal mucosa around ulcer); duodenal bulbs with scarred areas and villous atrophy; magnifying endoscopy showed loss of normal capillary network and pit pattern | Negative (serum IgG antibody negative; culture negative) | 60-100 μm (antrum and corpus nodular areas); normal peninsula areas: no collagen band | Up to 10 μm (no inflammatory cells; NOT collagenous colitis) | - Stomach: Collagen bands (60-100 μm) in lamina propria under mucoepithelial lesion; gland atrophy; eosinophils, lymphocytes, plasma cells (medium-grade inflammation); peninsula areas normal - Duodenum: Villous atrophy in bulb with collagen bands up to 60 μm; distal duodenum normal. Colon: Thin collagen band ≤10 μm, no inflammation | February 2006: PPI (2 weeks), H2RA (6 months). January 2011: Omeprazole 20 mg/day, iron supplements; changed to famotidine 40 mg/day after 2 months | Repeat gastroduodenoscopies at 1 month and 1 year after January 2011 bleeding; colonoscopy after discharge | No interval changes at 1 month and 1 year | February 2006: Dieulafoy ulcer diagnosed, bleeding stopped, nodular gastric findings noted. January 2011: Recurrent Dieulafoy ulcer with hematemesis/melena. Final diagnosis: Collagenous gastroduodenitis (CGD). Symptoms resolved with treatment but no endoscopic/pathological improvement |
| Ref(32) | 47 | Female | Yes (epigastric pain - two-month history; dull ache in nature, worsened by cold drinks) | No (denied nausea and vomiting) | No (denied diarrhea) | Yes (Hb 101 g/L; normal range 115-160 g/L; mild anemia) | No | Reflux symptoms; denied GI hemorrhage, fever; normal appetite | - Yes - Multiple autoimmune diseases:   - Systemic lupus erythematosus (SLE) - diagnosed 7 years ago  - Autoimmune hemolytic anemia - diagnosed 7 years ago  - Hypothyroidism   - First reported case of collagenous gastritis associated with SLE | No (endoscopy was normal) | No (endoscopy was normal) | No (endoscopy was normal) | - Esophagogastroduodenoscopy: Normal - Colonoscopy: Normal (including random colonic biopsies) | Positive | Approximately 70 μm thick (subepithelial collagenous layer - homogenous, lightly eosinophilic on H&E, stained with trichrome) | Normal (random colonic biopsies were normal; NO colonic involvement) | - Gastric biopsies: - - Thickened subepithelial layer with associated gastritis - - Subepithelial collagenous layer: homogenous, lightly eosinophilic on H&E, stained with trichrome - - Collagen layer approximately 70 μm thick - - Few entrapped fibroblasts, inflammatory cells and blood vessels in collagenous layer - - Inflammatory infiltrate: predominantly lymphoplasmacytic with few neutrophils and eosinophils - - Focal active inflammation: cryptitis and reactive glands present - Colonic biopsies: Normal | - PPI - *H. pylori* eradication therapy (10 days) - Medications for underlying conditions: Chloroquine and low-dose corticosteroids (for SLE and autoimmune hemolytic anemia) | Three months of follow-up | Three months of follow-up | - Presentation:   - Southeast Asian ethnicity  - Two-month history of epigastric pain (dull ache, worsened by cold drinks, associated with reflux)  - Denied nausea, vomiting, diarrhea, GI hemorrhage, fever  - Normal appetite   - Physical exam:   Mild epigastric tenderness   - Laboratory findings:   - Hemoglobin: 101 g/L (normal 115-160)  - MCV: 96.7 (normal 79-97)  - Albumin, vitamin B12, folate: Normal   - Past medical history:   - SLE (diagnosed 7 years ago)  - Autoimmune hemolytic anemia (diagnosed 7 years ago)  - Hypothyroidism  - Maintained on chloroquine and low-dose corticosteroids   - Endoscopy:   - Colonoscopy: Normal (including random biopsies)  - EGD: Normal endoscopically  - Gastric biopsies: Showed collagenous gastritis   - *H. pylori*: Positive - Treatment response:   - Started on PPI and 10-day *H. pylori* eradication  - Symptoms improved over next 3 months of follow-up   - Final diagnosis: Collagenous gastritis without colonic involvement in patient with SLE |
| Ref(33) | 12 years old at presentation | Female | Not explicitly mentioned | Hematemesis (recurrent episodes - initial episode and one 5 months later) | No diarrhea reported | Mild - hemoglobin 12.2 g/dL, hematocrit 38.3%, MCV 82.7 fL | Not mentioned | Emaciation (BMI 14.6, -2.4SD); recurrent gastric ulcers | - None identified - Anti-parietal cell antibody: negative - Serum gastrin: 499 pg/mL (not significantly elevated) - Autoimmune atrophic gastritis excluded | Many lotus leaf-like lesions on gastric mucosa; cobblestone appearance in duodenal bulb; nodular gastric mucosa | Not mentioned | Not mentioned | Atrophic gastric mucosa in the antrum; scarred duodenal ulcer in duodenal bulb; recurrent gastric ulcers during follow-up; decreased normal gastric mucosa area dividing into smaller nodules over time | - Negative on endoscopy - Negative on urea breath test (UBT) - Negative on serum *H. pylori* IgG antibody test - Negative on stool *H. pylori* antigen test - Initially treated for *H. pylori* eradication despite negative tests due to suspicious endoscopic findings | 16 μm (at endoscopy 4) | Not applicable - colonoscopy at age 19 showed no abnormalities; no collagenous colitis | - Subepithelial collagen deposition >10 μm - Inflammatory cell infiltration in lamina propria - Thin collagen band initially at edge of lotus leaf-like gastric mucosa - Collagen band found in atrophic areas, not in nodular mucosa - Both gastric (antrum) and duodenal (duodenal bulb) mucosa showed collagen deposition - Duodenal collagen deposition more prominent than gastric | - H2-blocker (famotidine) treatment over 6 years - Treatment administered in years 1, 3, and 5 - No treatment in years 2 and 4 | - 6 years of endoscopic follow-up (annual endoscopies) - At age 18 (6th year): weight increased to 45 kg, height 160 cm (BMI 18.0) - improved from initial BMI 14.6 - Capsule endoscopy at age 18: no abnormalities in small intestine - Colonoscopy at age 19: no abnormalities - Patient asymptomatic after continuous famotidine treatment | - More prominent subepithelial collagen deposition over time - Endoscopic and pathological findings unchanged after 1 year of famotidine treatment - No improvement in structural disorder despite symptom relief | - Recurrent gastric ulcers when famotidine treatment interrupted (active gastric ulcer in year 2, healing stage gastric ulcer in year 4) - No gastric ulcers when on famotidine treatment (years 1, 3, 5, and continuous treatment from year 6) - Symptoms controlled with H2-blocker therapy despite persistent histological changes - Weight gain and clinical improvement with continuous treatment - Disease remained stable without spreading to other parts of GI tract |
| Ref(34) | - Children (n=10): Range 3-18 years (mean 11.6 years, median 12 years) - Adults (n=21): Range 24-81 years (mean 57.5 years, median 56 years) | - Children: 5 males (50%), 5 females (50%) - Adults: 5 males (24%), 16 females (76%) - Total: 10 males (32%), 21 females (68%) | Children 5/10 (50%), Adults 9/20 (45%) | Children 2/10 (20%), Adults 5/20 (25%) | Children 4/10 (40%), Adults 11/20 (55%) | Children 5/10 (50%), Adults 7/20 (35%) | Not mentioned | Weight loss - Children 2/10 (20%), Adults 4/20 (20%); Heme-positive stools - Children 2/10 (20%), Adults 3/20 (15%); Dysphagia - Adults 1/20 (5%) | - Children: Common variable immunodeficiency (n=1), Celiac disease (n=1) - total 2/10 (20%) - Adults: Autoimmune metaplastic atrophic gastritis (n=2), SLE (n=1) - total 3/21 (14%) - Overall: 5/31 (16%) had immunodeficiency or autoimmune conditions - Collagenous sprue history (n=1), Collagenous colitis history (n=4) | Children 6/8 (75%), Adults 8/19 (42%); Total 14/27 (52%) - often diffuse distribution | Children 0/8, Adults 6/19 (32%) | Not specifically mentioned | Atrophic mucosa - Adults 4/19 (21%); Unremarkable stomach - Children 2/8 (25%), Adults 1/19 (5%) | - All negative on hematoxylin and eosin examination - Special stains performed in 23/31 (74%) specimens - all negative - Prior *H. pylori* treatment history in 3 adults | Range 12-233 μm (mean 78 μm); Children mean 77 μm (range 13-184), Adults mean 79 μm (range 12-233) | Collagenous colitis found in 10/14 (71%) cases with colon biopsies; Children 4/7 (57%), Adults 6/7 (86%) | - Sloughed/attenuated surface foveolar epithelium - Thickened subepithelial collagen table (>10 μm) - Patchy collagen distribution with ragged interface - Lamina propria inflammatory infiltrate (lymphocytes, plasma cells, eosinophils) - Eosinophils: >30 per high-power field in 11/31 (35%) cases; Children 5/10 (50%), Adults 6/21 (29%) - Surface intraepithelial lymphocytosis: 8-36 per 100 epithelial cells in all cases; >25 per 100 in 2/31 (6%) cases - Focal-mild neutrophilic inflammation in 16/31 (52%) - Location: Both corpus and antrum (70%), corpus only (21%), antrum only (9%) - Intestinal metaplasia: 2/31 (6%) adults - Pseudopyloric gland metaplasia with enterochromaffin-like cell hyperplasia: 2 adults (autoimmune metaplastic atrophic gastritis) - Associated collagenous enteritis: 9/25 (36%) - Children 2/8 (25%), Adults 7/17 (41%) - Associated collagenous colitis: 10/14 (71%) | - Children (n=5 with follow-up): Budesonide (n=2), Sucralfate (n=1), Gluten-free diet (n=1), Immunomodulatory regimen for common variable immunodeficiency (n=1) - Adults (n=17 with follow-up): Systemic steroids/budesonide (n=8), Proton-pump inhibitor (n=6), Loperamide (n=2), 6-mercaptopurine (n=1), No therapy (n=2) - Combined therapies: Steroids + proton-pump inhibitor + gluten-free diet (n=3) - Olmesartan cessation in 2 patients - Iron supplementation: Children (n=3), Adults (n=4) | - Children: 5/10 (50%) had follow-up; Range 5-48 months (mean 21.2 months, median 17 months) - Adults: 17/21 (81%) had follow-up; Range 2-122 months (mean 37.3 months, median 25 months) - Overall: 22/31 (71%) had follow-up data | - Children: Repeat biopsies in 4/5; Persistence of collagenous gastritis in 3/4 (75%); Normal gastric biopsy in common variable immunodeficiency patient (but continued collagenous colitis) - Adults: Repeat biopsies in 10/17 (59%); Persistent collagenous gastritis in 5/10 (50%); Absent collagen table in 5/10 (50%) - replaced by lymphoplasmacytic infiltrate with patchy eosinophils (n=4) or lymphocytic gastritis (n=1) - No dysplasia or malignancy developed in any patient | - Symptomatic relief: 7/22 (32%) overall; Children 1/5, Adults 6/17 - Complete resolution (both symptomatic and histologic): 3/14 (21%) - Persistence of symptoms and/or histologic findings despite treatment in majority - Children: 100% had persistent symptoms or collagenous gastritis on subsequent biopsies - Adults: 82% had persistent symptoms or collagenous gastritis - One adult on olmesartan had complete resolution after medication cessation - No progression to dysplasia or adenocarcinoma during follow-up period |
| Ref(35) | - Total cohort (n=40): Median 16 years, range 3-89 years - Pediatric patients (n=24, 60%): ≤18 years of age - Adult patients (n=16, 40%): >18 years of age | - Female: 26/40 (65%) - Male: 14/40 (35%) - Pediatric: 14 females (58%), 10 males (42%) - Adult: 12 females (75%), 4 males (25%) | Pediatric 13/23 (57%), Adults 4/15 (27%); Total 17/38 (45%) | Pediatric 6/23 (26%), Adults 0/15; Total 6/38 (16%) | Pediatric 3/23 (13%), Adults 4/15 (27%); Total 7/38 (18%) | Pediatric 9/23 (39%), Adults 10/15 (67%); Total 19/38 (50%) | Not mentioned | Gastrointestinal bleed (hematemesis/melena) - Pediatric 5/23 (22%), Adults 1/15 (7%), Total 6/38 (16%); Weight loss - Pediatric 3/23 (13%), Adults 0/15, Total 3/38 (8%); Incidental finding - 1/38 (3%) | - Celiac sprue: 3/40 (8%) - Pediatric 2, Adult 1 (confirmed by histology and serology) - Additional patients with duodenal intraepithelial lymphocytosis (>30 per 100 enterocytes): 4 patients - Pediatric 3, Adult 1 (normal villous architecture, negative or unknown celiac serology) - One pediatric patient: positive Tissue Transglutaminase but normal duodenal histology - Type 1 diabetes mellitus: 1 pediatric patient - Juvenile arthritis: 1 pediatric patient - Graves disease: 1 adult - Rheumatoid arthritis: 1 adult - Collagenous sprue: 5/40 (12%) - Pediatric 1, Adults 4 - Collagenous colitis: 7/40 (18%) - Pediatric 1, Adults 6 - Associated collagenous sprue and/or colitis: More common in adults (7/16, 44%) than pediatric (2/24); P<0.05 - Other conditions: Asthma, eczema (1 pediatric each); liver cirrhosis, epilepsy, coronary artery disease (1 adult each) | 22/29 (76%) - Pediatric 14/18 (78%), Adults 8/11 (73%) | 7/29 (24%) - Pediatric 4/18 (22%), Adults 3/11 (27%) | Erosions/ulcers 3/29 (10%) - Pediatric 3/18 (17%), Adults 0/11 | Normal stomach - 0/29 cases | - Positive: 1/40 (2.5%) - a 45-year-old male - Negative: 39/40 (97.5%) - Confirmed negative by immunohistochemistry (23 patients), Giemsa/Diff-Quick (5 patients), fecal PCR (1 patient) | - Range 15-145 μm (mean 55.1 μm, 95% confidence interval 45-65 μm) - Pediatric: mean 54.6 μm (range 20-120) - Adult: mean 55.9 μm (range 15-145) | Collagenous colitis in 7/40 (18%) - measurement not specified | - Subepithelial collagen deposition >10 μm (required for diagnosis) - Entrapped capillaries: 36/40 (90%) - Surface epithelial sloughing: None 3/40 (8%), Focal (<30% surface) 32/40 (80%), Prominent (>30% surface) 5/40 (12%) - Collagen distribution: Focal (<30% surface) 3/40 (8%), Patchy (30-69% surface) 20/40 (50%), Diffuse (≥70% surface) 17/40 (42.5%) - Location: Corpus predominant 22/40 (55%) - more common in pediatric 17/24 (71%) than adults 5/16 (31%), P<0.05; Antrum predominant 11/40 (28%); Equal involvement 7/40 (18%) - Corpus only involved: 9 cases; Antrum only involved: 2 cases - Chronic (lymphoplasmacytic) inflammation: Mild 18/40 (45%), Moderate 22/40 (55%) - more common in adults (75%) than pediatric (42%), P=0.05 - Active (neutrophilic) inflammation: Present 20/40 (50%) - None 20/40, Focal 6/40 (15%), Mild 13/40 (32%), Moderate 1/40 (2%) - Intraepithelial lymphocytes: Range 5-45 per 100 surface epithelial cells; ≥25 per 100 cells in 5/40 (12%) - Pediatric 2, Adults 3 - Eosinophils: Mean 41/high power field in pediatric, 29/high power field in adults; >30 eosinophils/high power field in 21/40 (52%) - Pediatric 15/24 (62%), Adults 6/16 (38%) - Intestinal metaplasia: 3/40 (8%) - all adults; on initial or follow-up biopsy - Moderate to severe gastric corpus atrophy: 7/40 (18%) - Pediatric 5, Adults 2 (aged 24 and 44); with pseudopyloric metaplasia and smooth muscle hyperplasia; minimal enterochromaffin-like cell hyperplasia - Associated collagenous enteritis: 5/40 (12%) - Associated collagenous colitis: 7/40 (18%) | - Not specifically detailed for individual patients in this article - Article focuses on pathologic features rather than treatment protocols | - Follow-up biopsies available: 23/40 (58%) - Pediatric: 18/24 had follow-up - Adults: 5/16 had follow-up - Median follow-up time: 1.9 years (mean 2.4 years, range 0.02-10 years) - Pediatric: median 1.1 years (range 0.02-10 years) - Adults: median 4 years (range 0.95-6 years) | - Persistent collagen at last biopsy: 18/23 (78%) - Pediatric 15/18 (83%), Adults 3/5 (60%) - No collagen at last biopsy: 5/23 (22%) - Pediatric 3/18 (17%), Adults 2/5 (40%) - Time to no residual collagen: mean 3 years (range 0.8-6 years) after initial presentation - Of 5 with no residual collagen: 4 had >30 eosinophils/high power field initially, 1 was pediatric with moderate gastric atrophy - Collagen persisted up to 10 years from initial biopsy in some patients - One patient with tenascin-positive thin collagen layer on follow-up (10 months) that was not detectable by hematoxylin and eosin or trichrome | - Persistent disease histologically in majority (78%) of patients with follow-up - Symptom resolution documented in at least 1 patient with no residual collagen - Incomplete clinical follow-up data for most patients regarding symptom persistence or resolution - No development of dysplasia or malignancy mentioned - Disease can occasionally resolve fully (5/23 patients with no collagen at final biopsy) - Absence of collagen on follow-up may represent sampling error due to patchy distribution - Long-term outcome remains uncertain - Persistent symptoms in at least one patient (epigastric pain) despite follow-up at 3 years, with thin collagen detected only by tenascin immunohistochemistry |
| Ref(36) | 10.5 years old (10 years and 6 months) at diagnosis | Female | Yes - abdominal pain present since age 4 years | Not mentioned | Yes - chronic diurnal diarrhea (5 liquid stools per day, non-bloody, non-mucoid) since age 4 years | Yes - severe anemia (hemoglobin 62 g/L) with asthenia and exertional dyspnea | Not mentioned | Weight loss/growth impairment - weight growth retardation between ages 6-8 years, followed by height growth retardation; microcytic, hypochromic, hypochromic iron deficiency anemia | - None identified - Celiac disease serology: negative - Antinuclear antibodies (ANA): negative - Anti-neutrophil cytoplasmic antibodies (ANCA): negative - Anti-Saccharomyces cerevisiae antibodies (ASCA): negative - Anti-tissue antibodies: negative - No significant family history - No other autoimmune conditions reported | Yes - micronodular gastritis with inflammatory area predominantly in the antrum | Yes - inflammatory area in the antrum | No ulceration or active bleeding visible | Macroscopically normal duodenal, terminal ileal, and colonic mucosa | - Negative - *H. pylori* testing on biopsies: negative | Subepithelial collagenous band present (specific thickness not mentioned) | Continuous collagen deposit >10 μm thickness located under the surface epithelium (pancolitis) | - Severe atrophic gastritis with fibrosis and notable eosinophilia - Pancollagenous colitis with significant eosinophilia - No epithelioid or tuberculoid granulomas - Subepithelial fibrosis with laminated appearance - Rarefaction of glands - Presence of superficial subepithelial collagenous band - Surface epithelial cell alteration - Chorion inflammation - Intense lymphocytic and eosinophilic intraepithelial infiltrate (>20 per 100 epithelial cells) - At 6-month follow-up endoscopy: chronic atrophic and fibrosing gastritis; pancolitis of collagenous and lymphocytic type with intense eosinophilic infiltrate, more severe than initially - No Cytomegalovirus or Epstein-Barr virus identified - Bacteriological cultures negative (Yersinia, Salmonella, Campylobacter jejuni, Shigella) - Parasite testing negative | - Initial treatment: Proton-pump inhibitor 1 mg/kg/day for 1 month - resulted in cessation of diarrhea and weight gain (+2 kg) - After PPI cessation: Diarrhea recurrence at 2 months, partially relieved by racecadotril - Mesalazine: Failed - Oral budesonide: Started at progressively increasing doses, associated with proton-pump inhibitor 1 mg/kg/day - Budesonide 9 mg/day + proton-pump inhibitor: Effective treatment - Treatment duration: 6 months, then stopped - Iron supplementation: Implied for anemia treatment | - 6-month endoscopic follow-up performed - Treatment stopped after 6 months of budesonide - Rapid recurrence of diarrhea and abdominal pain after treatment cessation - Treatment restarted - Further tapering planned when asymptomatic for 6 months | - At 6 months: Chronic atrophic and fibrosing gastritis persisted - Pancollagenous and lymphocytic colitis with intense eosinophilic infiltrate, more severe than initially - No resolution of histological changes despite treatment | - Symptoms began at age 4 years after a trip to Tunisia (treated with amoxicillin-clavulanic acid for foot wound) - Chronic evolution for 6.5 years before diagnosis - Weight growth retardation between ages 6-8 years, followed by height growth retardation - Initial etiological workup at ages 3 and 7 years before diagnosis were negative (inflammatory bowel disease, celiac disease, food allergy) - After initial proton-pump inhibitor treatment: Diarrhea cessation and weight gain (+2 kg) - Relapse 2 months after proton-pump inhibitor cessation - Response to budesonide: Complete cessation of diarrhea within 2 weeks, improvement in general condition and appetite - At 3 months of budesonide 9 mg/day + proton-pump inhibitor: Normal transit, weight gain of 4 kg, height increase of 2 cm - After treatment cessation at 6 months: Rapid recurrence of diarrhea and abdominal pain - Treatment dependence noted - requires ongoing therapy - Demonstrates importance of vigilance with transit disorders impacting growth kinetics in children |
| Ref(37) | 13 years old at presentation | Male | Yes - generalized abdominal pain (6-month history) | Not mentioned | Yes - frequent loose, nonbloody stools (6-month history) | Not mentioned (complete blood count was normal) | Not mentioned | Weight loss - 5-pound weight loss over 6 months | - No celiac disease (celiac panel normal - serum immunoglobulin A levels, endomysial antibodies, tissue transglutaminase antibodies all normal) - Celiac genetics: Heterozygosity for HLA-DQ8/HLA-DQ2 (maternal history of "gluten sensitivity") - Duodenal histology: Normal villi, no evidence of celiac disease - No other autoimmune conditions reported - No increased intraepithelial lymphocytes on duodenal biopsy | Yes - diffuse nodularity involving the gastric body and fundus | Not mentioned | Not mentioned | Antral sparing; visually normal colonoscopy | - Negative - *H. pylori* antibodies: negative | Thickening of the collagen table present (specific measurement not provided) | Normal histological appearance on colonoscopy (no collagenous colitis) | - Gastric body: Gastritis with thickening of the collagen table - Antrum: Mild chronic gastritis - Duodenum: Normal villi, normal disaccharidases - Colon: Normal histological appearance - Subepithelial collagen deposition confirmed on histology - At 6-month follow-up: Histology showed improvement; continued normal linear growth velocity | - Gluten-free diet - No mention of medications (proton-pump inhibitors, steroids, etc.) | - Symptom abatement at 1 month on gluten-free diet - Complete symptom resolution at 6 weeks - Repeat esophagogastroduodenoscopy at 6 months - Continues to do well 3 years following diagnosis on gluten-free diet | Repeat esophagogastroduodenoscopy at 6 months showed:   - Subjective normalization of the fundus - Unchanged gastric body nodularity - Persistent antral sparing   - Histology showed improvement  - Patchy collagenous involvement while on gluten-free diet (vs initial diffuse thickening) | - Initial presentation: 6-month history of symptoms with 5-pound weight loss - Unrestricted gluten exposure at time of initial endoscopy - After starting gluten-free diet:   - Symptom abatement at 1 month  - Complete resolution at 6 weeks  - Abdominal pain resolved  - Stools normalized  - Resumption of appropriate weight gain  - Continued normal linear growth velocity   1. At 6 months: Clinical improvement with histological improvement 2. At 3 years: Continues to do well on gluten-free diet 3. Only transient diarrhea attributed to known gluten exposure 4. Rapid and sustained symptom resolution on gluten-free diet 5. Suggests possible role of gluten in some individuals with collagenous gastritis, despite absence of celiac disease 6. Case raises question about relationship between gluten and collagenous gastritis |
| Ref(38) | 19 years | Male | Yes | Not mentioned | No (had constipation instead) | Not mentioned | Not mentioned | Constipation | - Hypogammaglobulinemia (decreased IgG, IgM, IgA) - No other autoimmune diseases mentioned | Yes (nodular gastric mucosa, well demarcated, prominent in gastric body and antrum) | Not mentioned | Not mentioned | None mentioned | Not mentioned/Not reported | Not specifically measured in text | Not applicable (patient had gastritis, not colitis) | - Collagenous gastritis - Basal cell apoptosis present - Increased plasma cells in mucosa (atypical for immunodeficiency) | - Fiber supplement - Miralax | - Follow-up biopsy performed (timeframe not specified) - Symptoms improved despite worsening collagen thickness on follow-up biopsy | Worsening thickness of collagen band on follow-up biopsy | - Symptoms improved paradoxically despite histological worsening - No immunoglobulin therapy or budesonide trial mentioned for this patient (unlike the collagenous colitis patients in the series) |
| Ref(39) | - 15 years old at presentation - Follow-up to age 18 years | Male | Yes (acute epigastric pain, presented as acute abdomen) | Not mentioned at presentation | Not mentioned | Yes (iron deficiency anemia - hemoglobin 7.1 mmol/l, MCV 65 fl, serum iron 4 mmol/l, serum ferritin 3 g/l) | Not mentioned | Gastric perforation; Tachycardia (110/min); Pallor | - Suggested autoimmune background (per discussion) - No specific autoimmune diseases mentioned | Yes (nodular aspect of the corpus) | Not mentioned | Not mentioned | Remarkably coarse gastric folds; Pseudo-polyps; Thickened folds; Macroscopically inflamed appearance | Negative (not demonstrable on histopathology) | - Initial: up to 23 μm - Repeated gastroscopies over the years: 22-29 μm | Normal (no collagenous changes; both macroscopically and histologically normal) | - Active chronic inflammation with lympho-plasmacellular infiltration - Thickened sub-epithelial collagenous band (irregularly thickened, not extending around foveolae and glandular tubes) - Entrapped capillaries within collagen layer - Increased intraepithelial inflammatory cells in lamina propria - Ulceration at perforation site - Hypertrophic mucosa with irregularly thickened sub-epithelial membrane - Duodenal mucosa normal | - Surgical: Laparotomy with repair of gastric perforation (margins refreshed) - Medical: Iron supplementation for persistent anemia | - Repeated gastroscopies over the years (from age 15 to 18) - Colonoscopy performed (normal results) - At age 18: asymptomatic | - Persistent findings similar to initial presentation - Collagen bands remained elevated (22-29 μm) on repeated gastroscopies | - Presented with acute abdomen due to spontaneous gastric perforation - Required surgical intervention - Persistent anemia requiring frequent courses of iron replacement therapy - Became asymptomatic by age 18 despite persistent histological abnormalities - Continued need for iron supplementation |
| Ref(40) | - 11 years old at presentation - Follow-up at 6 and 18 months | Male | No | Nausea (sensation of nausea) present; No vomiting | Not mentioned | Mild (hemoglobin 13.3 g/dl, ferritin 15 μg/l) | Yes (postprandial fullness/plenitud posprandial) | - No heartburn - Normal bowel habits - Normal development - 3-year symptom duration | Strong family history of autoimmune diseases:   - Mother: Non-specific IBD, chronic gastritis, arthritis - Father: Psoriatic arthritis - Maternal relatives: Chronic gastritis, pernicious anemia, Sjögren's syndrome, celiac disease - Patient history: Recurrent orchiepididymitis (testicular and epididymal biopsy normal); Retention polyp at age 3 - Suspected autoimmune origin for atrophic pattern (though not demonstrated in this patient) - Autoantibodies all negative (ANA, ANCA, ASCA, anti-transglutaminase IgA, anti-parietal cells, anti-LKM, anti-smooth muscle, anti-mitochondrial, anti-intrinsic factor) | Yes (nodular aspect - typical finding, more frequent in children) | Not mentioned | Not mentioned | Pangastric involvement with thickened folds throughout stomach | Negative (not observed) | Greater than 10 μm (thick subepithelial collagen band - "gruesa banda colágena subepitelial") | Normal (colonoscopy ruled out colonic involvement) | - Thick subepithelial collagen band (>10 μm) - Chronic inflammatory cell infiltrate - Marked atrophy of oxyntic glands (marked atrophic pattern - very rare in children) - No *H. pylori* - No intestinal metaplasia - No epithelial dysplasia - Atrophic histological pattern (one of 3 patterns: eosinophilic, lymphocytic, atrophic) | - Vitamin B12 supplementation - Iron supplementation - PPIs and H2 antagonists contraindicated due to pangastric atrophy associated with hypochlorhydria - No corticosteroids used - Symptomatic management only | - Follow-up endoscopies at 6 and 18 months - Patient remained asymptomatic | - Persistence of collagenous gastritis on follow-up endoscopies at 6 and 18 months - No changes mentioned | - Chronic but benign course - Patient became asymptomatic with conservative treatment (B12 and iron) - No symptom recurrence during follow-up period - Histological findings persisted despite clinical improvement |
| Ref(41) | - 21 years old at presentation to Mayo Clinic - 14 years old when symptoms began (abdominal pain and anemia) - 20 years old at first EGD | Female | Yes (intermittent abdominal pain starting at age 14, severe cramping, disabling abdominal pain worsened after food ingestion) | Not mentioned | Yes (severe watery diarrhea shortly after birth, postprandial diarrhea) | Yes (refractory iron-deficiency anemia) | Not mentioned | Poor feeding tolerance, low-grade fevers, acid reflux, persistent hiccups, heartburn | None (autoimmune panel was unremarkable) | Yes (gastric body nodularity at first EGD, resolved after *H. pylori* eradication) | Yes (erythema in gastric body at first EGD, blotchy areas of erythema at second EGD) | Not mentioned | Diffuse severe inflammation with edema in gastric body, diffuse gastric atrophy on second EGD, normal esophagus and duodenum | - Positive at first EGD - Successfully eradicated with triple therapy (clarithromycin, amoxicillin, omeprazole) - Negative on immunostaining at second EGD | 200 μm | No evidence of collagenous colitis (colonoscopy with random biopsies showed no collagenous colitis) | - Chronic gastritis with subepithelial collagen thickening (confirmed with Masson trichrome stain) - Scattered infiltrate of plasma cells and eosinophils - Normal duodenal mucosa without villous atrophy - Collagen type I, II, and III mentioned as predominant | - Triple therapy for *H. pylori* eradication (clarithromycin, amoxicillin, omeprazole) - Oral and intravenous iron supplements - Oral budesonide delayed-release capsules 3 mg twice daily (capsules opened and granules mixed with maple syrup) - Dietary restrictions (avoided gluten, lactose, and fruits - no significant improvement) | - Second EGD at age 21 (one year after *H. pylori* eradication) - Six months after starting budesonide therapy | - Subepithelial collagen band persisted (200 μm) - *H. pylori* negative on immunostaining• Chronic gastritis with collagen deposition remained | - Prior nodularity resolved after *H. pylori* eradication - Severe cramping persisted after *H. pylori* eradication - Six months after budesonide: moderate improvement of abdominal pain, anemia resolution, persistent heartburn - Classified as pediatric type collagenous gastritis (associated with abdominal pain, anemia, colon sparing) |
| Ref(42) | 26 | Female | Yes (intermittent abdominal pain for 6 months, mild epigastric tenderness) | Not mentioned as presenting symptoms (mentioned in discussion as possible symptoms) | Yes (irregular bowel habits with tendency for diarrhea, two loose bowel movements daily initially, three loose bowel movements per day at follow-up) | No (CBC was normal) | Not mentioned | Weight loss (20 pounds over 6 months), heartburn/gastroesophageal reflux disease | - Scleroderma - IgA deficiency (low total IgA level) | Not mentioned in this specific case | Not mentioned in this specific case | Not mentioned | Gastritis and duodenitis on EGD, normal colonoscopy | Not mentioned | Significant thickening of sub-epithelial collagen (specific measurement not provided) | Normal colonoscopy, no colonic involvement | - Gastric biopsy: Significant thickening of sub-epithelial collagen and chronic inflammation consistent with collagenous gastritis - Duodenal biopsy: Preservation of villous architecture with mildly increased intraepithelial lymphocytic inflammation consistent with focal mild lymphocytic duodenitis - Mild increase in lymphocyte count on CBC | - Omeprazole 20 mg (for heartburn, started 6 months before diagnosis) - Gluten-free diet (started 3 months before diagnosis, with partial improvement) | 3 months after diagnosis | Not mentioned (no repeat biopsy reported) | - Partial improvement after 3 months of treatment with omeprazole and gluten-free diet - Abdominal pain resolved - Persistent diarrhea (three loose bowel movements per day) - Classified as adult type with isolated gastric involvement (no colonic involvement, which is rare) |
| Ref(43) | - Range: 5-18 years - Mean age at presentation: 11.5 years - Individual ages: 14F, 15F, 12F, 9M, 18F, 11M, 13M, 10F, 5F, 10F, 9M, 13M | - Female: 7 patients (58%) - Male: 5 patients (42%) | 6 patients (50%) | 1 patient (recurrent vomiting) | 3 patients (25%) - all had adult-onset phenotype with collagenous colitis | 11 patients (92%) - iron deficiency anemia | Not mentioned | Upper gastrointestinal bleeding/hematemesis (1 patient), retrosternal pain (1 patient) | - Type 1 diabetes mellitus: 1 patient (5-year-old girl) - Achalasia and psoriasis: 1 patient (11-year-old boy) - Celiac disease: 1 patient (14-year-old girl) - Autoimmune thyroid disease: 1 patient (developed as adult) - No autoimmune disorder: 1 patient with adult-type disease (13-year-old) - Adult-onset phenotype (with collagenous colitis): 3 patients (25%) | Yes - nodularity of gastric mucosa (most characteristic finding), especially gastric body | Yes - mucosal erythema | Yes - erosive gastritis, erosions mentioned | Mucosal edema, hyperplastic rugae, pronounced folds, polypoid lesions, ulceration, patchy irregular mucosa, linear whitish infiltration, prominent rugae, hyperplastic rugal folds | Not mentioned in any of the 12 cases | Thicker than 10 μm (diagnostic criterion); specific measurements not provided for individual cases | 3 patients (25%) had collagenous colitis with subepithelial collagen deposition | - Subepithelial collagen band deposition (>10 μm) in lamina propria - Chronic inflammatory infiltrate consisting of lymphocytes, plasma cells, and eosinophils - Most common location: gastric body - Also involved: antrum (in several cases), colon (in 3 cases) - Normal duodenum in most cases (except celiac disease patient and one patient who developed duodenal involvement on follow-up) - Patches or thick layers of hyaline material beneath surface epithelium - Increased inflammatory cells in lamina propria | - Oral iron supplementation: 11 patients (92%) - PPIs: 4 patients - Gluten-free diet: 2 patients - Gluten and dairy-free diet: 1 patient - 4-food elimination diet (cow's milk, soy, wheat/gluten, egg): 1 patient - Oral prednisolone followed by azathioprine: 1 patient (11-year-old boy with adult-type disease) | - 9 of 12 patients (75%) had documented follow-up - Median duration: 30 months (range: 1-78 months) - Repeat endoscopic examinations with biopsy: 8 patients (67%) | - 7 of 8 patients: histological findings either unchanged or progressed - 1 patient (11-year-old boy treated with corticosteroids and azathioprine): marked reduction of subepithelial collagen deposition, but developed additional duodenal involvement - 1 patient with celiac disease: worsening of collagenous gastritis despite improvement in celiac disease on gluten-free diet - Several patients showed increased subepithelial collagen or increased inflammatory activity - No histological improvement with iron supplementation, PPIs, or elimination diets | - Iron deficiency anemia resolved in all 9 followed patients on oral iron supplementation, but required ongoing therapy - No clinical improvement with elimination diets or PPI treatment - 1 patient (treated with corticosteroids and azathioprine): clinical and histological improvement - Some patients developed further involvement of antrum and/or duodenum over time - No patients with pediatric phenotype progressed to adult phenotype during follow-up - 1 patient developed autoimmune thyroid disease as adult - Overall: chronic, intermittent clinical course with persistent histological abnormalities despite symptom improvement |
| Ref(44) | 31 | Male | No (asymptomatic) | No | No | Not mentioned | Not mentioned | None (asymptomatic, detected during health checkup) | - None mentioned - No medical history of allergies or other conditions | Yes (characteristic diffusely nodular atrophy, pseudopolyposis consisting of isolated normal mucosa) | Not mentioned in this case | Not mentioned | Diffuse nodular atrophy limited to whole gastric body, broad atrophy in whole gastric body, clearly visible atrophic border between gastric body and angle/antrum, normal mucosa in angle and antrum without atrophy, normal cardiac mucosa without atrophy, distinctive borders visible on retroflexion view | Not mentioned | Abnormal and thickened subepithelial collagen (specific measurement not provided) | Not mentioned (no colonic involvement mentioned) | - Abnormal and thickened subepithelial collagen confirmed by Masson-trichrome stain - Atrophic mucosa - Patchy deposition of collagen in lamina propria (mentioned in commentary) - Inflammatory infiltrate of mucosa (mentioned in commentary) - Collagen types I, II, and III and tenascin predominant | No treatment (observation only due to asymptomatic presentation) | 10 years follow-up | - No endoscopic changes observed over 10 years - No repeat biopsy mentioned | - Remained asymptomatic - No endoscopic changes over 10-year observation period - Classified as adult type based on age, but atypical presentation (asymptomatic, no colonic involvement) |
| Ref(45) | 15 months old | Female | Not mentioned | Not mentioned as presenting symptom | Yes (passage of intermittent loose stools for 4 months) | Yes (severe iron deficiency anemia with hemoglobin 4.8 g/dl, serum iron 0.7 mcg/dl, transferrin 1.16%, MCV 67.8 fl) | Not mentioned | Generalized body oedema (swelling), pica (eating papers), severe pallor, hypoalbuminaemia (17 g/l), hypoproteinaemia (total protein 37 g/l) | - None mentioned - Coeliac disease screening was negative - Tuberculosis workup and retroviral screening were negative | Not mentioned (stomach appeared macroscopically normal) | Not mentioned | Not mentioned | Oesophagus appeared normal, stomach appeared normal macroscopically, duodenum looked pale with thick folds, colon was oedematous with pale looking mucosa and some areas of atrophy | Negative | >10 μm (marked collagen deposition, specific measurement not provided) | No evidence of collagenous colitis (mild chronic colitis in ascending colon, descending colon and sigmoid colon with mild chronic proctitis, but no collagenous colitis) | - Gastric body showed replacement of lamina propria by hyalinized collagen - Collagen stains pink on haematoxylin and eosin stain - Collagen stains green on Masson's trichrome stain - Scattered plasma cells, focal lymphoid aggregates and associated glandular atrophy - Mild non-specific chronic oesophagitis - Mild chronic colitis in ascending colon, descending colon and sigmoid colon with mild chronic proctitis - Cytomegalovirus qualitative PCR from duodenal tissues was negative - Moderate faecal fat with normal faecal elastase - Normal faecal alpha-I anti-trypsin level | - Packed red cells and albumin transfusions (initially) - Elemental iron supplementation - Proton-pump inhibitor (omeprazole) - Zinc - Second course of albumin infusion - Oral prednisone 20 mg daily - Azathioprine 12.5 mg daily - Phosphate sandoz (for hypophosphataemia correction) - Maintenance therapy: Low-dose combined prednisone 2.5 mg and azathioprine 6.25 mg daily | Over 8 months of maintenance therapy | Not mentioned (no repeat biopsy reported) | - Initially improved with iron, omeprazole and zinc - oedema resolved - One week later oedema re-accumulated with worsening hypoproteinaemia, hypoalbuminaemia, and electrolyte imbalances (marginal hypocalcaemia and moderate hypophosphataemia) - After starting prednisone and azathioprine: oedema resolved, albumin normalized to 32 g/l, iron deficiency anemia resolved (hemoglobin 12.2 g/dl, MCV 83.6 fl) - Remission maintained on low-dose combined prednisone and azathioprine for over 8 months - Classified as pediatric type (affecting primarily stomach, though had some colonic inflammation but not collagenous colitis) - Unusual presentation with generalized body oedema and severe protein-losing state |
| Ref(46) | 30 | Male | Yes (chronic intermittent abdominal pain, epigastric tenderness) | Not mentioned as presenting symptom | No | Yes (hemoglobin level 9 g/dL) | Not mentioned | Significant weight loss for about 4 months | - None (autoimmune evaluation was not positive) - Celiac serology was normal | Yes (nodular gastric mucosa in stomach wall and antrum) | Yes (erythematous mucosa of stomach and duodenum) | Not mentioned | Nodular and erythematous appearing gastric mucosa, appearance did not improve on repeat endoscopy after 3 months | Not mentioned | >10 μm (marked thickening of sub-epithelial basement membrane in antral mucosa, specific measurement not provided) | Normal colonoscopy, no colonic involvement | - Initial gastric biopsy: chronic gastritis with no specific finding - Repeat gastric biopsy: marked thickening of sub-epithelial basement membrane in antral mucosa (H&E stain) - Moderately severe atrophy of glandular component of antrum - Sub-epithelial surface collagen deposition >10 μm accompanied by inflammatory infiltrate in lamina propria - Identified with trichrome stain | PPIs for 3 months | 3 months (repeat upper gastrointestinal endoscopy performed) | - Appearance of endoscopy did not improve - Gastric mucosal biopsy showed marked thickening of sub-epithelial basement membrane and moderately severe atrophy of glandular component of antrum | - After 3-month treatment with PPIs: abdominal pain slightly improved but continued - No improvement in endoscopic appearance - Classified as adult type (isolated collagenous gastritis without colonic involvement, which is exceptionally rare in adults) - Treatment with anti-secretory agents showed limited success - Article notes this is a rare cause of dyspepsia resistant to early therapies - No follow-up data beyond 3 months provided in this report |
| Ref(47) | 19 | Male | Yes (dizziness and gradually increasing abdominal pain, intermittent abdominal pain for one year prior to admission) | Not mentioned | No (specifically mentioned "without diarrhea") | Yes (severe anemia with hemoglobin 6 g/dL, hematocrit 20%, MCV 64 fl, severe iron deficiency with ferritin 1.3 ng/mL) | Not mentioned | Conjunctival pallor, hypoalbuminemia (serum albumin 2.5 g/dL, total protein 5.3 g/dL), elevated lactate dehydrogenase (743 U/L) | - None - Antigliadin, anti-endomysial and tissue transglutaminase antibodies were negative - Total Immunoglobulin A level was normal - No coexisting immune-related disorders mentioned | Yes (nodular gastric mucosa) | Yes (congestive gastric mucosa) | Not mentioned | Ileocolonoscopic examination was normal, findings remained unchanged on third upper GI endoscopy | Not mentioned | >10 μm (thick subepithelial collagenous band, specific measurement not provided) | Normal (biopsy specimens obtained throughout colon and ileum were normal, no collagenous colitis) | - Thick subepithelial collagenous band (>10 μm) - Inflammatory infiltrate in lamina propria including lymphocytes and plasma cells - Overlying epithelium was focally detached - Trichrome stain confirmed collagenous nature of the band - Presence of cells expressing CD25 in lamina propria - Increased number of intraepithelial T lymphocytes (expressing CD3) - Signs of immune activation - Entrapping dilated capillaries mentioned | - Prednisone 40 mg per day (started September 2008) - Progressive decrease of prednisone after 2 months of full dose (40 mg/day) - Treatment duration: approximately 6 weeks at full dose with subsequent tapering, stopped after total treatment period (18 months after stopping mentioned in follow-up) | - 2 months after starting treatment - 5 months after starting treatment (repeat biopsy) - October 2010 (last check, 18 months after stopping prednisone) - Total follow-up: 24 months | - Persistence of collagenous gastritis on biopsy specimens taken from gastric body and antrum 5 months after treatment - Third upper GI endoscopy with biopsy: findings remained unchanged - Histological lesions remained unchanged despite clinical remission - Collagen deposits remained unchanged | - Two months after starting prednisone: significant clinical and biological improvement with disappearance of abdominal pain and increase in hemoglobin level to 10 g/dL - Clinical remission maintained after progressive decrease of prednisone - At last check (18 months after stopping prednisone): stable BMI (21 kg/m²), hemoglobin rose to 14 g/dL - Classified as pediatric type/first subset (occurring in children and young adults, characterized by severe anemia and nodular gastric pattern, gastric involvement only) - Rapid clinical remission sustained after stopping treatment, but histological lesions persisted - First reported Tunisian case - No transformation to adult form during follow-up |
| Ref(48) | 85 | Female | Yes (several days of diffuse, crampy abdominal pain, worse with food; diffusely tender abdomen in all quadrants with guarding) | No (specifically denied) | No (specifically denied) | Yes (mild microcytic hypochromic anemia: hemoglobin 11.4 g/dl, MCV 77.5 fl, MCH 23.4 pg) | Not mentioned | Weight loss (9.1 kg/20 lb over 5 months), difficulty eating, severe anxiety when sitting down to a meal, no melena or hematochezia | - None - Celiac sprue serology negative (antigliadin, antitissue transglutaminase and antiendomysial antibodies were negative) - Past medical history: mild dementia, hypertension, hyperlipidemia | Yes (endoscopic nodular appearance mentioned as common) | Yes (diffuse severe erythema and inflammation) | Yes (erosion seen on histology) | Friability, granularity around entire examined stomach, gastric wall thickening on CT scan suggestive of gastritis; repeat endoscopy showed grossly normal stomach and duodenum | Negative (Warthin Starry stain was negative for *H. pylori*) | >10 μm (sub-epithelial collagen deposition, specific measurement not provided) | Not mentioned (colonoscopy was not performed, cannot confirm whether there was lower GI involvement) | - Active chronic inflammation with sub-epithelial collagen deposition and erosion - Plasma cell and lymphocytic infiltration of superficial and deep mucosa - Thickened sub-epithelial collagen deposition >10 μm - Entrapping dilated capillaries - Hematoxylin and eosin staining used - Repeat biopsies at 6 months: normal | - Initial unsuccessful treatment: Pantoprazole 40 mg twice daily and sucralfate 1 g orally four times daily for several days - Methylprednisolone 20 mg IV every 12 hours for 4 days (with rapid improvement) - Transitioned to prednisone 40 mg daily at discharge - Prednisone taper over 2.5 months | - 6 months follow-up - 1 year follow-up mentioned (remains without complaints) | - Repeat endoscopy at 6 months: grossly normal stomach and duodenum - Repeat biopsies at 6 months: normal (complete histological resolution) - Complete absence of collagen deposit on repeat biopsy | - Several days without response to pantoprazole and sucralfate - Rapid improvement after starting methylprednisolone IV - At 6-month follow-up: marked improvement in appetite, resolution of abdominal pain, tolerating oral diet, gained 6.8 kg (15 lb) - Complete and rapid resolution of symptoms with steroid treatment - Remains without complaints 1 year later - Rare presentation: elderly patient with presentation more typical among younger cases (abdominal pain and anemia rather than diarrhea) - Unusual for geriatric case - no lower GI symptoms - Complete clinical, endoscopic, and histological resolution achieved - One of the rare cases with total resolution including histologic improvement after steroid therapy |
| Ref(49) | 42 | Female | Not mentioned | Not mentioned | Not mentioned | Not mentioned | Not mentioned | None mentioned (underwent upper gastrointestinal barium radiography for gastric cancer screening) | Sjögren syndrome (7-year history) | Yes (nodular, flat-topped lesions diffusely distributed in upper and middle thirds of stomach, multiple flatly elevated lesions, fine and fading nodularity) | Not mentioned | Not mentioned | Slightly depressed atrophic mucosa surrounding nodules; Magnifying endoscopy with narrow-band imaging (M-NBI) revealed tubular structures on surface of nodules and areas of non-structure accompanied by thin and tortuous vessels in intervening mucosa; double-contrast radiography showed nodular, flat-topped lesions | Negative (histology, serology and urea breath test were negative for *H. pylori* infection) | Markedly thickened subepithelial collagen bands (specific measurement not provided; dense collagen depositions in intervening atrophied mucosa; specimens from nodules showed mild inflammation without thickening in subepithelial collagen band) | No collagenous colitis (colonoscopy with biopsies did not show any sign of collagenous colitis) | - Infiltration with inflammatory cells in lamina propria - Severely atrophied epithelium - Markedly thickened subepithelial collagen bands (revealed by Azan staining at 200x) - Dense collagen depositions in intervening, atrophied mucosa - Nodules contained mild inflammatory infiltrates without thickened collagen bands - No sign of collagenous sprue (capsule endoscopy performed) | Not mentioned | Not mentioned | Not mentioned (no repeat biopsy reported) | - Diagnosed as collagenous gastritis - Classified as adult type based on age and association with autoimmune disease (Sjögren syndrome) - No colonic or small bowel involvement - Investigation included capsule endoscopy and colonoscopy with biopsies - Article focuses on novel M-NBI (magnifying narrow-band imaging) endoscopic findings - Co-existence of two distinctive M-NBI findings: (1) tubular gastric micromucosal pattern (representative of inflammatory cell infiltration and epithelial degeneration) and (2) obscure surface structure with irregular, narrowed, and coiled subepithelial capillaries (compatible with moderate to severe mucosal atrophy and intestinal metaplasia) - No clinical symptoms mentioned despite histological findings |
| Ref(50) | 15 | Male | Yes | Not mentioned | Yes (daily rectal blood loss mixed with normal stool) | Yes (significant microcytic anemia: Hb 7.5 g/dl, MCV 58.8 fL; chronic iron deficiency with iron 7 µg/dl, ferritin 1.5 ng/l, reticulocyte count 33,900/µl) | Not mentioned | Rectal blood loss, recurrence of anemia (11.5 g/dl) after stopping PPI | - None - Anti-transglutaminase antibodies excluded celiac disease | Yes (nodular/pavimentous aspect of fundus) | Not mentioned | Not mentioned | Normal esophagus, antrum, and duodenum; rectoscopy revealed slightly hemorrhoidal congestion; normal ileo-colonoscopy | Negative (*H. pylori* was not found) | >10 µm (significant thickening of basement membrane) | Normal (normal ileo-colonoscopy including histological analysis) | - Significant thickening of basement membrane (>10 µm) - Chronic inflammatory infiltration of chorion - Some stripping of surface epithelium - Normal duodenal villi - Fecal calprotectin slightly elevated (223 µg/g) | - Oral iron supplement (iron sulfate 80 mg a day) - PPI (esomeprazole 40 mg a day) - Previous treatment: PPI stopped several months before with recurrence of anemia | 3 months (0.25 years) | Not mentioned (no repeat biopsy reported) | - Anemia resolved within 3 months under oral iron supplement and PPI - Hemoglobin value reached 13.5 g/dl, MCV increased to 78 fL - Previous recurrence of anemia after stopping PPI - Classified as pediatric subtype with severe anemia and gastrointestinal bleeding without other digestive involvement |
| Ref(50) | 10 | Female | Yes (epigastric pain 6 months earlier) | Not mentioned | No | Yes (microcytic anemia: Hb 6.9 g/dl, MCV 62 fL, serum iron 15 µg/dl, ferritin 5 µg/l) | Not mentioned | Fatigue, pallor, irritability, recurrent upper airway infections, no melena or hematemesis | - None - Coeliac disease serology negative (anti-transglutaminase antibodies) | Yes (nodular pattern of gastric fundus) | Yes (erosive pattern) | Yes (diffuse erosive pattern of gastric fundus) | Small fundic ulcer showing signs of recent bleeding, normal ileo-colonoscopy | Negative (*H. pylori* was not found) | Thickening of basement membrane (>10 µm, specific measurement not provided) | Normal (macroscopically and histologically normal ileo-colonoscopy) | - Thickening of basement membrane - Chronic inflammatory infiltration of chorion - No other abnormalities on antral and duodenal samples - Normal B9 and B12 vitamin serum values | - Blood transfusion (initially) - Oral iron supplement | - 6 months after transfusion - 0.5 years (6 months) | Not mentioned (no repeat biopsy reported) | - Hemoglobin increased to 13.7 g/dl after blood transfusion and iron supplements - Three months after transfusion under oral iron supplement only: hemoglobin 11.4 g/dl - Six months after transfusion and three months after stopping iron supplement: hemoglobin decreased to 9.7 g/dl with decreased serum iron and ferritin (low normal range), reticulocyte count increased to 105,700/µl - Prompt recurrence after stopping treatment - Classified as pediatric subtype |
| Ref(51) | 18 | Male | Yes (2-month history of diffuse cramping abdominal pain of fluctuating intensity with postprandial exacerbation, diffuse abdominal tenderness to palpation) | Not mentioned | Not mentioned | Not mentioned | Not mentioned | No rebound or guarding on physical examination | - None mentioned - Medical history was unremarkable before these symptoms | Yes (diffuse nodular appearance of gastric fundus and body) | Not mentioned | Not mentioned | Antral sparing; repeat EGD after treatment showed persistent findings | Not mentioned | Subepithelial collagenous band thickening (specific measurement not provided, confirmed with trichrome staining); repeat biopsy showed patchy subepithelial collagenous band thickening | Not mentioned (no colonoscopy performed) | - Mild active chronic inflammation of fundic mucosa - Subepithelial collagenous band thickening (confirmed with trichrome staining) - Repeat biopsy after treatment: persistent mild chronic inflammation and patchy subepithelial collagenous band thickening | - Topical budesonide therapy (initiated after diagnosis) - Several months of tapered therapy - Continued low-dose topical budesonide (ongoing) | - Several months after treatment initiation - Ongoing endoscopic surveillance planned | - Repeat EGD with gastric biopsy specimens showed persistent mild chronic inflammation and patchy subepithelial collagenous band thickening - No complete histological resolution despite symptomatic improvement | - Complete symptomatic resolution after several months of tapered budesonide therapy - Despite clinical improvement, histological findings persisted on repeat biopsy - Continued on low-dose topical budesonide with ongoing endoscopic surveillance planned - Classified as young adult/pediatric type based on age and presentation (abdominal pain, gastric involvement) - No mention of colonic involvement - Article notes that etiology, pathogenesis, and prevalence remain to be fully determined - Limited knowledge of relationship with other collagenous gastroenteritides and effective treatment algorithms due to rarity |
| Ref(52) | - 12 years old (at presentation of gastritis symptoms) - Previously diagnosed with celiac disease at 13 months old | Female | Yes (recurrent periumbilical abdominal pain starting at age 8) | Yes (frequent vomiting) | Not mentioned | Yes (iron deficiency anemia: Hb 11.1 g/dl, MCV 72.9 fl, ferritin 2 ng/ml, poor response to oral iron therapy) | Not mentioned | Growth stagnation (weight and height), vitamin B12 deficiency (86 pg/ml), achlorhydria, hypergastrinemia (gastrin 617 pg/ml) | - Celiac disease (diagnosed at 13 months with failure to thrive, positive celiac antibodies, HLA-DQ 2/8 positive, intestinal biopsy with villous atrophy Marsh 3C) - Maintained asymptomatic on gluten-free diet until age 8 - Autoimmune gastritis antibodies negative (anti-parietal cell, anti-intrinsic factor, anti-gastric ATPase) | Yes (markedly nodular appearance of gastric mucosa, micronodular fundic mucosa, nodular pangastritis) | Not specifically mentioned | Not mentioned | Markedly friable gastric mucosa, atrophic appearance at pyloric antrum level, presence of eosinophils in lamina propria; persistence of nodular pangastritis on follow-up despite clinical improvement | Empirical *H. pylori* eradication therapy was given without clinical or histological improvement (suggests negative or unsuccessful eradication) | Not specifically measured, but histological findings confirmed collagenous gastritis with mild atrophy of oxyntic mucosa | Not mentioned (no colonoscopy performed) | - Collagenous gastritis with mild atrophy of oxyntic mucosa - Presence of eosinophils in lamina propria - Atrophic pattern (uncommon in childhood) - Achlorhydria associated - Initial biopsies were non-conclusive - Follow-up: persistence of nodular pangastritis histologically despite clinical improvement | - Iron and vitamin B12 supplementation - *H. pylori* eradication therapy (empirical) - no clinical or histological improvement - Oral prednisone for 8 weeks - no response - Elemental diet (hypoallergenic diet excluding cow's milk protein, egg, soy, legumes, gluten, fish/seafood, and nuts) - significant clinical improvement - Current diet: gluten-free, soy-free, egg-free - Contraindication noted: PPIs or H2 antagonists contraindicated due to associated achlorhydria | - At least 6 months (when food reintroduction began) - Currently asymptomatic on restricted diet | - Last endoscopic control: histological findings confirmed collagenous gastritis with mild atrophy of oxyntic mucosa - Persistence of nodular pangastritis despite clinical improvement - No complete histological resolution | - Celiac disease diagnosed at 13 months, asymptomatic on gluten-free diet until age 8 - At age 8: onset of abdominal pain, vomiting, growth stagnation - Poor response to oral iron therapy for anemia - No improvement with *H. pylori* eradication or prednisone - Significant clinical improvement with elemental/hypoallergenic diet - Recovery of iron and vitamin B12 levels, able to discontinue supplementation - After 6 months: progressive food reintroduction - Currently asymptomatic on diet excluding gluten, soy, and egg - Classified as pediatric type with association to autoimmune disease (celiac disease) - Unusual presentation: rare combination of celiac disease and collagenous gastritis in childhood - Atrophic pattern uncommon in childhood - Unique favorable response to hypoallergenic diet |
| Ref(53) | 8 | Male (Boy) | Yes (newly developed during treatment) | Yes (6-month history of intermittent vomiting, occurring once or twice per month; increased frequency later; intractable vomiting) | No | Yes (initially Hb 10.4 g/dL, MCV 80 fl, ferritin 3.5 ng/mL, iron 25 mg/dL, TIBC 390 mg/dL; later worsened to Hb 8.6 g/dL, MCV 65 fl, ferritin 2.4 ng/mL, iron 19 mg/dL, TIBC 414 mg/dL) | Not mentioned | No dysphagia, no melena (initially); stool occult blood positive (later) | None mentioned | Yes (nodular mucosa throughout gastric body with relative sparing of antrum; more prominent nodular mucosa at 6-month follow-up, well demarcated and irregular in size with multiple small polypoid lesions) | Not mentioned | Not mentioned | Colonoscopy with biopsy unremarkable | Negative (no detected *H. pylori* organisms) | Patchy deposition of collagenous tissue (up to 43.8 μm thickness confirmed by Masson trichrome staining) | Not applicable (colonoscopy unremarkable) | - Initial biopsy from nodules: chronic active gastritis with lymphocytes and eosinophils, no *H. pylori* or granulomas - Second biopsy from intervening mucosa between polypoid lesions: patchy deposition of collagenous tissue with prominent eosinophil infiltration (40-70 eosinophils/high power field) in lamina propria - These findings not observed in first biopsy specimens - Confirmed by Masson trichrome staining showing blue thick band of collagen in subepithelial area | - Initial: histamine receptor antagonist (2-week course) and iron replacement therapy (5 mg/kg/d) - After diagnosis: 10-week course of prednisolone with PPI for abdominal pain and vomiting - Continued iron supplementation | - 6 months between first and second EGD - 6-month follow-up after starting prednisolone | - Second EGD showed more prominent findings - No information on repeat biopsy after treatment | - Initial vomiting ceased with treatment, iron status showed slight improvement - About 1 year later: increased vomiting frequency, worsened iron status - First EGD: nodular mucosa, diagnosis not confirmed - Second EGD (6 months later): more prominent nodularity, collagenous gastritis confirmed - After prednisolone course: clinical symptoms improved, iron level within normal range with continued supplementation - Hb level returned to normal but ferritin levels consistently low - Required targeted biopsies of intervening mucosa (not nodules) to confirm diagnosis |
| Ref(53) | 11 | Male (Boy) | No | No | No | Yes (intractable anemia: Hb 6.0 g/dL, MCV 60 fl, ferritin <5 ng/mL, iron 15 mg/dL, TIBC 472 mg/dL) | Not mentioned | Fatigue, pallor, no GI symptoms | None mentioned | Yes (coarsely nodular mucosa in gastric body with normal antrum; striking nodularity of gastric body at 20-month follow-up with relative sparing of antrum) | Not mentioned | Not mentioned | Ileocolonoscopy with colon biopsy and capsule endoscopy unremarkable | Negative (no *H. pylori* or lymphoid follicles) | Focal deposition of fibrocollagenous tissue (specific measurement not provided) | Not applicable (ileocolonoscopy unremarkable) | - Initial biopsy: diffuse lymphocytic infiltrations, no *H. pylori* or lymphoid follicles, chronic active gastritis - Repeat histology (20 months later): focal deposition of fibrocollagenous tissue and diffuse lymphoplasmacytic infiltrate in lamina propria with partial detachment of surface epithelium, consistent with collagenous gastritis | - Oral iron replacement therapy - No other treatments mentioned | - 20 months between first and second EGD - Regular follow-up after diagnosis | Repeat histology showed fibrocollagenous deposition consistent with collagenous gastritis | - Previously diagnosed with IDA in hematology department - • Initially treated with oral iron, Hb reached maximum 11.4 g/dL - Hb levels fluctuated with adherence to iron treatment - No focus of GI bleeding identified initially - Follow-up EGD at 20 months: striking nodularity, collagenous gastritis diagnosed - After diagnosis: remained asymptomatic with normal growth and resolution of anemia on continued oral iron - Required repeat endoscopy and targeted biopsy for diagnosis |
| Ref(53) | 15 | Female (Girl) | No | No | No | Yes (asymptomatic with recurrent IDA episodes: initially Hb 8.1 g/dL, reached maximum 14.8 g/dL with treatment, decreased to 10.5 g/dL after discontinuation) | Not mentioned | Asymptomatic, normal menstruation, denied any medication | None mentioned | Yes (prominent nodularity in antrum at 7-month follow-up) | Not mentioned | Yes (multiple ulcerations initially) | Initially edematous mucosa with multiple ulcerations and whitish mucus throughout gastric antrum; few polyps on gastric body at follow-up; ileocolonoscopy and capsule endoscopy unremarkable | Negative (no *H. pylori*, cytomegalovirus, or granuloma identified) | Irregular subepithelial collagenous band averaging 35 to 45 μm thickness | Not applicable (ileocolonoscopy unremarkable) | - Initial biopsy: chronic active gastritis without *H. pylori*, cytomegalovirus, or granuloma - Follow-up biopsy (7 months later) from intervening mucosa between polyps: irregular subepithelial collagenous band averaging 35-45 μm thickness with mild inflammation, confirming collagenous gastritis | - Oral iron supplementation - Short course of PPI - No other treatments mentioned | - 7 months between first and second EGD - Continued follow-up | Follow-up biopsy confirmed collagenous gastritis with collagen band 35-45 μm | - 4-year history of recurrent IDA requiring oral or IV iron - Initially no evidence of occult GI blood loss - Anemia did not completely resolve with iron and PPI initially - Follow-up EGD at 7 months: prominent nodularity, collagenous gastritis diagnosed - After diagnosis: doing well without GI symptoms, maintained Hb >12 g/dL with continuing oral iron - Required repeat endoscopy and targeted biopsy for diagnosis |
| Ref(54) | 37 | Female | No (denied abdominal pain) | Not mentioned | No (denied) | No (CBC performed one year prior was normal: hemoglobin 145 g/L) | Not mentioned | Dysphagia to solid foods (several year history), denied symptoms of reflux; increased NSAID intake for a month prior to presentation | - None - Celiac screen negative (anti-transglutaminase IgA <1.0 U/ml, IgA 2.36 g/L) - IgG4 titre normal (0.27 g/L) | Yes (vague nodularity in gastric body, mucosal cobblestoning in gastric body) | Not mentioned | Not mentioned | Gastritis appearance; friable tissue in gastric body; esophageal webs (two), abnormal esophageal mucosa, multiple rings in esophagus, friable esophageal tissue; gastroesophageal junction outlet obstruction; colonoscopy normal; persistent esophageal webs and gastric mucosal cobblestoning on repeat EGD 6 months later | Negative (Giemsa stain negative; not detected in any biopsies taken) | Significant subepithelial collagen plate thickening (>10 μm, specific measurement not provided; highlighted by trichrome stain) | Normal (colonoscopy and biopsies unremarkable) | - Mild chronic active gastritis with mild focal gastric atrophy - Significant subepithelial collagen plate thickening (highlighted by trichrome stain) - Entrapped inflammatory cells, red blood cells, and small capillaries - Lamina propria expanded, predominantly by plasma cells with admixed eosinophils and lymphocytes - Surface epithelium atrophic with intraepithelial neutrophils - Congo red stain: no amyloid deposition - Serum protein electrophoresis: no monoclonal protein - Urine protein electrophoresis: minor albuminuria only - First repeat EGD: minimal chronic inflammatory cell infiltrates and prominent mucosal lymphoid aggregates despite endoscopic cobblestoning (collagenous gastritis not confirmed in this biopsy) - Second repeat EGD (6 months): moderate chronic active gastritis with increased fibrosis beneath surface epithelium and within lamina propria (gastric body); mild increase in fibrosis of lamina propria (gastric antrum) - Duodenum, ileum, colon biopsies: unremarkable - Esophageal biopsies: no significant pathological abnormality | - Dexlansoprazole (already taking before presentation, continued on same dose) - No new treatment initiated after collagenous gastritis diagnosis - Esophageal dilation (for gastroesophageal junction outlet obstruction) | - Subsequent upper and lower endoscopy performed - Repeat EGD 6 months later - Barium swallow study performed | - First repeat EGD: minimal chronic inflammation, prominent mucosal lymphoid aggregates, no significant collagenous gastritis findings despite endoscopic cobblestoning - Second repeat EGD (6 months): persistent histological evidence of collagenous gastritis with moderate chronic active gastritis and increased fibrosis | - Presented with dysphagia (several year history) - Initial EGD: esophageal webs and gastritis with vague nodularity, collagenous gastritis diagnosed on biopsy - Subsequent investigations to rule out collagenous disease elsewhere: colonoscopy normal, duodenum/ileum/colon biopsies unremarkable - Barium swallow: gastroesophageal junction outlet obstruction - Esophageal dilation performed - First repeat EGD: minimal chronic inflammation despite endoscopic cobblestoning (possible sampling issue) - Second repeat EGD (6 months): persistent esophageal webs and gastric cobblestoning with confirmed collagenous gastritis - Remained asymptomatic for gastric pathology throughout - Clinical management unchanged after extensive investigations - Unusual presentation: Incidental finding of collagenous gastritis in asymptomatic patient (dysphagia explained by esophageal webs, likely NSAID-related) - Third reported case of incidental collagenous gastritis in asymptomatic patient or patient with unrelated symptoms - Does not fit either classic presentation (pediatric with pain/anemia or adult with diarrhea/collagenous colitis) - Article highlights importance of proper sampling (collagen deposition occurs preferentially in depressed areas of cobblestoning) - First repeat biopsy may have missed collagenous gastritis due to inadequate sampling of depressed areas - Emphasizes judicious use of biopsies and careful interpretation in clinical context |
| Ref(55) | - Median age at diagnosis: 9.9 years (range 4.4-17 years) - Median age at presentation: 9.8 years (range 4.3-16 years) - Age at study inclusion: 8.7-23 years (median 15 years) | - 12 female patients (80%) - 3 male patients (20%) - Female to male ratio: 4:1 | - 6/15 patients (45%) reported recurrent abdominal pain at presentation - Pain was not severe in most cases and did not affect daily activities - 1 patient experienced recurrent abdominal pain affecting daily activities during follow-up | Not specifically reported | - 1 patient (Patient H) presented with watery, non-bloody diarrhea (had concurrent collagenous colitis) - 1 patient (Patient F) had non-bloody diarrhea | - 14/15 patients (93%) had iron deficiency anemia at presentation - Most cases were severe anemia - Hemoglobin range: 28-137 g/L (reference 115-160 g/L) - Iron deficiency recurred in 13/14 patients within median 1 year (range 1-2 years) | Not specifically reported | - Fatigue (5 patients as chief complaint) - Pallor (4 patients) - Palpitation (2 patients) - Headache (1 patient) - Vertigo (1 patient) - Gastroesophageal reflux (1 patient) - No hematemesis, melena, or hematochezia in any patient - Fecal hemoglobin testing negative in 12/15 tested patients | - Personal autoimmune diseases:   - No established autoimmune comorbidities in any patient at diagnosis or during follow-up  - 1 patient developing weak tissue transglutaminase IgA antibodies suggesting celiac disease under development   - Family history (first-degree relatives):   - 7/15 patients (47%) had heredity for autoimmune diseases  - Celiac disease in relatives (2 patients)  - Hypothyroidism in relatives (3 patients)  - Type 1 diabetes in relatives (2 patients)  - Ulcerative colitis in relatives (1 patient)  - Psoriasis in relatives (1 patient)  - Latent autoimmune diabetes in adults in relatives (1 patient)   - Autoantibodies detected:   - 6/15 patients (40%) tested positive for autoantibodies  - ANA positive: 3/15 (20%)  - Anti-smooth muscle antibody: 2/15 (13%)  - Anti-thyroid peroxidase antibodies: 1/15  - Tissue transglutaminase IgA: 1/15  - Atypical pANCA: 1/15   - HLA genotype:   - HLA DQ2/DQ8 positive: 8/15 (53%)  - DQ2.5 haplotype: 5/15 (33%) | - 13/15 patients (87%) showed mucosal nodularity - Locations: gastric body, fundus, and/or antrum | - 4/15 patients (27%) - Locations: gastric antrum, body | 1/15 patient had a few erosions in gastric body and fundus | - Edematous mucosa (1 patient) - Traces of coagulated blood (1 patient) - Hypertrophic mucosa/folds (2 patients) - Mucosal irritation (1 patient) - Raised mucosal stripes (1 patient) - Cobblestone appearance (1 patient) - Pseudopolyposis (1 patient) - Unremarkable appearance (1 patient, but abnormal in follow-up) | No signs of concurrent *H. pylori* gastritis in any patient | - Range: 15-100 μm at diagnosis - All patients had >10 μm thickness (diagnostic criterion) | - 1/15 patient had concurrent collagenous colitis (Patient H) - Collagen thickness not specifically stated for colon | - Inflammatory pattern:   - 13/15 patients (87%) exhibited eosinophil-rich inflammation (≥30 eosinophils/HPF)  - Range: up to 150 eosinophils/HPF  - Intraepithelial lymphocytosis (>25 lymphocytes/100 epithelial cells): 1/15 patient   - Collagen distribution:   - Patchy: 9 patients  - Focal: 4 patients  - Diffuse: 3 patients   - Collagen location:   - Equal antrum and corpus: 6 patients  - Corpus predominant: 6 patients  - Antral predominant: 2 patients  - Corpus only: 1 patient   - Associated findings:   - Collagenous inflammation restricted to stomach: 13/15 (87%)  - Concurrent collagenous duodenitis: 1 patient  - Concurrent collagenous colitis: 1 patient  - Subtotal/total villous atrophy in duodenum: 3 patients  - No intestinal metaplasia or advanced mucosal atrophy in gastric corpus in any patient | - Iron supplementation:   - All 14 patients with iron deficiency anemia received iron supplementation  - Oral iron (ferrous asparto glycinate plus polysaccharide iron complex or iron sulphate): primary choice  - Intravenous iron (ferric carboxymaltose): used when oral iron caused gastrointestinal side effects   - PPI:   - Attempted in 5 patients  - 3 patients receiving PPI at study inclusion  - No endoscopic or histologic improvement observed   - Dietary modifications:   - Attempted in 7 patients  - Cow's milk-free diet: 4 patients  - Gluten-free diet: 2 patients  - Diet free of cow's milk, soy, egg, and wheat: 1 patient  - No endoscopic or histologic improvement observed   - Corticosteroids/immunomodulators:   - 1 patient with concurrent collagenous colitis receiving maintenance budesonide therapy  - No steroids or immunomodulators administered to remaining 14 patients | - Duration:   - Median follow-up from initial presentation: 6.2 years (range 0.4-11 years)  - Median duration of disease at study inclusion: 6.2 years (range 1.0-11 years)   - Follow-up endoscopies:   - Performed in 11/15 patients (73%)  - Median time from diagnosis to latest gastroscopy: 4.4 years (range 1.4-7.4 years)   - Colonoscopies:   - Performed in 11/15 patients (73%)  - No additional pathologies except 1 patient with collagenous colitis | - Gastric mucosa:   - No endoscopic or histologic normalization in any patient during follow-up  - Persistence of >10 μm-thick subepithelial collagen deposits in all examined patients  - No worsening of mucosal inflammation over time  - No development of intestinal metaplasia  - No development of advanced mucosal atrophy in gastric corpus   - Other sites:   No new collagenous inflammation developed in colon or ileum (except the 1 patient with pre-existing collagenous colitis) | - Chronic disease:   - Endoscopic and histologic findings remained pathologic in all examined patients  - Median follow-up: 4.4 years   - Symptom evolution:   - Most patients remained asymptomatic during follow-up except for recurrent iron deficiency  - Iron deficiency recurred in 13/14 patients, requiring repeated iron supplementation  - 1 patient experienced recurrent abdominal pain affecting daily activities  - 1 patient with collagenous colitis had occasional watery diarrhea despite budesonide therapy   - Treatment response:   - All 14 patients with iron deficiency anemia responded to iron supplementation with normalization of hemoglobin  - No response to PPI or dietary modifications (no endoscopic/histologic improvement)   - Inflammatory markers:   - Serum calprotectin increased: 10/15 (67%)  - Serum amyloid A increased: 5/15 (33%)  - Plasma CRP normal in all but 1 patient  - ESR and CRP normal in all tested patients during disease course   - No malignant transformation observed |
| Ref(56) | 71 | Female | - Intermittent epigastric spasms, predominantly after food ingestion - Multiple hospital admissions for epigastric spasms | Not reported | Not reported | - Not reported - Complete blood count was negative | Yes - epigastric fullness associated with early satiety | - Dyspepsia (worsening over 6 months) - Weight loss: 27 kg over 6 months - Long history of gastroesophageal reflux disease (GERD) - Unable to tolerate food at 8 months follow-up (worsening symptoms) | - Personal autoimmune diseases:   No autoimmune diseases reported   - Family history:   Not reported   - Autoantibodies/Laboratory:   - Anti-transglutaminase immunoglobulin A: negative  - CA 19-9: negative  - Vitamin B12: normal  - Complete metabolic panel: normal   - Other associations:   - Literature notes multiple case reports describe association between autoimmune diseases and CG, most commonly celiac disease  - No such associations found in this patient | Not mentioned in this case | Not mentioned in this case | Not mentioned in this case | - Tubular shaped stomach - unique finding resembling gastric sleeve (not previously reported in literature) - Diffuse atrophic mucosa in gastric body - Chronic gastritis on initial EGD - Localized submucosal wall thickening in entire body of stomach (up to 5 mm thickness on EUS) - Some case reports in literature show normal gastric mucosa; others show mucosal erythema, erosions, or ulcers | Not reported | - Greater than 10 µm (diagnostic criterion met) - Thickened subepithelial collagen layer confirmed on Masson Trichrome stain - Exact measurement not specified | - No evidence of collagen deposition in colon - No evidence of collagen deposition in small bowel | - Collagen deposition:   - Thickened subepithelial collagen layer on Masson Trichrome stain (blue color)  - Subepithelial collagen deposition >10 µm with associated inflammatory infiltrate  - Surface epithelial damage present  - Heterogeneous collagen deposition (tends to be found in depressed areas between nodularity)   - Inflammatory pattern:   - Atrophic pattern classification  - Atrophic antral glands with pyloric-type gland hyperplasia  - Dense chronic inflammatory infiltrates within lamina propria  - Mucosal inflammatory infiltrates with lymphocytes, eosinophils, and plasma cells (mostly in lamina propria - per literature)   - Special stains:   - Crystal Violet: negative  - Congo Red: negative (no evidence of amyloid deposition)   - Initial biopsies:   - Random gastric biopsies with H&E and trichrome staining did not confirm diagnosis initially  - Revealed collagen deposition but diagnosis not confirmed at that time  - Repeat EGD with tunneled biopsies from gastric body and antrum confirmed diagnosis   - Distribution:   Isolated to stomach (no small bowel or colon involvement) | - Prior to diagnosis:   - PPIs for 5 years for GERD control  - PPIs continued during workup   - After diagnosis:   - Sucralfate - added initially  - Small frequent meals - recommended  - Mild symptom improvement with above measures   - Budesonide:   - Started on budesonide 9 mg/day after worsening symptoms at 8 months  - Good clinical response at 6 months follow-up after starting budesonide  - Significant clinical improvement noted   - Not used:   - No alcohol, tobacco, or nonsteroidal anti-inflammatory drugs  - Endoscopic ultrasound-fine needle biopsy not performed | - Duration:   - Initial follow-up at 8 months (worsening symptoms, repeat EGD performed)  - Follow-up 6 months after starting budesonide (total ~14 months from diagnosis)   - Additional workup:   - Upper gastrointestinal series: negative for gastric outlet obstruction  - Gastric emptying study: negative  - Magnetic resonance imaging enterography: negative for small bowel strictures, noted tubular shaped stomach  - EGD with endoscopic ultrasound performed for further characterization   - Repeat EGD:   - Repeat EGD with tunneled biopsies performed at 8 months for diagnosis confirmation  - Repeat EGD for biopsy has not yet been performed after budesonide treatment | Not available:   - Repeat EGD for biopsy has not yet been performed after budesonide treatment - No histological follow-up data available | - Initial presentation:   6-month history of worsening dyspeptic symptoms with significant weight loss (27 kg)   - Diagnostic delay:   - Initial EGD showed only chronic gastritis  - Random biopsies showed collagen deposition but did not confirm diagnosis  - Sampling error due to heterogeneous collagen deposition  - Diagnosis confirmed only after repeat EGD with tunneled biopsies   - Disease progression:   - Progressive worsening over 8 months  - Multiple hospital admissions for intermittent epigastric spasms  - Became unable to tolerate food   - Treatment response:   - Mild improvement with sucralfate and dietary changes  - Significant clinical improvement with budesonide 9 mg/day at 6 months follow-up   - Unique features:   - Tubular shaped stomach - unique finding not previously reported in literature  - Likely associated with collagen deposition  - Presented with early satiety and weight loss as result of tubular stomach configuration   - Complications:   - No gastric perforation  - No evidence of malignancy   - Overall:   - Chronic progressive course with good response to budesonide therapy  - Isolated gastric involvement without colonic or small bowel disease |
| Ref(57) | 45 | Female (Caucasian woman) | - Intermittent epigastric discomfort for several months - Minimal epigastric pain during readmission - Continued intermittent epigastric discomfort at 3-month follow-up | Denied nausea and vomiting initially | 3-4 loose, non-bloody bowel movements per day at 3-month follow-up | - Microcytic anemia: hematocrit 24%, MCV 78 fL - Iron deficiency anemia - After treatment: hematocrit improved to 35%, iron 127 µg/dL, ferritin 32 mg/mL, MCV 81 fL | Denied early satiety | - Melena for 3 days (initial presentation) - Recurrent melena (readmission 6 months later) - Substernal pyrosis and dysphagia - Daily GERD symptoms - Denied long-term NSAID use or weight loss | - No significant past medical history - No autoimmune diseases | - Nodular gastric body (initial EGD) - Cobblestone-appearing proximal gastric body (repeat EGD) - Previously noted nodular area in gastric body (third EGD) - Pale-appearing mucosa in gastric body (fourth EGD after treatment) | Not specifically mentioned | Areas of erosions noted on histopathology | - Normal esophagus and duodenal mucosa (initial EGD) - Otherwise normal stomach initially - Single 1.5 cm linear gastric ulcer in lesser curvature of gastric body (third EGD) - Colonoscopy and small bowel capsule endoscopy unremarkable - Nodular mucosa was patchy and in proximal gastric body - Cobblestone appearance of gastric mucosa typical | - Negative for *H. pylori* | - Thickening of subepithelial collagen layer in areas of erosions (initial biopsy) - Focal fibrosis of superficial lamina propria (trichrome staining, third EGD) - Thickened basement membrane (fifth EGD - recurrent disease) - >10 µm thick | - No collagenous colitis - Article focuses on collagenous gastritis without enteritis or colitis | - Initial biopsy (gastric body, 4 biopsies):   - Moderate mixed chronic inflammation of lamina propria  - Mild increase in eosinophils  - Thickening of subepithelial collagen layer in areas of erosions   - Second EGD:   - Gastric fundic mucosa: chronic inflammation and glandular microcysts  - Eosinophilic infiltrate >25 eosinophils/HPF  - Antral mucosa: no significant inflammation  - Presumed diagnosis: eosinophilic gastritis   - Third EGD (gastric body biopsies):   - Suggestive of collagenous gastritis  - Trichrome staining confirmed focal fibrosis of superficial lamina propria  - Entrapping capillaries and inflammatory cells   - Fourth EGD (after treatment):   - Multiple biopsies from duodenum, antrum, incisura, body, fundus: unrevealing  - Resolution of collagenous gastritis both endoscopically and histologically   - Fifth EGD (recurrent disease):   - Antrum and fundus biopsies: normal  - Body biopsies: chronic inactive gastritis with 5-10 eosinophils/HPF   - Thickened basement membrane consistent with recurrent collagenous gastritis - Follow-up EGD (4 and 6 years):   - Resolution of collagenous gastritis  - No intestinal metaplasia | - Initial treatment:   - Pantoprazole 40 mg daily for 1 month  - Oral iron supplementation  - Marked improvement in symptoms and laboratory values   - After symptom recurrence:   - Omeprazole 20 mg daily for 4 months (partial symptom relief)  - 6-food elimination diet (soy, dairy, wheat, peanuts/tree nuts, shellfish/fish, eggs)  - Declined prednisone initially   - After third EGD (confirmed CG diagnosis):   - Prednisone 20 mg daily + Pantoprazole 40 mg daily  - Resolution of collagenous gastritis after 1 month  - Tapered off prednisone  - Continued pantoprazole due to GERD symptoms and recurrent bleeding   - Long-term maintenance:   Treated indefinitely with pantoprazole without need for steroid therapy | - Timeline:   - 1 month after initial treatment: marked improvement  - 1 month after finishing pantoprazole: symptoms returned  - 6 months later: readmitted with melena  - 1 month after starting prednisone: fourth EGD showing resolution  - 3-month follow-up after stopping prednisone: intermittent symptoms, fifth EGD  - 4 years after initial presentation: follow-up EGD  - 6 years after initial presentation: follow-up EGD   - Endoscopic follow-up:   - First EGD: initial diagnosis consideration  - Second EGD: presumed eosinophilic gastritis  - Third EGD: confirmed collagenous gastritis  - Fourth EGD (1 month after prednisone): resolution  - Fifth EGD (3 months after stopping prednisone): recurrent disease  - Follow-up EGDs at 4 and 6 years: continued resolution | - Fourth EGD (after 1 month of prednisone 20 mg daily):   - Complete histological resolution  - Multiple biopsies from duodenum, antrum, incisura, body, fundus: unrevealing  - Prednisone 20 mg daily resolved collagenous gastritis both endoscopically and histologically   - Fifth EGD (3 months after stopping prednisone):   - Recurrent collagenous gastritis  - Antrum and fundus: normal  - Body: chronic inactive gastritis with 5-10 eosinophils/HPF and thickened basement membrane   - Follow-up EGDs (4 and 6 years after initial presentation):   - Resolution of collagenous gastritis  - No intestinal metaplasia  - On indefinite pantoprazole without steroids | - Initial presentation:   - Several months of intermittent epigastric discomfort  - 3 days of melena with microcytic anemia   - Response to initial treatment:   - Marked improvement with pantoprazole and iron supplementation after 1 month  - Symptom recurrence 1 month after stopping pantoprazole   - Disease evolution:   - Initial histology suggested mild collagen deposition  - Subsequent biopsies showed eosinophilic gastritis  - Later confirmed as collagenous gastritis  - Late onset of collagenous thickening (reason unclear)   - Recurrent bleeding:   - Readmitted 6 months later with 3 days of melena  - Found to have gastric ulcer   - Treatment response:   - Complete endoscopic and histologic resolution with prednisone after 1 month  - Recurrence 3 months after stopping prednisone  - Long-term resolution with indefinite PPI therapy alone (no steroids needed)  - Resolution maintained at 4 and 6 years |
| Ref(57) | 20 | Male (Caucasian man) | Denied abdominal pain | - Daily vomiting for 2 months - No antecedent travel or sick contacts | Denied change in bowel movements | Complete blood count normal (no anemia) | Not reported | - History of autism - Denied NSAID or drug use | - History of autism (neurodevelopmental disorder, not autoimmune) - No autoimmune diseases | Nodular gastric body | Not specifically mentioned | Not specifically mentioned | CT scan showed mesenteric adenitis | No evidence of *H. pylori* on histopathology | - Patchy thickening of surface basement membrane - >10 µm thick | - No collagenous colitis - Article focuses on collagenous gastritis without enteritis or colitis | Gastric body biopsies:   - Patchy thickening of surface basement membrane - Increases in eosinophils - Chronic inflammation - No evidence of *H. pylori* - Consistent with collagenous gastritis | - Induced into remission with prednisone 20 mg daily tapered over 2 months - Maintained in clinical remission using omeprazole 20 mg daily | - Maintained in clinical remission on omeprazole - Duration of follow-up not specified | - No histological follow-up data reported - Clinical remission maintained | - Presentation:   - Daily vomiting for 2 months  - No identified trigger  - Normal metabolic panel and CBC  - CT showed mesenteric adenitis   - Treatment response:   - Successfully induced into remission with prednisone tapered over 2 months  - Maintained in clinical remission with omeprazole |
| Ref(57) | 19 | Female (mixed Japanese/Caucasian ancestry) | - 3 episodes of diffuse post-prandial abdominal cramping lasting up to a week - Each episode started after breakfast and lasted 2-3 hours | Two episodes of emesis over 3 months | - Increased bowel frequency (up to 4 bowel movements per day) during episodes - Normalized as episodes subsided | Complete blood count normal without evidence of iron deficiency | Not reported | - Weight loss: 5 lbs - Denied recent travel, NSAID or alcohol use | Behçet's disease complicated by:  - Uveitis  - Glaucoma  - Cataracts  - Oligoarticular juvenile idiopathic arthritis  - Aphthous ulcers of terminal ileum and cecum consistent with Behçet's disease | Endoscopically normal EGD | Not specifically mentioned | Not specifically mentioned | - Colonoscopy: aphthous ulcers of terminal ileum and cecum (Behçet's disease) - Terminal ileum intubated | Not reported | - Prominent thickening of subepithelial collagen layer - >10 µm thick | - No collagenous colitis - Article focuses on collagenous gastritis without enteritis or colitis | - Random gastric body biopsies:   - Focal collagenous gastritis  - Prominent thickening of subepithelial collagen layer  - Increased lymphoplasmacytic inflammatory infiltrate  - Admixed eosinophils in lamina propria  - Entrapment of some inflammatory cells within collagen layer  - Scattered eosinophils in lamina propria, some entrapped in collagen layer   - General histological features:   - Irregular deposition of collagen >10 µm thick in subepithelium  - Entrapping dilated capillaries and inflammatory cells  - Eosinophilic infiltrate (prominent in all cases)  - Chronic inflammation  - Type III collagen (repair type) presence mentioned in literature  - Eosinophil degranulation could provoke tissue damage and fibroblast proliferation | - No PPI therapy initiated (absence of upper GI symptoms) - Treated with adalimumab and azathioprine for Behçet's disease - GI symptoms resolved following initiation of adalimumab | - GI symptoms resolved following initiation of adalimumab - Duration of follow-up not specified | - No histological follow-up data reported - Clinical symptom resolution | - Presentation:   - 3 episodes of diffuse post-prandial abdominal cramping  - Episodes lasted up to a week  - Intermittent increased bowel frequency  - 5 lbs weight loss  - Known autoimmune disease (Behçet's disease, JIA)   - Diagnostic findings:   - Elevated fecal calprotectin (328 µg/g)  - Normal CBC without iron deficiency  - Endoscopically normal stomach but focal collagenous gastritis on random biopsies  - Aphthous ulcers in terminal ileum and cecum (Behçet's)   - Treatment response:   - GI symptoms resolved following initiation of adalimumab (for Behçet's disease)  - No PPI therapy needed |
| Ref(58) | - 20 years old at initial presentation with autoimmune encephalitis - Approximately 21-22 years old when gastrointestinal symptoms developed (18 months after initial presentation) | Female (woman) | Not specifically mentioned | Not specifically mentioned | - Watery, non-bloody diarrhea (developed 18 months after initial presentation) - 6 to 8 bowel movements per day | - Not reported - Full blood cell count was normal at initial presentation | Not reported | - Initial neurological presentation:   - Generalized motor seizures  - 1 week of fever, coryza, and headache preceding seizures  - Required multiple anticonvulsant agents and intubation  - Depression (treated with sertraline 50 mg daily)   - Gastrointestinal symptoms (at 18 months):   - Watery, non-bloody diarrhea  - 6-8 bowel movements per day  - Symptoms responded to treatment   - No history of recurrent infection despite immunodeficiency | - Personal autoimmune/immune disorders:   - Common Variable Immunodeficiency (CVID) - primary diagnosis  - Autoimmune encephalitis (AE) - initial presentation  - Depression (treated with sertraline)   - Immunological findings:   -Panhypogammaglobulinemia:   1. IgG: 2.99 g/L (reference 6.72-14.40) 2. IgA: 0.11 g/L (reference 0.69-3.37) 3. IgM: 0.33 g/L (reference 0.48-2.38)   - Lymphocyte subsets:   1. Total lymphocyte count: 752 cells/µL 2. CD4^+^ T cells: 332 cells/µL 3. CD8^+^ T cells: 208 cells/µL 4. CD19^+^ B cells: 132 cells/µL 5. CD16^+^/56^+^ NK cells: 45 cells/µL 6. Low proportion of class-switched memory B cells (CD19^+^CD27^+^IgM^−^IgD^−^ 0.39% of total lymphocytes)   - CVID classifications:   1. EuroClass: B^+^/smB^+^/CD21-N/Tr-lo 2. Paris phenotype: MB1 3. Freiburg phenotype: 1b 4. Isohemagglutinins: undetectable   - Functional antibody responses:   1. Impaired response to 23-valent antipneumococcal vaccine 2. Total antipneumococcal IgG: 62 mg/mL pre-vaccine, 106 mg/mL post-vaccine (normal >2-fold increase and >110 mg/mL post-vaccination) 3. Pre- and post-tetanus vaccine antibodies: 1.38 and 1.28 IU/mL   - Autoantibodies tested (all negative):   1. Antineuronal and limbic encephalitis antibodies (anti-NMDAR, anti-LGI1, anti-GABA-B, anti-DPPX, anti-IgLON-5, anti-PCA, anti-sox-1, anti-CRMP-5, anti-Ma, anti-Tr) 2. Antinuclear antibody 3. Extractable nuclear antigens 4. Antineutrophil cytoplasmic antibody 5. Celiac disease serology: negative   - Complement levels:  C3/C4: normal  - Genetic findings:   1. 2 heterozygous variants of unknown significance detected by massively parallel sequencing 2. SAMD9L gene: c3778T>G missense variant (SIFT score 0.15) 3. PGM3 gene: c.1354C>A missense variant (SIFT score 0.36, allele frequency 0.06%)   - Family history:  No family history of autoimmune disease or immunodeficiency | Not mentioned | Not mentioned | Not mentioned | - Moderate ulcerative gastritis - Most severe in fundus - Relative sparing of antrum | Not reported | - Significant thickening of subepithelial collagen plate - >10 µm (normal 0.3-2.8 µm) - Exact measurement not specified | - Subepithelial collagen deposition in cecal biopsy - Exact measurement not specified - Consistent with collagenous colitis | - Gastric biopsy:   - Basal lymphoplasmocytic infiltrate  - Significant thickening of subepithelial collagen plate (>10 µm; normal 0.3-2.8 µm)  - Consistent with collagenous gastritis  - No evidence of lymphoma   - Cecal biopsy:   - Subepithelial collagen deposition  - No intraepithelial lymphocytes  - Consistent with collagenous colitis   - Notable absence of typical CVID enteropathy features:   - No intraepithelial lymphocytosis  - No villous atrophy  - No nonspecific inflammation  - No nodular lymphoid hyperplasia  - Patient had subepithelial collagen deposition without typical histologic features of CVID enteropathy   - Other investigations:   - Stool microscopy and culture: no pathogenic organism detected  - No evidence of lymphoma   - Inflammatory markers:   - Fecal calprotectin: elevated at 141 mg/g (reference <50)  - Fecal alpha-1 antitrypsin: elevated at 2.8 mg/g (reference <1.0) - suggestive of intestinal inflammation  - C-reactive protein: mildly elevated at 20 mg/L (reference <5) at initial presentation  - 24-hour urine protein: normal at initial presentation  - Fecal alpha-1 antitrypsin: normal at initial presentation | - For Autoimmune Encephalitis:   - Multiple anticonvulsant agents: midazolam, levetiracetam, phenytoin, sodium valproate  - Intubation required  - Methylprednisolone 1 g daily for 3 days  - Intravenous immunoglobulin (IVIG) 0.5 g/kg daily for 5 days  - IVIG continued monthly for 12 months  - Empirical antimicrobial therapy (discontinued after 7 days - no infection detected)   - For CVID:   - Continuing antibody replacement immunoglobulin commenced  - Subcutaneous immunoglobulin preparation (patient's choice)   - For Collagenous Gastritis and Colitis:   - Mesalazine  - Budesonide  - Continued immunoglobulin replacement   - Concurrent medication:   Sertraline 50 mg daily for depression | - 3 months post-treatment for gastrointestinal symptoms - 18 months from initial presentation when GI symptoms developed | Not mentioned | - Initial presentation with autoimmune encephalitis at age 20 - CVID diagnosed during workup (panhypogammaglobulinemia, low class-switched memory B cells, impaired functional antibody responses) - GI symptoms developed 18 months after initial presentation - Elevated fecal calprotectin (141 mg/g) and fecal alpha-1 antitrypsin (2.8 mg/g) indicating intestinal inflammation - Symptoms responded to mesalazine and budesonide - Normalization of fecal A1AT (0.3 mg/g) by 3 months - Two heterozygous variants of unknown significance detected: SAMD9L (c3778T>G) and PGM3 (c.1354C>A) |
| Ref(59) | - Mean: 11.3 ± 3.7 years - Range: 2-16 years | - Female: 57.5% (23/40) - Male: 42.5% (17/40) | 77.5% (31/40) | - Vomiting: 65% (26/40) - Nausea: 52.5% (21/40) - Hematemesis: 2/26 of those with vomiting | 32.5% (13/40) | - 57.5% (23/40) at diagnosis - 75% (30/40) during follow-up - Mean Hgb: 7.1 ± 2.3 g/dL (median 6.3, range 3.7-10.9) - Severe anemia (Hgb ≤7): 56.5% (13/23) - Symptomatic anemia: 82.6% (19/23) | Not specifically mentioned | - Anorexia: 25% (10/40) - Weight loss: 25% (10/40) - GI bleed: 22.5% (9/40) - hematochezia (7/9), melena (2/9) - Poor growth: 20% (8/40) - Poor weight gain: 12.5% (5/40) - Heartburn: 12.5% (5/40) - Constipation: 10% (4/40) - Abdominal distension: 7.5% (3/40) - Dysphagia: 5% (2/40) | - Autoimmune comorbidities: 12.5% (5/40)   - Celiac disease (n=2)  - Inflammatory arthropathy (n=2)  - Crohn disease (n=1)  - Erythema nodosum (n=1)  - Psoriasis (n=2)   - Family history of autoimmunity: 30% (12/40) - Atopy: 25% (10/40) - Immune deficiency: 5% (3/40) - IgA deficiency | - Any nodularity: 77.5% (31/40) - Diffuse: 45% (18/40) - Corpus only: 27.5% (11/40) - Antrum only: 2.5% (1/40) | Not specifically quantified | - Erosions and/or superficial ulcerations: 10% (4/40) - Ulcers: 7.5% (3/40) | - Visible blood/blood clots: 20% (8/40) - Polyps: 2.5% (1/40) - Aphthae: 2.5% (1/40) | 0% (0/40) - All negative by immunohistochemistry | ≥10 μm (diagnostic criterion: thickened subepithelial collagen layer ≥10μm in at least 1 focus of 3 contiguous gastric inter-pit spaces) | - Collagenous colitis: 7.5% (3/40) at diagnosis - Additional CC developed: 1 patient later in course - Collagenous duodenitis: 17.5% (7/40) at diagnosis, 2 more on subsequent EGDs - Collagenous ileitis: 2.5% (1/40) | - All patients (100%): increased subepithelial collagen deposition - Mixed inflammatory infiltrate in lamina propria - Prior EGDs (in 13 patients before diagnosis):   - Chronic inactive gastritis: 76.9% (10/13)  - Chronic mildly active gastritis: 7.7% (1/13)  - Increased lamina propria eosinophils: 38.5% (5/13)  - Focal abnormal intraepithelial migration of eosinophils: 15.4% (2/13) | - No significant association of clinical or histologic improvement with specific treatments - Iron supplementation: effective for all patients with anemia - Most common treatments used:   - PPI or H2 blocker: 40% at last follow-up  - Laxatives: 20%  - Cyproheptadine: 12.5%  - Antiemetics: 12.5%  - Budesonide: 7.5%  - 6-Mercaptopurine/Infliximab: 5%  - Dietary allergen restriction: used in some patients  - Oral steroids: used in some patients   - Mean elemental iron dose: 1.5 ± 0.64 mg/kg/day (median 1.3, range 0.7-2.5) | - Mean follow-up: 2.9 ± 2.3 years (34.8 ± 27.0 months) - Median: 2.6 years - Range: 3 weeks to 10.6 years - Patients with at least 1 follow-up EGD: 65% (26/40) - Mean follow-up EGDs: 2.2 ± 1.5 (median 3.5, range 1-6) - Time from diagnosis to last EGD: 2.9 ± 2.1 years (median 2.9, range 0.3-8.3) | Persistent abnormal subepithelial collagen: 73.1% (19/26)  Resolution of collagen: 26.9% (7/26)  Time to EGD showing collagen resolution: 2.4 ± 1.6 years (median 2.4, range 0.4-4.4)  Persistent endoscopic abnormality at last EGD: 92.3% (24/26)   - Nodularity: 76.9% (20/26) - Granularity: 7.7% (2/26) - Furrowing: 3.8% (1/26) | - Improved or resolved symptoms: 87.5% (35/40)   - Improvement: 45% (18/40)  - Complete resolution: 37.5% (15/40)  - Remained asymptomatic: 5% (2/40)   - No improvement: 12.5% (5/40) - Anemia outcome:   - Resolution with iron supplementation: 100% of patients with anemia  - No anemia on ongoing iron: 55% (22/40)  - No anemia, off iron ≥6 months: 12.5% (5/40)  - Recurrence after stopping iron: 10% (4/40)  - Time to iron discontinuation: 34.0 ± 17.9 months (range 6 months-5.2 years)   - Residual symptoms in improved patients:   - Intermittent abdominal pain: 72.2% (13/18)  - Nausea: 44.4% (8/18)  - Vomiting: 22.2% (4/18)   - Active medications at last follow-up: 57.5% (23/40) - Clinical improvement despite persistent histologic findings in most cases - Longest follow-up: 10.6 years with ongoing symptoms and persistent collagen deposition |
| Ref(60) | - 15 years old at initial presentation - 11 years old at initial iron deficiency anemia diagnosis (4 years before collagenous gastritis diagnosis) - 16 years old at diagnosis of collagenous gastroduodenocolitis | Male | - Chronic abdominal pain for one year - Mainly epigastric - Rapidly aggravated during fasting - Intermittent abdominal pain persisted after *H. pylori* eradication | Not specifically mentioned as presenting symptoms | - Unformed stool for one month at initial visit - Diarrhea newly developed one year after initial visit | - Iron deficiency anemia present at age 11 (4 years before collagenous gastroduodenocolitis diagnosis) - Initial hemoglobin: 7.1 g/dL (at age 11) - Hemoglobin at first visit: 9.8 g/dL - Hemoglobin at collagenous gastroduodenocolitis diagnosis: 9.3 g/dL - Iron: 10-12 μg/dL - Ferritin: 2-5 ng/mL - Total iron binding capacity: 352-448 μg/dL - Transferrin saturation: 2.7-3.1% - Anemia did not improve despite continuous oral iron supplementation | Not mentioned | - Weight loss: from 49.6 to 47.6 kg over 2 weeks - Fecal immunochemistry test positive initially (357 ng/mL) - Fecal calprotectin: 573 mg/kg initially, 331 mg/kg at collagenous gastroduodenocolitis diagnosis | - Serum immunological tests all negative:   - Antinuclear antibody: negative  - Anti-smooth muscle antibody: negative  - Antibodies to autoimmune diseases commonly observed in adult-type collagenous gastritis: all negative   - No autoimmune comorbidities reported - No family history of autoimmune diseases mentioned | - Diffuse mucosal nodularity in stomach - Diffuse nodular hypertrophic mucosa extended through pylorus to duodenal bulb and second portion - Nodularity persisted on follow-up endoscopies | Mucosal erythema on follow-up esophagogastroduodenoscopy | Erosions observed on follow-up esophagogastroduodenoscopy | - Multiple large ulcers at anterior portion of cardia (initial esophagogastroduodenoscopy) - Active ulcers and scars (second esophagogastroduodenoscopy) - Improvement of ulcers and scars on follow-up esophagogastroduodenoscopy at 1 year - Colonoscopy: grossly normal at all time points (initial, diagnosis, and follow-ups) | - Campylobacter-like organism test: positive at initial esophagogastroduodenoscopy - Histology: did not reveal *H. pylori* organisms initially - Campylobacter-like organism test: negative at second esophagogastroduodenoscopy (after eradication treatment) - Urea breath test: negative 6 weeks after eradication treatment - Suspected *H. pylori* gastritis treated with triple therapy (lansoprazole, amoxicillin, clarithromycin for 2 weeks) | 60 μm in stomach antrum, body, and pylorus | 30 μm in all colonic segments (cecum, ascending, transverse, descending, sigmoid colon) | - Initial biopsies (before collagenous gastroduodenocolitis diagnosis): chronic inflammation only, no thickened subepithelial collagenous bands detected - At collagenous gastroduodenocolitis diagnosis:   - Irregularly thickened subepithelial collagenous bands  - Strongly stained with Masson-trichrome  - Negative for amyloid with Congo red stain  - Chronic inflammation in lamina propria   - Masson-trichrome stain retrospectively conducted on initial biopsies: no subepithelial collagenous bands detected - Follow-up histology: persistent collagen deposits with chronic inflammation in stomach, duodenum, and colon | - Triple therapy for *H. pylori* eradication: lansoprazole, amoxicillin, clarithromycin for 2 weeks (initially) - Lansoprazole: continued for at least 3 years - Oral iron supplementation: continuous throughout follow-up - Oral prednisolone: attempted between 2nd and 3rd year follow-up for 2 months   - Not effective for iron deficiency anemia   - No adverse events during eradication treatment | - Total follow-up: 3.5 years from collagenous gastroduodenocolitis diagnosis - Follow-up esophagogastroduodenoscopy at 1 year after collagenous gastroduodenocolitis diagnosis - Second follow-up at 2 years - Third follow-up at 3 years - Serial laboratory monitoring showing persistent iron deficiency anemia despite treatment | - One year after collagenous gastroduodenocolitis diagnosis: persistent collagen deposits with chronic inflammation in stomach, duodenum, and colon - Second and third year follow-up: no significant changes in histologic findings - Endoscopic findings at 1-year follow-up:   - Improvement of previously observed active ulcers and scars  - Continuous diffuse nodularity, mucosal erythema and erosions  - Colonoscopy repeatedly showed no endoscopic abnormality | - Initial symptoms: chronic abdominal pain, weight loss, unformed stool, iron deficiency anemia - After *H. pylori* eradication:   - Weight recovered within 2 weeks  - Intermittent abdominal pain persisted  - Diarrhea newly developed 1 year later  - Iron deficiency anemia did not improve despite successful eradication and continuous iron supplementation   - At 3.5 years follow-up:   - No symptoms of abdominal pain and diarrhea  - Iron deficiency anemia has not shown remarkable improvement despite continuous lansoprazole and oral iron supplementation  - Laboratory at 3 years: hemoglobin 10.3 g/dL, iron 16 μg/dL, ferritin 4 ng/mL, transferrin saturation 4%   - Treatment with systemic corticosteroids (prednisolone for 2 months) was not effective - Clinical improvement of gastrointestinal symptoms but persistent iron deficiency anemia and histologic findings |
| Ref(61) | - Mean age: 54.6 ± 26.3 years - Bimodal age distribution:   - First peak: 10-19 years old  - Second peak: primarily females aged ≥60 years   - Highest prevalence: 10-19 years age group (for both genders) - Young patients (10-29 years): slightly higher proportion - Most patients: 50-79 years | - Female: 73.2% (123/168) - Male: 26.2% (44/168) - Female predominance: odds ratio 1.69 (95% confidence interval: 1.20-2.39) - Second age peak primarily in females aged ≥60 years | - 24.4% (41/168) - Odds ratio: 0.84 (95% confidence interval: 0.59-1.19) - Not significantly different from controls | - Nausea: 8.3% (14/168), odds ratio 1.67 (0.97-2.89) - Vomiting: 10.1% (17/168), odds ratio 1.84 (1.11-3.03, statistically significant) | - 17.9% (30/168) - Odds ratio: 2.79 (1.88-4.15, statistically significant) - In patients with celiac disease: 29% (2/7) had diarrhea - In patients with duodenal lymphocytosis: 32% (6/19) had diarrhea - In patients without celiac disease or lymphocytosis: 15.5% (22/142) had diarrhea - Diarrhea associated with comorbid celiac sprue and collagenous colitis | - 11.9% (20/168) - Odds ratio: 1.70 (1.06-2.71, statistically significant) | Not mentioned | - Weight loss: 10.7% (18/168), odds ratio 2.43 (1.49-3.96, statistically significant) - Gastroesophageal reflux disease symptoms: 28.6% (48/168), odds ratio 0.44 (0.31-0.61, less common than controls) - Dysphagia: 12.5% (21/168), odds ratio 0.76 (0.48-1.20) - Dyspepsia: 8.3% (14/168), odds ratio 1.01 (0.59-1.75) | - Autoimmune gastritis: 7.1% (12/168)   - Odds ratio: 10.9 (6.05-19.6, highly significant)   - Celiac disease: 4.2% (7/168)   - Odds ratio: 15.9 (7.46-34.0, highly significant)   - Lymphocytic gastritis: 4.2% (7/168)   - Odds ratio: 23.2 (10.9-49.5, highly significant)   - Duodenal intraepithelial lymphocytosis: 11.3% (19/168)   - Odds ratio: 3.71 (2.30-5.98, highly significant)   - Significantly associated with lymphocytic disorders of upper gastrointestinal tract - Possible common pathogenetic mechanisms with these conditions | Not specifically quantified in this study | Not specifically quantified in this study | Not specifically quantified in this study | - Study focused on histopathology rather than endoscopic findings - Endoscopic procedure records available in most cases but specific findings not detailed | - *H. pylori* positive chronic active gastritis: 0.6% (1/168)   - Odds ratio: 0.05 (0.01-0.38, significantly less frequent)   - *H. pylori* negative chronic active gastritis: 1.2% (2/168)   - Odds ratio: 1.01 (0.25-4.08)   - *H. pylori* infection significantly less frequently associated with collagenous gastritis | - Diagnostic criterion: continuous subepithelial collagen band ≥10 μm thickness - In most cases: 40-100 μm - Associated inflammatory infiltrate in lamina propria required | - Not specifically measured in this study - Study focused on gastric collagenous gastritis | - All 168 patients: thick band of collagen immediately beneath surface epithelial cells - Collagen does not extend to deeper portions of lamina propria - Background of chronic, sometimes active gastritis - Trichrome-Masson stain performed in 75 cases for confirmation - Retrospective review of 60 biopsy specimens: confirmed original diagnosis in all cases - Diagnostic criteria: Colletti and Trainer 1989 criteria applied - Associated histopathological findings:   - Chronic inactive gastritis: 5.4% (9/168), odds ratio 1.10 (0.56-2.16)  - Reactive gastropathy: 6.0% (10/168), odds ratio 0.25 (0.13-0.47, significantly less frequent)  - Intestinal metaplasia: 9.5% (16/168), odds ratio 1.11 (0.66-1.85)  - Fundic gland hyperplasia: 1.2% (2/168), odds ratio 0.14 (0.03-0.57, significantly less frequent)  - Eosinophilic esophagitis: 1.2% (2/168), odds ratio 1.05 (0.26-4.25, not associated)  - Barrett's metaplasia: 3.6% (6/168), odds ratio 0.88 (0.39-1.98) | - Not reported in this epidemiological study - Study focused on prevalence and associations rather than treatment | - Subgroup of 52 patients underwent more than one esophagogastroduodenoscopy - Average time interval between first and last esophagogastroduodenoscopy: 2.2 ± 1.9 years (range 0.2-6.9 years) - Number of consecutive esophagogastroduodenoscopies:   - 2 esophagogastroduodenoscopies: 39 patients  - 3 esophagogastroduodenoscopies: 8 patients  - 4 esophagogastroduodenoscopies: 4 patients  - 5 esophagogastroduodenoscopies: 1 patient   - Total: 123 esophagogastroduodenoscopies among 52 patients, leading to 71 transitions - Study period: 10 years (2009-2018) | - Total transitions analyzed: 71 transitions between consecutive histopathological evaluations - Transition patterns:   - Collagenous gastritis to collagenous gastritis (persistent): 24 transitions  - Collagenous gastritis to no collagenous gastritis (resolved): 18 transitions  - No collagenous gastritis to no collagenous gastritis: 8 transitions  - No collagenous gastritis to collagenous gastritis (new occurrence): 21 transitions   - Most common transitions: persistence of collagenous gastritis or first occurrence - Collagenous gastritis observed at last esophagogastroduodenoscopy: 69% (33/52 patients with multiple esophagogastroduodenoscopies) - Permanence of collagenous gastritis in majority of patients - Collagenous gastritis remained permanent feature in 69% of patients (similar to 75-79% reported in other series) | - Prevalence of collagenous gastritis: 13 per 100,000 esophagogastroduodenoscopies - Rare condition confirmed - Bimodal age distribution suggests different etiologies in children versus adults - In adults: underlying immune disease affecting duodenum (celiac disease) or colon (microscopic colitis) may also involve stomach - In children: additional mechanisms such as viral infections or environmental risks may be primary contributing factors - Common symptoms: diarrhea, anemia, weight loss, vomiting - Strong association with lymphocytic disorders of upper gastrointestinal tract suggests possible common pathogenetic mechanisms - Natural history: collagenous gastritis tends to persist in majority of patients (69% at last follow-up) - Clinical presentation influenced by comorbid conditions (celiac disease, collagenous colitis) |
| Ref(62) | - 11 years old at presentation - 12 years old at final diagnosis with collagenous gastritis | Male | Intermittent abdominal pain with cramping | - Occasional nausea - No vomiting mentioned | Not mentioned | - Severe iron deficiency anemia at presentation - Hemoglobin: 4.1 g/dL (severe anemia) - Mean corpuscular volume: 62 fL (microcytic) - Absolute reticulocyte count: 74.8 thou/mL - Ferritin: 1.9 ng/mL - Anemia returned after cessation of oral iron - Recurrence of anemia 2 months after initial treatment | Not mentioned | - Increasing fatigue for 3 months - Pallor - No evidence of gastrointestinal bleeding (fecal occult blood negative) | - Celiac disease testing:   - Initial testing: borderline antiendomysial antibodies, normal tissue transglutaminase  - Repeat testing: normal  - Histology: no evidence of celiac disease   - No other autoimmune comorbidities reported - No family history mentioned | - Initial esophagogastroduodenoscopy: visually normal (no nodularity) - Second endoscopy (2 months later): marked nodularity of stomach - Third endoscopy (after 3 months wheat elimination): nodularity with diffuse friability and granularity in gastric fundus, body, and antrum - Evolution from normal-appearing stomach to marked nodularity over time | Not specifically mentioned | Not mentioned | - Diffuse friability - Granularity in gastric fundus, body, and antrum - Edema of gastric mucosa with distinct granularity - Histologically demonstrates endoscopic evolution of disease over time | No evidence of *H. pylori* on initial esophagogastroduodenoscopy histology | - First biopsy (retrospectively with trichrome stain):   - Maximum subepithelial collagen thickness: 34 μm  - 5% of total tissue surface with subepithelial collagen bands >10 μm  - Very minimal subepithelial deposition on hematoxylin and eosin, not impressive enough to initially suggest trichrome staining   - Second biopsy (retrospectively with trichrome stain): collagen deposition confirmed (originally diagnosed as eosinophilic gastritis with 50 eosinophils per high power field) - Third biopsy (first diagnosis of collagenous gastritis):   - Patchy subepithelial collagen deposition up to 100 μm in thickness  - 70-90% of tissue surface involved   - Fourth biopsy (after budesonide trial):   - Continued subepithelial collagen deposition: 65 μm  - Collagen deposition ranging from 60-100 μm on subsequent biopsies   - Progressive increase in collagen deposition over time | Terminal ileum and colon: histologically normal | - First biopsy:   - Mild chronic active gastritis only on hematoxylin and eosin stain  - No evidence of intestinal pathology, celiac disease, or *H. pylori*  - Retrospective trichrome staining revealed focal/minor collagen deposition (34 μm maximum)   - Second biopsy:   - Patchy increase in lamina propria eosinophils (up to 40 per high power field)  - Focal eosinophilic microabscesses  - Mild chronic inflammation with lymphoid follicle  - Retrospective trichrome staining confirmed collagen deposition   - Third biopsy:   - Chronically inflamed antral and oxyntic type gastric mucosa  - Moderate eosinophilia (65 per high power field)  - Patchy subepithelial collagen deposition (up to 100 μm) confirmed by Gomori trichrome stain   - Fourth biopsy (after budesonide):   - Continued subepithelial collagen deposition (65 μm)  - Mucosal eosinophilia (65 per high power field)   - Histologic evolution: inactive gastritis → eosinophilia → marked collagen deposition - Demonstrates importance of trichrome staining for diagnosis | - Initial treatment for iron deficiency anemia:   - 10 cc/kg packed red blood cell transfusion  - Ferrous sulfate 325 mg twice daily for 3 months  - Instruction to decrease milk consumption   - Dietary intervention:   - Stopped wheat consumption for 3 months (patient's choice instead of prednisone)  - No improvement   - Topical budesonide: trial attempted   - No improvement in collagen deposition or eosinophilia   - Current management:   - Oral iron supplementation (ongoing)  - No food elimination from diet  - Management focused on anemia, growth development, and potential long-term effects of collagenous gastritis   - Prednisone was offered but patient declined in favor of wheat elimination trial | - Initial presentation to final diagnosis: approximately 8-9 months - Timeline:   - 3 months of symptoms before presentation  - Initial treatment with iron for 3 months  - 2 months later: second endoscopy  - 3 months wheat elimination trial  - Third endoscopy with collagenous gastritis diagnosis  - Fourth endoscopy after budesonide trial   - Patient remains under care for management - Ongoing monitoring for anemia, growth development, and potential long-term effects | - Progressive increase in collagen deposition over time:   - First biopsy: 34 μm maximum (5% of tissue surface)  - Second biopsy: collagen present on retrospective staining  - Third biopsy: 60-100 μm (70-90% of tissue surface)  - Fourth biopsy: 65 μm (persistent after budesonide trial)   - Persistent mucosal eosinophilia throughout follow-up (65 per high power field) - Collagen deposition confirmed on all biopsies with Gomori trichrome stain retrospectively - Demonstrates histologic evolution and progression of disease - Patchy nature of early collagen deposition highlighted - Increasing collagen deposition correlates with endoscopic progression from normal to marked nodularity | - Initial presentation: severe iron deficiency anemia (hemoglobin 4.1 g/dL), fatigue, pallor, intermittent abdominal pain - Initially presumed diagnosis: iron deficiency anemia due to excess milk intake - Anemia returned after cessation of oral iron - Initially diagnosed as eosinophilic gastritis based on histology - Dietary wheat elimination trial for 3 months: no improvement - Final diagnosis: collagenous gastritis (initially masquerading as eosinophilic gastritis) - Key diagnostic evolution:   - First endoscopy: visually normal, mild chronic active gastritis  - Second endoscopy: marked nodularity, eosinophilic gastritis suspected  - Third endoscopy: nodularity with friability, collagenous gastritis diagnosed   - Highlights importance of trichrome staining in persistent eosinophilic gastritis or unexplained iron deficiency anemia - Current status: remains under care, dependent on oral iron supplementation - No definitive treatment available; management focuses on treating ongoing blood loss - Case illustrates natural history of collagenous gastritis with progressive collagen deposition and endoscopic changes - Emphasizes need for broad differential diagnosis including drug reaction, allergy, infection, and collagenous gastritis when eosinophilic inflammation is present |
| Ref(63) | 66 | Female | - Recurrent moderate cramp-like abdominal pain - Mild epigastric tenderness on abdominal palpation | - Periodic nausea - No vomiting mentioned | Non-bloody diarrhea | Mild anemia | Not mentioned | - Fatigue - Increased fecal calprotectin levels - Increased C-reactive protein levels - Medical history of terminal ileitis for 2 years - Improved symptoms after 4 months of treatment with mesalazine and methylprednisolone (9 mg daily) - Intramucosal ruptures and hemorrhage after biopsy (no perforation) | - No autoimmune diseases mentioned - Human immunodeficiency virus-1,2 Western blots: negative (non-acquired immune deficiency syndrome/human immunodeficiency virus patient) - No mention of celiac disease or other autoimmune conditions - Terminal ileitis present but nature not specified | - Mucosal nodularity throughout gastric antrum - No abnormal capillary vessels observed | Not specifically mentioned | Not mentioned | - Atrophy in stomach body - Round, elevated maculopapular lesion with friability in stomach body (Kaposi's sarcoma) - Reddish appearance of lesion - Intramucosal ruptures and hemorrhage after biopsy - Findings observed in narrow-band imaging mode - Colonoscopy: unremarkable - Small bowel capsule endoscopy: unremarkable | Not detected | - Deposition of collagen bands > 10 μm thick in subepithelial layer - Exact thickness not specified beyond "> 10 μm" - Specimen positive for Masson's trichrome staining | - Chronic inflammatory cells infiltrated in subepithelial layer with collagen deposition - Exact thickness not specified - Diagnosis of microscopic colitis confirmed | - Spindle-cell proliferation in submucosa (Kaposi's sarcoma) - Immunohistochemistry:   - CD31: strong immunopositivity  - Human herpesvirus 8: strong positive staining   - Deposition of > 10 μm thick collagen bands in subepithelial layer - Positive Masson's trichrome staining - Atrophic changes | - Prior to diagnosis (for terminal ileitis):   - Mesalazine  - Methylprednisolone 9 mg daily for 4 months  - Improved symptoms with this treatment   - After diagnosis of gastric Kaposi's sarcoma with collagenous gastritis and microscopic colitis:   - Budesonide  - Esomeprazole  - Pegylated liposomal doxorubicin   - For intramucosal ruptures and hemorrhage after biopsy:   Hemostatic agents (showed positive effects) | - Not reported in this case report - No follow-up data provided | - Not reported - No follow-up histology described | - Initial presentation: recurrent moderate cramp-like abdominal pain, non-bloody diarrhea, periodic nausea, fatigue - Medical history: terminal ileitis for 2 years - Symptoms improved with mesalazine and methylprednisolone treatment for 4 months - Upper gastrointestinal endoscopy findings:   - Atrophy and maculopapular lesion in stomach body  - Mucosal nodularity in gastric antrum  - Intramucosal ruptures and hemorrhage after biopsy (treated successfully with hemostatic agents)   - Final diagnosis: isolated gastric Kaposi's sarcoma with coexisting collagenous gastritis and microscopic colitis - Physical examination: no skin rash, mild epigastric tenderness, nonpalpable liver and spleen - Laboratory: mild anemia, increased fecal calprotectin and C-reactive protein - Imaging (abdominal ultrasonography and contrast-enhanced computed tomography): unremarkable - Unique case: isolated gastric Kaposi's sarcoma in non-acquired immune deficiency syndrome patient with coexisting collagenous gastritis and microscopic colitis - first such case reported - Kaposi's sarcoma characteristics:   - Spindle-cell tumor from vascular endothelium  - Associated with human herpesvirus 8 infection  - Incidence: 1.58/100,000 persons/year in non-acquired immune deficiency syndrome patients  - Gastrointestinal involvement in 55% of cases with stomach affection  - Can occur without skin lesions  - Can be asymptomatic or cause gastrointestinal bleeding or diarrhea   - Collagenous gastritis characteristics noted:   - Rare condition  - Poorly understood etiology  - Approximately 60 cases reported in English literature at time of publication   - Treatment initiated with combination therapy for all three conditions |
| Ref(64) | - 53 years old at initial presentation - History of symptoms since teenage years (frequent acute sinusitis) - In his 30s: meningitis and multiple pneumonias - Collagenous gastritis diagnosed at age 53 - Gastric adenocarcinoma diagnosed 8 years later at age 61 | Male | - Epigastric burning - Severe upper abdominal pain | Not mentioned | Not mentioned | Not mentioned in this patient | Not mentioned | - Medical history:   - Asthma (well controlled on inhaled corticosteroid therapy)  - Allergic rhinitis (well controlled with allergen immunotherapy and nasal fluticasone spray)  - Recurrent upper respiratory tract infections  - Frequent acute sinusitis in teenage years  - Meningitis in his 30s  - Multiple pneumonias in his 30s   - No new symptoms at gastric adenocarcinoma diagnosis (detected on screening endoscopy) | - Primary Selective IgM Deficiency (first reported case of collagenous gastritis in this condition)   - Severely reduced IgM: 9 mg/dL (control 37-336)  - Normal IgG: 698 mg/dL (control 660-1,660)  - Normal IgA: 145 mg/dL (control 80-400)  - Normal response to pneumococcal polysaccharide, diphtheria, and tetanus toxoid  - CD3^+^, CD4^+^, CD8^+^, CD19^+^ B cells, and natural killer cells: normal  - Secondary causes of low IgM excluded  - Follow-up immunoglobulin levels: IgG 698-718 mg/dL, IgM <9-17 mg/dL   - Asthma (allergic condition) - Allergic rhinitis | Not specifically mentioned | Not mentioned | Not mentioned | - Initial endoscopy: collagenous gastritis findings - Annual screening endoscopies with random biopsies performed - 8 years later): new polyp detected - Polyp biopsy revealed poorly differentiated gastric adenocarcinoma - No specific endoscopic appearance details provided | Negative for *H. pylori* infection | - Thickened subepithelial collagen table present - Diagnostic features of entrapped cellular elements (inflammatory cells and blood vessels) - Specific thickness not quantified in this case report | Not examined in this patient (isolated collagenous gastritis) | - Initial biopsy (collagenous gastritis):   - Nonspecific chronic inflammatory infiltrate  - Thickened subepithelial collagen table  - Entrapped cellular elements including inflammatory cells and blood vessels  - Subepithelial collagen deposition with mononuclear cell infiltration in lamina propria   - 8 years later (gastric adenocarcinoma, 2020):   - Poorly differentiated adenocarcinoma with surface ulceration  - High-grade dysplasia  - EBV-positive by EBV-related encoded RNA in situ hybridization assay (EBER ish)  - 0/22 positive lymph nodes after total gastrectomy   - EBV serology:   - EBV-VCA IgM antibodies: undetected  - EBV-VCA IgG antibodies: 388 U/mL (control <22)  - EBNA-IgG antibodies: 55.2 U/mL (control <22)  - EBV-EA diffuse antibodies: <5 U/mL (control <11)  - EBV-PCR: negative (no viremia at diagnosis) | - For collagenous gastritis:   - Twice daily PPI  - H2 blocker therapy  - Relief of symptoms achieved   - For gastric adenocarcinoma:   - Total gastrectomy performed  - PET CT negative for metastatic lesions  - 0/22 positive lymph nodes  - No chemotherapy instituted  - Patient doing well clinically post-surgery   - For Selective IgM Deficiency:   - No specific immunoglobulin replacement mentioned  - Management of allergic conditions (inhaled corticosteroid, allergen immunotherapy, nasal fluticasone) | - Annual endoscopies with random biopsies for monitoring gastric condition - 8 years of follow-up from collagenous gastritis diagnosis (2017) to gastric adenocarcinoma diagnosis (2020) - Immunoglobulin levels frequently repeated on several occasions - Post-gastrectomy: patient doing well clinically - No evidence of metastatic disease on PET CT | - Progression from collagenous gastritis to EBV-positive gastric adenocarcinoma over 8 years - First documented case of collagenous gastritis transitioning to gastric cancer - Annual biopsies performed but specific histological changes during transition period not detailed - Final histology: poorly differentiated adenocarcinoma | - Initial symptoms (epigastric burning, severe upper abdominal pain) relieved with PPI and H2 blocker therapy - Monitored with annual endoscopies - Developed EBV^+^ gastric adenocarcinoma 8 years after collagenous gastritis diagnosis - PET CT negative for metastatic lesion - Underwent successful total gastrectomy - Post-operative course: doing well clinically with no chemotherapy needed |
| Ref(65) | - 13 years old (at time of presentation to pediatric gastroenterology) - First visit at age 11 - Two-year history of intractable iron deficiency anemia | Female | Minimal tenderness in upper abdomen at first visit; no abdominal pain complaints initially | No vomiting | No diarrhea | Iron deficiency anemia (primary presentation) - Hb 8.6 g/dL, later 9.7 g/dL | Not mentioned | No dysphagia, no melena; asymptomatic at follow-up despite persistent histopathological findings | - None identified - Negative celiac serology - Negative ANA, Tissue transglutaminase IgA, Anti-thyroid peroxidase antibodies - Normal serum levels of amyloid A and IgG, A, M | Gastric corpus-fundus and antral mucosa was nodular; nodular lesions in the greater curvature of the gastric body | Gastric mucosal erythema | Not mentioned | Gastric mucosal edema; hyperplastic rugae; duodenum and esophagus were normal; no gastrointestinal bleeding | - Negative (excluded by fecal *H. pylori* antigen testing) - Not detected in histopathological samples | Thick collagen bands, patches of thick collagen bands (>10 μm conventionally defined; specific measurement not provided) | Not evaluated (colonoscopy not performed) | - Normal epithelial mucosa - Subepithelial layer (especially lamina propria) characterized by infiltration of chronic inflammatory cells - Deposition of collagen in focal areas - Patches of thick collagen bands - Hyaline collagen deposition in subepithelial layer (strongly stained with Masson-trichrome) - Increased number of plasma cells, eosinophils, and lymphocytes in lamina propria - All gastric specimens (fundus, corpus, antrum) revealed same histopathologic changes - No *H. pylori* or granulomas detected | - Initial: Oral iron replacement therapy (4 mg/kg/day) for 6 months - no response - Parenteral iron replacement therapy - Hb increased to 12.5 g/dL - After diagnosis: High-dose PPI (2 mg/kg/day) and oral iron supplementation - Intermittent iron treatment planned for follow-up | - 6 weeks after treatment: Physical examination completely normal - Control endoscopic evaluation repeated - Long-term follow-up planned in outpatient clinic with intermittent iron treatment - Colonoscopy planned if lower gastrointestinal symptoms develop | - No tissue healing after 6 weeks of treatment - Similar histopathological findings persisted on repeat endoscopy - No endoscopic or histologic improvement | - Two-year history of intractable iron deficiency anemia before diagnosis - Initially no response to oral iron therapy - Response to parenteral iron (Hb increased to 12.5 g/dL) - Hb decreased again to 9.7 g/dL at age 13 - After collagenous gastritis diagnosis and treatment: Insufficient Hb rise despite oral iron supplementation and PPI - No complaints and normal physical examination at follow-up despite persistent histopathological findings - Chronic disease course |
| Ref(66) | - Mean age: 41.5 ± 22.8 years overall - Pediatric group (<19 years): n=14, mean age 13.8 ± 4.2 years - Young adults (19-50 years): n=24, mean age 31.4 ± 7.3 years - Older adults (>50 years): n=26, mean age 65.6 ± 10.9 years | - Female: 42 (66%) - Male: 22 (34%) - Female predominance across all age groups | 38 (59%) overall; pediatric 64%, young adults 75%, older adults 42% | 39 (61%) overall; similar across age groups | 26 (41%) overall; pediatric 29%, young adults 42%, older adults 46% | 22 (34%) overall; pediatric 64%, young adults 25%, older adults 27% | Not mentioned | Weight loss 27 (42%) overall; pediatric 7%, young adults 42%, older adults 62%; GI bleeding 13 (20%); hypoalbuminemia 7/31 (23%) | - • Autoimmune disease: 16 (25%) - • Celiac disease: 6 (9%) - • Autoimmune gastritis: 4 (9%) - • Tried gluten-free diet: 28 (45%) - • HLA-DQ genes (celiac permissive genes): 14/28 (50%) of those tested | Present in most patients (diffuse nodular appearance) | Erythematous mucosa in most patients | Not specifically mentioned | Friable mucosa; gastric mucosal bleeding in 7 (10%); 2 required hospitalization for endoscopic hemostatic therapy | - Positive: 1 (2%) - Negative or not mentioned: 63 (98%) | - >10 μm (defined as diagnostic criteria); trichrome stain positive in 16 (25%) patients tested - Most common sites: Antrum 15 (33%), fundus or body 10 (22%), 2 or more sites 24 patients, pangastric involvement 3 (5%) | 11 (32% of 34 with colon biopsies) showed collagenous colitis | - Infiltration of chronic inflammatory cells (lymphocytes, plasma cells, eosinophils) into subepithelial layer (lamina propria) - Collagen bands greater than 10 μm thick - Focal collagen deposition in most cases - Trichrome stain confirmed dense collagen bands (when performed) - Small bowel involvement: villous atrophy with increased intraepithelial lymphocytes 9 (14%), villous atrophy only 3 (5%), increased intraepithelial lymphocytes only 7 (11%) - Colon biopsies (n=34): collagenous colitis 11 (32%), lymphocytic colitis 3 (9%), normal 20 (59%) | - Drug removal: 10 (20%) responded after stopping offending drugs (angiotensin receptor blockers, nonsteroidal anti-inflammatory drugs, statins, antidepressants) - Immunosuppressive therapy (except topically targeted budesonide): 17 (34%) - Systemic corticosteroids: 12 patients - Regular budesonide (closed enteric capsule): 5 patients - Immunomodulating agents: 2 patients - Topically targeted budesonide: 28 patients total (13 as initial treatment, 15 after other therapies failed); formulations included open-capsule budesonide and Mayo Clinic-compounded budesonide - PPIs at diagnosis: 13 (26%) - Other therapies: azathioprine, nortriptyline, mirtazapine, pulmicort, gluten-free diet: 8 (16%) - Total parenteral nutrition requirement: 5 (8%) | - Clinical follow-up available: 50 (78%) patients - Median duration: 28 months (interquartile range 15.5-59.5) - Lost to follow-up: 14 (22%) - For topically targeted budesonide-treated patients (n=28): median follow-up 27.5 months (interquartile range 16-58.5) - Median topically targeted budesonide treatment duration: 9 months (interquartile range 4-29) for responders | - Follow-up biopsies available: 15 (54% of topically targeted budesonide-treated patients) - Histologic response to topically targeted budesonide: 13/15 (87%); complete response 8 (53%), partial response 5 (33%), no response 2 (13%) - Among those with failed previous immunosuppressive therapies: histologic response 5/6 (83%) | - Clinical response to topically targeted budesonide: 25/28 (89%); complete response 11 (42%), partial response 12 (46%), no response 3 (11%) - Previous immunosuppressive therapy failure: 15 patients, of which topically targeted budesonide showed clinical response in 13/15 (87%) - Topically targeted budesonide dependency: 18/24 (75%) became dependent; 6/24 (25%) tapered off successfully - Among those who tapered off (n=6): 4 (67%) stable with no recurrence, 2 (33%) had relapse or mild persistent symptoms - Among topically targeted budesonide-dependent patients (n=18): 15 stable with no symptom recurrence, 3 had mild persistent symptoms - No adverse events or systemic toxicity reported with topically targeted budesonide - One non-responder to topically targeted budesonide received infliximab with complete clinical response - Concomitant collagenous sprue or collagenous colitis: 22 (35%) overall; more common in greater than 50 years (83%) vs 19-50 years (27%) or less than 19 years (50%) |
| Ref(67) | 31 | Male | Chronic dyspepsia | Not mentioned | Not specifically mentioned as a presenting symptom | Mild anemia (hemoglobin level 11.2 g/dL) | Not mentioned | Weight loss, thinness, anorexia | - None identified - Celiac sprue serology negative (anti-tissue Transglutaminase and anti-Endomysial antibodies were negative) - No evidence of celiac disease in duodenal specimens - No history of lymphocytic gastritis, Sjogren syndrome, or other autoimmune diseases - No other medical history reported | Erosions with nodularity, particularly in the body and antrum | Not specifically mentioned | Gastritis and erosions with nodularity | Narrowing, probable gastric atrophy zones, polyp-shaped lesions, scattered mucosal atrophy; colonoscopy revealed fragile mucosa | Negative (no *H. pylori* infection detected in histologic evaluation) | Sub-epithelial collagen deposition present (specific measurement not provided); confirmed by hematoxylin and eosin staining and specific collagen trichrome staining | Sub-epithelial collagen deposition present (specific measurement not provided); confirmed by trichrome staining | - Gastric mucosa: Chronic gastritis with atrophy and low-grade dysplasia; sub-epithelial collagen deposition with active chronic inflammation; increased thickness and subepithelial collagen band - No *H. pylori* infection - Hematoxylin and eosin staining performed - Specific collagen trichrome staining performed on gastric and colonic specimens - Colonic mucosa: Sub-epithelial collagen deposition with chronic inflammation - Duodenal specimens: No evidence of celiac disease | - Prior treatment: PPIs over the years without evidence of recovery - After diagnosis: Prednisolone 15 mg | - Partial response after two months of prednisolone treatment - Patient is followed up regularly - Duration of follow-up not specified | Not mentioned (no follow-up biopsy data provided) | - Long history of chronic dyspepsia resistant to PPI treatment - Diagnosed with concurrent collagenous gastritis and collagenous colitis - Partial response to prednisolone (15 mg) after two months - Ongoing regular follow-up - Chronic and resistant presentation prior to correct diagnosis - Patient had been treated for dyspepsia for years without recovery before diagnosis |
| Ref(68) | 45 | Female | Not mentioned | Nausea | No diarrhea | Not mentioned | Not mentioned | Weight loss | - Major depressive disorder (treated with venlafaxine and duloxetine - serotonin and norepinephrine reuptake inhibitors) - Celiac disease serology: Negative - No other autoimmune diseases mentioned | Not mentioned | Not mentioned | Not mentioned | Grossly normal mucosa in all segments (esophagus, stomach, duodenum); colonoscopy also showed normal mucosa upon endoscopic inspection | Not mentioned | Significant thickening of subepithelial collagen band (greater than 10 μm); demonstrated by chromotrope aniline blue stain and tenascin immunostain | Mild thickening of subepithelial collagen band (5-10 μm); positive for tenascin | - Esophagus: Lymphocytic esophagitis (greater than 40 lymphocytes per high power field); periodic acid-Schiff stain negative for fungal organisms - Gastric corpus: Mild chronic inactive inflammation with mild increase of intraepithelial lymphocytes (less than 20 per 100 epithelial cells); significant thickening of subepithelial collagen band (greater than 10 μm); chromotrope aniline blue stain and tenascin immunostain highlighted subepithelial deposits; diagnosis of collagenous gastritis - Duodenum: Celiac disease-like morphology with significant increase of intraepithelial lymphocytes (greater than 60 per 100 epithelial cells); normal villous and crypt architecture; lymphocytic duodenitis - Ileum: Normal histologically - Colon: Mild increase of cell content within lamina propria with overrepresentation of eosinophils; few intraepithelial lymphocytes; mild thickening of subepithelial collagen band (5-10 μm; positive for tenascin); diagnosis of incomplete collagenous colitis | - Combined therapy with venlafaxine and duloxetine (serotonin and norepinephrine reuptake inhibitors) for major depressive disorder - Drug-induced injury suspected (SNRI-related toxicity) | - Short-term follow-up - No detailed follow-up information available - Follow-up biopsies to document histologic resolution not yet performed | Not available (follow-up biopsies not performed) | - Developed nausea and weight loss under combined SNRI therapy (venlafaxine and duloxetine) - Endoscopic evaluation showed grossly normal mucosa in all segments - Histological examination revealed mixed lymphocytic and collagenous inflammation affecting entire gastrointestinal tract: lymphocytic esophagitis, collagenous gastritis, lymphocytic duodenitis (celiac disease-like pattern), and incomplete collagenous colitis - Drug-induced gastrointestinal injury suspected - First report of mixed lymphocytic and collagenous pattern of injury affecting esophagus, stomach, duodenum, and colon triggered by combined treatment with venlafaxine and duloxetine - Symptoms related to SNRI therapy - No information on symptom resolution after drug cessation (short-term follow-up only) |
| Ref(69) | - 16 years old at time of case report - Initially diagnosed at 11 years old - Approximately 5 years between initial diagnosis and treatment with mesalamine | Female | Diffuse, severe abdominal pain occurring 2 to 3 times per week; diffuse abdominal pain at initial presentation | Not mentioned | Episodes of diffuse watery diarrhea initially; increased non-bloody watery diarrhea at follow-up | Not mentioned in this specific patient | Not mentioned | Stooling urgency; partial relief with stooling; symptoms frequent and intrusive, disrupting regular activities | - No past medical history of autoimmune diseases - Celiac serology normal (Deaminated Gliadin Peptide IgG and IgA, Anti-Human Tissue Transglutaminase IgG and IgA, Anti-Endomysial IgA) - No immunological disorders mentioned | Not mentioned | Not mentioned | Not mentioned | Specific endoscopic findings not described; esophagogastroduodenoscopy and colonoscopy performed with biopsies | Negative (*H. pylori* immunohistochemical stain was negative) | Thick subepithelial collagen band with secondary surface epithelial stripping; confirmed by trichrome stain (specific measurement not provided) | Thick subepithelial collagen band with secondary surface epithelial stripping; diffuse evidence of collagenous colitis involving right colon, transverse colon, left colon, and rectum (specific measurement not provided) | - Gastric biopsies: Collagenous gastritis confirmed by trichrome stain; thick subepithelial collagen band with secondary surface epithelial stripping - Colonic biopsies: Diffuse evidence of collagenous colitis involving right colon, transverse colon, left colon, and rectum; thick subepithelial collagen band with secondary surface epithelial stripping - Trichrome stain: Stains collagen blue - Esophageal biopsies: No significant pathology - Duodenal biopsies: No significant pathology - Terminal ileum biopsies: No significant pathology - *H. pylori* immunohistochemical stain: Negative - Gastrointestinal involvement was severe in both stomach and all biopsied segments of colon (not patchy) | - Initially proposed: Oral budesonide (parents chose not to follow this recommendation) - Trial of dietary modification (symptoms intermittent but tolerable; no further medical intervention for approximately 5 years) - After symptom recurrence: Mesalamine 2.4 g per day; Dicyclomine 20 mg up to 3 times per day as needed for 4 weeks - Patient reported significant improvement in initial month with mesalamine - No dicyclomine taken; consistent mesalamine use | - Approximately 5 years between initial diagnosis and recurrence of symptoms requiring treatment - Follow-up at 58 days after initiation of mesalamine therapy - Family refused repeat endoscopic evaluation to determine status of pathology - Regular activities resumed; no weight fluctuation; physical examination unremarkable at follow-up | - Not available (family refused repeat endoscopic evaluation) - Calprotectin used as marker: decreased from 218 μg/g to 158 μg/g after 58 days of mesalamine therapy (normal range 0-120 μg/g) | - Initial diagnosis at age 11 with episodes of diffuse watery diarrhea, abdominal pain, and stooling urgency - Parents chose dietary modification over budesonide; symptoms intermittent but tolerable for approximately 5 years - Symptoms recurred at age 16 (increased non-bloody watery diarrhea, stooling urgency, severe abdominal pain 2-3 times per week) - Symptoms were frequent and intrusive, disrupting regular activities - Body weight fluctuating between 60th and 75th percentile; height at 70th percentile - Elevated stool calprotectin 218 μg/g; stool cultures, Giardia antigen, white cells, occult blood negative - Complete blood count, liver function panel, celiac serology all normal - Mesalamine therapy initiated with significant clinical improvement - After 58 days: no GI symptoms, consistent mesalamine use, calprotectin decreased to 158 μg/g - Excellent response to mesalamine therapy both subjectively (clinical improvement) and objectively (down-trending calprotectin) - First report describing response to mesalamine therapy in pediatric patient with both collagenous gastritis and collagenous colitis |
| Ref(70) | - Collagenous gastritis group (n=14): Mean age 13.9 years (SD 3.1) - Noncollagenous gastritis group (n=36): Mean age 16.0 years (SD 4.50) - Nongastritis group (n=25): Mean age 16.9 years (SD 3.53) - SOMAscan analysis subset: 9 collagenous gastritis patients, 12 noncollagenous gastritis patients, 8 nongastritis patients | - Collagenous gastritis: Female 5 (36%), Male 9 (64%) - Noncollagenous gastritis: Female 9 (25%), Male 27 (75%) - Nongastritis: Female 12 (48%), Male 13 (52%) - Male predominance in each group | 10 patients with collagenous gastritis | Not specifically quantified | Not specifically quantified | 10 patients with collagenous gastritis | Not mentioned | Gastrointestinal bleeding 4 patients; abdominal perforation 2 patients | - Collagenous gastritis patients did not have other autoimmune disorders - Only 1 patient had Immunoglobulin A deficiency | Corpus nodular 14 patients (all collagenous gastritis patients); antrum nodular 3 patients; 1 nongastritis patient had nodular appearance | Corpus erythema 8 noncollagenous gastritis patients, 2 nongastritis patients; antral erythema 10 noncollagenous gastritis patients, 2 nongastritis patients | Not mentioned | Diffuse nodularity in gastric corpus; large sessile polyps; 1 gastric ulcer in noncollagenous gastritis group | - All biopsies from collagenous gastritis patients negative for *H. pylori* - 1 patient in noncollagenous gastritis group had *H. pylori* | At least 10 microns in thickness (diagnostic criteria); thickened band of subepithelial collagen; all 14 collagenous gastritis patients had collagen deposition in gastric corpus, 2 patients also had collagen deposition in antrum | Not mentioned | - Corpus biopsy in collagenous gastritis: Collagen deposition 14 patients, gastritis 11 patients, eosinophils 7 patients (half of patients had increased eosinophils in gastric corpus) - Antral biopsy in collagenous gastritis: Collagen deposition 2 patients, gastritis 3 patients, eosinophils 0 patients (none had increased eosinophils in antrum) - Noncollagenous gastritis: 33 chronic inactive gastritis, 1 focal active gastritis, 1 gastric ulcer; no nodularity, no subepithelial collagen; all had antral gastritis; 7 had gastritis in corpus, 11 had normal corpus biopsies, 17 did not have corpus biopsy - Nongastritis: Normal antral biopsies, 15 had normal corpus biopsies - All collagenous gastritis biopsies reviewed by single pathologist | - No standard therapy recommended for collagenous gastritis except to correct anemia with oral or intravenous iron replacement - Specific treatments for individual patients not detailed in this proteomics study | - Time between diagnosis and blood draw ranged from 1 month to 162 months - No significant correlation between time of diagnosis and blood draw with EGF levels found | Not mentioned | - Study focused on proteomics biomarker discovery rather than clinical course - Key findings: 63 proteins significantly dysregulated in collagenous gastritis (39 increased, 24 decreased) - Epidermal growth factor (EGF) identified as novel biomarker: significantly decreased in collagenous gastritis (median approximately 2-fold decrease) compared to noncollagenous gastritis and nongastritis - FGF19 showed trend toward increase in collagenous gastritis but did not reach statistical significance - Systems biology analysis revealed: enhanced inflammatory response, immune cell migration, cell death, fibrosis; reduced vascular functions - Predicted upstream regulators: proinflammatory cytokines (TNF, IL6, IFNG), extracellular matrix and fibrosis regulators (TGFB1) - EGF potentially plays pathophysiological role in collagen deposition - First comprehensive proteome analysis in collagenous gastritis patients - Proteomics study approximately 100 cases reported worldwide; childhood-onset incidence 1/400,000 person-years |
| Ref(71) | 83 | Male | Not mentioned | Nausea, vomiting | Diarrhea | Not mentioned | Not mentioned | Weight loss over 3 months | - Celiac disease - Collagenous colitis - Collagenous sprue - Patient had history of all three conditions prior to collagenous gastritis diagnosis | Not mentioned | Not mentioned | Not mentioned | Initial forward view unremarkable; on retroflexion, diffuse oozing blood seen in fundus; repeat endoscopy 3 days later revealed serpiginous ulceration in gastric body and fundus; 3 months later (after treatment), endoscopy demonstrated healed ulcers with scarring | Negative | Thickened subepithelial collagen band (specific measurement not provided); demonstrated by trichrome stain | Not mentioned in this report (patient had known collagenous colitis from history) | - Thickened subepithelial collagen band - Stripping of luminal epithelium - Increased intraepithelial lymphocytes - Expansion of lamina propria by plasma cells and lymphocytes with scattered eosinophils - Loss of fundic/oxyntic glands - Atrophic collagenous gastritis pattern - Trichrome stain performed - Negative for amyloid deposition - Negative for *H. pylori* - Distinct histologic form: atrophic pattern of collagenous gastritis | - Hemostatic powder spray for acute bleeding - Viscous budesonide (resulted in resolution of symptoms) | - Repeat endoscopy 3 days after initial procedure - Follow-up endoscopy 3 months later | Not mentioned (follow-up endoscopy at 3 months showed healed ulcers with scarring, but no histological data provided) | - Presented with 3-month history of nausea, vomiting, diarrhea, and weight loss - Initial esophagogastroduodenoscopy: forward view unremarkable, but retroflexion revealed diffuse oozing blood in fundus - Gastric barotrauma suspected despite usual insufflation - Acute bleeding treated with hemostatic powder spray - Repeat endoscopy 3 days later showed serpiginous ulceration in gastric body and fundus - Diagnosis of atrophic collagenous gastritis made on histopathology - Treatment with viscous budesonide resulted in symptom resolution - Follow-up endoscopy at 3 months demonstrated healed ulcers with scarring - Underlying inflammation and atrophy likely contributed to development of barotrauma |
| Ref(72) | 39 | Male | Not specifically mentioned | Not specifically mentioned | Yes (nocturnal diarrhea) | Not mentioned | Not mentioned | Changes in bowel habit; dyspeptic symptoms | - IgG4-related disease - Serum IgG4 level: 1.269 g/L (reference range 0.039-0.864 g/dL) | Not mentioned | Yes (hyperemic antral mucosa) | Not mentioned | Loss of gastric folds; irregular antral mucosa; polypoid mucosal pattern in terminal ileum | Not mentioned | Not specified | Present (collagenous colitis diagnosed, but thickness not specified) | - Collagenous gastritis - IgG4-positive plasma cell infiltration (64 IgG4-positive plasma cells per HPF) - Gastritis cystica profunda - Intraepithelial lymphocytosis in duodenum - Collagenous colitis - IgG4-positive plasma cells in colonic mucosa and around mucosal lymphoid follicles in terminal ileum - EUS: thickening of mucosa and submucosa layers of fundus and corpus with numerous hypoechoic cystic lesions | Immunosuppressive therapy | Follow-up performed (duration not specified) | Improvement in histopathologic IgG4-positive plasma cell infiltration | - Improvement in clinical symptoms after immunosuppressive therapy - This is noted as the first case report to speculate on the relationship between IgG4-related GI disease and collagenous gastritis with submucosal hypoechoic-cystic lesions |
| Ref(73) | 24 | Female | Not mentioned as presenting symptom | Not mentioned | Not mentioned | Not mentioned | Not mentioned | Recurrent abdominal distension and discomfort for 3 months; epigastric distension after eating lasting 30 minutes to 12 hours; intermittent abdominal distension continued for >6 months | - No autoimmune diseases mentioned - History of allergies, asthma denied - Extractable nuclear antigen antibodies negative | Yes (diffuse nodular elevation-depression changes in the mucosa of the entire gastric corpus) | Yes (nodular reddening-like changes in the anterior wall of the gastric antrum) | Yes (mentioned in pathology reports) | Mucosa of angulus was smooth; mucosa of gastric corpus and fundus appeared uneven with elevation-depression changes; ME-BLI showed clear borders of elevation, regular arrangement of MCE, widening of microvascular vessels in elevated areas; irregular microvascular pattern with dendritic changes in depressed areas | Negative (13C urea breath test negative) | Approximately 40 μm (in gastric fundus, body, and antrum) | Positive Masson's trichrome staining in terminal ileum, ascending colon, and rectum (thickness not specified) | - Moderate chronic inflammation with erosion and mild activity - Higher number of lymphocytes infiltrating the interstitium - Few neutrophils and eosinophils (20-30 per high-powered field) infiltrating the interstitium - Multifocal lymphoid tissue hyperplasia - Collagenous bands deposited under the mucosal epithelial (about 40 micrometers) - Positive Masson's trichrome staining - Negative Congo red staining - Eosinophilic type collagenous gastritis - Endoscopic ultrasound: thickened mucosal layer of gastric corpus appearing slightly hypoechoic with wavy changes; normal structures of mucosa, muscularis mucosa, and submucosa at gastric antrum elevation replaced by inhomogeneous hypoechoic changes - Computed tomography: relative thickening of gastric wall in gastric corpus and antrum with mild enhancement in arterial phase - Colonoscopy and biopsy showed collagenous colitis (moderate mucosal chronic inflammation with erosion, mild activity, focal lymphocytosis, positive Masson's trichrome staining) | - Esomeprazole 20 milligrams once daily (30 minutes before breakfast) for 2 weeks - Mosapride 5 milligrams three times daily (before each meal) for 2 weeks, then irregularly - Prednisone acetate 30 milligrams daily for 2 weeks, then tapered by 5 milligrams every 2 weeks until discontinued (total 8 weeks) - Mosapride 5 milligrams three times daily continued during glucocorticoid treatment | - July 2020: abdominal distension relieved after initial treatment - Late 2020 to first half of 2021: continued intermittent abdominal distension for more than 6 months - June 2021: Pepsinogen I was 105.21 micromoles per liter, Pepsinogen II was 24.3 micromoles per liter (elevated), Pepsinogen I/II ratio was 4.33 (decreased), gastrin-17 was 46.99 picomoles per liter - October 2021: Pepsinogen I was 118 micromoles per liter, Pepsinogen II was 33.74 micromoles per liter (elevated), Pepsinogen I/II ratio was 3.5 (decreased), gastrin-17 was 39.69 picomoles per liter (elevated) - As of November 2021: still undergoing follow-up | - No follow-up histological examination reported - White-light endoscopic findings in 2020 and 2021 were similar - Endoscopic ultrasound findings showed progression: only mucosa involved in gastric corpus, while mucosa, muscularis mucosa, and submucosa all involved in gastric antrum in 2021 | - Initial treatment with esomeprazole and mosapride provided temporary relief for 2-3 days per week - Symptoms recurred intermittently for more than 6 months despite irregular medication use - After 8 weeks of glucocorticoid treatment, patient continued to have intermittent abdominal distension lasting 12 hours in severe episodes - After treatment, abdominal distension was relieved for 2-3 days every week - Classified as child-adolescent type of collagenous gastritis (inflammation limited to stomach, no intestinal involvement on colonoscopy) - Eosinophilic type based on pathological findings - Serum Pepsinogen I, Pepsinogen II, Pepsinogen I/II ratio, and gastrin-17 suggested as potential non-invasive monitoring markers - Patient continued follow-up as of November 2021 |
| Ref(74) | - Median age at diagnosis: 12.5 years (range: 7-15.2 years) - Individual ages: Case 1: 13.2 years, Case 2: 11.7 years, Case 3: 11.0 years, Case 4: 7.0 years, Case 5: 13.6 years, Case 6: 10.5 years, Case 7: 14.1 years, Case 8: 8.8 years, Case 9: 13.5 years, Case 10: 13.2 years, Case 11: 15.2 years, Case 12: 11.4 years | - Female: 8 (67%) - Male: 4 (33%) - Male/female ratio: 1/2 - Mild female predominance | 6/11 (55%); types included umbilical abdominal pain (3 cases), epigastric pain (1 case), hypogastric pain (1 case) | Not mentioned | No diarrhea observed in any patient | Present in all children 11/11 (100%); median lowest hemoglobin level 5.1 g/dL (range: 2.8-9.1 g/dL) | Not mentioned | Nonspecific symptomatology attributed to anemia 8/10 (80%); pallor and asthenia (6 cases); heart palpitations exercise-induced (2 cases); hematemesis and melena (1 case from gastric ulcer); asymptomatic iron deficiency anemia discovered incidentally (4 cases); four patients required transfusions before first endoscopic evaluation | - Only one patient had family history of autoimmune disorders (mother had hypothyroidism, paternal grandparents had diabetes and thyroid disorders) - Anti-transglutaminase antibodies negative (celiac disease excluded) in 9 patients - Complete autoimmune panel (anti-smooth muscle, anti-TPO, anti-gastric parietal cells, anti-mitochondria, anti-nuclear, ANCA, ASCA, anti-thyroglobulin, anti-tyrosine peroxidase antibodies) negative in Case 7 - Vitamin B12 levels normal in 2 patients - Ferritin level less than 10 mg/L in 8 patients with data | Present in 10/12 patients (83%); antrum only (2), fundus only (4), antrum and fundus (4); micronodules observed in follow-up (Case 1) | Not mentioned | Diffuse superficial erosions (Case 8); ulcerations in corpus (Case 6) | Normal macroscopic evaluation (Case 1); slight gastric bleeding (Cases 11 and 12); petechial lesion in rectum and sigmoid colon (Case 7); video capsule endoscopy showed active gastric bleeding (Case 3 of 4 who had procedure); all duodenal macroscopic evaluations standard; no macroscopic lesions in colon except Case 7 | - Positive: 1/12 (Case 8 - active pangastritis positive for *H. pylori*) - Negative: 11/12 (92%) | Range from 19 to 100 μm; individual measurements: Case 1: 35 μm, Case 2: 44 μm, Case 4: 25 μm, Case 5: 20 μm, Case 7: 20 μm, Case 8: 100 μm, Case 9: 35 μm, Case 10: 30 μm, Case 11: 41 μm, Case 12: 19 μm; data not provided for Cases 3 and 6 | No associated collagenous colitis observed in any patient | - All cases: Collagen deposits greater than 10 μm under epithelial mucosa; thickness range 19-100 μm - Visible on standard stains (hematoxylin and eosin) or special staining (Masson trichrome or Sirius red) - All cases: Inflammatory infiltrate of lamina propria of variable intensity, consisting mainly of lymphocytes and plasma cells - Specific findings by case: lymphocyte infiltration (Case 1), polynuclear and lymphoplasmacytic infiltration (Cases 2, 3, 6), lymphoplasmacytic infiltration (Cases 4, 5, 7, 9, 10, 11, 12), neutrophils and lymphoplasmacytic infiltration with *H. pylori* infection (Case 8) - Duodenal mucosa: Normal or sometimes with discrete nonspecific chronic inflammatory infiltrates without villous atrophy - Ileal biopsies: Normal in all cases - Colonic biopsies: All normal or sometimes with discrete nonspecific chronic inflammatory infiltrate - None had eosinophilic chorionic infiltrate or biopsies consistent with lymphocytic gastritis - No dysplasia or metaplasia reported | - PPIs: 11/12 patients (all except Case 6); decrease in abdominal pain reported in 3 patients - Iron supplementation (oral or intravenous): All 12 patients; noticeable improvement in hemoglobin levels in all patients; none required transfusion after diagnosis with prescribed iron supplementation - Viscous budesonide: 1 patient (Case 1, started 2 years after diagnosis) - Oral prednisone: 1 patient (Case 8, interrupted after 1 month due to recurrent anemia) - No diet modifications attempted in any patient | - Estimated median time between onset of symptoms and first endoscopic examination: 15 months (range: 2-30 months) - Follow-up endoscopies: 8/12 patients - Median endoscopic follow-up: 2.9 years (range: 0.5-6.5 years) - Years of diagnosis ranged from 1995 to 2022 | - Case 1: Biopsies remained stable after 2 years (micronodules with mild hemorrhage in antrum observed) - Case 5: No improvement in biopsies despite macroscopic improvement - Case 8: Reduction in collagenous thickening on gastric biopsy after 7 years of follow-up; macroscopic improvement also observed - Other patients with follow-up: No specific histological improvement data provided | - Recurrence of iron deficiency when supplementation interrupted: 9/10 patients (90%) - Case 1: After 1 year of viscous budesonide treatment (started 2 years post-diagnosis), no longer manifested anemia or abdominal pain; steroids and iron supplementation could be interrupted - Case 8: Treated with oral prednisone but interrupted after 1 month due to recurrent anemia; showed macroscopic and histological improvement after 7 years associated with PPIs and oral iron supplements - Case 6 (patient with collagenous colitis): Normal stool after budesonide treatment, but diarrhea reappeared after treatment withdrawal - Biological response to oral iron supplementation satisfactory despite association with antisecretory treatment, rejecting malabsorption - Digestive hemorrhage demonstrated in 5 patients - Continuous gastric bleeding and ongoing inflammation possibly responsible for profound anemia and recurrence when martial supplementation stopped - Four patients required blood transfusions before diagnosis - Abdominal echography normal in 6 patients; magnetic resonance imagery normal in 1 patient - Fecal calprotectin elevated in 2 patients (Cases 11 and 12: 151 mg/g and 479 mg/g respectively) - C-reactive protein and sedimentation rate normal in all patients tested - Childhood-onset collagenous gastritis with lesions mainly restricted to stomach - No overlap with collagenous colitis in any patient - Isolated forms of collagenous gastritis do not correlate with collagenous colitis in this cohort |
| Ref(75) | - Case 1: 13 years old (female) - Case 2: 13 years old (male) - Case 3: 11 years old (female) - Mean age: 12.3 years | - Female: 2 (67%) - Male: 1 (33%) | All 3 patients (100%); epigastric pain (Cases 1, 3), asthenia (Case 2) | Not mentioned | Not mentioned | All 3 patients (100%); 2 with moderate anemia, 1 with severe anemia | Not mentioned | Asthenia and headache (2 patients), constipation (Case 3), abdominal distention (Case 3) | - Case 1: Family history - mother with hypothyroidism, grandmother with diabetes mellitus type 1; positive ANA test - Case 2: Family history - grandmother with rheumatoid arthritis and systemic lupus erythematosus; positive serum Waaler Rose test (10 UI/mL) - Case 3: No autoimmune diseases mentioned - Celiac serology negative in all patients - Auto-antibodies negative except as noted above | - Case 1: Slightly hyperemic and edematous gastric body mucosa with diffuse micronodularity - Case 2: Diffuse hyperemia with macroscopic micronodular pattern - Case 3: Slight hyperemia and edema with some erosions and widespread sessile polyps at fundus and gastric body - Nodularity: Not mentioned - Erythema: Present in all 3 cases - Erosion: Case 3 | | | | - Case 1: Positive (*H. pylori* infection detected); eradicated with triple therapy - Case 2: Not mentioned - Case 3: Not mentioned | - All cases: Greater than 10 μm (diagnostic criterion) - Specific measurements not provided - Stomach: Thickened sub-epithelial collagen band greater than 10 μm in all cases - Colon: None (no collagenous colitis in any patient) | | - Case 1: Diffuse sloughing of surface epithelium with thickened sub-epithelial collagen band greater than 10 μm; infiltration of lymphomononuclear and eosinophils in lamina propria - Case 2: Prominent sub-epithelial collagen band greater than 10 μm; moderate superficial chronic inflammatory infiltrate, especially in lamina propria, affecting mainly gastric body - Case 3: Widespread flaking of surface epithelium; irregular, marked thickened sub-epithelial collagen bands greater than 10 μm containing dilated capillaries and some eosinophils; mild-moderate increase in lymphomononuclear and eosinophilic infiltrate in lamina propria; slight atrophy of some glands in pseudopiloric metaplasia - Ileocolonoscopy normal in all cases except lymphoid hyperplasia (Case 1: ileum and sigmoid colon; Case 2: ileum and colon; Case 3: sigmoid colon and ileum) | - Case 1: Oral iron supplements (poor response); ferric carboxymaltose (FCM) infusion 15 mg/kg (2 infusions, 7 days apart); folic acid; *H. pylori* eradication triple therapy for 14 days; later maintained with oral iron during menstruation only - Case 2: Oral iron supplements (poor response, worsening epigastric pain); FCM infusion 15 mg/kg (2 infusions over follow-up period); PPIs (pantoprazole 1 mg/kg/day) - escalated; poor compliance to oral iron maintenance - Case 3: PPIs (omeprazole 40 mg/day) - gradually escalated; oral iron supplements plus folic acid; FCM infusion 15 mg/kg (1 infusion); sucralfate; macrogol for constipation; maintained with oral iron during menstrual periods - All patients: Mean of 2 FCM infusions/year | - Case 1: 37 months (3 years) - Case 2: 15 months - Case 3: 27 months - Mean follow-up: 26.3 months | - Case 1: Upper GI endoscopy repeated 6 months after diagnosis - previous findings confirmed despite *H. pylori* eradication; repeat at 3 years showed slight reduction of inflammatory infiltrate with persistence of thickened sub-epithelial collagen band - Case 2: Upper GI endoscopy 3 months after diagnosis showed comparable macroscopic findings with partial improvement of inflammatory infiltrate - Case 3: Upper GI endoscopy repeated at 3 months, 6 months, and 27 months - all unchanged | - All patients: Initially treated with oral iron supplements with no benefit, principally due to poor compliance secondary to worsening of epigastric pain and PPI resistance - FCM infusion: Good clinical and laboratory response in all cases; median rise in hemoglobin after single infusion 2.9 g/dL (range 1.6-3.3 g/dL) - Stable hemoglobin levels with FCM infusions; no adverse outcomes - No blood transfusions required after diagnosis - Case 1: Mean hemoglobin 12.2 g/dL at last follow-up; no GI symptoms, only sporadic headache; 13CU-breath test confirmed *H. pylori* eradication; ferritin 9 mcg/L, iron 8 mmol/L - Case 2: Hemoglobin 14 g/dL at 15-month follow-up; stable hematologic indices; complete remission of GI symptoms; ferritin 36 mcg/L, iron 17.2 mcg/dL - Case 3: Asymptomatic at 27-month follow-up except copious menstrual cycle; hemoglobin 11.7 g/dL, ferritin 58 mcg/dL, iron 6.9 mmol/L; poor compliance to oral iron due to recurrent abdominal pain |
| Ref(76) | 38 | Female | Intermittent mild periumbilical cramping; occasional sporadic abdominal cramping at 2-month follow-up | Not mentioned | Denied diarrhea | One-year history of microcytic anemia; hemoglobin 9.9 g/dL (baseline 13-14 g/dL) | Not mentioned | Pagophagia (ice eating); six-year history of iron deficiency; hypothalamic amenorrhea; denied melena, hematochezia, fevers, chills, weight loss, dysphagia | - Hypothalamic amenorrhea - Celiac disease panel negative - Anti-parietal cell antibodies negative - Intrinsic factor antibodies negative - No other autoimmune diseases mentioned | Diffusely nodular gastric mucosa in antrum and pylorus; pre-pyloric region | Diffusely erythematous gastric mucosa in antrum and pylorus | Not mentioned | Marked friability of gastric mucosa with subsequent bleeding during random gastric biopsies; famotidine 40 mg intravenously given intra-procedurally | Negative (*H. pylori*  testing negative both serologically and on biopsy) | Greater than or equal to 10 μm (diagnostic criterion); described as "thick band of collagen" | Not mentioned (colonoscopy with intubation of terminal ileum was unremarkable) | - Gastric body biopsy: Deposition of thick band of collagen under surface epithelium infiltrated by few eosinophils - Complete loss of oxyntic glands - Absence of parietal cells - Collagenous gastritis with severe atrophy - Eosinophil-rich pattern - *H. pylori* testing negative on pathology - Hematoxylin and eosin staining performed | - Omeprazole 40 mg by mouth once daily for 6 weeks (acid suppression therapy) - Vitamin B12 supplementation - Ferrous sulfate 325 mg by mouth once daily (started at 2-month follow-up) - Famotidine 40 mg intravenously given intra-procedurally during endoscopy for bleeding | 2-month follow-up | Not mentioned (no repeat endoscopy or biopsy data provided) | - Presented with 1-year history of anemia and 6-year history of iron deficiency - Laboratory findings: Hemoglobin 9.9 g/dL (reference range 11.1-15.9), mean corpuscular volume 75 fL (reference range 79-97), iron saturation 7% (reference range 15-55), iron 33 mg/dL (reference range 27-159), ferritin 4 ng/mL (reference range 15-150), vitamin B12 144 pg/mL - Concerns for malabsorption as cause of iron and vitamin B12 deficiency - Esophagogastroduodenoscopy revealed diffusely nodular and erythematous gastric mucosa - Random gastric biopsies demonstrated marked friability with subsequent bleeding - Diagnosis of collagenous gastritis with severe atrophy (eosinophil-rich pattern, atrophic gastritis pattern) - Colonoscopy with intubation of terminal ileum unremarkable (no collagenous colitis) - Complete metabolic panel, fat-soluble vitamins, and immunoglobulin levels normal - At 2-month follow-up: occasional sporadic abdominal cramping but otherwise well; hemoglobin 10.1 g/dL, ferritin 5 ng/mL - Atypical presentation: adult patient with symptoms and endoscopic findings consistent with previously described pediatric (not adult) cases - Patient had iron deficiency anemia and abdominal pain (typical pediatric presentation) rather than diarrhea (typical adult presentation) - No coexisting collagenous colitis or collagenous sprue (common in adult cases) |
| Ref(77) | - 11 years old at time of presentation - 14 years old at time of esophagogastroduodenoscopy and diagnosis | Female | Not mentioned as presenting symptom | Not mentioned | Not mentioned | Yes (severe symptomatic iron deficiency anemia; long history of iron deficiency anemia) | Not mentioned | Six-week history of worsening nonexertional shortness of breath; palpitations (intermittent, self-resolving, not associated with exercise); chest pain (described as sharp, burning, or heavy; self-resolving, not associated with palpitations or exercise); reduced exercise tolerance; profound lethargy after just 2 exercise sessions (usually exercised 7 times per week); dyspnea (initially believed to be contributed to episodes of hyperventilation with anxiety, not associated with exercise) | - Strong family history of autoimmune conditions: father had alopecia; maternal grandmother had Hashimoto thyroiditis - Previously, adult phenotype of collagenous gastritis predominantly associated with autoimmune disorders - Increasing evidence suggests link also exists with pediatric phenotype - Celiac serology normal - Thyroid function tests normal - Total Immunoglobulin A, Immunoglobulin M, and Immunoglobulin G all normal | Not mentioned | Not mentioned | Not mentioned | Four regions of superficial gastritis measuring from 5 to 7 millimeters; endoscopic appearance can include nodularity, mucosal erythema, hemorrhage, or ulceration (general description, not specific to this case) | Negative (no *H. pylori* infection confirmed using Loeffler methylene blue stain) | At least 10 micrometers (characteristic histological finding; specific thickness not reported for this case) | Not mentioned; no collagenous colitis reported | - Esophageal biopsies: squamous mucosa with no evidence of inflammation, hyperplasia, or fungal colonization (using periodic acid-Schiff stain) - Gastric biopsies: no active gastritis; patchy but focally increased hyaline collagen deposition (confirmed with Masson trichrome stain) within superficial lamina propria, extending to just beneath superficial epithelium; associated with trapped lymphocytes - Duodenal biopsies: no evidence of duodenitis; villous surface epithelium indicated reactive changes with patchy, overall-moderate intraepithelial lymphocytosis extending to tips of villi with no identified organism - Subepithelial thickening of collagen band at least 10 micrometers in thickness - Five random esophageal, gastric, and duodenal biopsies obtained | - Transfusion of packed red blood cells (improved dyspnea, lethargy, and palpitations) - Oral iron supplementation - Vitamin C supplementation - Focus on monitoring and maintaining appropriate iron stores to address severe symptomatic iron deficiency anemia - Regular long-term follow-up because of high suspicion for additional autoimmune diseases - Current key focus: identification and treatment of symptoms to improve patient's overall quality of life | - Outpatient follow-up with general pediatrician arranged - Recurrent persistent symptoms led to further workup - Referred to tertiary center at 14 years for further evaluation by gastroenterologist - Regular long-term follow-up and monitoring of disease recommended | No follow-up histological examination reported in this case report | - Long history of iron deficiency anemia - Six-week history of worsening symptoms before admission - Received transfusion of packed red blood cells which improved dyspnea, lethargy, and palpitations - Commenced on oral iron and vitamin C supplementation - Recurrent persistent symptoms led to further workup - Premenarchal at time of presentation - Eating varied, nonrestrictive diet including red meat 5 times per week - No suggestive cause of anemia from patient's and parents' collateral history - Diagnosed with collagenous gastritis based on histopathological findings in association with clinical history - Natural history and long-term progression of collagenous gastritis is variable; majority seems to have chronic persistent disease pattern |
| Ref(78) | 71 | Female | Denied | Yes (nausea and vomiting for approximately 5 years; experienced several weeks of nausea and vomiting after motor vehicle accident) | Yes (alternating episodes of diarrhea and constipation) | Yes (anemia with blood hemoglobin 9.5 grams per deciliter; improved to 13.2 grams per deciliter after treatment) | Yes (chronic early satiety) | Progressive weight loss (initial 20-pound weight loss, then additional 80 pounds over several years for total of 100 pounds); denied hematemesis, melena, or rectal bleeding; symptoms first noticed after hospitalization for injuries sustained during motor vehicle accident | - Incidentally discovered hypogammaglobulinemia and Immunoglobulin A deficiency - Low levels of serum Immunoglobulin G and Immunoglobulin A (616 and 27 milligrams per deciliter, respectively) - Workup for celiac sprue and autoimmune gastritis unrevealing - Normal testing for iron, anti-tissue transglutaminase/gliadin antibodies, and gastrin - However, in setting of hypogammaglobulinemia, results could not definitively rule out celiac sprue - Human leukocyte antigen-D2 and Human leukocyte antigen-DQ8 testing negative - Celiac sprue could not be ruled out | Yes (hypertrophic, nodular-appearing mucosa in stomach antrum and body) | Not specifically mentioned | Not mentioned | Duodenum featured no endosonographic abnormalities and was not sampled; endoscopically mucosa appears nodular | Not mentioned | Patchy thickening of collagen bands up to 12 micrometers within lamina propria in body biopsies | Not mentioned; no colonoscopy performed | - Atrophic gastritis with moderate activity and diffuse intestinal metaplasia - Body biopsies showed patchy thickening of collagen bands up to 12 micrometers within lamina propria - Entrapped capillaries and surface epithelial disruption - Overall findings consistent with collagenous gastritis - Clinical and serologic correlation required to exclude celiac sprue and autoimmune gastritis - Duodenum was not sampled as it featured no endosonographic abnormalities - Gastric emptying study unremarkable | Advised to try gluten-free diet | - Early satiety resolved - Gained weight - Hemoglobin improved up to 13.2 grams per deciliter - Patient recommended to repeat esophagogastroduodenoscopy/colonoscopy but did not pursue additional care within first year of follow-up | No follow-up histological examination reported | - Past medical history notable for hyperlipidemia, osteoarthritis, and longstanding thrombocytosis treated with hydroxyurea - Experienced nausea, vomiting, progressive weight loss, and early satiety for approximately 5 years - Symptoms first noticed after hospitalization for motor vehicle accident injuries - Gastrointestinal upset initially subsided but chronic early satiety and progressive weight loss continued over following several years - After gluten-free diet, early satiety resolved, gained weight, and hemoglobin improved - Atypical presentation of collagenous gastritis |
| Ref(78) | 43 | Female | Yes (abdominal pain following ingestion of spicy foods; abdominal discomfort resolved with rabeprazole) | Yes (nausea and vomiting associated with excessive postprandial pharyngeal secretions for six months) | Not mentioned | Not mentioned | Not mentioned | Excessive postprandial pharyngeal secretions for six months associated with cough; denied hematemesis, melena, weight changes, or rectal bleeding | - Developed diffuse myalgias one and one-half years after initial presentation - Extensive rheumatologic laboratory testing provided no evidence for connective tissue disease or inflammatory myopathy - Symptoms most consistent with myofascial pain syndrome/fibromyalgia - Fibromyalgia successfully managed by acupuncture and physical therapy - Allergy testing and workup for celiac sprue and autoimmune gastritis unremarkable (antigliadin antibody, anti-transglutaminase antibody, antiparietal cell antibody, and intrinsic factor analysis) - Human leukocyte antigen testing not performed - Duodenal biopsy showed no disruption of normal villous architecture - Vague history of irritable bowel syndrome | Yes (nodular gastric mucosa) | Yes (erythematous gastric mucosa) | Not mentioned | Mild reflux esophagitis; sessile serrated adenoma of cecum; tubular adenoma of sigmoid colon; congested gastric mucosa; endoscopically mucosa appears nodular and erythematous with depressed areas surrounding nodules corresponding to subepithelial collagen deposition seen histologically | Negative (*H. pylori* breath test negative) | Focal thickening of subepithelial collagen bands up to 10 micrometers in random biopsies of stomach antrum and body | Not mentioned; colonoscopy performed but no collagenous colitis reported | - Moderate chronic active gastritis - Increased intraepithelial lymphocytes - Focal thickening of subepithelial collagen bands up to 10 micrometers - Findings most consistent with collagenous gastritis - Duodenal biopsy showed no disruption of normal villous architecture - Random biopsies of stomach antrum and body obtained - Endoscopy did not demonstrate visual colonic abnormalities - Extragastric collagen deposition not observed within duodenal biopsy | - Empirically treated for gastroesophageal reflux disease with rabeprazole (resulted in resolution of only abdominal discomfort) - Recommended to avoid nonsteroidal anti-inflammatory drugs - Low-gluten diet | - Complete symptom resolution after low-gluten diet - One and one-half years later developed diffuse myalgias diagnosed as myofascial pain syndrome/fibromyalgia - At four years of follow-up, patient continued to deny gastrointestinal complaints - Esophagogastroduodenoscopy/colonoscopy at four years showed unchanged gastric mucosal findings | Unchanged gastric mucosal findings observed by esophagogastroduodenoscopy/colonoscopy at four years of follow-up | - Past medical history: migraines, anxiety, vague history of irritable bowel syndrome - Complained of excessive postprandial pharyngeal secretions for six months with associated cough, nausea, and vomiting - Abdominal pain following ingestion of spicy foods - Empirically treated with rabeprazole for gastroesophageal reflux disease with resolution of abdominal discomfort only - After avoiding nonsteroidal anti-inflammatory drugs and beginning low-gluten diet, complete symptom resolution occurred - One and one-half years later developed fibromyalgia - At four years of follow-up, continued to deny gastrointestinal complaints despite unchanged gastric mucosal findings - Did not require medications to manage gastrointestinal complaints; completely responded to restricting gluten intake - Atypical presentation of collagenous gastritis - Chief complaints likely attributed to isolated collagenous gastritis and not concurrent collagenous colitis |
| Ref(79) | - Median age: 32.5 years (range: 17-80 years) - Individual ages: Case 1: 67 years, Case 2: 47 years, Case 3: 19 years, Case 4: 45 years, Case 5: 20 years, Case 6: 80 years, Case 7: 47 years, Case 8: 17 years, Case 9: 18 years, Case 10: 18 years | - Female: 6 (60%) - Male: 4 (40%) | 7/10 patients (70%) | Not mentioned | Not mentioned | 10/10 patients (100%); iron deficiency anemia | 2/10 patients (20%); Case 1 and Case 2 | Gastroesophageal reflux disease (3 patients, 30%); weight loss (1 patient, Case 1); overt bleeding from upper gastrointestinal tract (1 patient, Case 7); Type 1 diabetes mellitus (1 patient, Case 4); asthma (1 patient, Case 4); pagophagia (ice eating) mentioned in discussion section | - Type 1 diabetes mellitus: 1 patient (Case 4) - Pernicious anemia (vague history): 1 patient (Case 7), but negative antibodies detected - Asthma: 1 patient (Case 4) - No other autoimmune diseases mentioned | Gastric mucosal nodularity 6/8 patients with available endoscopic reports (75%); diffuse nodular gastric mucosa (Case 2, Case 3), patchy nodular mucosa (Case 9), gastric mucosa nodularity (Case 7, Case 8, Case 10) | 6/8 patients with available endoscopic reports (75%); diffuse nodular gastric mucosa with erythema (Case 2, Case 3), gastric mucosa nodularity with erythema (Case 7, Case 8, Case 10), mild erythema (Case 9) | 1/8 patients (Case 5); erosive gastric mucosa | Normal mucosa (1 patient, Case 6); endoscopic reports not available for 2 patients | Not mentioned for individual patients in this study | - Greater than or equal to 10 micrometers (diagnostic criterion); thickened subepithelial collagen band with "basketweave" pattern typically greater than or equal to 10 micrometers in thickness - Specific measurements not provided for individual patients - Collagen deposition locations: Gastric antrum and body (4 cases, 40%), body only (5 cases, 50%), antrum only (1 case, 10%) | None (patients with synchronous or metachronous collagenous colitis excluded from study) | - All cases: Subepithelial collagen deposition with "basketweave" pattern (typically greater than or equal to 10 micrometers thick); chronic inflammation of lamina propria; intraepithelial lymphocytosis - Eosinophils: Moderate number present in affected gastric mucosa; more than 30 eosinophils per high power field (6 patients, 60%); intraepithelial eosinophilia (4 patients, 40%) - Individual patient eosinophil counts per high power field and intraepithelial migration: Case 1: 87 eosinophils with yes intraepithelial migration, Case 2: 52 eosinophils with no intraepithelial migration, Case 3: 57 eosinophils with yes intraepithelial migration, Case 4: 12 eosinophils with no intraepithelial migration, Case 5: 27 eosinophils with no intraepithelial migration, Case 6: 5 eosinophils with no intraepithelial migration, Case 7: 35 eosinophils with no intraepithelial migration, Case 8: 53 eosinophils with yes intraepithelial migration, Case 9: 29 eosinophils with no intraepithelial migration, Case 10: 71 eosinophils with yes intraepithelial migration | - PPIs: 8/9 patients with documented treatment (89%) - H2-receptor antagonists: Mentioned as part of treatment - Steroids: 3/9 patients (33%); prednisone and/or budesonide - Immunomodulators: Azathioprine (1 patient, Case 2), vedolizumab (1 patient, Case 2) - Iron supplement: 1 patient (Case 3) - Aspirin use: 2 patients combined with antisecretory antacids (20%) | - Follow-up documentation available for 8 patients - Follow-up duration: Up to 5 years | - Persistent disease (symptoms and histology): 1 patient (Case 2) - Persistent symptoms but no follow-up endoscopy or biopsies: 3 patients - Asymptomatic but patchy residual collagen deposition in biopsies: 4 patients (Case 3, Case 8, Case 9, Case 10) | - Persistent symptoms and biopsy features: 1 patient (Case 1) - persistent anemia, no endoscopy follow-up - Disease persistent (symptoms and histology) with persistent anemia: 1 patient (Case 2) - Follow-up biopsy showed persistent residual collagen band, negative symptoms: 1 patient (Case 3) - Patient lost to follow-up: 2 patients (Case 4, Case 6) - Intermittent symptoms including abdominal pain, no follow-up biopsy: 1 patient (Case 5) - Intermittent symptoms including anemia: 1 patient (Case 7) - Persistent residual collagen band, negative symptoms: 3 patients (Case 8, Case 9, Case 10) |
| Ref(80) | 20 | Female | Chronic and diffuse abdominal pain; diffuse abdominal pain to deep palpation on physical examination | Nausea, regurgitation; improvement in nausea and vomiting after treatment | Not mentioned | Not mentioned | Early satiety | Gastroesophageal reflux disease; migraines; allergic rhinitis | - No autoimmune diseases mentioned - Allergic rhinitis (part of medical history) - No history of chronic pediatric disorders - Family history: Grandmother had similar gastrointestinal symptoms but no confirmed diagnosis - Workup for celiac disease negative (tissue transglutaminase and deaminated gliadin antibodies negative) | Significant gastric nodularity; nodularity in gastric body; characteristic nodular appearance of gastric antrum caused by intervening areas of depressed mucosa with glandular atrophy and collagen deposition; persistent gastric nodularity after treatment despite histopathologic improvement | Gastric erythema; erythema and nodularity | Focal erosion on biopsy | Atrophic gastric body; esophagus showed normal-appearing mucosa; narrow-band imaging highlighted contrast between nodular areas representing normal tissue architecture and depressive mucosal lesions seen between nodular lesions; mucosal surface of nodular lesions did not show marked changes or abnormal capillary vessels on narrow-band imaging | Not mentioned | - Greater than 10 micrometers in thickness; subepithelial collagen deposition greater than 10 micrometers thick - Histologically characterized by heterogeneous subepithelial surface collagen deposition generally between 10 and 100 micrometers in thickness - Thickness of collagen deposition directly related to chronicity of disease - Collagenous deposition remained largely unchanged after treatment despite significant histopathologic improvement | Not mentioned | - Initial histopathology of gastric body: Subepithelial collagen deposition greater than 10 micrometers in thickness; sloughing of overlying surface epithelium; loss of specialized gastric glands; inflammatory infiltrate consisting of eosinophils, plasma cells, and lymphocytes - Trichrome stain highlighted characteristic collagen deposition; increased subepithelial collagen with some loss of specialized gastric glands - Type III collagen deposition from subepithelial fibroblasts as reparative response to inflammatory environment - Inflammatory infiltrate consists of plasma cells, eosinophils, and lymphocytes (suggests possible autoimmune pathophysiology) - Collagen deposition in colon seen with collagenous colitis appears more uniform compared to heterogeneous deposition in collagenous gastritis - After treatment (7 months after initiation of intravenous vedolizumab): Significant improvement in histopathology with healthy surface foveolar cells and return of specialized gastric glands with few plasma cells in lamina propria; collagenous deposition remained largely unchanged | - Initial medications: Esomeprazole (PPI), hyoscyamine, rizatriptan, gabapentin, acetaminophen, amitriptyline - Topical budesonide 9 milligrams daily: Stopped after 2 weeks because of no significant improvement in symptoms; initial therapy did not result in clinical or endoscopic improvement - Repeat esophagogastroduodenoscopy: No improvement in gastric body nodularity or subepithelial collagen deposition on biopsy - Intravenous vedolizumab infusions: Loading dose of 300 milligrams at 0, 2, and 6 weeks, followed by maintenance dosing at 300 milligrams every 8 weeks; continued as maintenance therapy every 8 weeks thereafter - No other medications including budesonide or systemic oral steroids given while on intravenous vedolizumab - Vedolizumab is monoclonal antibody against alpha-4-beta-7-integrin; binding prevents adhesion to ligand MAdCAM-1 which prevents T-cell adhesion; gut-specific anti-inflammatory activity reduces likelihood of infection from systemic immunosuppression | - Monitored for symptoms every 6 months for first year while on therapy - Follow-up esophagogastroduodenoscopy 7 months after initiation of intravenous vedolizumab | - Follow-up esophagogastroduodenoscopy 7 months after initiation of intravenous vedolizumab revealed significant improvement in histopathology: Healthy surface foveolar cells and return of specialized gastric glands with few plasma cells in lamina propria - Collagenous deposition remained largely unchanged despite biopsy results showing significant improvements in gastric body architecture - Deposition of type III collagen may not reverse quickly despite reducing inflammation as it is reparative response to inflammatory environment | - Presented with chronic diffuse abdominal pain, nausea, regurgitation, and early satiety - Physical examination significant for diffuse abdominal pain to deep palpation, otherwise unremarkable - Initial workup normal: Complete blood count, basic metabolic panel, inflammatory markers (erythrocyte sedimentation rate less than 3 millimeters per hour, C-reactive protein less than 0.30 milligrams per deciliter), thyroid-stimulating hormone, liver function tests (all within normal limits) - Initial esophagogastroduodenoscopy revealed significant gastric nodularity, erythema, and nodularity - Started on topical budesonide 9 milligrams daily but stopped after 2 weeks due to no significant improvement in symptoms - Repeat esophagogastroduodenoscopy showed no improvement in gastric body nodularity or subepithelial collagen deposition on biopsy - Transitioned to intravenous vedolizumab owing to persistent symptoms and lack of significant resolution on endoscopy - Reported steady improvement in abdominal pain, nausea, and vomiting 2 weeks after vedolizumab loading doses - Follow-up esophagogastroduodenoscopy 7 months after vedolizumab initiation: Significant improvement in histopathology but persistent gastric nodularity and largely unchanged collagenous deposition - Successfully managed with intravenous vedolizumab after initial therapy with topical budesonide did not result in clinical or endoscopic improvement - Continued on intravenous vedolizumab infusions every 8 weeks as maintenance therapy |
| Ref(81) | - Median age: 18.0 years (interquartile range: 12.0–36.8 years) - Bimodal age distribution: First peak between ages 10–19 years, second peak at greater than 60 years of age - From included observational studies: Population consisted of children less than 19 years of age (21.9%), younger adults 19–50 years (37.5%), and adults over 50 years (40.6%) | - Male: 36/88 (40.9%) - Female: 52/88 (59.1%) | 52/86 (60.5%) | 20/87 (23.0%) | 22/87 (25.3%); more common in adults (46% in adults over age 50) | 54/88 (61.4%); more characteristic of isolated collagenous gastritis; present in 50% of children and 40% of adults; most common presenting symptom related to anemia in 33% in one pediatric study | 6/87 (6.9%) | Weight loss 19/87 (21.8%); more in adults (62% in adults over age 50); melena 10/87 (11.5%); fever 2/87 (2.3%) | - Autoimmune hemolytic anemia: 2/87 (2.3%) - Systemic lupus erythematosus: 1/87 (1.1%) - Sjögren syndrome: 1/87 (1.1%) - Hashimoto's thyroiditis: 1/87 (1.1%) - Common variable immunodeficiency: 2/87 (2.3%) - Selective immunoglobulin M deficiency: 1/87 (1.1%) - Atopic dermatitis: 2/87 (2.3%) - Concurrent celiac disease: 4/88 (4.5%) - Predisposition to autoimmune disease: 60% in one pediatric study (9/15 patients); positive results for auto-antibodies in 40% (6/15 patients) - Positive HLA-DQ2/DQ8: 53% in one study (8/15 patients) - Association with HLA-DQ2 antigen suggested in two observational studies - Concomitant autoimmune condition: 12.5% in one pediatric study - Immunodeficiency or autoimmune condition: 16% (5/31 patients) in one study - Immunodeficiency: 5% in one pediatric study - Iron deficiency anemia: 6/87 (6.9%) - Others: 7/87 (8.0%) - None: 47/87 (54.0%) | - 53/88 (60.2%) - most common gross endoscopic finding - In one study: 77% had nodularity of any type - 75% of children and 42% of adults in another study | 23/88 (26.1%) - erythema/erosion combined | - 23/88 (26.1%) - erythema/erosion combined - One study: 1 patient had erosive gastric mucosa | - Normal appearance: 11/88 (12.5%) - Diffuse edema: 6/88 (6.8%) - Atrophy: 5/88 (5.7%) - Ulcer: 4/88 (4.5%) - Mucosal thickening: 3/88 (3.4%) - Other nonspecific findings: 2/88 (2.3%) - Unreported: 4/88 (4.5%) - Most common features: mucosal nodularity, hypertrophic gastric folds, erythema - Pseudopolyposis or cobblestone-like mucosal nodularity noted | - Concurrent *H. pylori* infection: 7/80 (8.8%) - *H. pylori* gastritis as initial diagnosis: 3/80 (3.8%) | - Subepithelial collagen band (pathological finding): 58/88 (65.9%) - Characteristic finding: collagen bands thicker than 10 micrometers - One representative case: 16.5 micrometers | - Concurrent collagenous colitis: 12/82 (14.6%) - 9 concurrent collagenous colitis in one study - 25% had concomitant collagenous colitis in another study - 7.5% associated with collagenous colitis in one study | - Subepithelial collagen band: 58/88 (65.9%) - Chronic gastritis with lymphocyte infiltrate: 33/88 (37.5%) - Eosinophilic infiltrate: 5/88 (5.7%) - Atrophy: 4/88 (4.5%) - Nondiagnostic: 4/88 (4.5%) - Nonspecific spindle cells: 1/88 (1.2%) - Unreported: 10/88 (11.7%) - Repeat esophagogastroduodenoscopy needed: 43/88 (48.9%) - Chronic inflammatory cells in lamina propria - Deposition of collagen bands >10 micrometers - Histologic variations: infiltration with lymphocytes, plasma cells, or eosinophils; atrophic pattern - 87% eosinophilic-like pattern in one study - 6% intraepithelial lymphocytic pattern in one study - 52% meeting criteria for eosinophilic gastritis in one study - 12% with lymphocytic gastritis in one study - 18% with moderate-severe atrophy-like pattern in one study - Focal superficial epithelial degenerative changes - Inflammatory cell infiltrates in lamina propria - Thickened subepithelial collagen band | - First-line treatment:   - Symptomatic management (mainly iron supplementation): 37/88 (42.0%)  - PPIs: 27/88 (30.7%)  - Clinical observation: 9/88 (10.2%)  - Prednisone: 8/88 (9.1%)  - Budesonide: 7/88 (8.0%)  - H2 blocker: 7/88 (8.0%)  - *H. pylori* eradication: 4/88 (4.5%)  - Gluten-free diet: 4/88 (4.5%)  - Dietary change: 2/88 (2.3%)  - Fiber/bowel regimen: 2/88 (2.3%)  - 5-Aminosalicylic acid: 2/88 (2.3%)  - Adalimumab and azathioprine: 1/88 (1.2%)  - Anti-helminth: 1/88 (1.2%)  - Unspecified: 7/88 (8.0%)   - Second-line treatment:   - PPIs: 12/88 (13.6%)  - Supportive management: 7/88 (8.0%)  - Budesonide: 6/88 (6.8%)  - Systemic corticosteroid: 3/88 (3.4%)  - 5-Aminosalicylic acid: 3/88 (3.4%)  - H2 blocker: 2/88 (2.3%)  - Dietary change: 2/88 (2.3%)  - Surgical intervention: 2/88 (2.3%)  - Gluten-free diet: 1/88 (1.2%)  - Azathioprine: 1/88 (1.2%)  - None: 56/88 (63.6%)   - Third-line treatment:   - Systemic corticosteroid: 5/88 (5.7%)  - PPIs: 3/88 (3.4%)  - Budesonide: 2/88 (2.3%)  - Azathioprine: 2/88 (2.3%)  - Empiric *H. pylori* eradication: 1/88 (1.2%)  - Dietary change: 1/88 (1.2%)  - None: 78/88 (88.6%)   - Response rates to different medications:   - Systemic corticosteroids: 14/16 (87.5%)  - PPIs: 34/42 (81.0%)  - Other symptomatic treatment: 33/44 (75.0%)  - Budesonide: 10/15 (66.7%)  - 5-Aminosalicylic acid: 3/5 (60.0%)  - H2 blocker: 4/9 (44.4%) | - Median follow-up duration: 16.0 months (IQR: 4.3-46.5 months) - Clinical improvement: 43/67 (64.2%) | - Not specifically detailed in pooled data - One study noted clinical and histologic improvement with treatment | - Preceding PPI use: 5/88 (5.7%) - Distribution of lesions:   - Gastric body: 34/88 (38.6%)  - Antrum: 24/88 (27.3%)  - Fundus: 16/88 (18.2%)  - Diffuse: 8/88 (9.1%)  - Duodenum: 4/88 (4.5%)  - Cardia: 1/88 (2.4%)  - Pylorus: 1/88 (1.1%)  - Unspecified: 29/88 (33.0%)   - Corpus predominant: 55% in one study - Associated collagenous duodenitis: 17.5% - Concurrent collagenous sprue: 9 cases in one study - 3 cases with both collagenous colitis and collagenous sprue - Estimated incidence: approximately 13 per 100,000 upper endoscopies - Prevalence of histopathological diagnosis: 13/100,000 - Prevalence of childhood-onset collagenous gastritis: 2.1/100,000 - Correctly diagnosed as collagenous gastritis: 52/80 (73.8%) - Misdiagnosed as:   - Iron deficiency anemia: 7/80 (8.8%)  - Collagenous colitis: 5/80 (6.3%)  - Celiac disease: 4/80 (5.0%)  - Nonspecific gastritis: 4/80 (5.0%)  - Eosinophilic esophagitis or gastritis: 3/80 (3.8%)  *- H. pylori* gastritis: 3/80 (3.8%)  - Others: 8/80 (10.0%) |
| Ref(82) | 54 | Female | Yes - epigastric pain that was sharp in nature | No coffee ground emesis mentioned | No - occasionally complained of constipation instead | Not mentioned | Not mentioned | - Heartburn - Bloating or gas - Occasional constipation - No history of weight loss - No appetite changes - No dysphagia or odynophagia - No melena - No blood in feces | - Past medical history of diabetes mellitus - No other autoimmune diseases mentioned | Not mentioned | Yes - erythema in the stomach | Non-erosive gastritis | - Esophageal hiatal hernia - Colonoscopy revealed 4 mm polyp in sigmoid colon - According to literature cited: erythematous gastritis was commonest endoscopic finding in 74% of *H. pylori* cases - Antral erosion had specificity of 92% and sensitivity of 22% with positive predictive value of 86% to *H. pylori* infection | - Positive - *H. pylori*-associated chronic active gastritis - Immunohistochemistry for *H. pylori* showed positive staining - At least 85% of patients with *H. pylori*-associated diseases exhibit presence of bacterium in antrum - Up to 15% involve body of stomach | - Increased subepithelial collagen present - Specific thickness not measured in this case - General characteristic: thick subepithelial collagen bands typically >10 micrometers - Masson trichrome stain showed subepithelial collagenous band (blue color) | - Not involved - 4 mm polyp found in sigmoid colon on colonoscopy but no collagenous colitis | - *H. pylori*-associated chronic active gastritis with increased subepithelial collagen - Scattered mixed inflammatory cells within and underneath collagen table:   - Plasma cells  - Eosinophils  - Neutrophils   - Masson trichrome stain showing subepithelial collagenous band - Immunohistochemistry for *H. pylori*: positive - Hematoxylin and eosin (H&E) staining showed chronic active gastritis associated with *H. pylori* and thickened collagen - Chronic inflammatory cells, plasma cells, and thickened collagen under gastric epithelium - Type III collagen is released to repair inflammatory damage caused by subepithelial fibroblasts - Polymorphonuclear leukocyte infiltrate in gastric mucosa | - *H. pylori* eradication therapy - Recommended 14-day course of triple therapy:   - Amoxicillin  - PPI  - Either clarithromycin or metronidazole   - Testing for eradication of *H. pylori* recommended at least 4 weeks after completion of therapy | - Patient clinically improved after treatment - Histologic findings resolved on follow-up endoscopy - Patients should be tested for *H. pylori* eradication at least 4 weeks after therapy completion - Regular follow-up with gastroenterologist crucial to monitor disease progression and management | - Histologic findings were resolved after *H. pylori* eradication therapy - Significant improvement in collagenous gastritis on endoscopy after treatment | - Physical examination initially unremarkable with soft, non-tender, non-distended abdomen - Final diagnosis: Collagenous gastritis based on clinical presentation, upper GI endoscopic findings, histopathologic analysis, and immunohistochemical evaluation - After *H. pylori* eradication treatment, patient showed:   - Clinical improvement  - Resolution of histologic findings  - Significant improvement in collagenous gastritis on endoscopy |
| Ref(83) | 16 (adolescent male) | Male | Denied abdominal pain at gastroenterology evaluation | Denied nausea or vomiting | - Stooling 3-4 times per day - Stools were not melenic - No bright red blood in stools | - Yes - Refractory iron-deficiency anemia (primary presenting feature) - Initial hemoglobin: 9.9 g/dL - Initial hematocrit: 33.2% - Initial mean corpuscular volume: 74.3 fl - Initial ferritin: 5 ng/mL - Total iron saturation: 5% - Iron level: 19 mcg/dL - Red cell distribution width: 15.2% | Not mentioned | - Fatigue (began 2-3 months before evaluation) - Dizziness - Lightheadedness - Inability to focus - Unintentional weight loss of 20 pounds over prior 6 months - History of asthma | - Family history pertinent for:   - Brother with history of Hodgkin's lymphoma  - Brother with celiac disease   - Patient had positive tissue transglutaminase (TTG) immunoglobulin A (IgA) of 4 U/mL (normal <4 U/mL)   - Likely false positive  - Patient on gluten-free diet at time of testing  - No evidence of celiac disease on histology despite obtaining 6 duodenal samples   - Had been following gluten-free diet for months since brother's diagnosis - Never undergone HLA genetic testing for celiac disease - Family deferred HLA testing | - Yes - Significantly nodular gastric body - Nodularity of gastric corpus (characteristic endoscopic finding) - Within nodular lesions, depressed mucosal pattern from inflammation and collagen deposition leading to atrophic appearance - Follow-up endoscopy at 18 months: gastric body had patchy erythema and visually improved nodularity | Follow-up endoscopy showed patchy erythema | Areas of superficial epithelial erosions noted on histology | - Grossly normal esophagus - Grossly normal duodenum - Literature notes other possible findings: bleeding, ulceration - Follow-up endoscopy at 2 months showed visual persistence of gastric nodularity | Not mentioned in this case | - Thickened subepithelial collagen bands greater than 15 micrometers - Collagen wrapped around subsurface capillaries - Dark blue subepithelial thick collagen bands noted within lamina propria on trichrome stain - Follow-up endoscopy at 2 months: reduced collagen thickening in biopsied sections - Antrum trichrome stain: normal whispy staining of subepithelial collagen, not increased | - Colonoscopy performed at 2 months was visually and histologically normal - No collagenous colitis in this patient | - Thickened subepithelial collagen bands >15 micrometers wrapped around subsurface capillaries - Trichrome stained gastric biopsies of antral and fundic mucosa - Increased lymphoplasmacytic cells within lamina propria - Lymphoid aggregates with reactive germinal centers - Dark blue subepithelial thick collagen bands within lamina propria - Areas of superficial epithelial erosions - Antrum (20×): antral type gastric mucosa with lymphoplasmacytic infiltrate in lamina propria without neutrophils in epithelium, consistent with chronic inactive gastritis - Basement membrane not thickened in antrum - Antrum trichrome stain: normal whispy staining of subepithelial collagen, not increased - Characteristic: subepithelial patches of collagen deposits and inflammatory infiltrates in gastric lamina propria usually limited to corpus - Repeat endoscopy showed visual persistence with reduced collagen thickening | - Initial treatments:   - Oral ferrous sulfate 325 mg once daily (started by primary care)  - Increased to twice daily after minimal improvement  - Still no improvement with iron supplementation alone   - Successful combination therapy:   - PPI  - Oral iron supplementation  - Continuation of gluten-free diet (patient elected)   - Response to treatment:   - Prompt improvement of symptoms  - Lab values normalized:   1. Hemoglobin increased to 14.2 g/dL 2. Ferritin increased to 28 ng/mL 3. Total iron saturation increased to 28% 4. Iron levels increased to 82 mcg/dL | - Evaluated by pediatric hematology 1 month after initial diagnosis - Minimal improvements in hemoglobin initially despite oral iron - Referred to pediatric gastroenterology - Stool evaluation obtained: hemoccult and fecal calprotectin both negative - Celiac screen obtained - Instructed to resume gluten-containing diet for 2 weeks before endoscopy - Repeat endoscopy at 2 months: visual persistence of gastric nodularity with reduced collagen thickening - Colonoscopy at 2 months: normal - Follow-up endoscopy at 18 months: visually improved nodularity with patchy erythema | - Repeat endoscopy at 2 months showed reduced collagen thickening in biopsied sections - Visual persistence of gastric nodularity | - Initial evaluation:   - Symptoms began 2-3 months before gastroenterology evaluation  - Weight loss of 20 pounds over 6 months  - Failed oral iron supplementation alone  - Minimal improvement with increased iron dosing   - Differential diagnosis considered:   - Celiac disease  - Eosinophilic gastritis  - Lymphocytic gastritis  - Autoimmune gastritis  - Inflammatory bowel disease  - *H. pylori* gastritis   - Diagnostic workup:   - Stool hemoccult: negative  - Fecal calprotectin: negative  - Celiac screen: positive TTG IgA (likely false positive)  - Endoscopy: significantly nodular gastric body  - Histology: diagnostic of collagenous gastritis  - No celiac disease on histology despite 6 duodenal samples   - Final diagnosis:   - Collagenous gastritis (confirmed by biopsy, endoscopic findings, symptomatology, and response to treatment)  - Iron deficiency secondary to collagenous gastritis   - Treatment response:   - Prompt improvement with PPI + iron supplementation + gluten-free diet  - Lab normalization  - Visual improvement at 18 months  - Reduced collagen thickening at 2 months |
| Ref(84) | 26 | Female (woman) | Recurrent upper abdominal distention (chief complaint for 3 years)  Upper abdominal discomfort | Not mentioned | Not mentioned in this case | - Yes - Recurrent anemia (chief complaint for 3 years) - Anemic appearance on physical examination - Hemoglobin: 86 g/L - Hematocrit: 0.26 L/L - Mean corpuscular volume: 77.7 fl - Mean corpuscular hemoglobin: 25.5 pg - Mean corpuscular hemoglobin concentration: 329.0 g/L - Ferritin: 2.6 ng/mL - White blood cells: 4.63 × 10⁹/L - Platelet: 203 × 10⁹/L - Hypoferric anemia (iron-deficiency anemia as main symptom) | Not mentioned | No other symptoms specifically mentioned | - No autoimmune diseases mentioned in this case - Liver and kidney functions: normal - Tumor markers: normal - Coagulation function: normal - Thyroid function: normal - Immunoglobulin G4: normal - Autoantibodies: normal - No history of allergy | - Yes - Multiple island residual mucosa and diffuse nodular mucosa observed in corpus ventriculi (gastric body) - Localized nodular eminences noticed in greater curvature of corpus ventriculi | Not mentioned | Not mentioned | - Mucosa around nodules was concave and atrophic - Multiple island residual mucosa with surrounding atrophic changes - Enhanced computed tomography scans for whole abdomen: no abnormality - Endoscopic ultrasonography (12-megahertz ultrasound):   - Clear structure with approximately 6.3-millimeter-thick gastric wall  - Thickness of mucosa and submucosa increased  - Mucosa approximately 2.2-millimeter thick | Negative - Test for *H. pylori* was negative | - 17.68 to 35.73 micrometers thick - Collagen band in superficial mucosa showed patchy distribution - Tested positive for Masson staining - Formation of belt hyperplasia of collagen in superficial mucosa | - Colonoscopy did not show any obvious abnormality - No collagenous colitis in this case | - Subepithelial collagen band 17.68 to 35.73 micrometers thick - Positive for Masson staining - Formation of belt hyperplasia of collagen in superficial mucosa - Infiltration of inflammatory cells - Gastric mucosa showed atrophic changes with atrophic and flat pits - Number of oxyntic glands in corpus ventriculi reduced - Hematoxylin and eosin staining: lamina propria infiltrated with lymphocytes, plasma cells, and eosinophils - Masson staining: collagen band in superficial mucosa showed patchy distribution - Congo red staining: negative - Characteristic pathology: thickening of subepithelial collagen band to more than 10 micrometers along with infiltration by inflammatory cells - Subepithelial layer infiltrated with chronic inflammatory cells (lymphocytes, plasma cells, eosinophils), especially in lamina propria | - Polysaccharide iron complex capsule: 0.3 grams three times a day, oral administration - Omeprazole capsule: 20 milligrams once daily, oral administration - Treatment duration: 8 weeks | - 8-week treatment follow-up - Symptoms ameliorated (upper abdominal distention and anemia) - Blood routine showed hemoglobin level rose to 91 grams per liter | Not performed/not mentioned | - Initial presentation:   - Chief complaint: recurrent upper abdominal distention and anemia for 3 years  - Physical examination: anemic appearance, flat and soft stomach, liver and spleen not palpable  - Denied any history of allergy   - Diagnostic workup:   - Blood routine showing iron-deficiency anemia  - Liver and kidney functions, tumor markers, coagulation function, thyroid function, Immunoglobulin G4, autoantibodies: all normal  - Enhanced computed tomography scans: no abnormality  - Gastroscopy: diffuse nodular mucosa in corpus ventriculi  - Endoscopic ultrasonography: thickened mucosa and submucosa  - Colonoscopy: no obvious abnormality  - Biopsy pathology: confirmed collagenous gastritis  - *H. pylori* test: negative   - Final diagnosis:   - Collagenous gastritis confirmed by:   1. Clinical manifestations 2. Endoscopic findings 3. Pathological features (subepithelial collagen band 17.68 to 35.73 micrometers thick with inflammatory cell infiltration)  - Treatment response:   - 8-week treatment with iron supplementation plus PPI  - Symptoms ameliorated  - Hemoglobin improved from 86 grams per liter to 91 grams per liter |
| Ref(85) | - 9 years old (at diagnosis of collagenous gastritis) - 2 years old (at diagnosis of collagenous colitis) | Female (girl) | - Epigastric pain (started at age 9 years) - Intermittent colicky abdominal pain (during follow-up from age 2) - Increased abdominal cramps (with mesalazine treatment) | Not mentioned | - Yes - Persistent watery diarrhea (main symptom from age 2) - Initially resolved with antibiotic treatment - Two episodes in following year with isolation of Aeromonas hydrophila - Watery diarrhea subsequently reappeared - Follow-up characterized by persistent watery diarrhea - Reappeared 1 month after discontinuing budesonide | - Yes - Presented at age 9 years - Pale appearance on physical examination - Hemoglobin level: 9.5 grams per deciliter - Ferritin: 2.71 nanograms per milliliter - Erythrocyte sedimentation rate: 34 millimeters per hour | Not mentioned | - Weight and height at 10th and 3rd percentiles for age, respectively - Pale appearance - Intermittent colicky abdominal pain (from age 2) | - Congenital cardiac defect - Serum Immunoglobulin A antiendomysial antibodies: negative - Immunoglobulin A antitransglutaminase antibodies: negative - Antinuclear antibodies: negative - Antimitochondrial antibodies: negative - Rheumatoid factor: negative - Perinuclear anti-neutrophil cytoplasmic antibodies: negative - Cytoplasmic anti-neutrophil cytoplasmic antibodies: negative - Serum electrolytes, glucose, creatinine, urea, transaminases, albumin and immunoglobulins: within normal limits | - Yes - Nodularity of gastric corpus and antrum (at age 9) - Literature notes: gastric nodularity frequent finding in collagenous gastritis (11 children, 7 adults) - Only reported in 2 adult patients with collagenous colitis and collagenous gastritis | Not mentioned in this case | Not mentioned in this case | - Normal duodenum - Normal esophagus - Initial upper gastrointestinal endoscopy at age 2: normal (gastric biopsies not taken) - Flexible sigmoidoscopy: normal at age 9 - Colonoscopy on 2 occasions: colonic findings at endoscopy unchanged | Negative - *H. pylori* was not detected | - Not specifically measured in micrometers in this case report - Masson trichrome staining showed thickened subepithelial collagenous band - Moderate chronic inflammatory infiltrate - Striking subepithelial and interlacing bundles of thick collagen fibers between the atrophic glands | - Colonic mucosal biopsies demonstrated typical features of collagenous colitis (at age 2 and age 9) - Subepithelial collagen band present - Specific thickness not mentioned | - Stomach (at age 9):   - Moderate-intense glandular atrophy  - Moderate chronic inflammatory infiltrate without signs of activity  - Striking subepithelial and interlacing bundles of thick collagen fibers between the atrophic glands  - Masson trichrome staining: thickened subepithelial collagenous band  - *H. pylori* not detected   - Colon:   - Typical features of collagenous colitis  - Initially: collagenous colitis diagnosed at age 2  - At age 9: collagenous colitis persisted  - Colonic findings at endoscopy and histology unchanged on 2 occasions   - Characteristic pathology:   - Deposition of subepithelial collagen band in mucosa  - Initially diagnosed as collagenous colitis at age 2  - Collagenous gastritis diagnosed at age 9 | - Initial treatment (age 2-6):   - Antibiotics (for Aeromonas hydrophila)  - Stool culture 1 month later: negative for bacterial pathogens  - Bismuth for 7 weeks: partial and temporary improvement  - Loperamide: not mentioned in this case but mentioned in literature review   - Age 6:   - Oral budesonide: 9 milligrams per day for 6 months  - Complete clinical improvement observed  - Diarrhea reappeared 1 month after discontinuing medication   - Subsequent treatment:   - Oral mesalazine: 50 milligrams per kilogram per day  - Diarrhea continued  - Increased abdominal cramps reported  - Discontinued one month later   - Age 9 (after collagenous gastritis diagnosis):   Not specifically mentioned in case report | - Age 2-9 years:   - Symptoms persisted during following years after initial diagnosis  - Routine blood parameters: normal  - Erythrocyte sedimentation rate: mildly elevated  - Colonoscopy on 2 occasions: colonic findings at endoscopy and histology unchanged  - Multiple stool cultures during follow-up: sterile  - Aeromonas hydrophila not isolated again after initial episodes  - Complete clinical improvement with budesonide at age 6  - Diarrhea reappeared 1 month after discontinuing budesonide  - Mesalazine trial unsuccessful   - Age 9:   - Started with epigastric pain  - Weight and height at 10th and 3rd percentiles  - Hemoglobin: 9.5 grams per deciliter  - Ferritin: 2.71 nanograms per milliliter  - Upper gastrointestinal endoscopy revealed gastric nodularity  - Diagnosed with collagenous gastritis | - Colonic findings at endoscopy and histology unchanged on 2 occasions during follow-up (age 2-9) - Typical features of collagenous colitis persisted at age 9 | - Initial presentation (age 2):   - Watery diarrhea  - Aeromonas hydrophila isolated in feces  - Diarrhea resolved gradually with antibiotic treatment  - Stool culture 1 month later: negative for bacterial pathogens  - Upper gastrointestinal endoscopy: normal (gastric biopsies not taken)  - Diagnosed with collagenous colitis at age 2   - Subsequent course (age 2-6):   - Following year: 2 episodes of watery diarrhea with isolation of Aeromonas hydrophila, resolved with antibiotics  - Third episode: treated with bismuth for 7 weeks, partial and temporary improvement  - Watery diarrhea subsequently reappeared  - Follow-up characterized by persistent watery diarrhea and intermittent colicky abdominal pain  - Routine blood parameters: normal  - Erythrocyte sedimentation rate: mildly elevated  - Colonoscopy on 2 occasions: unchanged findings   - Age 6:   - Oral budesonide 9 milligrams per day for 6 months  - Complete clinical improvement  - Diarrhea reappeared 1 month after discontinuing medication  - Oral mesalazine trial: unsuccessful, discontinued due to continued diarrhea and increased abdominal cramps   - Age 6-9:   - Symptoms persisted  - Multiple stool cultures: sterile  - Aeromonas hydrophila not isolated again   - Age 9:   - Started with epigastric pain  - Pale appearance, weight and height growth percentiles low  - Anemia present (hemoglobin 9.5 grams per deciliter, ferritin 2.71 nanograms per milliliter)  - Upper gastrointestinal endoscopy: nodularity of gastric corpus and antrum  - Gastric biopsies: collagenous gastritis  - Flexible sigmoidoscopy: normal  - Colonic biopsies: typical features of collagenous colitis persisted  - Diagnosis: both collagenous colitis and collagenous gastritis |
| Ref(86) | Male in his 50s | - Male - Non-Hispanic white | Mild left upper quadrant abdominal tenderness to percussion | Not reported | Denied | Iron deficiency without microcytic anemia (ferritin 10.2 ng/mL) | Denied | - 10-kg weight loss over 3 months - Diffuse leg cramps - New intolerance to spicy foods - Fatigue - Previously tested positive for COVID-19 approximately 3 months prior to presentation | - None specifically mentioned - No family history of autoimmune disorders | Not reported | Diffuse gastritis observed on esophagogastroduodenoscopy | Not reported | - Normal colonic mucosa on colonoscopy - Previous diagnosis of collagenous gastritis and colitis confirmed on direct mucosal biopsy in 2019 | Negative (*H. pylori* stool antigen testing performed 6 months prior to presentation) | Not specified in this case (previous diagnosis confirmed in 2019, but measurements not provided) | Not specified (previous diagnosis confirmed in 2019, but measurements not provided) | - Diffuse gastritis confirmed on upper endoscopy - Previous diagnosis of collagenous gastritis and colitis based on direct mucosal biopsy in 2019 - No specific histological details provided for current presentation | - Ferrous sulfate 325 mg daily for iron deficiency - Oral budesonide 9 mg daily for 4 weeks with 4-week taper - Prophylactic famotidine therapy during budesonide treatment - Omeprazole 40 mg daily (PPI therapy) | - Leg cramps resolved with 3 months of ferrous sulfate supplementation - Minimal improvement after 4-week course of oral budesonide - Currently improving with ongoing PPI therapy and ferrous sulfate supplementation - Steady but slow weight gain | - Esophagogastroduodenoscopy and colonoscopy performed at end of 2022 - Showed diffuse gastritis - Normal colonic mucosa - No specific comparison to previous histology provided | - Initial diagnosis of collagenous gastritis and colitis in 2019, treated with oral steroids and sucralfate with symptom improvement - COVID-19 infection approximately 3 months before current presentation - Developed weight loss, leg cramps, and food intolerance - EBV early antigen IgG positive, suggesting possible EBV reactivation - Minimal response to budesonide therapy - Ongoing improvement with acid suppression therapy (omeprazole) and iron supplementation - Authors hypothesize COVID-19-induced EBV reactivation may have contributed to recurrence of collagenous gastritis |
| Ref(87) | 63 | Female | Not reported | Recurrent vomiting for 6 months | Not reported | Normal complete blood count | Not reported | - Weight loss of 5 kg over 6 months - Generally unwell and underweight - BMI 16 kg/m² | - Antinuclear antibody titre elevated at 1:160 (normal <1:40) - Autoimmune gastritis considered in working diagnosis but not confirmed - No history of gastrointestinal disorders | Not reported | Rough atrophic mucosa | Mucosa bled easily upon contact | - Distended stomach - Linear scars - Substantial improvement in mucosal inflammation on repeat OGD 6 months after olmesartan discontinuation | Negative (no history of *H. pylori* infection) | - Markedly thickened subepithelial collagen bands (specific measurements not provided) - No evidence of residual collagen bands on follow-up biopsy 6 months after drug discontinuation | Small and large intestines showed no specific findings | - Patchy subepithelial collagen deposition - Inflammatory cell infiltration including eosinophils - Stromal fibrotic changes - On follow-up (6 months post-discontinuation): no evidence of residual collagen bands or significant inflammatory cell infiltration | - Initially prescribed PPI (esomeprazole 20 mg orally) - no improvement - Olmesartan 20 mg daily discontinued (patient had been taking for past decade) - Azelnidipine 8 mg daily prescribed as alternative treatment for hypertension | - 2 weeks post-discontinuation: vomiting stopped - 2 months post-discontinuation: gained 7 kg - 6 months post-discontinuation: repeat OGD showed substantial improvement - 2 years after discontinuation: no recurrence | - 6 months post-discontinuation of olmesartan: - No evidence of residual collagen bands - No significant inflammatory cell infiltration - Complete histological resolution | - 6-month history of symptoms while on olmesartan (taken for decade) - Initial treatment with PPI ineffective - Olmesartan discontinued based on suspicion of drug-induced gastritis - Rapid clinical improvement within 2 weeks - Complete weight recovery within 2 months - Complete endoscopic and histological resolution at 6 months - No recurrence at 2 years follow-up - Drug-induced collagenous gastritis (olmesartan) with complete resolution after discontinuation |
| Ref(88) | 13 | Female | 2-month history of epigastric pain | Not reported | Not reported | - Severe anemia (hemoglobin 4 g/dL) - Required red cell concentrate transfusion | Not reported | - Orthostatic dizziness - Headache - Hair loss - Nail fragility - Tachycardia - Marked mucocutaneous pallor | - No previous medical history - No associated gastrointestinal or autoimmune pathology | Not reported | Not reported | Not reported | Diffuse pallor of the gastric chamber | Not mentioned | Subepithelial collagen deposits greater than 10 µm | Not evaluated | - Subepithelial collagen deposits greater than 10 µm - Finding confirmed by Masson's trichrome stain | - PPIs - Oral iron therapy (OIT) | - Currently asymptomatic and under follow-up - No new EGD controls performed to date | No follow-up endoscopy or biopsy performed | - Presented with severe anemia and epigastric pain - Favorable response to treatment with OIT and PPIs - Currently asymptomatic - Pattern: Patient without associated autoimmune pathology with favorable clinical response |
| Ref(88) | 8 years and 7 months | Male | Abdominal pain | Not reported | Not reported | - Severe anemia (hemoglobin 5.1 g/dL) - Resolution of anemia after treatment | Not reported | Asthenia | No associated gastrointestinal or autoimmune pathology | Control EGD (1 year after diagnosis): diffuse nodular pattern | Not reported | Not reported | - Initial EGD: marked mucosal friability and scattered fibrin plaques at gastric body level - Control EGD: diffuse nodular pattern with mucosal friability | Not mentioned | - Subepithelial collagen deposits (specific measurement not provided) - Persistence of subepithelial collagen deposits on 1-year follow-up | Not evaluated | - Initial biopsy: subepithelial collagen deposits - Follow-up biopsy (1 year): chronic active gastritis, persistence of subepithelial collagen deposits, intramucosal eosinophil infiltration (5 eosinophils per field) | - Oral iron therapy (OIT) - PPIs - 99m-Tc scintigraphy performed (normal result) | - Adequate clinical and analytical response - Resolution of anemia - EGD control performed 1 year after diagnosis - Currently asymptomatic and under follow-up | - 1 year after diagnosis: - Chronic active gastritis - Persistence of subepithelial collagen deposits - Intramucosal eosinophil infiltration (5 eosinophils per field) | - Presented with abdominal pain, asthenia, and severe anemia - Favorable response to treatment with OIT and PPIs - Currently asymptomatic despite persistence of histological findings - Pattern: Patient without associated autoimmune pathology with favorable clinical response |
| Ref(88) | 11 | Female | - Chronic diffuse abdominal pain - Persistence of intermittent discomfort despite treatment | Not reported | Not reported | Iron deficiency anemia refractory to medical treatment | Not reported | Weight-status stagnation | - Autoimmune thyroiditis diagnosed in following years - Positive anti-nuclear antibodies (ANA) - Positive anti-gastric parietal cell antibodies - Positive anti-extractable nuclear antigen (ENA) anti-Sm - Positive anti-peroxidase antibodies - Positive antithyroglobulin antibodies - HLA-DQ2 homozygotic carrier - Negative celiac disease serology | - Initial EGD: marked diffuse nodularity at gastric level - Control EGD (6 months): marked patchy gastric nodularity | Not reported | Not reported | - Control EGD: mucosal friability - Colonoscopy: no macroscopic alterations - Progressive worsening of EGD involvement during follow-up despite continued medical treatment | - Presence not confirmed histologically - Cocci identified on mucosal surface at Giemsa staining (no bacilli identified) | - Subepithelial collagen deposits (specific measurement not provided) - Persistence of subepithelial collagen deposits on 6-month follow-up | Colonoscopy performed - no alterations observed in colonic biopsies | - Initial biopsy: subepithelial collagen deposits - Follow-up biopsy (6 months):   - Persistence of subepithelial collagen deposits  - Focal complete intestinal metaplasia at fundus level  - Cocci on mucosal surface at Giemsa staining  - No colonic alterations | - PPIs - incomplete clinical response - Oral iron therapy (OIT) - worse response compared to cases 1 and 2 - Oral budesonide for 6 months - no clinical improvement, subsequently suspended - Operated for acute appendicitis 1 month after diagnosis (appendectomy specimen showed acute polymorphonuclear infiltrate without other alterations) | - EGD control performed 6 months after diagnosis - Diagnostic colonoscopy performed at same time as control EGD - Currently under follow-up - Persistence of intermittent discomfort despite treatment | 6 months after diagnosis:   - Persistence of subepithelial collagen deposits - Development of focal complete intestinal metaplasia at fundus level - Progressive worsening of EGD involvement | - Presented with chronic diffuse abdominal pain, weight stagnation, and refractory iron deficiency anemia - Developed autoimmune thyroiditis during follow-up - Poor response to corticosteroid treatment and PPIs - Worse response to OIT compared to other cases - Progressive worsening of endoscopic findings despite treatment - Development of intestinal metaplasia - Pattern: Patient with associated autoimmune pathology with poor treatment response and progressive disease - No correlation observed between thickness of deposits and clinical severity in this series |
| Ref(89) | 41 | Female | - Epigastric pain unrelated to food intake - Umbilical pain (improved with treatment) | Nausea (improved with treatment) | - Diarrhea twice daily without blood for the past 10 years - Diarrhea disappeared with treatment | Not reported | Not reported | - Heartburn - Flatulence | - Thyroid nodules with normal thyroid function without presence of autoantibodies - Stage II atrophic gastritis according to OLGA classification - *H. pylori*-negative gastritis with atrophic changes mainly in body area, suggesting autoimmune origin - No family history of immune-mediated disorders | Not specifically mentioned as nodular | - Mucosa focally hyperemic in body of stomach - Antrum: mucosa focally hyperemic | Multiple hemorrhagic erosions up to 0.2 cm in diameter in antrum distributed around entire perimeter | - Diffusely thinned mucosa with multiple flat areas of intact mucosa appearing as islands up to 0.5 cm in diameter in body of stomach - Antrum: focally thinned mucosa - Colonoscopy: mucosa in transverse, descending, and sigmoid colon edematous with whitish tint, areas of granular appearance; capillary pattern blurred and sometimes not visible - Other colon sections: mucosa pale pink with regular capillary pattern | *H. pylori*-negative | - Subepithelial collagen deposits exceeding 10 microns (specific measurement not provided) - Red staining of subepithelial deposits with picrosirius red | Focal thickening of subepithelial layer with collagenization reaching thickness up to 28 µm | - Stomach:   - Surface deformation with collagenization of subepithelial stroma  - Picrosirius red staining revealed red staining of subepithelial deposits  *- H. pylori*-negative gastritis with atrophic changes mainly in body area   - Colon:   - Smooth surface with evenly distributed crypts  - Focal thickening of subepithelial layer with collagenization (up to 28 µm)  - Increased density of cellular infiltrate in lamina propria of mucosa  - Multifocal biopsies from ascending, transverse, descending, sigmoid, and rectum | - Oral Budesonide 9 mg daily for 8 weeks - Maintenance therapy with lowest effective dose of budesonide: 6 mg daily for 12 months | - Clinical improvement achieved - Disappearance of diarrhea - Reduction of umbilical pain and nausea - No adverse events reported - On maintenance therapy for 12 months | No follow-up biopsy mentioned | - 10-year history of diarrhea before diagnosis - Previous colonoscopy one year ago was normal with no histological pathology - Endoscopy repeated due to 1-month persistent complaints - Confirmed diagnoses: collagen gastritis and microscopic collagen colitis - Favorable response to corticosteroid treatment (budesonide) - Successful maintenance therapy with complete resolution of diarrhea - Combined autoimmune gastrointestinal pathology demonstrated favorable response to corticosteroid treatment - Authors note no correlation observed between thickness of deposits and clinical severity |
| Ref(90) | Mean age: 10.5 years (overall cohort mean 9.07±5.04 years) | - 5 females (45.5%) - 6 males (54.5%) | Gastrointestinal symptoms (including abdominal pain) present in 7 (63.6%) | Included in gastrointestinal symptoms (7/11, 63.6%) | Not specifically reported for collagenous gastritis group | - Iron deficiency anemia present in 9 (81.8%) - Mean hemoglobin level: 7.9 g/dL | Not reported | Growth delay: 1 (9%) | - Celiac disease diagnosed in 2 (18%) - Study notes: signs of autoimmune predisposition were frequent, but early development of autoimmune comorbidities occurred rarely | - Nodularity present in 8/11 (72.7%) - significantly more prevalent than in lymphocytic gastritis (p=0.005) - Often seen in both antrum and corpus - In some cases, nodules were extremely large mimicking polyps (pseudo-polyps) | Not specifically reported | Not specifically reported | - None had normal-appearing mucosa (0/11) - All collagenous gastritis patients had abnormal gastric mucosa | Positive in 1 (9%) | - Characterized by thick sub-epithelial collagen bands (greater than 10 μm) - Trichrome staining highlights thickening of sub-epithelial basement membrane | Not evaluated (disease usually limited to stomach in children) | - Thick sub-epithelial collagen bands (greater than 10 μm) - Associated inflammatory infiltrate of gastric mucosa - Chronic inflammation present in lamina propria - Surface epithelial damage of varying degrees | - Iron supplementation: intravenous and/or oral (specific numbers not separated by group) - PPIs for gastrointestinal symptoms - *H. pylori* eradication in 1 patient - Gluten-free diet in 2 patients with celiac disease | - Mean follow-up time: 2.9±2.2 years - Hemoglobin levels normalized in 9/11 (though specific number not clearly stated, overall 21/22) - Some patients required repeat iron supplementation | - 6/11 had repeat endoscopy - Histopathological findings remained unchanged in all 6 cases - Persistent sub-epithelial collagen deposition despite clinical improvement | - Despite persistent histological findings, most patients showed remission or resolution of clinical symptoms - Anemia responsive to iron supplementation - Recurrence of anemia after stopping iron supplementation occurred in some patients - Chronic disease course - Short-term prognosis good - Most patients show clinical improvement despite unchanged histopathology - Higher rate of gastrointestinal symptoms compared to lymphocytic gastritis (though not statistically significant) |
| Ref(91) | 15 | Male | - Epigastric pain (persistent) - Diffuse abdominal pain | Not reported | Not reported (no chronic diarrhea typical of adult-onset collagenous gastritis) | - Severe iron deficiency anemia - Initial hemoglobin 6.8 grams/deciliter (normal 12-16.9 grams/deciliter) - Hematocrit 21.5% - Mean corpuscular volume 65.8 femtoliters - Required two units packed red blood cells transfusion | Not reported | - Dizziness - Pallor - Palpitations - Fatigue - No obvious rectal bleeding | - Selective immunoglobulin A deficiency without history of chronic infections - Growth hormone deficiency (managed with growth hormone therapy for 6 years) - Family history of autoimmunity: mother with psoriatic arthritis, maternal grandfather with psoriasis - Negative human leukocyte antigen DQ2 and DQ8 - Negative immunoglobulin G celiac screen (normal tissue transglutaminase immunoglobulin G, endomysial antibody immunoglobulin G, gliadin immunoglobulin G) - Immunoglobulin A-deficient patients are 10 to 20 times more likely to develop autoimmune response to gluten | - Gastric antrum, body, and fundus: diffuse nodularity - Duodenal bulb: diffuse nodularity | - Gastric antrum, body, and fundus: diffuse erythema - Duodenal bulb: diffuse erythema - Proximal jejunum (pill cam): diffuse erythema | - Gastric antrum, body, and fundus: scattered superficial erosions - Erosive nodular gastritis | - Initial esophagogastroduodenoscopy (outside provider): erosive nodular gastritis of antrum, body, and fundus, nodularity of duodenal bulb - Repeat esophagogastroduodenoscopy: gastric antrum, body, and fundus with diffuse swelling; duodenal bulb with diffuse swelling, fissured appearance, flattened villi, few aphthous ulcerations - Second and third portions of duodenum: normal - Pill cam of proximal jejunum: diffuse swelling, villous blunting - Entire colon and terminal ileum: normal - After 8 weeks of treatment: mild endoscopic improvement in gastric antrum, body, and fundus with decreased nodularity and erythema; duodenal bulb remained unchanged - Narrow band imaging of gastric body: no changes in mucosal surface of nodular lesions; depressed mucosa surrounding nodular lesions showed amorphous or absent surface structure and abnormal capillary vessels | Negative *H. pylori* | - Thickened subepithelial collagen layer (greater than 10 μm) - Positive trichrome stain for subepithelial collagen deposition - Gastric antrum, body, and fundus all showed thickened subepithelial collagen layer | Normal (no collagenous colitis) | - Initial biopsy (outside provider):   - Limited to antrum and second part of duodenum  - Mild active gastritis  - Nonspecific chronic duodenitis   - Repeat biopsy (at study center):   - Gastric antrum: active chronic gastritis, 40 eosinophils per high-powered field in lamina propria, thickened subepithelial collagen layer, positive trichrome stain  - Gastric body: active chronic gastritis, 47 eosinophils per high-powered field in lamina propria, thickened subepithelial collagen layer  - Gastric fundus: 15 eosinophils per high-powered field in lamina propria, thickened subepithelial collagen layer  - Duodenal bulb: 45 eosinophils per high-powered field in lamina propria, thickened subepithelial collagen layer, villous blunting, positive trichrome stain  - Mononuclear inflammatory infiltrate   - After 8 weeks of budesonide treatment:   - Gastric body: 30 eosinophils per high-powered field in lamina propria, thickened subepithelial collagen layer, positive trichrome stain  - Duodenal bulb: 70 eosinophils per high-powered field in lamina propria (increased), thickened subepithelial collagen layer, villous blunting, positive trichrome stain  - Virtually unchanged histopathology despite clinical improvement | - Initial treatment (outside provider):   - Two units packed red blood cells transfusion  - Two intravenous iron sucrose infusions  - Sucralfate  - Omeprazole   - Treatment at study center:   - Crushed budesonide extended-release 9 milligrams mixed with water once daily given at bedtime  - Omeprazole 40 milligrams twice daily  - After 8 weeks: significant improvement in abdominal pain and anemia  - Budesonide weaned to 6 milligrams for 4 weeks, then 3 milligrams for 4 weeks  - Patient ultimately stopped budesonide, continued omeprazole 40 milligrams once daily  - Two ferric carboxymaltose injections (due to development of intolerance to intravenous iron sucrose)  - Gluten-free diet discussed with patient  - Total of 5 intravenous iron sucrose infusions before switch to ferric carboxymaltose | - After 8 weeks of budesonide 9 milligrams:   - Significant improvement in abdominal pain and anemia  - Hemoglobin 13.2 grams/deciliter (improved from 6.8)  - Hematocrit 43.4%  - Iron 61 micrograms/deciliter  - Total iron binding capacity 399 micrograms/deciliter  - Iron saturation 15  - Ferritin 16.60 nanograms/milliliter  - Mild endoscopic improvement but virtually unchanged histopathology   - After 4 weeks of budesonide 3 milligrams:   - Hemoglobin 11.4 grams/deciliter  - Iron 34 micrograms/deciliter  - Total iron binding capacity 440 micrograms/deciliter  - Iron saturation 8  - Ferritin 2 nanograms/milliliter   - After stopping budesonide:   - Anemia worsened  - Required additional iron supplementation (ferric carboxymaltose)  - Currently undergoing immunological work-up  - Repeat esophagogastroduodenoscopy planned after patient has been off growth hormone therapy for at least 6 months | After 8 weeks of budesonide 9 milligrams:   - Gastric body: decreased eosinophils from 47 to 30 per high-powered field, but persistent thickened subepithelial collagen layer - Duodenal bulb: increased eosinophils from 45 to 70 per high-powered field, persistent thickened subepithelial collagen layer and villous blunting - Overall virtually unchanged histopathology despite clinical improvement - Persistent endoscopic abnormalities with some improvement | - Timeline:   - Two months prior to initial presentation: diagnosed with strep throat  - Over following 2 months: developed abdominal pain, dizziness, pallor, palpitations  - Initial presentation: severe anemia requiring transfusion and intravenous iron  - First esophagogastroduodenoscopy: limited biopsies missed diagnosis  - Two months later: re-evaluation at study center  - Repeat comprehensive endoscopy and pill cam: diagnosis of collagenous gastroduodenitis with suspected jejunal involvement  - 8 weeks of treatment: significant clinical improvement despite minimal histologic change  - After stopping budesonide: recurrence of anemia   - Disease characteristics:   - Diagnosis: collagenous gastroduodenitis with suspected jejunal involvement  - Represents overlap between pediatric and adult phenotypes  - Pediatric phenotype typically: confined to stomach, presents with abdominal pain and anemia  - Adult phenotype typically: involves small and/or large intestine, presents with chronic non-bloody diarrhea and weight loss  - Now acknowledged that adult-onset and pediatric-onset collagenous gastritis should be considered similar disease on continuous spectrum  - Case demonstrates profound anemia without obvious rectal bleeding   - Proposed pathogenesis in this case:   - Hypothesis: compromise to gastric and duodenal mucosa from strep throat two months prior, with resulting collagen deposition from patient's growth hormone therapy  - Growth hormone important for tissue remodeling, extracellular matrix formation, and fibrosis  - Growth hormone causes rise in matrix collagen synthesis   - Diagnostic considerations:   - First endoscopy with limited biopsies missed diagnosis  - Emphasizes importance of multiple biopsies from multiple areas  - Recommended biopsy protocol: 8 biopsies from stomach (2-fundus, 2-body, 4-antrum), 6 biopsies from duodenum/bulb (4-duodenum, 2-duodenal bulb)  - Narrow band imaging findings: no changes in mucosal surface of nodular lesions; changes found in depressed mucosa surrounding nodular lesions indicating depressed mucosal pattern results from inflammation with atrophic changes and collagen deposition, whereas nodular lesions are remaining undamaged mucosa  - Proper sampling from multiple areas and biopsy of depressed mucosal pattern maximizes diagnostic sensitivity   - Treatment response:   - Most promising therapy for collagenous gastritis: topically targeted budesonide  - Resolution of symptoms and anemia with treatment  - Mixed results in endoscopic appearance on repeat endoscopy  - Histopathologic reversal difficult to substantiate owing to focal, sometimes extensive nature of pathologic process  - This case: significant clinical improvement but virtually unchanged histopathology  - Recurrence of anemia after stopping budesonide |
| Ref(92) | 16 | Female | Yes - daily moderate and intermittently severe sharp lower abdominal pain in the mornings or an hour after meals; decreased to every other day frequency on follow-up | Mild nausea occasionally associated with abdominal pain | No | Yes - iron-deficiency anemia for 4.5 years starting at 11 years old (hemoglobin 7.8 g/dL); largely improved with iron supplementation; resolved at presentation (hemoglobin 14.5 g/dL) | Not reported | History of multiple years of episodic abdominal pain; menorrhagia; some improvement with limiting lactose in diet and using Lactaid | - None identified - Tissue transglutaminase Immunoglobulin A negative (celiac disease ruled out) - No autoimmune gastritis or lymphocytic gastritis identified | Yes - significant antral nodularity | Not reported | Not reported | One small antral polyp removed by forceps; otherwise macroscopically normal; ileocolonoscopy otherwise macroscopically normal | Negative - stool antigen negative (tested twice); Gram stain and culture of gastric antral biopsy negative | Thickened subepithelial collagen band (specific measurement ≥10 μm based on diagnostic criteria mentioned, exact measurement not provided) | Normal - no collagenous colitis identified | - Gastric antral and body mucosa showed thickened subepithelial collagen band - Increased intraepithelial lymphocytes - Surface epithelial damage with denudation, detachment, and atrophy - Lamina propria revealed prominent chronic inflammation with abundant lymphocytes, plasma cells, and many eosinophils - No iron deposits identified - 12 colonic biopsies (two each from cecum, ascending, transverse, descending, sigmoid, and rectum): microscopically normal - Eight duodenal biopsies: microscopically normal - Three terminal ileum biopsies: microscopically normal - Six esophageal biopsies: microscopically normal | - Iron supplementation discontinued at follow-up - Planned treatment with budesonide and fish oil | - Patient reported decreased frequency of abdominal pain from daily to every other day without intervention - Plan to trend fecal calprotectin with possible further investigation using video capsule endoscopy | Not available - no repeat biopsies reported | - History of chronic iron-deficiency anemia for 4.5 years (starting premenarche) - Anemia largely improved with 1 year of 130 mg/day elemental iron despite developing menorrhagia - At presentation: resolved anemia with normal hemoglobin, mean corpuscular volume, and iron levels - Persistently elevated fecal calprotectin (507 and 796 mcg/g) - Spontaneous improvement in abdominal pain frequency without intervention - Authors suspect a waxing and waning course like other chronic disorders |
| Ref(93) | Median age: 60 years (range 8–88 years) | - Female: 145 patients (71.1%) - Male: 59 patients (28.9%) - Odds Ratio for being Female: 1.96 (95% Confidence Interval 1.44–2.65) | 54 patients (26.5%) | 21 patients (10.3%) | 37 patients (18.1%) - significantly associated (Odds Ratio 2.78; 95% Confidence Interval 1.94–3.97) | 20 patients (9.8%) | Not reported | Dysphagia: 24 patients (11.8%); Dyspepsia: 15 patients (7.4%); Weight loss: 21 patients (10.3%) - significantly associated (Odds Ratio 2.35; 95% Confidence Interval 1.50–3.69) | - Microscopic colitis (collagenous and lymphocytic): found in 21.2% of subjects who had collagenous gastritis and same-day colonoscopy (Odds Ratio 18.56; 95% Confidence Interval 13.40–25.73) - Strong association with other lymphocytic conditions of the gastrointestinal tract | Not specifically reported | Ubiquitous (described as erythematous gastric mucosa) | 6.9% had erosions or ulcers (not statistically significant compared to controls) | Edematous gastric mucosa was ubiquitous; no distinct or characteristic endoscopic features identified | Not specifically reported for collagenous gastritis cases | Band of subepithelial collagen ≥10 μm in thickness (diagnostic criterion); thickness varies from minimum of 10 μm to 100 μm and more | Not applicable (but association with microscopic colitis of colon noted in 21.2% of cases) | - Band of subepithelial collagen ≥10 μm in thickness - Background of chronic, often atrophic gastritis - Moderately to markedly thickened collagen table underlying the surface epithelium - Abundant mononuclear infiltrate in the lamina propria - When collagenous thickening affects the corpus, variable degrees of atrophy frequently found - Collagen creeps down into interglandular spaces - Mononuclear infiltrate with scattered eosinophils present | - Recent reports suggest beneficial therapeutic role for budesonide - No specific treatment details provided for the 204 cases in this study | - Not reported for individual cases - Natural course uncertain | Not reported | - Prevalence: 204 cases out of 1,781,005 unique patients (0.01% or approximately 1 in 8,700 patients who had esophagogastroduodenoscopy with gastric biopsies) - Clinical awareness dismal: suspected in only 11 patients and confirmed in 1 of them (most patients who were suspected had either microscopic colitis or celiac disease) - Diagnosis rests entirely on histopathologic findings - No characteristic clinical or endoscopic features allow clinical diagnosis or suspicion - Association with microscopic colitis may serve as useful sentinel diagnosis |
| Ref(94) | 25 | Male | Yes - improved after 1 month of treatment | Yes | No | Yes (hemoglobin 8.1 g/dL) | Not reported | Dyspepsia - improved after 1 month of treatment; gastrointestinal bleeding - improved after 1 month of treatment | No autoimmune disease history | Not specifically mentioned | Not specifically mentioned | Not specifically mentioned | Gastric atrophy and friability; bleeding | Not reported | Thickened subepithelial collagen layer (specific measurement not provided) | Not affected | - Collagenous gastritis - Glandular atrophy - Eosinophilic gastritis - 38 to >100 eosinophils per high-power field in gastric body - Confirmed eosinophilic gastritis on secondary review: 38–100 eosinophils in five high-power fields - Dense and diffuse lamina propria inflammatory cell infiltrates rich in plasma cells and eosinophils - Atrophic mucosa with thickened subepithelial collagen layer highlighted on trichrome staining | - PPI twice daily - Budesonide twice daily - Elimination diet (dairy) | - Time to first follow-up endoscopy: Pending - Clinical course: Improved abdominal pain, dyspepsia, and gastrointestinal bleeding after 1 month; continuing treatment | Not available - follow-up endoscopy pending | - Improved abdominal pain, dyspepsia, and gastrointestinal bleeding after 1 month with treatment - Continuing treatment at time of report |
| Ref(94) | 32 | Male | Yes - ongoing gastrointestinal bleeding and pain on budesonide, then improved after adding PPI and diet | No | No | Yes (hemoglobin 8.5 g/dL) | Not reported | Dyspepsia - improved after adding PPI and diet; gastrointestinal bleeding - ongoing on budesonide alone, improved after adding PPI and diet; constipation | Low Immunoglobulin A (53 mg/dL) | Yes - diffuse gastric nodularity at initial and 3-month follow-up endoscopy | Not specifically mentioned | Not specifically mentioned | Gastric polyps at initial endoscopy; interval gastric ulcer at 3 months which healed at 7 months | Not reported | Thickened subepithelial collagen layer (specific measurement not provided); reduced collagen deposition but still present at 12 months | Not affected | - Initial: Collagenous gastritis; eosinophils not mentioned in initial pathology report but confirmed on secondary review: 39–66 eosinophils in five high-power fields - Follow-up at 3 months: Collagenous gastritis; eosinophilic gastritis; eosinophils in terminal ileum - Secondary review of second biopsy: 40–100 eosinophils in five high-power fields | - Budesonide three times daily initially - Added PPI twice daily after 3 months - Elimination diet (dairy and gluten) | - Budesonide three times daily initially - Added PPI twice daily after 3 months - Elimination diet (dairy and gluten) | - At 12 months: Reduced collagen deposition but still present - Eosinophilic gastritis persisted - Eosinophils in terminal ileum noted | - Ongoing gastrointestinal bleeding and pain on budesonide monotherapy - Improved after adding PPI and elimination diet - Interval gastric ulcer at 3 months healed at 7 months - Reduced but persistent collagen deposition at 12 months |
| Ref(94) | 19 | Female | Yes - improved with treatment | Yes - improved with treatment | No | No (hemoglobin 12.6 g/dL) | Not reported | Dyspepsia; gastrointestinal bleeding - improved with treatment; delayed gastric emptying; small intestinal bacterial overgrowth | - Possible mast cell activation syndrome - Food allergies | Yes - gastric nodularity at initial endoscopy; diffuse gastric nodularity at 8-month follow-up | Yes - gastric erythema | Not specifically mentioned | Gastric polyps | Not reported | Thickened subepithelial collagen layer (specific measurement not provided); unchanged at 8 months | Not affected | - Initial: Collagenous gastritis; eosinophils noted up to 67 eosinophils per high-power field in gastric body 4 years prior to collagenous gastritis diagnosis; confirmed eosinophilic gastritis on secondary review: 62–87 eosinophils in five high-power fields - Follow-up at 8 months: Collagenous gastritis; no duodenal abnormality - Secondary review of second biopsy: 32–35 eosinophils in five high-power fields | - Histamine H2-receptor antagonist - PPI - Cromolyn at diagnosis - Added budesonide for 2 months | - Time to first follow-up endoscopy: 8 months - Clinical course: Improved abdominal pain, gastrointestinal bleeding, and nausea | - At 8 months: Collagen deposition unchanged - Eosinophil counts decreased from 62–87 to 32–35 in five high-power fields - No duodenal abnormality | - Eosinophilic gastritis diagnosed 4 years prior to collagenous gastritis - Improved abdominal pain, gastrointestinal bleeding, and nausea with treatment - Unchanged collagen deposition at 8 months - Decreased eosinophil counts at 8 months |
| Ref(94) | 74 | Male | Yes - resolved with treatment | No | Yes | Yes (hemoglobin 12.7 g/dL) | Not reported | Dyspepsia - resolved with treatment; weight loss - resolved with treatment | No autoimmune disease history | Not specifically mentioned | Not specifically mentioned | Not specifically mentioned | Gastropathy; gastric polyp; enteropathy | Not reported | Thickened subepithelial collagen layer (specific measurement not provided) | Yes - concurrent collagenous colitis | - Collagenous gastritis with intestinal metaplasia - Collagen enterocolitis (duodenum and colon affected) - Confirmed eosinophilic gastritis on secondary review: 41–94 eosinophils in five high-power fields | Stopped Tribenzor (olmesartan-amlodipine-hydrochlorothiazide) - patient was on this medication for 5–10 years, considered possible drug trigger | - Time to first follow-up endoscopy: Pending - Clinical course: Resolved abdominal pain, dyspepsia, and weight loss | Not available - follow-up endoscopy pending | - Only patient with subepithelial collagen deposition elsewhere in gastrointestinal tract beyond stomach (concurrent collagenous colitis and collagenous sprue) - Resolved abdominal pain, dyspepsia, and weight loss after stopping olmesartan - Olmesartan considered possible drug trigger |
| Ref(94) | 24 | Female | Yes - improved with treatment | No | Yes - improved with treatment (alternating diarrhea and constipation) | No (hemoglobin 13.7 g/dL) | Not reported | Dyspepsia - improved with treatment; constipation - improved with treatment (alternating diarrhea and constipation) | - Eczema - HLA-DQ genes positive; high risk antigen DQ2.5 (associated with celiac disease) | Yes - gastric nodularity | Not specifically mentioned | Not specifically mentioned | Gastric atrophy | Not reported | Thickened subepithelial collagen layer (specific measurement not provided) | Not affected | - Collagenous gastritis - Eosinophilic gastritis - 34 to 70 eosinophils per high-power field in gastric body - Confirmed eosinophilic gastritis on secondary review: 34–56 eosinophils in five high-power fields | - PPI twice daily - Probiotics - Elimination diet (dairy) | - Time to first follow-up endoscopy: Pending - Clinical course: Improved dyspepsia and alternating diarrhea and constipation | Not available - follow-up endoscopy pending | - History of nonsteroidal anti-inflammatory drug gastritis - On fluoxetine (considered possible drug trigger) - Improved dyspepsia and alternating diarrhea and constipation with treatment |
| Ref(94) | 21 | Female | No | No | No | Yes (hemoglobin 10.7 g/dL) | Not reported | Gastrointestinal bleeding; delayed gastric emptying ruled out | Low Immunoglobulin A (60 mg/dL) | Yes - gastric nodularity at initial endoscopy; progressed to diffuse friability and nodularity at 11-month follow-up | Not specifically mentioned | Not specifically mentioned | Gastric friability; blood | Not reported | Thickened subepithelial collagen layer (specific measurement not provided); progressed at 11 months | Not affected | - Initial: Collagenous gastritis; gland atrophy; eosinophilic gastritis - Confirmed eosinophilic gastritis on secondary review: 54–58 eosinophils in five high-power fields - Follow-up at 11 months: Collagenous gastritis; gland atrophy; eosinophilic gastritis - Secondary review of second biopsy: 36–98 eosinophils in five high-power fields | - PPI twice daily initially - Added budesonide three times daily after 3 months | - Time to first follow-up endoscopy: 3 months - Clinical course: No change in symptoms | - At 11 months: Progressed collagen deposition - Eosinophilic gastritis persisted (eosinophils 36–98 in five high-power fields) - Gland atrophy persisted | - No symptomatic improvement despite treatment - Progression of both endoscopic findings (diffuse friability and nodularity) and histologic findings (collagen deposition) at 11 months - Eosinophil counts variable (54–58 initially, 36–98 at follow-up) |
| Ref(94) | 36 | Male | Yes - improved with treatment | No | No | No (hemoglobin 14.0 g/dL) | Not reported | Dyspepsia - persistent despite treatment; gastrointestinal bleeding - improved with treatment; weight loss | - Childhood asthma - Hyper-eosinophilic syndrome | Not specifically mentioned at initial endoscopy (reported as normal) | Not specifically mentioned | Not specifically mentioned | Normal initial endoscopy; mild gastropathy at 3 months and 8 months; improved gastropathy at 12 months | Not reported | Thickened subepithelial collagen layer (specific measurement not provided); reduced collagen deposition but still present at 12 months | Not affected | - Initial: Collagenous gastritis; eosinophilic gastritis - 36 to 45 eosinophils per high-power field in stomach; eosinophils in duodenum (up to 50 eosinophils per high-power field) - Confirmed eosinophilic gastritis on secondary review: 36–40 eosinophils in five high-power fields - Follow-up: Collagenous gastritis; eosinophilic gastritis; eosinophils in duodenum - Secondary review of subsequent biopsies: 36–62 eosinophils on second biopsy; <30 eosinophils on third and fourth biopsies | - PPI daily - Elimination diet (dairy) | - Time to first follow-up endoscopy: 3 months - Additional follow-ups at 8 months and 12 months - Clinical course: Improved abdominal pain and gastrointestinal bleeding; persistent dyspepsia | - At 12 months: Reduced collagen deposition and eosinophils but still present - Eosinophil counts decreased to <30 in third and fourth biopsies - Improved gastropathy at 12 months | - Improved abdominal pain and gastrointestinal bleeding - Persistent dyspepsia despite treatment - Histologic improvement: reduced collagen deposition and eosinophil counts at 12 months - Eosinophils normalized (<30 per high-power field) on third and fourth biopsies |
| Ref(94) | 43 | Female | Yes - improved with treatment | Yes - ongoing episodic nausea and vomiting despite treatment | No | Yes (hemoglobin 11.3 g/dL) | Not reported | Gastrointestinal bleeding | - Seasonal allergies - Asthma | Yes - gastric nodularity | Not specifically mentioned | Not specifically mentioned | Thickened gastric folds; endoscopic eosinophilic esophagitis (Los Angeles Grade A reflux esophagitis) | Not reported | Thickened subepithelial collagen layer (specific measurement not provided) | Not affected | - Collagenous gastritis - Eosinophilic gastritis - scattered eosinophils in lamina propria of gastric mucosa - Confirmed eosinophilic gastritis on secondary review: 42–96 eosinophils in five high-power fields | PPI | - Time to first follow-up endoscopy: Pending - Clinical course: Ongoing episodic nausea and vomiting; improved abdominal pain | Not available - follow-up endoscopy pending | - Improved abdominal pain - Persistent episodic nausea and vomiting despite treatment |
| Ref(95) | 16 | Male | Yes - 1 year of abdominal pain | Not reported | No | Yes - severe iron deficiency anemia (undetectable ferritin, partially responsive to oral iron therapy); hemoglobin 6 mg/dL on presentation with hemorrhagic shock | Not reported | Syncopal event; hemorrhagic shock (heart rate 129, blood pressure 70/40 with orthostasis); recurrent gastrointestinal hemorrhage; had self-discontinued PPI 2 weeks before hemorrhagic shock presentation | - Suspected autoimmune-mediated subepithelial collagen deposition - No specific autoimmune diseases mentioned | Yes - nodular gastritis; diffusely nodular mucosa; diffuse patchy gastric nodularity | Yes - erythematous nodular gastropathy | Not specifically mentioned | Thickened mucosal folds; linear depressions (serpiginous depressions); 5 mm gastric ulcer with stigmata of recent extravasation requiring hemoclip placement; cherry red spots; actively bleeding visible vessel at site of prior hemorrhage (recurrent bleeding at same location); gastric ulceration (7.5% of pediatric collagenous gastritis cases have gastric ulceration based on large cohort study) | Negative - negative *H. pylori* testing; no evidence of *H. pylori* on pathology | Increased subepithelial collagen deposition >10 μm (up to 45 μm on Masson trichrome stain) | Not affected - lower endoscopy unremarkable | - Index endoscopy (2 months before hemorrhagic shock): Nonspecific chronic active inflammation; slightly increased eosinophilia in the superficial lamina propria (initially concerning for pediatric eosinophilic gastritis) - Second endoscopy (during hemorrhagic shock admission): Chronic active gastritis; increased subepithelial collagen deposition >10 μm compatible with collagenous gastritis (up to 45 μm); scattered entrapped capillaries and inflammatory cells within collagen; partial denudation of surface epithelium; no evidence of *H. pylor*i or celiac sprue - Follow-up endoscopy (3 months later): Biopsies of raised nodular mucosa showed mild nonspecific chronic gastritis; biopsies of depressed portions showed subepithelial collagen without evidence of lymphocytic gastritis - Later follow-up: Improvement of collagenous gastritis on biopsies despite worsening clinical symptoms - Collagen deposition may only be found on review of multiple level sections or areas (found in depressed rather than nodular areas in this case) - Abnormal vascular patterns with blind endings and irregular caliber changes that heighten bleeding risk | - Initial treatment: Omeprazole 20 mg twice daily; advised to avoid dairy; oral iron supplementation - After hemorrhagic shock: Intravenous PPI; intravenous iron; transfusion of 3 units of packed red blood cells in first 24 hours; budesonide 9 mg crushed in olive oil for gastric retention (in addition to PPI and supplemental iron) - For recurrent bleeding: Epinephrine injection; cauterization; application of hemostatic spray; hemoclip placement - Prophylactic intervention: Super selective prophylactic embolization targeting the hemoclip at site of recurrent bleeding area - Step-up therapy: Vedolizumab 300 mg intravenous infusion every 8 weeks; tapered off budesonide; gradually tapered down PPI and supplemental iron | - Follow-up endoscopy at 3 months: Erythematous nodular gastropathy with depressions and cherry red spots, improved from prior - 7 months after first intensive care unit admission: Second gastric hemorrhage from actively bleeding vessel at same site as first bleed - After prophylactic embolization and vedolizumab: Hemoglobin normalization; no further hemorrhage; clinical stabilization; remains in good health | - At 3 months: Mild nonspecific chronic gastritis in raised nodular mucosa; subepithelial collagen in depressed portions; continued gross gastric involvement - Later follow-up: Improvement of collagenous gastritis on biopsies despite worsening clinical symptoms | - Previously in good health with age-appropriate growth/weight; no additional nutrient deficiencies - 1 year of abdominal pain and severe iron deficiency anemia before hemorrhagic shock - Presented with hemorrhagic shock requiring intensive care unit admission - First such case of hemorrhagic shock due to collagenous gastritis described in literature - Only 22.5% of pediatric collagenous gastritis cases have overt gastrointestinal bleeding based on large cohort study - Only 7.5% have gastric ulceration - Refractory to first-line therapy (iron and PPI) - Required step-up therapy with vedolizumab - Recurrent hemorrhage at same site 7 months after first bleed - Required super selective prophylactic embolization - Clinical improvement with vedolizumab: able to taper budesonide, PPI, and iron supplementation - Clinical response to treatment did not correlate with histologic response (histologic improvement despite worsening clinical symptoms initially) - Remains in good health after vedolizumab initiation |
| Ref(96) | 10 | Male | No - no abdominal pain at presentation | No | No | Yes - iron deficiency anemia (hemoglobin 76 g/L [normal 120–150]; mean corpuscular volume 62.6 fL [77–95]; red cell distribution width 17.6% [11.5–14.5]; serum iron 1.97 μmol/L [9.5–29.9]; ferritin <1 μg/L [12.8–454]; total iron binding capacity 68.6 μmol/L [46.2–69.6]) | Not reported | Pallor of skin and mucous membranes; no constitutional symptoms; no visible blood loss | - No autoimmune disease detected - Testing for autoimmune gastritis negative (anti-parietal cell antibodies 3.01 U/mL [<20.0]) - Collagenous gastritis may be associated with coeliac disease, type 1 diabetes, and autoimmune thyroiditis (more common in collagenous colitis in adults, but not found in this case) | Yes - congested and nodular mucosa in gastric body; persistent nodular mucosa in gastric body at 4-year follow-up | Yes - congested mucosa in gastric body | No - no erosions or ulcers | Antrum and fundus unaffected; scar on posterior wall of duodenal bulb; normal mucosa in second portion of duodenum (D2); nodular/pseudopolypoid appearance at terminal ileum; no abnormalities in colonic mucosa; endoscopic findings mimicked *H. pylori* gastritis | Negative - excluded by negative Giemsa staining, Polymerase Chain Reaction testing, and culture | Subepithelial collagen deposition exceeding 10 μm in thickness in gastric body mucosa | No subepithelial collagen deposition | - Initial evaluation: Collagenous gastritis and duodenitis diagnosed; subepithelial collagen deposition exceeding 10 μm in thickness in gastric body and bulb mucosa; inflammatory infiltrate predominantly lymphocytic; active mucosal inflammation - Follow-up at 4 years: Inactive chronic gastritis; chronic duodenitis with subepithelial collagen deposition; relatively stable compared to initial evaluation - Terminal ileum: Minimally active chronic inflammatory changes; no subepithelial collagen deposition - Colon: No abnormalities; no subepithelial collagen deposition | - Oral iron supplementation (6 mg/kg/day) - No other pharmacological treatment (no PPIs, corticosteroids, or other medications mentioned) | - After 12 weeks of oral iron supplementation: Hemoglobin improved to 120 g/L - After 4 months of oral iron supplementation: Anemia resolved - 4-year follow-up period: No recurrence of anemia observed; patient remained asymptomatic throughout - Re-evaluation by upper gastrointestinal endoscopy at follow-up: Persistent nodular mucosa in gastric body - Histopathological examination at follow-up: Inactive chronic gastritis and chronic duodenitis with subepithelial collagen deposition, relatively stable across both evaluations | - At 4-year follow-up: Inactive chronic gastritis (changed from active to inactive) - Chronic duodenitis with subepithelial collagen deposition remained relatively stable - Collagen deposition persisted despite clinical resolution | - Medical history: Asymptomatic idiopathic splenomegaly detected in first year of life, extensively investigated, regressed clinically by age 5 years - Weight at evaluation: 34.4 kg (50th percentile); height: 149.3 cm (50th–85th percentile) - Detailed nutritional diet history excluded inadequate intake of iron-rich foods or excessive cow's milk consumption - C-reactive protein levels normal - Diagnostic workup for coeliac disease negative (IgA 1.92 g/L [0.5–2.2]; anti-transglutaminase IgA antibodies 0.8 U/mL [<10.0]) - Faecal occult blood negative; faecal calprotectin 21 μg/g (<50) - Abdominal ultrasound: Slightly enlarged spleen 120×40×109 mm (95th percentile for body height: 118 cm) - Technetium-99m scintigraphy excluded Meckel's diverticulum - Complete resolution of anemia with iron supplementation alone after 4 months - No recurrence during 4-year follow-up - Remained asymptomatic throughout follow-up period - Favorable clinical course despite persistent histological findings (nodular mucosa and collagen deposition) - Iron supplementation alone effective for anemia resolution - No long-term complications reported - Clinical remission achieved without need for additional pharmacological interventions - Histological remission not achieved (persistent collagen deposition and nodular mucosa) - Case demonstrates that clinical improvement can occur without histological resolution |
| Ref(97) | 82 | Female | Not reported | Not reported | Yes - chronic diarrhea described as loose, nonbloody, and intermittent, occurring several times per day | Not reported (complete blood cell count within normal limits) | Not reported | Worsening acid reflux symptoms (long-standing history that worsened during past couple of years); unintentional weight loss | - No specific autoimmune diseases mentioned - Medical history includes: chronic kidney disease, coronary artery disease, type 2 diabetes mellitus, hypothyroidism, hypertension, dyslipidemia | Not specifically mentioned | Yes - erythematous gastric mucosa; mild gastric erythema with congestion on prior endoscopies | Not specifically mentioned | Multiple esophagogastroduodenoscopies showed normal esophageal mucosa; relatively nonspecific endoscopic findings; colonoscopy 3 years earlier showed normal colonic mucosa; repeat ileocolonoscopy revealed colonic diverticulosis and 3 small tubular adenomas without endoscopic or histologic evidence of inflammation | Negative - gastric biopsies revealed chronic inactive gastritis without *H. pylori* | Subepithelial collagen band thickening >10 μm | Normal - random biopsy specimens from colon unremarkable; colonoscopy showed no endoscopic or histologic evidence of inflammation | - Gastric biopsies: Moderate chronic inflammation with reactive epithelial changes; subepithelial collagen band thickening (>10 μm); chronic inactive gastritis without *H. pylori* on prior biopsies - Duodenal biopsies: Unremarkable - Terminal ileum biopsies: Unremarkable - Colonic biopsies: Unremarkable; no histologic evidence of inflammation (despite finding 3 small tubular adenomas) - Isolated gastric involvement (helped exclude other causes of chronic diarrhea) | - Prior to diagnosis: PPI therapy twice daily; linaclotide (discontinued 1 year ago due to diarrhea); psyllium; loperamide (all with incomplete symptom relief) - After diagnosis: Oral budesonide 9 mg daily initially; developed oral candidiasis (thrush); treated with nystatin; budesonide dose reduced to 6 mg daily; continued PPI therapy; nutritional support - Planned taper: Reduce budesonide to 3 mg daily, followed by gradual taper based on clinical response, with goal of discontinuing therapy or maintaining lowest effective dose | - After 8-week course of budesonide 6 mg daily: Achieved symptomatic relief and weight stabilization - Follow-up endoscopy initially planned to assess histologic response and guide further management - Patient elected to defer repeat endoscopy after extensive discussion - Repeat endoscopy may be reconsidered if symptoms recur - Long-term clinical monitoring planned to detect potential relapses | Not available - patient deferred repeat endoscopy | - Previously under care of gastroenterologist with presumed diagnoses of gastroesophageal reflux disease and irritable bowel syndrome–mixed type - Long-standing history of acid reflux that worsened during past couple of years - Denied use of nonsteroidal anti-inflammatory drugs, alcohol, caffeine, or tobacco - Prior evaluation included multiple esophagogastroduodenoscopies - Contrast-enhanced computed tomography scan of abdomen and pelvis unremarkable - Laboratory tests (complete blood cell count, liver panel, thyroid-stimulating hormone) within normal limits - Infectious stool studies negative - Fecal calprotectin elevated at 278 mg/g (supporting presence of mucosal inflammation, though nonspecific) - Developed oral candidiasis on budesonide 9 mg daily - Successfully treated with dose reduction to 6 mg daily - Achieved symptomatic relief and weight stabilization after 8-week course - Given chronic nature of collagenous gastritis, long-term clinical monitoring essential |
| Ref(98) | 15 | Male | Yes | Not reported | Not reported | Yes (no specific values provided) | Not reported | No other symptoms mentioned | Not mentioned | Yes - striking nodularity in gastric body and fundus; smooth, peninsula-shaped areas; nodules varied in size; persistent diffuse nodularity on follow-up endoscopy; some nodules became larger and displayed flattened, disc-like appearance | Not specifically mentioned | Not mentioned | - Narrow-band imaging (NBI) with magnification: Widened glandular structures with rod-shaped openings; glandular atrophy around the nodules; mild irregularity in microvascular patterns - Endocytoscopy: Regular arrangement of small pit-like epithelial cells with spiny configuration; glandular lumens appeared slit-like with pale-staining nuclei | Not mentioned | Thickened subepithelial collagen layer (approximately 60 μm) in gastric body | Not mentioned | - Gastric body biopsy: Thickened (approximately 60 μm) subepithelial collagen layer; associated inflammatory cell infiltration in the lamina propria - Masson's trichrome staining: Positive for collagen (blue) - Immunohistochemistry results:   - Desmin: Positive (smooth muscle cells ^+^)  - SMA (smooth muscle actin): Positive (smooth muscle cells ^+^)  - CD34: Positive (vascular endothelium cells ^+^)  - S100: Negative (−)  - Ki67: Partially cells positive (+)  - Pepsinogen I: Positive (chief cells ^+^)  - AE1/AE3: Positive (epithelial cells ^+^)  - CK7: Positive (epithelial cells ^+^)  - H-K-ATPase: Positive (parietal cells ^+^)  - Elastin fibers present in vascular elastic layer | 2-month course of PPIs (with no notable improvement on follow-up endoscopy) | - Follow-up endoscopy after 2 months of PPIs: Persistent diffuse nodularity in gastric body and fundus; some nodules became larger and displayed flattened, disc-like appearance; no notable improvement compared with previous examination - Currently under regular endoscopic follow-up | Not available - no histological follow-up mentioned | - Diagnosis made based on clinical, endoscopic, and histopathological findings - No response to 2-month course of PPIs - Currently under regular endoscopic follow-up - No mention of clinical improvement or symptom resolution |
| Ref(99) | 15 | Male | Yes - chronic abdominal pain (2-year history) | Not reported | Yes - diarrhea (2-year history) | Yes - iron deficiency anemia (hemoglobin level 85 g/L 1 year before diagnosis, mean corpuscular volume 68 fL, MCH 19); microcytic hypochromic anemia; ferritin level 3 µg/L (lowest recorded) | Not reported | Significant fatigue; intermittent constipation; occasional bloating; chronic tiredness and low energy; pallor; no history of gastrointestinal bleeding, weight loss, or fever | - No autoimmune diseases reported in the patient - Past medical history notable for allergic rhinitis - No autoimmune diseases reported in family - Article notes high frequency of inheritance for autoimmune disorders and autoantibodies in Swedish cohort, with occasional early development of autoimmune comorbidities | Yes - marked gastric mucosal nodularity; nodular, polypoid stomach | Not specifically mentioned | Not mentioned | Thickened mucosal folds; small polypoid lesions predominantly in antral region; esophageal mucosa grossly normal; colonic mucosa grossly normal; extensive mucosal involvement confirmed by endoscopy | Negative - *H. pylori* microorganisms not detected with hematoxylin and eosin staining and modified Giemsa stain for bacteria | Subepithelial band of collagen thicker than 10 μm; ultrastructurally irregularly distributed collagen fibers; marked increase in thickness of subepithelial band | Not affected | - Gastric mucosa:   - Noticeable fibrosis of lamina propria  - Upgrowth of smooth muscle from muscularis mucosa in some areas of apparent atrophy  - Surface epithelium partially detached in certain regions  - Neutrophils focally infiltrated lamina propria  - Visible bands of hyalinized collagen partly continuous below surface epithelium or at level of foveolae  - Collagen bands contained entrapped capillaries, lymphocytes, and focal eosinophils  - Collagen bands blue when stained with Masson trichromic staining  - Overlying epithelium appeared normal in some areas and shorter/more disconnected in others  - No increase in white blood cells within epithelium  - Occasional nonprogressive patchy atrophic gastritis   - Hyperplastic polyp:   - Elongated, architecturally distorted pits with outpouchings  - Cystic dilation and papillary formation  - No dysplasia  - Dilated glands on Movat pentachrome staining   - Duodenal mucosa:   - Minimal chronic inflammation  - Intraepithelial lymphocytosis exceeding 30 lymphocytes per 100 epithelial cells (confirmed using CD3 antibody)  - Lymphocytes typically apically located in at least several villi  - No villous blunting (corresponding to Marsh I)   - Electron microscopy findings:   - Marked increase in thickness of subepithelial band  - Haphazardly distributed collagen fibers  - Within band: capillaries, lymphocytes, scattered eosinophils, mast cells, fibroblasts  - Surface epithelium separation from membrane  - Enlargement of intercellular gaps  - Localized reduction in mucous granules  - No notable changes in fibroblasts near glands and foveolae entrapped in subepithelial collagen bundles  - Some degranulating eosinophils in superficial and deep lamina propria  - Apparent reduplication of basal membrane in places | - Before diagnosis: Oral iron supplementation (ineffective); intravenous iron infusions (necessitated by ineffective oral supplementation) - After diagnosis: Lansoprazole 30 mg twice daily; famotidine 40 mg once daily; oral iron supplementation continued - Rationale for combination therapy: Addition of H2 blocker (famotidine) improved symptoms; reduces percentage of time with intragastric pH < 4 and nocturnal acid spikes/breakthroughs - Options not pursued: Vedolizumab infusions and topical budesonide not considered because patient improved with combination of PPI and H2 blocker, as well as iron therapy; planned if clinical symptomatology worsens | - At 12 months following therapy: Hemoglobin level 123 g/L; ferritin level 9 µg/L (while on iron supplementation) - Clinical improvement noted - Initial hemoglobin before assessment: 130 g/L; mean corpuscular volume 72 fL; ferritin 9 µg/L - Swedish study noted high frequency of recurrent iron deficiency in pediatric collagenous gastritis patients | Not available - no follow-up endoscopy or histology mentioned | - 2-year history of chronic abdominal pain, diarrhea, and iron deficiency anemia - Symptoms progressively worsened - Previous oral iron supplementation ineffective - Necessitated intravenous iron infusions - Body mass index 16.02 kg/m² - Weight 50 kg (25th percentile) - Height 178 cm (88th percentile) - Pale but otherwise appeared well - Abdominal examination unremarkable (no tenderness, organomegaly, or distension) - No peripheral stigmata of chronic disease - Inflammatory markers (C-reactive protein, erythrocyte sedimentation rate) within normal limits - Fecal calprotectin normal (74 µg/g) - Celiac serology (anti-tissue transglutaminase antibodies) negative - Stool studies negative for occult blood, parasites, and pathogens - Serum calprotectin and amyloid A levels unremarkable or within normal range - Clinical improvement with treatment - Currently stable on lansoprazole, famotidine, and iron supplementation |
| Ref(100) | 16 | Female | Yes - 7-year history of recurrent abdominal pain | Haematemesis - yes | Not mentioned | Yes - mild iron-deficiency anemia (hemoglobin 106 g/L, Fe 5.1 μmol/L, ferritin 5.6 μg/L) | Not mentioned | Melena (black, tarry stools indicating upper GI bleeding); gastric mucosa bleeding; upper gastrointestinal bleeding typical of pediatric cases | - No autoimmune diseases mentioned - Autoimmune antibodies negative - History of allergic conditions: allergic to penicillin and cephalosporin antibiotics; urticaria; allergic rhinitis - Elevated IgE level (IgE 478.0 KU/L) | Yes - nodular gastritis; smooth peninsula-shaped areas of corpus | Not specifically mentioned | Not mentioned | Discontinuous mucosal folds of corpus; gastric mucosa bleeding described once; normal antrum; no remarkable findings on capsule endoscopy and colonoscopy | - Not mentioned - No evidence for specific pathogen infection | Several patchy-thickened subepithelial collagen depositions observed at corpus (specific thickness not quantified) | Not mentioned; capsule endoscopy and colonoscopy unremarkable | - Initial biopsies (multiple times): Increase of eosinophils in lamina propria (up to 100/HPF) - initially diagnosed as eosinophilic gastritis - Final biopsies:   - Focal infiltrate of eosinophils in lamina propria (>50/HPF at antrum; >30/HPF at corpus)  - Several patchy-thickened subepithelial collagen depositions at corpus  - Eosinophil-rich pattern - could be easily confused with eosinophilic gastritis   1. Peripheral blood eosinophil count negative 2. Tumour markers negative | - Initial treatment (when misdiagnosed as eosinophilic gastritis): Oral prednisolone - Outcomes of initial treatment: Symptoms relapsed twice after weaning of prednisolone - After correct diagnosis:   - Oral iron supplement  - PPIs  - Topically targeted budesonide in open-capsule formulation: Starting from 3 mg three times a day with subsequent tapering and long-term maintenance  - Interventional procedures: Sclerosant injection and titanium clip for gastric mucosa bleeding | - Monthly follow-up - After 1 month of therapy: Normal complete blood count - Clinical remission for 7 months (as of manuscript writing) | Not mentioned - no follow-up histology reported | - 7-year history from symptom onset to correct diagnosis - Tortuous course from presentation to diagnosis - Initially misdiagnosed as eosinophilic gastritis due to eosinophil-rich pattern - Multiple previous oesophagogastroduodenoscopies - Multiple biopsies taken over time - Treated with oral prednisolone based on initial misdiagnosis - Symptoms relapsed twice after weaning of prednisolone - Gastric mucosa bleeding requiring interventional procedures (sclerosant injection and titanium clip) - After correct diagnosis and appropriate treatment: achieved clinical remission |
| Ref(101) | 13 | Male | No | Yes - nausea and vomiting for 1 month | No | Yes - severe iron deficiency anemia (hemoglobin 4.8 g/dL, mean corpuscular volume 51) | Not mentioned | Pallor; presyncope; no symptoms of GI blood loss | - No history of food allergies or asthma - No autoimmune diseases mentioned | Not mentioned | Yes - diffuse erythema throughout gastric mucosa initially; patchy erythema throughout gastric body and antrum on repeat endoscopy | Not mentioned | Normal macroscopic appearance of esophagus and duodenum | Not mentioned | - Patchy increased subepithelial collagen; subepithelial collagen entrapped superficial lamina propria capillaries - Specific thickness not quantified | Not assessed | - Initial biopsy: Increased chronic inflammation in lamina propria; patchy increased subepithelial collagen; subepithelial collagen entrapped superficial lamina propria capillaries (highlighted by Trichrome stain) - Repeat endoscopy (5.5 months): Chronic gastritis; patchy subepithelial collagen consistent with collagenous gastritis | - Two units of packed red blood cells - Oral iron supplementation - PPI daily | - After 6 weeks of therapy: Iron deficiency anemia and all symptoms resolved - Repeat endoscopy after 5.5 months of therapy: Similar pathology to initial specimen | Repeat endoscopy (5.5 months): Chronic gastritis and patchy subepithelial collagen, consistent with collagenous gastritis (similar to prior pathology) | - Previously healthy with no significant medical history - Laboratory studies: Hemoglobin 4.8 grams per deciliter, mean corpuscular volume 51 - Hemoccult stool testing negative - Fecal calprotectin 43 micrograms per gram (normal) - Successfully controlled with PPI alone - Clinical response to treatment good |
| Ref(101) | 15 | Male | Yes - generalized abdominal pain developed at follow-up | Yes - nausea | No | Yes - severe iron deficiency anemia (hemoglobin 6.4 grams per deciliter, mean corpuscular volume 55) | Not mentioned | Pallor; shortness of breath; fatigue; dizziness; chest pain; no symptoms of gastrointestinal blood loss | - Anxiety and depression - No history of food allergies or asthma - No autoimmune diseases mentioned | Not mentioned | Yes - diffuse severe erythema in cardia and fundus; diffuse inflammation of stomach; diffuse erythema and congestion throughout stomach on repeat endoscopy | Yes - nonbleeding erosions throughout fundus; erosions on repeat endoscopy | Esophageal congestion and longitudinal markings; normal colonoscopy | Not mentioned | Not specified in micrometers (described as "focal thickening of the subepithelial collagen" and "focal increased subepithelial collagen deposition") | Not assessed (colonoscopy performed but no collagen findings mentioned) | - Initial: Mild to moderate chronic inflammation including lymphocytes with patchy lamina propria eosinophils (initially diagnosed as eosinophilic gastritis) - Repeat biopsy: Prominent mixed chronic inflammation including patchy eosinophils with focal thickening of the subepithelial collagen (formal diagnosis of collagenous gastritis made) - Subepithelial collagen entrapping inflammatory cells and superficial capillaries - Two subsequent endoscopies showed persistent moderate to severe gastritis with continued patchy collagen deposition | - PPI (initially for eosinophilic gastritis diagnosis) - Oral iron supplementation - One unit packed red blood cells transfusion - Intermittent ferrous sulfate for iron deficiency | - Weaned off PPI after 6.5 months of therapy - Two subsequent endoscopies performed | Persistent moderate to severe gastritis with continued patchy collagen deposition on two subsequent endoscopies | - Initially diagnosed with eosinophilic gastritis, later re-diagnosed as collagenous gastritis - Minimal histologic response to PPI therapy - Required intermittent use of ferrous sulfate for iron deficiency - Stool hemoccult negative, calprotectin 182 mCg/g |
| Ref(101) | 14 years old at presentation (initial anemia at 12 years old, referred to gastroenterology 2 years later) | Male | Not mentioned | Not mentioned | Not mentioned | Yes (iron deficiency anemia, hemoglobin 6.1 g/dL, mean corpuscular volume 57 fL; persistent hypoferritinemia requiring intravenous iron repletion) | Not mentioned | Pallor, fatigue, constipation | - None mentioned - History of attention deficit hyperactivity disorder, asthma - Allergy to tree nuts | Not mentioned | Yes (moderate erythema in the gastric body initially; diffuse erythema and friability in the entire stomach on repeat endoscopy; diffuse gastritis on third endoscopy) | Not mentioned | Perianal skin tags found on colonoscopy; juvenile polyp in sigmoid colon; prominent folds noted | Negative | Not specified in micrometers (described as "patchy subepithelial collagen deposition") | No pathology findings consistent with inflammatory bowel disease; colonoscopy showed perianal skin tags and juvenile polyp but no collagenous colitis mentioned | - Initial: Chronic inactive gastritis and increased eosinophils in gastric biopsies (initially diagnosed as eosinophilic gastritis) - Repeat biopsy: Chronic inactive gastritis and patchy subepithelial collagen deposition (diagnosis of collagenous gastritis made) - Third endoscopy: Persistent collagenous gastritis | - Oral iron supplementation - PPI twice daily (initially for eosinophilic gastritis) - Intravenous iron repletion intermittently - Gluten-free diet attempted but not sustained - Budesonide and sucralfate offered but declined | - Anemia resolved after 2 months initially - Referred to gastroenterology 2 years after initial presentation - Repeat endoscopy at 3.5 months - Repeat endoscopy at 18 months | - Repeat endoscopy 3.5 months later showed chronic inactive gastritis and patchy subepithelial collagen deposition - Endoscopy at 18 months showed persistent collagenous gastritis | - Initially diagnosed with eosinophilic gastritis, later re-diagnosed as collagenous gastritis - Blood transfusion deferred - Fecal calprotectin 1100 mCg/g initially, 375 mCg/g on repeat - Currently managed on intravenous iron intermittently - Minimal response to PPI therapy |
| Ref(101) | 14 years old at presentation (initial anemia at 12 years old, referred to gastroenterology 2 years later) | Male | Not mentioned | Not mentioned | Not mentioned | Yes (iron deficiency anemia, hemoglobin 9.2 g/dL, mean corpuscular volume 66 fL; refractory anemia requiring intravenous iron) | Not mentioned | Screened after twin brother found to have profound iron deficiency anemia; remained asymptomatic | - None mentioned - No history of asthma - Allergy to tree nuts and shrimp | Not mentioned | Yes (moderate gastritis in the gastric body initially; erythema in the gastric antrum on repeat endoscopy; diffuse erythema on third endoscopy) | Not mentioned | Prominent gastric folds in gastric antrum on repeat endoscopy and in gastric body and antrum on third endoscopy; normal colonoscopy | Not mentioned | Not specified in micrometers (described as "focal subepithelial collagen deposition") | Colonoscopy normal, no collagenous colitis mentioned | - Initial: Chronic gastritis with focal subepithelial collagen deposition with mixed chronic inflammation with eosinophil predominance (diagnosed as eosinophilic gastritis) - Repeat biopsy: Mixed lymphocytic and eosinophilic inflammation with focal subepithelial collagen deposition (collagenous gastritis diagnosed) - Third endoscopy: Collagenous gastritis with lymphoplasmacytic inflammation | - Oral iron supplementation - PPI twice daily (initially for eosinophilic gastritis) - Intravenous iron intermittently - Completed 6 months of PPI therapy - Gluten-free diet trialed but not sustained - Budesonide and sucralfate offered but declined | - Repeat endoscopy 3.5 months later - Endoscopy 18 months later | - Repeat endoscopy showed mixed lymphocytic and eosinophilic inflammation with focal subepithelial collagen deposition - Endoscopy at 18 months showed collagenous gastritis with lymphoplasmacytic inflammation | - Initially diagnosed with eosinophilic gastritis, later re-diagnosed as collagenous gastritis - Remained asymptomatic - Stool hemoccult testing negative - Fecal calprotectin 144 mCg/m initially, 74 mCg/m on repeat - Currently managed on intravenous iron intermittently - Minimal histologic response to PPI therapy |

Table Abbreviation Legend:

*H. pylori*, *Helicobacter pylori*; μm, micrometers; IgG, Immunoglobulin G; IgA, Immunoglobulin A; CLO, Campylobacter-like organism; 13C, Carbon-13; HLA-DR, Human Leukocyte Antigen-DR; CD25^+^, Cluster of Differentiation 25 positive; IEL, Intraepithelial lymphocytes; M0/M2/M17/M18/M20/M26, Month 0/2/17/18/20/26; H2, Histamine-2; MCV, Mean Corpuscular Volume; fL, femtoliters; GI, gastrointestinal; CD4^+^, Cluster of Differentiation 4 positive; CD8^+^, Cluster of Differentiation 8 positive; CD79a^+^, Cluster of Differentiation 79a positive; IgM, Immunoglobulin M; κ, kappa (light chain); λ, lambda (light chain); Bcl-2, B-cell lymphoma 2; CMV, Cytomegalovirus; IV, intravenous; PPIs, proton pump inhibitors; Hb, hemoglobin; H&E, Hematoxylin and Eosin; WBC, White Blood Cell count; MPL, IgM Phospholipid units; EU/ml, ELISA Units per milliliter; U/mL, Units per milliliter; C3, Complement component 3; CH50, Total hemolytic complement; C4, Complement component 4; ASCA, Anti-Saccharomyces cerevisiae antibodies; OmpC, Outer membrane porin C; pANCA, perinuclear Anti-Neutrophil Cytoplasmic Antibodies; LUQ, Left Upper Quadrant; EMA, Endomysial Antibodies; NSAIDs, Nonsteroidal Anti-Inflammatory Drugs; TSH, Thyroid-Stimulating Hormone; HLA DQ2, Human Leukocyte Antigen DQ2; 5-ASA, 5-Aminosalicylic acid; 5-HIAA, 5-Hydroxyindoleacetic acid; HIV, Human Immunodeficiency Virus; EGD, Esophagogastroduodenoscopy; IgE, Immunoglobulin E; CT, Computed Tomography; ANCA, Anti-Neutrophil Cytoplasmic Antibodies; MT, Masson Trichrome; H2RA, Histamine-2 Receptor Antagonist; CGD, Collagenous Gastroduodenitis; SLE, Systemic Lupus Erythematosus; BMI, Body Mass Index; SD, Standard Deviation; UBT, Urea Breath Test; PCR, Polymerase Chain Reaction; ANA, Antinuclear Antibodies; HLA-DQ8, Human Leukocyte Antigen DQ8; IBD, Inflammatory Bowel Disease; anti-LKM, anti-Liver Kidney Microsomal antibodies; CBC, Complete Blood Count; CD3, Cluster of Differentiation 3; MCH, Mean Corpuscular Hemoglobin; M-NBI, Magnifying endoscopy with Narrow-Band Imaging; TIBC, Total Iron-Binding Capacity; IDA, Iron Deficiency Anemia; IgG4, Immunoglobulin G4; HPF, High-Power Field; CRP, C-Reactive Protein; ESR, Erythrocyte Sedimentation Rate; CA 19-9, Cancer Antigen 19-9; EUS, Endoscopic Ultrasound; GERD, Gastroesophageal Reflux Disease; JIA, Juvenile Idiopathic Arthritis; CVID, Common Variable Immunodeficiency; AE, Autoimmune Encephalitis; CD19^+^, Cluster of Differentiation 19 positive; CD16^+^/56^+^, Cluster of Differentiation 16/56 positive; NK cells, Natural Killer cells; IgM^−^IgD^−^, Immunoglobulin M negative/Immunoglobulin D negative; anti-NMDAR, anti-N-Methyl-D-Aspartate Receptor; anti-LGI1, anti-Leucine-rich Glioma-Inactivated 1; anti-GABA-B, anti-Gamma-Aminobutyric Acid B; anti-DPPX, anti-Dipeptidyl-Peptidase-like Protein-6; anti-IgLON-5, anti-IgLON Family Member 5; anti-PCA, anti-Purkinje Cell Antibody; anti-sox-1, anti-SRY-box Transcription Factor 1; anti-CRMP-5, anti-Collapsin Response Mediator Protein-5; anti-Ma, anti-Ma (onconeural antibody); anti-Tr, anti-Tr (Purkinje cell antibody); SIFT, Sorting Intolerant From Tolerant; IVIG, Intravenous Immunoglobulin; A1AT, Alpha-1 Antitrypsin; CD27^+^, Cluster of Differentiation 27 positive; CD31, Cluster of Differentiation 31; PET CT, Positron Emission Tomography-Computed Tomography; EBV, Epstein-Barr Virus; EBER ish, EBV-related Encoded RNA in situ Hybridization; EBV-VCA, Epstein-Barr Virus Viral Capsid Antigen; EBNA, Epstein-Barr Nuclear Antigen; EBV-EA, Epstein-Barr Virus Early Antigen; SNRI, Serotonin and Norepinephrine Reuptake Inhibitor; EGF, Epidermal Growth Factor; FGF19, Fibroblast Growth Factor 19; TNF, Tumor Necrosis Factor; IL6, Interleukin 6; IFNG, Interferon Gamma; TGFB1, Transforming Growth Factor Beta 1; 13CU-breath test, Carbon-13 Urea Breath Test; ME-BLI, Magnifying Endoscopy with Blue Laser Imaging; MCE, Marginal Crypt Epithelium; anti-TPO, anti-Thyroid Peroxidase; FCM, Ferric Carboxymaltose; MAdCAM-1, Mucosal Addressin Cell Adhesion Molecule-1; IQR, Interquartile Range; TTG, Tissue Transglutaminase; COVID-19, Coronavirus Disease 2019; OGD, Oesophagogastroduodenoscopy; OLGA, Operative Link on Gastritis Assessment; ENA, Extractable Nuclear Antigen; anti-Sm, anti-Smith antibodies; OIT, Oral Iron Therapy; D2, Second portion of duodenum; NBI, Narrow-Band Imaging; SMA, Smooth Muscle Actin; CD34, Cluster of Differentiation 34; S100, S100 protein; AE1/AE3, AE1/AE3 cytokeratin; CK7, Cytokeratin 7; H-K-ATPase, Hydrogen-Potassium ATPase.

**References**

1.	Colletti RB, Trainer TD. Collagenous gastritis. Gastroenterology. 1989;97(6):1552-5.

2.	Côté JF, Hankard GF, Faure C, Mougenot JF, Holvoet L, Cézard JP, et al. Collagenous gastritis revealed by severe anemia in a child. Hum Pathol. 1998;29(8):883-6.

3.	Pulimood AB, Ramakrishna BS, Mathan MM. Collagenous gastritis and collagenous colitis: a report with sequential histological and ultrastructural findings. Gut. 1999;44(6):881-5.

4.	Vesoulis Z, Lozanski G, Ravichandran P, Esber E. Collagenous gastritis: a case report, morphologic evaluation, and review. Mod Pathol. 2000;13(5):591-6.

5.	Groisman GM, Meyers S, Harpaz N. Collagenous gastritis associated with lymphocytic colitis. J Clin Gastroenterol. 1996;22(2):134-7.

6.	Stolte M, Ritter M, Borchard F, Koch-Scherrer G. Collagenous gastroduodenitis on collagenous colitis. Endoscopy. 1990;22(4):186-7.

7.	Borchard F, Niederau C. [Collagenous gastroduodenitis]. Dtsch Med Wochenschr. 1989;114(35):1345.

8.	Lagorce-Pages C, Fabiani B, Bouvier R, Scoazec JY, Durand L, Flejou JF. Collagenous gastritis: a report of six cases. Am J Surg Pathol. 2001;25(9):1174-9.

9.	Meunier S, Villard F, Bouvier R, Lachaux A, Bertrand Y. [Collagen gastritis, an unusual cause of anemia in children. Report of 2 cases]. Arch Pediatr. 2001;8(1):47-50.

10.	Freeman HJ. Topographic mapping of collagenous gastritis. Can J Gastroenterol. 2001;15(7):475-8.

11.	Winslow JL, Trainer TD, Colletti RB. Collagenous gastritis: a long-term follow-up with the development of endocrine cell hyperplasia, intestinal metaplasia, and epithelial changes indeterminate for dysplasia. Am J Clin Pathol. 2001;116(5):753-8.

12.	Stancu M, De Petris G, Palumbo TP, Lev R. Collagenous gastritis associated with lymphocytic gastritis and celiac disease. Arch Pathol Lab Med. 2001;125(12):1579-84.

13.	Kajino Y, Kushima R, Koyama S, Fujiyama Y, Okabe H. Collagenous gastritis in a young Japanese woman. Pathol Int. 2003;53(3):174-8.

14.	Wang HL, Shah AG, Yerian LM, Cohen RD, Hart J. Collagenous gastritis: an unusual association with profound weight loss. Arch Pathol Lab Med. 2004;128(2):229-32.

15.	Park S, Kim DH, Choe YH, Suh YL. Collagenous gastritis in a Korean child: a case report. J Korean Med Sci. 2005;20(1):146-9.

16.	Dray X, Reignier S, Vahedi K, Lavergne-Slove A, Marteau P. Collagenous gastritis. Endoscopy. 2007;39 Suppl 1:E292-3.

17.	Kori M, Cohen S, Levine A, Givony S, Sokolovskaia-Ziv N, Melzer E, et al. Collagenous gastritis: a rare cause of abdominal pain and iron-deficiency anemia. J Pediatr Gastroenterol Nutr. 2007;45(5):603-6.

18.	Kamimura K, Kobayashi M, Narisawa R, Watanabe H, Sato Y, Honma T, et al. Collagenous gastritis: endoscopic and pathologic evaluation of the nodularity of gastric mucosa. Dig Dis Sci. 2007;52(4):995-1000.

19.	Ravikumara M, Ramani P, Spray CH. Collagenous gastritis: a case report and review. Eur J Pediatr. 2007;166(8):769-73.

20.	Jain R, Chetty R. Collagenous gastritis. Int J Surg Pathol. 2010;18(6):534-6.

21.	Suskind D, Wahbeh G, Murray K, Christie D, Kapur RP. Collagenous gastritis, a new spectrum of disease in pediatric patients: two case reports. Cases J. 2009;2:7511.

22.	Brain O, Rajaguru C, Warren B, Booth J, Travis S. Collagenous gastritis: reports and systematic review. Eur J Gastroenterol Hepatol. 2009;21(12):1419-24.

23.	Leung ST, Chandan VS, Murray JA, Wu TT. Collagenous gastritis: histopathologic features and association with other gastrointestinal diseases. Am J Surg Pathol. 2009;33(5):788-98.

24.	Wilson C, Thompson K, Hunter C. Nodular collagenous gastritis. J Pediatr Gastroenterol Nutr. 2009;49(2):157.

25.	Billiémaz K, Robles-Medranda C, Le Gall C, Gay C, Mory O, Clémenson A, et al. A first report of collagenous gastritis, sprue, and colitis in a 9-month-old infant: 14 years of clinical, endoscopic, and histologic follow-up. Endoscopy. 2009;41 Suppl 2:E233-4.

26.	Gopal P, McKenna BJ. The collagenous gastroenteritides: similarities and differences. Arch Pathol Lab Med. 2010;134(10):1485-9.

27.	Macaigne G, Boivin JF, Harnois F, Chayette C, Dikov D, Cheaib S, et al. [Collagenous gastritis and ileo-colitis occurred in autoimmune context: report of a case and review of the literature]. Gastroenterol Clin Biol. 2010;34(8-9):e1-6.

28.	Jin X, Koike T, Chiba T, Kondo Y, Ara N, Uno K, et al. Collagenous gastritis. Dig Endosc. 2013;25(5):547-9.

29.	Mandaliya R, DiMarino AJ, Abraham S, Burkart A, Cohen S. Collagenous Gastritis a Rare Disorder in Search of a Disease. Gastroenterology Res. 2013;6(4):139-44.

30.	Tanabe J, Yasumaru M, Tsujimoto M, Iijima H, Hiyama S, Nishio A, et al. A case of collagenous gastritis resembling nodular gastritis in endoscopic appearance. Clin J Gastroenterol. 2013;6(6):442-6.

31.	Soeda A, Mamiya T, Hiroshima Y, Sugiyama H, Shidara S, Dai Y, et al. Collagenous gastroduodenitis coexisting repeated Dieulafoy ulcer: A case report and review of collagenous gastritis and gastroduodenitis without colonic involvement. Clin J Gastroenterol. 2014;7(5):402-9.

32.	Al-Kandari A, Al-Alardati H, Sayadi H, Al-Judaibi B, Mawardi M. An unusual case of collagenous gastritis in a middle- aged woman with systemic lupus erythromatosis: a case report. J Med Case Rep. 2014;8:278.

33.	Koide T, Mochizuki T, Kawai N, Yashiro K, Inoue T, Tsujimoto M, et al. Collagenous gastroduodenitis with recurrent gastric ulcer in 12-year-old girl. Pediatr Int. 2015;57(4):754-7.

34.	Ma C, Park JY, Montgomery EA, Arnold CA, McDonald OG, Liu TC, et al. A Comparative Clinicopathologic Study of Collagenous Gastritis in Children and Adults: The Same Disorder With Associated Immune-mediated Diseases. Am J Surg Pathol. 2015;39(6):802-12.

35.	Arnason T, Brown IS, Goldsmith JD, Anderson W, O'Brien BH, Wilson C, et al. Collagenous gastritis: a morphologic and immunohistochemical study of 40 patients. Mod Pathol. 2015;28(4):533-44.

36.	Hangard P, Lasfargue M, Rubio A. [Collagenous gastritis and colitis in a 10-year-old girl]. Arch Pediatr. 2016;23(7):747-50.

37.	Bajwa RU, Joshi A, Heikenen JB. Successful Treatment of Collagenous Gastritis in a Child With a Gluten-Free Diet. Wmj. 2015;114(6):271-3.

38.	Mandaliya R, Burkart AL, DiMarino AJ, Rattan S, Cohen S. Association between common variable immunodeficiency and collagenous infiltrative disorders of the gastrointestinal tract: A series of four patients. Indian J Gastroenterol. 2016;35(2):133-8.

39.	Appelman MH, de Meij TG, Neefjes-Borst EA, Kneepkens CM. Spontaneous Gastric Perforation in a Case of Collagenous Gastritis. APSP J Case Rep. 2016;7(1):7.

40.	Lázaro de Lucas C, Tesouro Rodríguez L, Magallares García LN, Martínez-Ojinaga Nodal E, Ramos Boluda E. [Collagenous gastritis: An unusual atypical form in a male infant]. An Pediatr (Engl Ed). 2018;88(4):225-6.

41.	Vinsard DG, Mejia Perez LK, Nassar A, Vazquez Roque MI. Collagenous Gastritis and Helicobacter pylori Infection: A Mere Coincidence? ACG Case Rep J. 2017;4:e69.

42.	Anwar MS, Aamar A, Marhaba A, Sidhu JS. Collagenous Gastritis in a Young Female With IgA Deficiency. Gastroenterology Res. 2017;10(2):126-7.

43.	Matta J, Alex G, Cameron DJS, Chow CW, Hardikar W, Heine RG. Pediatric Collagenous Gastritis and Colitis: A Case Series and Review of the Literature. J Pediatr Gastroenterol Nutr. 2018;67(3):328-34.

44.	Hayashi S, Nishida T, Adachi S, Mima A, Inada M. Collagenous gastritis: a rare disease with distinctive endoscopic findings. Gastrointest Endosc. 2018;88(1):186-7.

45.	Eke CB, Brown RA, De Lacy RJ, Pillay K, Goddard EA. Collagenous gastritis: An unusual cause of generalized oedema in a child. J Trop Pediatr. 2019;65(3):305-8.

46.	Zamani F, Boghratian A, Zare Mehrjardi A, Naserifar F, Vafaeimanesh J. Collagenous Gastritis, a Rare Cause of Dyspepsia Resistant to Treatment; A Case Report. Middle East J Dig Dis. 2018;10(4):263-6.

47.	Akkari I, Skandrani K, Abdelkader AB, Mrabet S, Jazia EB. Anemia revealing a collagenous gastritis in a young Tunisian man. Pan Afr Med J. 2018;30:231.

48.	Lim HW, Wong BY, Elkowitz D, Sultan K. An elderly patient's complete response to steroid therapy for collagenous gastritis. Ther Adv Chronic Dis. 2018;9(8):143-6.

49.	Kawasaki K, Fujita Y, Uesugi N, Sugai T, Matsumoto T. Collagenous Gastritis Observed by Magnifying Narrow-Band Imaging Endoscopy. J Gastrointestin Liver Dis. 2018;27(1):7.

50.	De Ronde O, Delos M, Jespers S, Gillain C, De Ronde T. Collagenous gastritis : about two paediatric cases and literature review. Acta Gastroenterol Belg. 2020;83(1):41-5.

51.	Anderson B, Ravi K. Chronic Abdominal Pain Caused by Collagenous Gastritis. Clin Gastroenterol Hepatol. 2019;17(12):e144.

52.	Varela Rey V, Alfonso Labandeira R, Martinón Torres N, Crujeiras Martínez V. [Celiac disease and collagenous gastritis: A rare combination in childhood]. An Pediatr (Engl Ed). 2019;90(5):325-6.

53.	Lee YJ, Lee M, Kim DJ, Lee S, Hong J. Three case reports of collagenous gastritis in children: Lessons for an endoscopic and histologic approach to mucosal nodularity of the stomach. Medicine (Baltimore). 2019;98(11):e14870.

54.	Liao CH, Saddik M, Ip S. An Unusual Case of Collagenous Gastritis: Incidental Finding in a Patient Presenting with Dysphagia. Case Rep Gastrointest Med. 2019;2019:5427085.

55.	Käppi T, Wanders A, Wolving M, Lingblom C, Davidsson Bården B, Arkel R, et al. Collagenous Gastritis in Children: Incidence, Disease Course, and Associations With Autoimmunity and Inflammatory Markers. Clin Transl Gastroenterol. 2020;11(8):e00219.

56.	Cortez N, Berzosa M, Jacobs A, Bloom M. Collagenous Gastritis: An Unusual Presentation With Tubular Shaped Stomach. J Investig Med High Impact Case Rep. 2020;8:2324709620944695.

57.	Singh S, Jing E, Shen J. In the Thick of It: The Many Faces of Collagenous Gastritis. Dig Dis Sci. 2020;65(6):1653-5.

58.	Jacob JG, Parratt JDE, Kiely CJ, Fernando SL. Common variable immunodeficiency in association with autoimmune encephalitis, collagenous gastritis, and colitis. Ann Allergy Asthma Immunol. 2021;127(1):137-8.

59.	Beinvogl BC, Goldsmith JD, Verhave M. Pediatric Collagenous Gastritis: Clinical and Histologic Outcomes in a Large Pediatric Cohort. J Pediatr Gastroenterol Nutr. 2021;73(4):513-9.

60.	Kang B, Um SH, Yun J, Kim HK, Choe BH, Lee YM. Collagenous gastroduodenocolitis in a Korean adolescent: first pediatric case report in Asia. Transl Pediatr. 2021;10(11):3096-103.

61.	Genta RM, Turner KO, Morgan CJ, Sonnenberg A. Collagenous gastritis: Epidemiology and clinical associations. Dig Liver Dis. 2021;53(9):1136-40.

62.	Klein J, Wilkins BJ, Verma R, Muir AB. Collagenous Gastritis Masquerading as Eosinophilic Gastritis. ACG Case Rep J. 2021;8(2):e00527.

63.	Kiryukhin AP, Tertychnyy AS, Shcherbakov PL, Inozemtsev AS, Kolokolnikova OA. Isolated gastric Kaposi's sarcoma in a non-AIDS/HIV patient with coexisting collagenous gastritis and microscopic colitis. Korean J Intern Med. 2021;36(6):1530-1.

64.	Narsai T, Su H, Braxton D, Gupta S. Collagenous Gastritis in Primary Selective IgM Deficiency: Transition to EBV+ Gastric Adenocarcinoma. Case Reports Immunol. 2021;2021:5574944.

65.	Çakar S, Çakır Y. Iron deficiency is not always innocent in childhood: a rare diagnosis of collagenous gastritis. Turk J Pediatr. 2022;64(1):147-51.

66.	Choung RS, Sharma A, Chedid VG, Absah I, Chen ZE, Murray JA. Collagenous Gastritis: Characteristics and Response to Topical Budesonide. Clin Gastroenterol Hepatol. 2022;20(9):1977-85.e1.

67.	Ziaei H, Boghratian A, Sohrabi M, Zare Mehrjardi A, Zamani F. Concurrent Collagenous Gastritis and Collagenous Colitis: A Case Presentation and Review of the Literature. Middle East J Dig Dis. 2022;14(1):131-5.

68.	Varelas AI, Fürst S, Langner C. Mixed lymphocytic and collagenous inflammation of the entire gastrointestinal tract under therapy with serotonin and norepinephrine reuptake inhibitors. Virchows Arch. 2022;481(5):779-83.

69.	Mishra A, Chung J, Mani H, Serrano M. Response to Mesalamine Therapy in Pediatric Collagenous Gastritis and Colitis: A Case Report and Review. Glob Pediatr Health. 2022;9:2333794x221094262.

70.	Curci D, Dillon ST, Gu X, Winter H, Libermann TA. Proteome-Wide Analysis Using SOMAscan Identifies and Validates Epidermal Growth Factor as a Disease Marker of Collagenous Gastritis. Gastro Hep Adv. 2022;1(5):689-702.

71.	Leung K, Thung I, Hammami MB. Severe Bleeding Gastric Barotrauma Unmasking Collagenous Gastritis. Clin Gastroenterol Hepatol. 2022;20(6):A18.

72.	Tiftikçi A, Koç ES, Çiçek B. IgG4-related disease: an unusual presentation as collagenous gastritis. Gastrointest Endosc. 2023;98(3):450-1.

73.	Zheng QH, Hu J, Yi XY, Xiao XH, Zhou LN, Li B, et al. Collagenous gastritis in a young Chinese woman: A case report. World J Gastroenterol. 2022;28(41):5993-6001.

74.	Illan Montero J, Viala J, Rebeuh J, Berthet S, Blais R, Caldari D, et al. Collagenous gastritis in children: A national cohort. Arch Pediatr. 2023;30(5):307-13.

75.	Isoldi S, Viola F, Cucchiara S, Dilillo A, Iorfida D, Testi AM, et al. Management of collagenous gastritis in children: Case series and literature review. Indian J Gastroenterol. 2024;43(3):567-77.

76.	Kagihara JE, Boland JL, Colon Rosa G, Mamilla D, Younes M, Borum ML, et al. Collagenous Gastritis: An Atypical Presentation of a Rare Disease. Cureus. 2023;15(2):e34698.

77.	Blackmore C, Leach M. Collagenous Gastritis: A Cause of Pediatric Iron Deficiency Anemia. ACG Case Rep J. 2023;10(2):e01000.

78.	Evans MG, Guccione JP, Crymes A, Li X, Johnson CA, Chandan VS, et al. Atypical Presentations of Collagenous Gastritis Mimicking Celiac Sprue. Case Rep Gastrointest Med. 2023;2023:4073588.

79.	Liu Q, Wang Y, Harpaz N. Coexisting Th1 and Th2 cytokines in patients with collagenous gastritis and implications for its pathogenesis. J Pediatr Gastroenterol Nutr. 2024;78(2):231-40.

80.	Patel PD, Doucette S, Sanvanson P. Clinical and Pathologic Response to Vedolizumab in a Young Female Patient With Collagenous Gastritis. ACG Case Rep J. 2023;10(10):e01175.

81.	Kozai L, Tan A, Nebrejas KE, Warashina C, Nishimura Y. Collagenous Gastritis Is an Underdiagnosed Cause of Anemia and Abdominal Pain: Systematic Scoping Review. Dig Dis Sci. 2023;68(7):3103-14.

82.	Kiran N, Kashi M, Khan S. Association of Collagenous Gastritis With Helicobacter pylori Infection. Cureus. 2023;15(7):e42172.

83.	Romano M, Plott N, Galligan A, Khalaf R. Literature Review and a Relevant Case of Pediatric Collagenous Gastritis: A Rare but Important Etiology of Iron-Deficiency Anemia. JPGN Rep. 2023;4(4):e351.

84.	Wu C, Wang L, Zhang L, Ni Y, Wang Z, Qu Y. A case of collagenous gastritis and literature review: A case report. Medicine (Baltimore). 2023;102(21):e33868.

85.	Camarero Salces C, Enes Romero P, Redondo C, Rizo Pascual JM, Roy Ariño G. Collagenous colitis and collagenous gastritis in a 9 year old girl: a case report and review of the literature. Acta Gastroenterol Belg. 2011;74(3):468-74.

86.	Duncan A, Veli I, Tsosie D, Koffler E. Probable collagenous gastritis via Epstein-Barr virus reactivation in the setting of coronavirus disease 2019: a case report. J Med Case Rep. 2024;18(1):605.

87.	Higashimori A, Maeda N, Kato M, Kohashi K, Fujiwara Y. Olmesartan-induced collagenous gastritis. Lancet. 2024;404(10461):1444.

88.	Arredondo Montero J, Peñafiel-Freire DM, Carracedo Vega E, Antona G, de Oliveira Gomes AS, Bronte Anaut M. Pediatric Collagenous Gastritis: A Case Series With Clinical, Endoscopic and Histopathological Correlation. Clin Pediatr (Phila). 2024;63(5):599-603.

89.	Kiryukhin A, Pavlov P, Shishkina A, Tertychnyy A, Feoktistova K. A case description of collagenous gastritis concurrent with collagenous colitis: visible endoscopic changes. Quant Imaging Med Surg. 2024;14(12):9756-9.

90.	Pinis M, Ziv-Sokolovskaya N, Kori M. Collagenous and lymphocytic gastritis in pediatric patients. A single-center experience observing an increase in diagnosis in recent years. Scand J Gastroenterol. 2024;59(10):1144-50.

91.	Agrawal P, Bhattar K, Rojas C, Larson J. Pediatric Collagenous Gastroduodenitis: A Rare Cause of Iron-Deficiency Anemia. Cureus. 2024;16(11):e72939.

92.	Rodriguez N, Kumar S, Mo J, Hartmann P. Collagenous gastritis with elevated fecal calprotectin in a pediatric patient. JPGN Rep. 2024;5(2):152-7.

93.	Genta RM, Rugge M. Host-related low-prevalence gastritides: Epidemiological and clinical characterization. Dig Liver Dis. 2024;56(10):1683-9.

94.	Shahrvini B, Wang HL, Weiss GA. Collagenous Gastritis in Adults: A Case Series Elucidating the Potential Overlap with Eosinophilic Gastritis. Dig Dis Sci. 2025.

95.	Davis BG, Goldman ML, Cherny SC, Lam AY, Fox JM. Collagenous Gastritis: A Rare Cause of Hemorrhagic Shock. ACG Case Rep J. 2025;12(7):e01762.

96.	Lima Ferreira M, Carvalho Queirós J, Mazeda I, Oliveira AG, Costa E, Wen X, et al. Collagenous Gastritis-A Rare Cause of Iron Deficiency Anaemia. J Paediatr Child Health. 2025;61(9):1516-9.

97.	Liu J, Jones KO, Hammami MB. A Case of Chronic Gastrointestinal Symptoms: Peeling Back the Mucosal Layers. Gastroenterology. 2025;169(7):1350-2.

98.	Wang Y, Zhang H, Chen X. A case of collagenous gastritis: endoscopic and endocytoscopic features. Endoscopy. 2025;57(S 01):E287-e9.

99.	Sergi C, Patry J, Kehar M. Collagenous gastritis, hyperplastic polyp, and intraepithelial duodenal lymphocytosis in a 15-year-old child: An iconographic case report. Medicine (Baltimore). 2025;104(46):e45993.

100.	Ma XY, Cui Y, Fang JY, Sun D. Underdiagnosed cause of gastrointestinal bleeding. Gut. 2025;74(3):386-439.

101.	Rompca A, Davis J, Pitman R, Sparks B. The plot thickens: A pediatric cases series on collagenous gastritis. JPGN Rep. 2025;6(4):368-71.
